# *Gordonia sputi*-associated bloodstream infection in a renal transplant patient with chronic indwelling central venous catheter: a case report and literature review

**DOI:** 10.1099/acmi.0.000560.v3

**Published:** 2023-06-28

**Authors:** Calvin Ka-Fung Lo, Conor Broderick, Aleksandra Stefanovic, William Connors, Melanie Murray

**Affiliations:** ^1^​ Department of Pathology and Laboratory Medicine, University of British Columbia, Vancouver, British Columbia, Canada; ^2^​ Division of Medical Microbiology and Virology, Providence Health Care, St Paul’s Hospital, Vancouver, British Columbia, Canada; ^3^​ Division of Infectious Diseases, University of British Columbia, Vancouver, British Columbia, Canada; ^4^​ Oak Tree Clinic, BC Women’s Hospital, Vancouver, British Columbia, Canada

**Keywords:** 16S rRNA, Actinomycetes, central line-associated bloodstream infection, *Gordonia*

## Abstract

**Introduction.:**

Although rare, human infections caused by *Gordonia* spp. have been reported, especially within the immunocompromised population and those with long-term indwelling devices. We report a case of *Gordonia* spp. bacteraemia in a renal transplant patient and present a literature review on microbiological identification methods of this organism.

**Case Presentation.:**

A 62-year-old female renal transplant recipient admitted to hospital with a 2-month history of dry cough and fevers occurring weekly when receiving electrolyte replacement infusions via a Groshong line. Over 2 weeks, blood cultures repeatedly isolated a Gram-positive bacillus solely in aerobic bottles, and this was initially reported as *Rhodococcus* spp. by the local microbiology laboratory. Chest computed tomography (CT) showed multiple ground-glass lung opacities suggestive of septic pulmonary emboli. As central line-associated bloodstream infection was suspected, empirical antibiotics were initiated and the Groshong line was removed. The Gram-positive bacillus was later confirmed by the reference laboratory as *Gordonia sputi* via 16S rRNA sequencing. Vancomycin and ciprofloxacin for a duration of 6 weeks were completed as targeted antimicrobial therapy. After treatment, the patient remained symptom-free with marked improvement on repeat CT chest imaging.

**Conclusion.:**

This case illustrates the challenges surrounding identification of *Gordonia* spp. and other aerobic actinomycetes. 16S rRNA gene sequencing may be a preferred identification method, especially when initial workup of a weakly acid-fast organism fails to make an identification or shows discrepant results using traditional diagnostic modalities.

## Data Summary

All data generated or analysed during this study are included in this published article and its Supplementary Material. Please see Tables 1 and 2 for data extracted from eligible articles within our literature review.

## Introduction

*Gordonia* spp. are aerobic, Gram-positive, rod-shaped bacteria of the order Actinomycetales. They are ubiquitous in the environment and are regarded as opportunistic pathogens [1].

Although rare, reported infections range from mediastinitis post-sternotomy for cardiac surgery, pneumonia, brain abscesses and meningitis through to various skin and soft tissue infections [2]. Among immunocompromised patients with chronic indwelling catheters and medical devices, *Gordonia* spp. have also been implicated in cases of catheter-related bacteraemia [2].

Frequent misidentification of *Gordonia* spp. as other Gram-positive bacilli (e.g. *Corynebacteria, Nocardia*, *Rhodococcus *) may delay appropriate diagnosis and treatment, including the dismissal of positive blood cultures as contaminating diphtheroids [3–5]. We report here a case of central line-associated bloodstream infection (CLABSI) due to *Gordonia sputi* in a renal transplant recipient that was initially misidentified as *Rhodococcus* spp. by the analytic profile index (API) Coryne system (bioMérieux, Marcy L’Étoile, France). We performed a literature review of human infections caused by *Gordonia* spp., as well as reviewing the diagnostic modalities used to identify the organism and the challenges faced.

## Case presentation

A 62-year-old female renal transplant recipient was admitted to hospital on 20 August 2021, with a 5 week history of recurrent fevers and progressive dry cough without haemoptysis or dyspnoea. She did not report any unintentional weight loss, night sweats, fatigue or altered mentation. She had no recent sick contact exposures (including no prior risk of exposure to tuberculosis) or significant travel history. Her past medical history included deceased donor renal transplant in October 2012, stable on tacrolimus and mycophenolate mofetil without any history of rejection. Other medical and surgical history included workup for chronic hypophosphataemia and hypocalcaemia requiring weekly electrolyte replacement infusions via Groshong line (inserted March 2021), prior hemicolectomy for diverticular disease and parathyroidectomy.

On examination, she was haemodynamically stable with blood pressure of 151/67, pulse rate of 69 beats min^−1^ and body temperature of 36.8 °C. Lung auscultation was normal without any bilateral adventitious sounds, and cardiovascular examination did not reveal any murmurs or other stigmata of infective endocarditis. Groshong line site did not demonstrate purulent discharge or overlying cellulitis. Laboratory investigations showed a peripheral white blood cell count of 5.1×10^9^ l^−1^ (normal range 4.5–11.0×10^9^ l^−1^) with a neutrophil count of 3.4×10^9^ l^−1^ (normal range 2.0–8.0 x 10^9^ l^−1^). Inflammatory markers were elevated with C-reactive protein of 23.2 mg l^−1^ (normal <3.1 mg l^−1^).

**Fig. 1.**
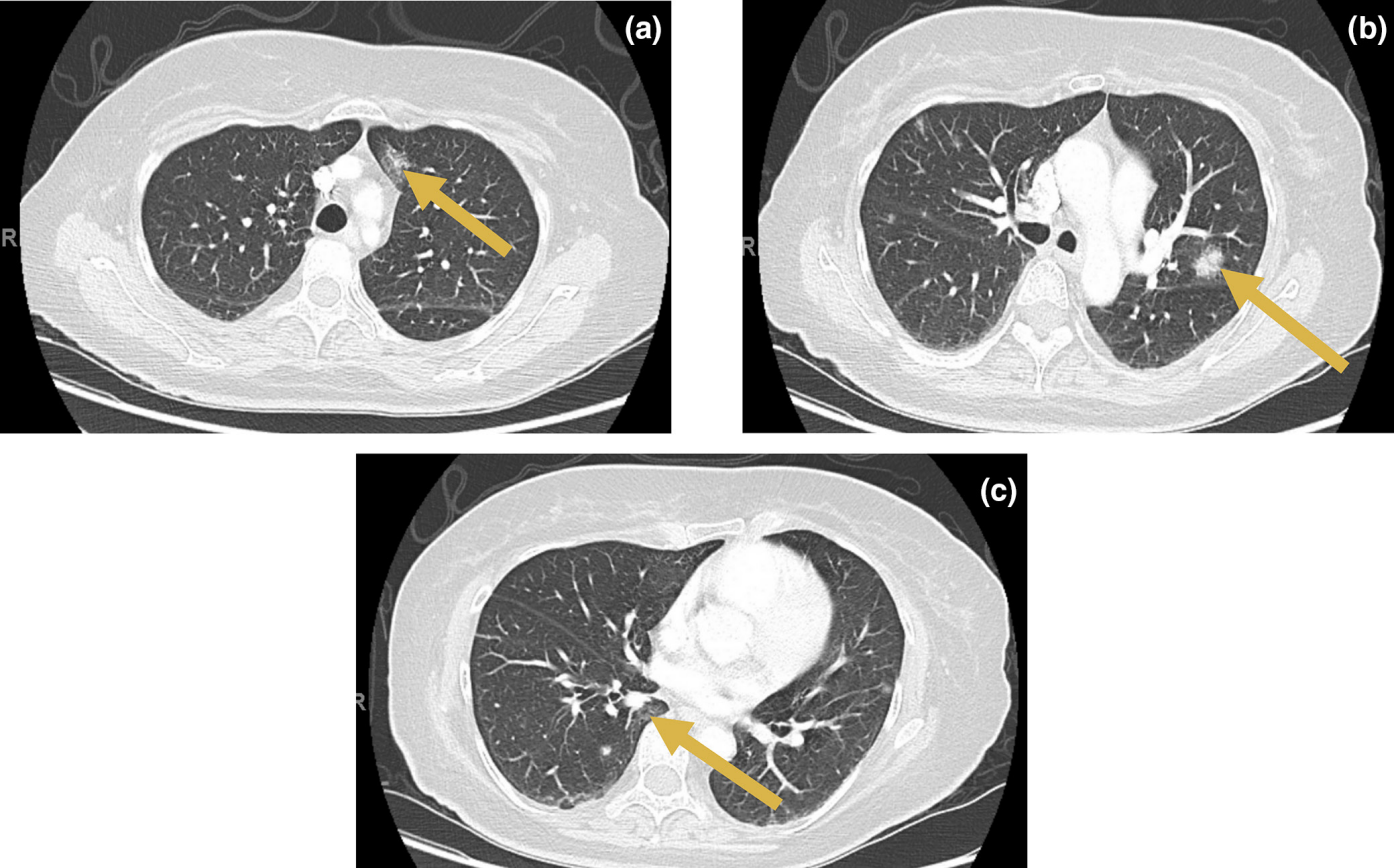
Chest computed tomography (CT) findings with contrast axial views. Yellow arrows: new ground-glass changes, anterior left upper lobe (**a**); solid to ground-glass opacity, posterior left upper lobe (**b**); subpleural ground-glass changes, anterior right lower lobe (**c**).

Chest radiograph followed by whole-body computed tomography (CT) scan revealed multifocal ground-glass opacities suspicious for pulmonary septic emboli, without cavitary lesions (Fig. 1). Transthoracic and subsequent trans-oesophageal echocardiography were negative for vegetations or haemodynamically significant valvular dysfunction.

**Fig. 2.**
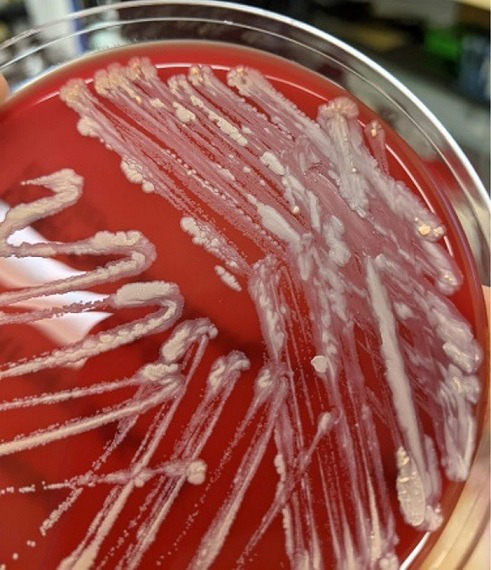
Colony morphology on sheep blood agar plate post-incubation. Beige non-haemolytic colonies, sheep blood agar, 115 h of incubation, 35±2 °C.

**Fig. 3.**
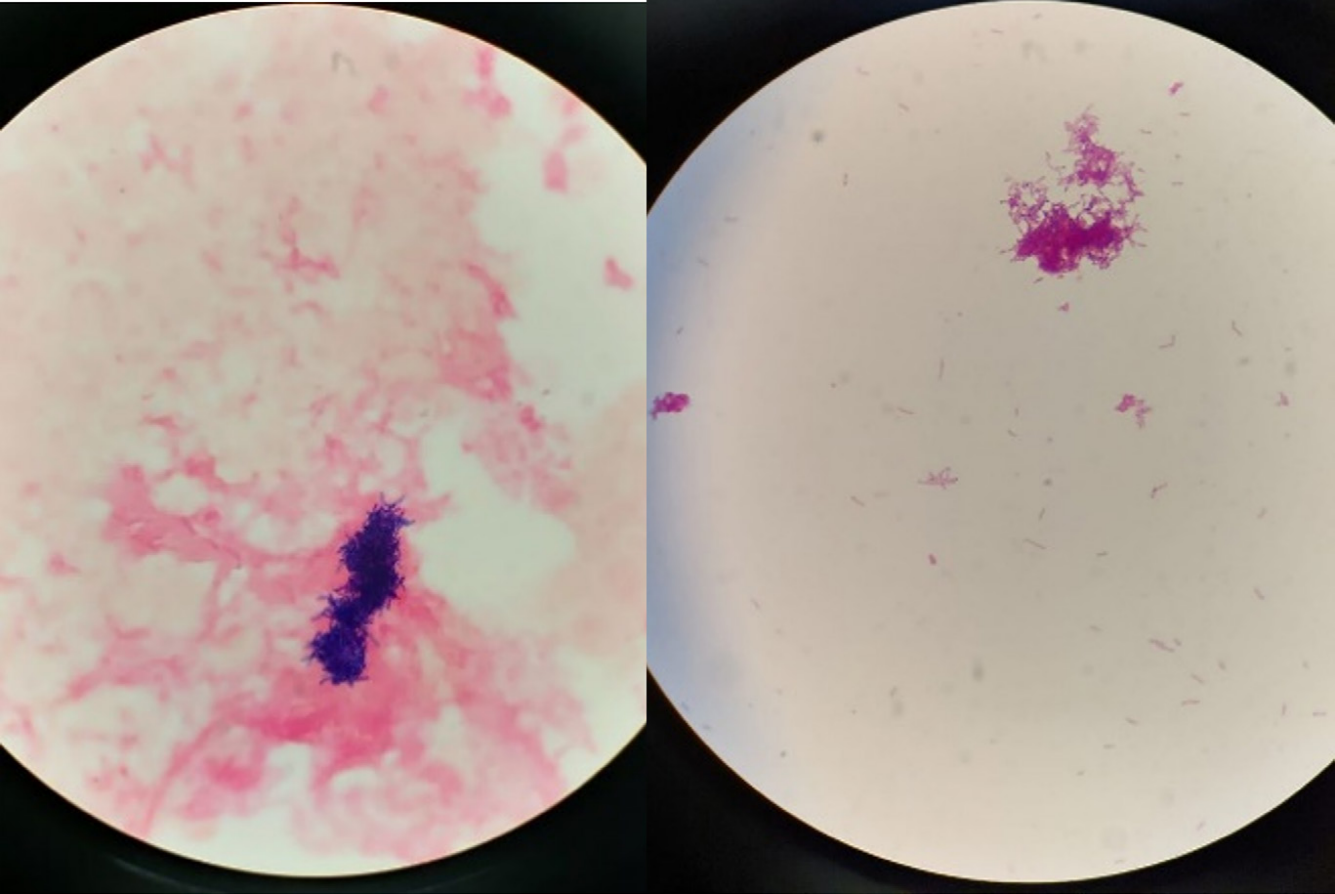
Gram stain of blood culture sample. Demonstration of Gram-positive coccobacilli under light microscopy, 100×.

In the context of recurrent fevers occurring in temporal relation to weekly electrolyte infusions, outpatient blood cultures were initially obtained 2 weeks prior to admission and Gram-positive bacilli were only isolated from the aerobic bottle. The patient was initiated on intravenous (IV) vancomycin. Questions remained as to whether the positive blood culture was attributable to contamination and initially the organism was not identified using MALDI-TOF VITEK MS V3 (bioMérieux, Marcy L’Étoile, France). Subsequent repeat blood cultures collected both from peripheral draw and tunnelled Groshong line over a 2 week period continued to isolate Gram-positive bacilli only in aerobic bottles (Figs 2 and 3). The organism showed beige, non-haemolytic colonies that were mucoid in appearance and adherent to media, catalase-positive and partially acid-fast. In our local microbiology laboratory, API Coryne system (bioMérieux, Marcy L’Étoile, France) gave an identification of *Rhodococcus* spp.

Inoculum was prepared from blood agar after incubating the isolate for 3–5 days at 35 °C (+/− 2 °C), with suspension made to 0.5 McFarland standard. The minimum inhibitory concentrations (MICs) were determined using E-test gradient strip (bioMérieux, France) as part of non-standardized susceptibility testing for this isolate. Findings were interpreted as per Clinical and Laboratory Standards Institute (CLSI) M24 [6]. The results showed susceptibility to amoxicillin/clavulanate, azithromycin, ciprofloxacin, linezolid, penicillin and vancomycin; it was intermediate to doxycycline. Hence, oral ciprofloxacin 500 mg twice daily was added to the patient’s regimen on the third day of admission as combination antimicrobial therapy. As her Groshong line was the suspected source of bacteraemia, it was subsequently removed and the catheter tip bacterial culture isolated Gram-positive bacilli after 4 days of incubation. Repeat blood cultures after 5 days of antibiotics (1 day post-line removal) were negative.

## Diagnosis, treatment and follow-up

As our local diagnostic methods failed to identify an organism matching with grown colony morphology and biochemical properties, the isolate was forwarded to the provincial reference laboratory for 16S rRNA sequencing, where the organism was subsequently identified as *G. sputi.*

Symptom association with Groshong catheter use, suspected septic pulmonary emboli and prompt resolution of bacteraemia following central venous catheter removal were consistent with a diagnosis of CLABSI. Six weeks of IV vancomycin plus oral ciprofloxacin post-line removal were given based on concerns for complicated bacteraemia in the setting of radiographical evidence of suspected pulmonary septic emboli and lack of standardized susceptibility results or treatment standards. The patient was clinically stable and thus discharged home to complete the course of antibiotics. By completion of antibiotics, her fever and dry cough had resolved, and a new Groshong line (placed 8 days post-clearance of blood cultures) was functioning well with no new signs of infection. Repeat CT chest post-treatment showed marked interval improvement of the multifocal ground-glass opacities with only minimal residual left upper lobe parenchymal scarring. The patient remained symptom-free 3 months post-completion of therapy.

## Discussion

Reported risk factors for *Gordonia *-associated infections include implanted catheters and medical devices, active chemotherapy treatment, haematological malignancies, receipt of solid organ or haematopoietic stem cell transplant, or recent surgery [e.g. coronary artery bypass graft (CABG)] [2, 3, 7, 8]. Gram-positive bacilli isolated in blood cultures of patients with clinical suspicion of infection should not be routinely dismissed as contaminating diphtheroids, especially in patients who are immunocompromised or have long-term indwelling catheters or medical devices, or when isolated in multiple culture bottles [3–5].

The number of recognized infections due to *Gordonia* spp. may rise in the future because of the increased use of long-term indwelling central venous catheters and medical devices, extended survival of immunocompromised patients and improved laboratory identification methods. Early genus- or species-level identification of Gram-positive bacilli such as *Gordonia* spp. from cultures of individuals with suspected infections is key for early diagnosis and targeted therapy. However, challenges remain in the ability to identify these organisms accurately and reliably via traditional culture identification methods.

A comprehensive literature review was conducted on all English language articles published on human infections due to *Gordonia* spp. using EMBASE and Ovid MEDLINE from 1946 (inception) to February 2022 (File S1, available in the online version of this article). All cases of human infections with *Gordonia* spp. isolated in cultures, including from case report/series, observational studies, or clinical trials, were included. We excluded any duplicate data, non-English articles, non-human infections, review articles (without primary data) and reports from reference laboratory or public health reports (i.e. those that focused on evaluation and workup of non-clinical isolates). Our search identified 57 eligible articles, of which 92 documented human infections associated with *Gordonia* spp. were reported [1–5, 7–58] (see Table 1 for details).

**Table 1.** Literature review on reported human infections due to *Gordonia* spp

Patient characteristics and variables	Pooled results (*n*=92)
**Age, median (IQR)**	50 years (30–65)
Sex* **Male** **Female**	47/86 (54.7 %) 39/86 (45.3 %)
**Risk factors**	Central venous catheter/haemodialysis lines 29/92 (31.5 %) Peritoneal dialysis 19/92 (20.7 %) Chemotherapy with haematological malignancy 11/92 (15.2 %) Post-operative, e.g. coronary bypass surgery, mitral valve replacement 8/92 (9.0 %) Chemotherapy with solid malignancy 7/92 (7.6 %) Diabetes 7/92 (7.6 %) Solid organ transplant/bone marrow transplant 5/92 (5.4 %)
**Continent**	Asia 35/92 (38.0 %) North America 34/92 (37.0 %) Europe 15/92 (16.3 %) Africa 2/92 (2.2 %) Australia/Oceania 2/92 (2.2 %) South America 1/92 (1.1 %)
**Type of Infection**	Bacteraemia/catheter-related bloodstream infection 33/92 (35.9 %) Peritoneal dialysis-associated peritonitis 14/92 (15.2 %) Skin/soft tissue infections 11/92 (12.0 %) Respiratory infections 10/92 (10.9 %) Central nervous system/ocular 9/92 (9.8 %) Surgical site infections, including sternal wounds 9/92 (9.8 %) Other 6/92 (6.5 %), e.g. septic arthritis (*n*=2), endocarditis (*n*=2), cholecystitis (*n*=1), renal abscess (*n*=1)

*Only included those with reported gender (*n*= 86).

The median age was 50 years (interquartile range 30–65 years) and 54.7 % of patients were male. Reported cases predominantly involved healthcare sites in Asia (38 %) and North America (37 %). Of reported clinical risk factors, 31.5 % of patients had central venous catheter or haemodialysis lines, and 20.7 % were receiving peritoneal dialysis. Approximately a quarter of patients were immunocompromised, including haematological malignancy with active chemotherapy (15.2 %), solid tumours with active chemotherapy (7.6 %) and solid organ or bone marrow transplant (5.4 %). Other risk factors included post-operative status from cardiac surgery (e.g. CABG) 9.0 % and diabetes mellitus (7.6 %).

The most frequently reported *Gordonia* spp.-associated infections were bacteraemia or CLABSI (35.9 %), peritoneal dialysis-associated peritonitis (15.2 %) and skin/soft tissue infections (12.0 %) (Table 1).

For the preliminary identification modality (Table 2), the API Coryne system (bioMérieux, Marcy L’Étoile, France) was most frequently used (20 of 92 isolates; 21.7 %), but 17/20 (85.0 %) species were misidentified as *Rhodococcus.* MALDI-ToF VITEK MS (8 of 92 isolates, 8.7 %) showed more accurate performance, although 2 of the 8 isolates in that subgroup (25.0 %) failed to provide any identification. With MALDI-ToF VITEK MS, six of the eight isolates were successfully identified *Gordonia* spp., although 16S rRNA sequencing was required for all for species-level identification. PCR for the hsp65 gene (3.3 %, three isolates) also showed successful identification up to genus level prior to proceeding to 16S rRNA sequencing for species-level identification. Thirty of the 92 isolates (32.6 %) in our listed studies did not clearly report any diagnostic modality for preliminary identification. For definitive identification of *Gordonia* isolates, 16S rRNA sequencing was the most common reported modality, with 80/92 isolates (87.0 %).

**Table 2.** Initial identification method used per isolate and proportion of misidentification

Method used for identification (ID)	No. of isolates tested	Correct identification	Misidentification	No identification
API Coryne	20 (21.7 %)	1/20 (5 %) had a potential ID of *Gordonia *, but also mixed with *Nocardia *. All 20 isolates required 16S for definitive ID	17 (85 %) – all misidentified as *Rhodococcus *	2 failed to provide clear organism ID
MALDI-TOF/Biotyper	8 (8.7 %)	6 of 8 identified *Gordonia *, with 16S rRNA sequencing for species-level ID	None	2
PCR for hsp65	3 (3.3 %)	All successfully identified *Gordonia* to genus level with hsp65, likewise 16S for species confirmation	None	None
Other	30 (32.6 %) had no reported workup; 31 (33.7 %) based preliminary ID on either biochemical tests or morphology	NA – required 16S for definitive ID	18 presumed *Nocardia, Rhodococcus, Actinomyces* or *Corynebacterium* spp.	na
Total	92	na	na	na

Our clinical case and literature review highlight issues surrounding misidentification of *Gordonia* spp. using conventional methods of identifying modified acid-fast organisms. Based on our review, 16S rRNA sequencing is the preferred identification method.

As 16S rRNA sequencing may not be readily available and requires submission to a reference laboratory, consideration of prioritization of early 16S rRNA sequencing may include: isolates staining weakly acid-fast with discrepant or no initial identification, suspicious characteristics from morphology (e.g. non-motile short Gram-positive rods as opposed to coccobacillary, suggesting non-* Rhodococcus* spp.; absence of aerial hyphae suggesting a non-* Nocardia* spp.), or biochemical results (e.g. positive for urea hydrolysis and nitrate reduction; or unable to grow in the presence of lysozyme, also suggesting a non-* Nocardia* spp.).

There are currently no guidelines for the management of *Gordonia* infections [2] or established breakpoints per the Clinical and Laboratory Standards Institute (CLSI) and the European Committee on Antimicrobial Susceptibility Testing (EUCAST) for *Gordonia* spp. While dual empirical antimicrobial therapy may be considered (e.g. carbapenems, fluoroquinolones, vancomycin), treatment should be individualized and informed by the isolate’s *in vitro* antimicrobial susceptibility test results and clinical response to treatment [3, 39].

Our literature review showed successful treatment using various antimicrobials of varying durations, depending on the type and severity of infection. Recently, the *in vitro* antimicrobial susceptibilities of 13 human isolates of *Gordonia polyisoprenivorans* were reported, where nearly half of the strains were resistant to trimethoprim–sulfamethoxazole [59]. At least for *G. polyisoprenivorans *, some strains were resistant to tigecycline, minocycline and clarithromycin. All isolates were susceptible to amikacin, ampicillin, ceftriaxone, imipenem, amoxicillin–clavulanate, ciprofloxacin, vancomycin and linezolid [59]. With the absence of guidelines, treatment duration and the role of lifelong suppression in immunocompromised hosts or infections with non-removable hardware require further study. Treatment decisions should be based on the host’s underlying immune function, specific immunosuppression and modifiability of infection risk factors.

## Supplementary Data

Supplementary material 1
