# Supplementary material for: Gordonia sputi-associated bloodstream infection in a renal transplant patient with chronic indwelling central venous catheter: a case report and literature review
Source: Access Microbiol. 2023 Jun 28;5(6):acmi000560.v3. doi: 10.1099/acmi.0.000560.v3 (PMC10323808; doi:10.1099/acmi.0.000560.v3)
Supplement: Supplementary material 1 [file acmi-5-560.v3-s001.pdf]

Database(s): **Embase** 1974 to 2022 August 15**, Ovid MEDLINE(R) and Epub Ahead of Print, In-Process, In-Data-Review & Other Non-Indexed Citations, Daily and Versions** 1946 to August 15, 2022

Search Strategy:

#	Searches	Results
1	exp Gordonia/ or gordonia.mp.	1598
2	exp human/ or human.mp.	46083619
3	1 and 2	292
4	bloodstream infection/ or exp infection/ or infection.mp.	7663091
5	3 and 4	190
6	remove duplicates from 5	120

1.

Molecular signature of postmortem lung tissue from COVID-19 patients suggests distinct trajectories driving mortality.

Budhraja A., Basu A., Gheware A., Abhilash D., Rajagopala S., Pakala S., Sumit M., Ray A., Arulselvi S., Mathur P., Nambirajan A., Kumar S., Gupta R., Wig N., Trikha A., Guleria R., Sarkar C., Gupta I., Jain D.

Embase
bioRxiv. (no pagination), 2021. Date of Publication: 08 Nov 2021.

[Preprint]

AN:
2015896824

The precise molecular mechanisms behind life-threatening lung abnormalities during severe SARS-CoV-2 infections are still unclear. To address this challenge, we performed whole transcriptome sequencing of lung autopsies from 31 patients suffering from severe COVID-19 related complications and 10 uninfected controls. Using a metatranscriptome analysis of lung tissue samples we identified the existence of two distinct molecular signatures of lethal COVID-19. The dominant "classical" signature (n=23) showed upregulation of unfolded protein response, steroid biosynthesis and complement activation supported by massive metabolic reprogramming leading to characteristic lung damage. The rarer signature (n=8) potentially representing "Cytokine Release Syndrome" (CRS) showed upregulation of cytokines such IL1 and CCL19 but absence of complement activation and muted inflammation. Further, dissecting expression of individual genes within enriched pathways for patient signature suggests heterogeneity in host response to the primary **infection**. We found that the majority of patients cleared the SARS-CoV-2 **infection**, but all suffered from acute dysbiosis with characteristic enrichment of opportunistic pathogens such as Staphylococcus cohnii in "classical" patients and Pasteurella multocida in CRS patients. Our results suggest two distinct models of lung pathology in severe COVID-19 patients that can be identified through the status of the complement activation, presence of specific cytokines and characteristic microbiome. This information can be used to design personalized therapy to treat COVID-19 related complications corresponding to patient signature such as using the identified drug molecules or mitigating specific secondary infections.
Copyright The copyright holder for this preprint is the author/funder, who has granted bioRxiv a license to display the preprint in perpetuity. It is made available under a CC-BY 4.0 International license.

Status

In-Process

Author NameID

Sarkar, Chitra; ORCID: https://orcid.org/0000-0002-4315-9316 Pakala, Suman; ORCID: https://orcid.org/0000-0001-5156-0823
Ray, Animesh; ORCID: https://orcid.org/0000-0002-9434-5338
Wig, Naveet; ORCID: https://orcid.org/0000-0002-6603-601X
Mathur, Purva; ORCID: https://orcid.org/0000-0003-4429-3688
Kumar, Sachin; ORCID: https://orcid.org/0000-0002-8526-6013

Institution

(Budhraja, Basu, Abhilash, Sumit, Gupta) Department of Biochemical Engineering and Biotechnology, Indian Institute of Technology, New Delhi 110016, India (Gheware, Nambirajan, Sarkar, Jain) Department of Pathology, All India Institute of Medical Sciences, New Delhi 110029, India
(Rajagopala, Pakala) Department of Medicine, Division of Infectious Diseases, Vanderbilt University Medical Center, Nashville, TN 37232, United States
(Ray, Wig) Department of Medicine, All India Institute of Medical Sciences, New Delhi 110029, India
(Arulselvi, Mathur) Department of Laboratory Medicine, JPNATC, All India Institute of Medical Sciences, New Delhi 110029, India

(Kumar) Department of Medical Oncology, All India Institute of Medical Sciences, New Delhi 110029, India
(Gupta) Laboratory Oncology, Dr. B. R. Ambedkar Institute Rotary Cancer Hospital (IRCH), All India Institute of Medical Sciences, New Delhi 110029, India
(Trikha) Department of Anaesthesiology, Critical Care and Pain Medicine, All India Institute of Medical Sciences, New Delhi 110029, India
(Guleria) Department of Pulmonary Medicine and Sleep Disorders, All India Institute of Medical Sciences, New Delhi 110029, India

Publisher

bioRxiv

Emtree Heading

adult; animal experiment; animal model; autopsy; complement activation; complication; controlled study; *coronavirus disease 2019; cytokine release syndrome; female; gene expression;
Gordonia
;
human
; infectious agent; *lung injury; male; microbiome; *mortality; nonhuman; personalized medicine; primary infection; secondary infection; Staphylococcus warneri; steroidogenesis; unfolded protein response; upregulation; whole transcriptome sequencing; endogenous compound; interleukin 1; macrophage inflammatory protein 3beta.

Drug Index Terms

endogenous compound [m]; interleukin 1 [m]; macrophage inflammatory protein 3beta [m].

Other Index Terms

adult [m]; animal experiment [m]; animal model [m]; autopsy [m]; complement activation [m]; complication [m]; controlled study [m]; *coronavirus disease 2019 [m]; cytokine release syndrome [m]; female [m]; gene expression [m]; Gordonia [m]; human [m]; infectious agent [m]; *lung injury [m]; male [m]; microbiome [m]; *mortality [m]; nonhuman [m]; personalized medicine [m]; primary infection [m]; secondary infection [m]; Staphylococcus warneri [m]; steroidogenesis [m]; unfolded protein response [m]; upregulation [m]; whole transcriptome sequencing [m].

Year of Publication

2021

Cite

2.
Sternal osteomyelitis caused by **Gordonia** bronchialis in an immunocompetent patient following coronary artery bypass surgery.

Nwaedozie S., Mojarrab J.N., Gopinath P., Fritsche T., Nasser R.M.

Embase
IDCases. 29 (no pagination), 2022. Article Number: e01548. Date of Publication: January 2022.

[Article]

AN:
2019013697

Skin commensals, especially gram-positive cocci, are the usual microbial organisms that cause post-operative sternal wound infections. Rarely, environmental bacteria such as **Gordonia** spp. have been implicated as etiological agents in post-cardiac procedure surgical site infections. We report a case of a patient who presented with post-coronary artery bypass sternal osteomyelitis caused by this uncommon pathogen, and review relevant medical literature to identify commonalities in presentation, diagnosis and management. Repeat isolation of **Gordonia** bronchialis in the setting of post-procedure wound **infection** should raise suspicion for a real pathogenicity. Definitive identification requires a broad range of bacterial PCR DNA amplification and sequencing followed by susceptibility testing as treatment may require a prolonged course of antibiotics.
Copyright © 2022

Status

Embase

Institution

(Nwaedozie, Mojarrab, Gopinath) Department of Internal Medicine, Marshfield Clinic Health System - Marshfield, Marshfield, WI 54449, United States (Fritsche) Department of Pathology, Marshfield Clinic Health System - Marshfield, Marshfield, WI 54449, United States
(Nasser) Department of Infectious Disease, Marshfield Clinic Health System - Marshfield, Marshfield, WI 54449, United States

Publisher

Elsevier Ltd

Keyword Heading

Gordonia bronchialis, Sternal osteomyelitis, Surgical site infection

Emtree Heading

Actinobacteria; aged; article; *bacterial infection/co [Complication]; *bacterial infection/di [Diagnosis]; *bacterial infection/dt [Drug Therapy]; bacterium identification; case report; clinical article; computer assisted tomography; *coronary artery bypass surgery; coronary artery disease; DNA sequencing; emergency ward; erythema; follow up;

Gordonia
;
human
; human tissue; hyperbaric oxygen therapy; hyperlipidemia; hypertension; *immunocompetence; leukocyte count; leukocytosis; male; nuclear magnetic resonance imaging; *osteomyelitis/co [Complication]; polymerase chain reaction; *postoperative infection/co [Complication]; *postoperative infection/di [Diagnosis]; *postoperative infection/dt [Drug Therapy]; surgical infection; thorax pain; very elderly; wound infection; amoxicillin plus clavulanic acid/dt [Drug Therapy]; amoxicillin plus clavulanic acid/po [Oral Drug Administration]; amoxicillin plus clavulanic acid/pv [Special Situation for Pharmacovigilance]; C reactive protein/ec [Endogenous Compound]; cefazolin/dt [Drug Therapy]; cefazolin/iv [Intravenous Drug Administration]; cefazolin/pv [Special Situation for Pharmacovigilance]; ceftriaxone/dt [Drug Therapy]; ceftriaxone/pv [Special Situation for Pharmacovigilance]; sultamicillin/dt [Drug Therapy]; sultamicillin/iv [Intravenous Drug Administration]; sultamicillin/pv [Special Situation for Pharmacovigilance]; vancomycin/dt [Drug Therapy]; vancomycin/iv [Intravenous Drug Administration]; vancomycin/pv [Special Situation for Pharmacovigilance]; Gordonia bronchialis; *Gordonia bronchialis infection/co [Complication]; *Gordonia bronchialis infection/di [Diagnosis]; *Gordonia bronchialis infection/dt [Drug Therapy].

Candidate Terms

Gordonia bronchialis [other term]; *Gordonia bronchialis infection / *complication / *diagnosis / *drug therapy [other term].

Drug Index Terms

amoxicillin plus clavulanic acid / drug therapy / oral drug administration / special situation for pharmacovigilance; C reactive protein / endogenous compound; cefazolin / drug therapy / intravenous drug administration / special situation for pharmacovigilance; ceftriaxone / drug therapy / special situation for pharmacovigilance; sultamicillin / drug therapy / intravenous drug administration / special situation for pharmacovigilance; vancomycin / drug therapy / intravenous drug administration / special situation for pharmacovigilance.

Other Index Terms

Actinobacteria; aged; Article; *bacterial infection / *complication / *diagnosis / *drug therapy; bacterium identification; case report; clinical article; computer assisted tomography; *coronary artery bypass surgery; coronary artery disease; DNA sequencing; emergency ward; erythema; follow up; Gordonia; human; human tissue; hyperbaric oxygen therapy; hyperlipidemia; hypertension; *immunocompetence; leukocyte count; leukocytosis; male; nuclear magnetic resonance imaging; *osteomyelitis / *complication; polymerase chain reaction; *postoperative infection / *complication / *diagnosis / *drug therapy; surgical infection; thorax pain; very elderly; wound infection.

CAS Registry Numbers

74469-00-4 (amoxicillin plus clavulanic acid); 79198-29-1 (amoxicillin plus clavulanic acid); 9007-41-4 (C reactive protein); 25953-19-9 (cefazolin); 27164-46-1 (cefazolin); 73384-59-5 (ceftriaxone); 74578-69-1 (ceftriaxone); 58694-35-2 (sultamicillin); 76497-13-7 (sultamicillin); 1404-90-6 (vancomycin); 1404-93-9 (vancomycin)

Year of Publication

2022

Cite

3.

A pediatric case of **Gordonia** otitidis bacteremia detected by long-term blood culture.

Kobayashi T., Otake S., Mori T., Hasegawa D., Kosaka Y., Ohkusu K., Kasai M.

Embase

Journal of Infection and Chemotherapy. 28(10) (pp 1427-1429), 2022. Date of Publication: October 2022.

[Article]

AN:
2018887940

For immunocompromised patients receiving chemotherapy or bone mallow transplantation, slow-growing bacteria should also be considered one of the pathogenic microorganisms. However, there is no evidence pertaining to the microbiological tests associated with a patient with febrile neutropenia before peripheral blood stem cell harvest (PBSCH). We report a case of a 4-year-old cancer-bearing female presenting with a catheter-related bloodstream **infection** due to **Gordonia** otitidis. We detected G. otitidis from long-term blood cultures for approximately 6 days and prevented iatrogenic bacteremia by identifying the same organism from the culture of the PBSC sample and postponing the scheduled PBSCH. If febrile neutropenia occurs before PBSCH, we should collect multiple sets of blood cultures and culture them for a longer period.
Copyright © 2022 Japanese Society of Chemotherapy and The Japanese Association for Infectious Diseases

PMID

35724915 [https://www.ncbi.nlm.nih.gov/pubmed/?term=35724915]

Status

In-Process

Author NameID

Otake, Shogo; ORCID: https://orcid.org/0000-0002-0084-9878 Mori, Takeshi; ORCID: https://orcid.org/0000-0003-0029-7265

Institution

(Kobayashi, Otake, Kasai) Division of Infectious Diseases, Department of Pediatrics, Kobe Children's Hospital, Hyogo, Japan (Mori, Hasegawa, Kosaka) Department of Hematology and Oncology, Children's Cancer Center, Kobe Children's Hospital, Hyogo, Japan (Ohkusu) Department of Microbiology, Tokyo Medical University, Hyogo, Japan

Publisher

Elsevier B.V.

Keyword Heading

Autologous peripheral blood stem cell, Catheter-related bloodstream infection, Gordonia otitidis, Long-term culture, Pediatrics

Emtree Heading

adverse device effect; article; *bacteremia; bacterium culture; *blood culture; cancer model; case report; *catheter infection; cell culture; child; clinical article; febrile neutropenia; female; *Gordonia;
human
; human cell; human tissue; nonhuman; *pediatrics; *peripheral blood stem cell; preschool child.

Other Index Terms

adverse device effect [m]; article [m]; *bacteremia [m]; bacterium culture [m]; *blood culture [m]; cancer model [m]; case report [m]; *catheter infection [m]; cell culture [m]; child [m]; clinical article [m]; febrile neutropenia [m]; female [m]; *Gordonia [m]; human [m]; human cell [m]; human tissue [m]; nonhuman [m]; *pediatrics [m]; *peripheral blood stem cell [m]; preschool child [m].

Year of Publication

2022

Cite

4.
Trends and Species Diversity of Non-tuberculous Mycobacteria Isolated From Respiratory Samples in Northern China, 2014-2021.

Sun Q., Yan J., Liao X., Wang C., Jiang G., Dong L., Wang F., Huang H., Wang G., Pan J.

Embase
Frontiers in public health. 10 (pp 923968), 2022. Date of Publication: 2022.

[Article]

AN:
638663527

Background: Pulmonary non-tuberculous mycobacteria (NTM) **infection** has become a public health concern in China and around the world. The objective of this study was to describe the longitudinal changes in the frequency and diversity of NTM in northern China.
Method(s): We retrospectively analyzed data on mycobacterium species in Beijing Chest Hospital from January 2014 to December 2021. The isolates were identified to species level by targeted DNA sequencing.
Result(s): After excluding duplicates, 1,755 NTM strains were analyzed, which were from 27 provinces in China over 8 years. Among all mycobacteria, the proportion of NTM increased each year, from 4.24% in 2014 to 12.68% in 2021. Overall, 39 different NTM species were identified, including 23 slow growing mycobacteria (SGM) and 16 rapid growing mycobacteria (RGM). The most common species were M. intracellulare (51.62%), M. abscessus (22.22%), M. kansasii (8.32%), M. avium (7.75%) and M. fortuitum (2.05%). The number of NTM species identified also increased each year from 9 in 2014 to 26 in 2021. Most species showed stable isolation rates over the years; however, the proportion of M. avium increased from 3.85 to 10.42% during the study period. Besides, 81 non-mycobacteria strains, including **Gordonia** (21 isolates), Nocardia (19 isolates) and Tsukamurella (17 isolates), etc., were also discovered.
Conclusion(s): The proportion of NTM and species diversity increased considerably in northern China from 2014 to 2021. M. intracellulare was the most common NTM isolated among respiratory specimens, followed by M. abscessus and M. kansasii. Rare NTM species and non-mycobacteria pathogens also need attention.
Copyright © 2022 Sun, Yan, Liao, Wang, Wang, Jiang, Dong, Wang, Huang, Wang and Pan.

PMID

35923959 [https://www.ncbi.nlm.nih.gov/pubmed/?term=35923959]

Institution

(Sun, Yan, Liao, Wang, Wang, Jiang, Dong, Wang, Huang, Wang) National Clinical Laboratory on Tuberculosis, Beijing Key Laboratory for Drug-Resistant Tuberculosis Research, Beijing Chest Hospital, Capital Medical University, Beijing Tuberculosis and Thoracic Tumor Institute, Beijing, China (Pan) Beijing Chest Hospital, Capital Medical University, Beijing Tuberculosis and Thoracic Tumor Institute, Beijing, China

Publisher

NLM (Medline)

Keyword Heading

identification, M. intracellulare, mycobacterium, non-tuberculous mycobacteria, species

Emtree Heading

*atypical mycobacteriosis/ep [Epidemiology]; *atypical Mycobacterium; China; genetics;
human
; microbiology; public health; retrospective study.

Other Index Terms

*atypical mycobacteriosis / *epidemiology; *atypical Mycobacterium; China; genetics; human; microbiology; public health; retrospective study.

Year of Publication

2022

Cite

5.
Saving Time in Blood Culture Diagnostics: a Prospective Evaluation of the Qvella FAST-PBC Prep Application on the Fast System.

Grinberg S., Schubert S., Hochauf-Stange K., Dalpke A.H., Encalada M.N.

Embase
Journal of Clinical Microbiology. 60(5) (no pagination), 2022. Date of Publication: May 2022.

[Article]

AN:
2018990241

Time to results for identification (ID) and antimicrobial susceptibility testing (AST) from blood cultures is an important factor impacting outcome in sepsis. In this study we evaluated a novel device, the FAST^{TM} system from Qvella that concentrates microbial biomass from positive blood culture flasks with the FAST-PBC Prep^{TM} cartridge thereby producing a liquid colony^{TM} (LC), which can be used immediately in standard laboratory downstream applications. We tested 250 positive blood culture bottles collected from January 2021 to May 2021. Results were obtained either with LC or from bacterial overnight cultures using Bruker's MALDI Biotyper^{TM} and bioMerieux's Vitek 2. We compared ID and AST results obtained by both methods and evaluated turnaround times. Two-hundred and fourteen blood cultures could be included in the analysis. In 94% of the cases (n = 201) identification was obtained directly from the LC with concordant results compared to the standard workflow. No discordant results were observed. AST results could be analyzed for 175 samples. Using categorical analysis, concordant agreement was 97.4% of 1,676 AST results for Gram positive bacteria. Agreement for Gram negative bacteria was 98.5% of 980 AST results. Times-to-result were 36.9 h versus 12.8 h for ID and 52.9 h versus 26.8 h for AST in routine workflow vs FAST^{TM} system, respectively. The FAST^{TM} system gives reliable results for ID and AST directly from positive blood cultures and allows for significant time savings in blood culture diagnostics.
Copyright © 2022 American Society for Microbiology. All Rights Reserved.

PMID

35387489 [https://www.ncbi.nlm.nih.gov/pubmed/?term=35387489]

Status

Embase

Institution

(Grinberg, Schubert, Hochauf-Stange, Dalpke, Encalada) Institute of Medical Microbiology and Virology, University Hospital Carl Gustav Carus, Medical Faculty, Technische Universitat Dresden, Dresden, Germany

Publisher

American Society for Microbiology

Keyword Heading

blood culture, diagnostics

Emtree Heading

antibiotic sensitivity; article; Bacillus cereus; bacterium culture; bacterium identification; *blood culture; blood sampling; bloodstream infection/di [Diagnosis]; Candida albicans; controlled study; Cryptococcus neoformans; *diagnostic procedure; Enterobacter cloacae; Escherichia coli; evaluation study;
Gordonia
; Gram negative bacterium; Gram positive bacterium;
human
; intermethod comparison; Klebsiella pneumoniae; Kocuria; Leptotrichia; major clinical study; nonhuman; prospective study; Proteus mirabilis; Pseudomonas aeruginosa; Staphylococcus epidermidis; Staphylococcus hominis; Streptococcus sanguinis; *turnaround time; workflow; *blood culture system/dc [Device Comparison]; microbial identification system/dc [Device Comparison]; Gordonia bronchialis; Kocuria rhizophilia; Leptotrichia trevisanii; Staphylococcus saccharolyticus; FAST-PBC; MALDI Biotyper.

Candidate Terms

Gordonia bronchialis [other term]; Kocuria rhizophilia [other term]; Leptotrichia trevisanii [other term]; Staphylococcus saccharolyticus [other term]; FAST-PBC [device term]; MALDI Biotyper [device term].

Device Index Terms

*blood culture system / *device comparison; microbial identification system / device comparison.

Other Index Terms

antibiotic sensitivity; Article; Bacillus cereus; bacterium culture; bacterium identification; *blood culture; blood sampling; bloodstream infection / diagnosis; Candida albicans; controlled study; Cryptococcus neoformans; *diagnostic procedure; Enterobacter cloacae; Escherichia coli; evaluation study; Gordonia; Gram negative bacterium; Gram positive bacterium; human; intermethod comparison; Klebsiella pneumoniae; Kocuria; Leptotrichia; major clinical study; nonhuman; prospective study; Proteus mirabilis; Pseudomonas aeruginosa; Staphylococcus epidermidis; Staphylococcus hominis; Streptococcus sanguinis; *turnaround time; workflow.

Year of Publication

2022

Cite

6.

Tuberculosis-like pneumonias by the aerobic actinomycetes Rhodococcus, Tsukamurella and **Gordonia**.

Savini V., Fazii P., Favaro M., Astolfi D., Polilli E., Pompilio A., Vannucci M., D'Amario C., Di Bonaventura G., Fontana C., D'Antonio D.

Embase
Microbes and Infection. (no pagination)

[Article In Press]

AN:
51817503

The order Actinomycetales includes phylogenetically diverse but morphologically similar aerobic and anaerobic organisms, exhibiting filamentous branching structures which fragment into rods or coccoid forms. Lung pathogens of the order comprise Mycobacterium, Nocardia, Corynebacterium, Actinomyces, Kytococcus, Rothia, Williamsia, as well as **Gordonia**, Tsukamurella and Rhodococcus. Particularly, members of the last three genera are uncommon aerobic agents of lung cavitations and tuberculosis(TB)-like syndromes, that should be carefully considered in the aetiology of parenchymal lesions. Correct identification of such organisms is hard to obtain, but is crucial to provide patients with adequate diagnose and treatment. Then, this review aims to unearth their airway tropism, as well as their clinical impact as agents of lung disease. © 2011 Institut Pasteur.

Status

Article-in-Press

Institution

(Savini, Fazii, Astolfi, Polilli, D'Antonio) Clinical Microbiology and Virology, Spirito Santo Hospital, via Fonte Romana 8, CAP 65124, Pescara (Pe), Italy (Favaro, Fontana) Department of Experimental Medicine and Biochemical Sciences, Tor Vergata University of Rome (Rm), Italy
(Pompilio, Di Bonaventura) Clinical Microbiology, G. d'Annunzio University of Chieti-Pescara, Department of Biomedical Sciences (Ch), Italy
(Vannucci) Infectious Diseases, G. d'Annunzio University of Chieti-Pescara (Ch), Italy
(D'Amario) Clinical Pathology, San Liberatore Hospital, Atri (Te), Italy
(Fontana) Clinical Microbiology Laboratories, Polyclinic of Tor Vergata, Rome (Rm), Italy

Keyword Heading

Actinomycetes, Gordonia, Pneumonia, Rhodococcus, TB, Tsukamurella

Emtree Heading

*tuberculosis; *Rhodococcus; *Actinobacteria; *Gordonia; *pneumonia;
human
; airway; tropism; Actinomycetales; lung; pathogenesis; Mycobacterium; Nocardia; Corynebacterium; Actinomyces; Rothia; genus; lung cavitation; etiology; patient; lung disease.

Other Index Terms

*tuberculosis; *Rhodococcus; *Actinobacteria; *Gordonia; *pneumonia; human; airway; tropism; Actinomycetales; lung; pathogenesis; Mycobacterium; Nocardia; Corynebacterium; Actinomyces; Rothia; genus; lung cavitation; etiology; patient; lung disease.

Cite

7.

Clinical impact of the use of 16S rRNA sequencing method for the identification of "difficult-to-identify" bacteria in immunocompromised hosts.

Bharadwaj R., Swaminathan S., Salimnia H., Fairfax M., Frey A., Chandrasekar P.H.

Embase
Transplant Infectious Disease. (no pagination)

[Article In Press]

AN:
51694607

Molecular method of 16S rRNA sequencing is reported to be helpful in the accurate identification of organisms with ambiguous phenotypic profiles. We analyzed the use of 16S rRNA sequencing method to identify clinically significant, "difficult-to-identify" bacteria recovered from clinical specimens, and evaluated its role in patient management and consequent clinical outcome. Among the 172 "difficult-to-identify" bacteria recovered over a 4-year period, 140 were gram-positive cocci or gram-negative bacilli; identification by 16S rRNA did not play a role in the management of patients infected with these bacteria. From 32 patients, 33 "difficult-to-identify" gram-positive bacilli were identified; the organisms were mycobacteria, Nocardia, Tsukamurella, Rhodococcus, and **Gordonia**. In 24 patients for whom clinical data were available, results from the 16S rRNA sequencing method led to treatment change in 14 immunocompromised patients (including 7 hematopoietic stem cell recipients and 1 liver transplant recipient). Therapy was modified in 9 patients, initiated in 3 patients, and discontinued in 2 patients. Most patients' therapy was switched to oral antibiotics with discontinuation of intravascular catheters, facilitating early hospital discharge. All 14 patients were alive 30 days after **infection** onset. The present study demonstrates the clinical application of 16S rRNA sequencing method to identify "difficult-to-identify" mycobacteria and other gram-positive bacilli in clinical specimens, particularly in immunocompromised hosts. © 2011 John Wiley & Sons A/S.

Status

Article-in-Press

Institution

(Bharadwaj, Swaminathan, Chandrasekar) Division of Infectious Diseases (Salimnia, Fairfax, Frey) Department of Pathology Wayne State University Detroit Michigan USA
(Salimnia, Fairfax) Detroit Medical Center University Laboratories Detroit Michigan USA

Keyword Heading

16S rRNA, Immunocompromised hosts

Emtree Heading

*bacterium; *immunocompromised patient;
human
; patient; therapy; Mycobacterium; Bacilli; hospital patient; patient care; Gram positive cocci; Gram negative bacterium; Nocardia; Rhodococcus;
Gordonia
; clinical study; hematopoietic stem cell; recipient; liver graft; graft recipient; intravascular catheter; hospital discharge;
infection
; son; *RNA 16S; antibiotic agent.

Drug Index Terms

*RNA 16S; antibiotic agent.

Other Index Terms

*bacterium; *immunocompromised patient; human; patient; therapy; Mycobacterium; Bacilli; hospital patient; patient care; Gram positive cocci; Gram negative bacterium; Nocardia; Rhodococcus; Gordonia; clinical study; hematopoietic stem cell; recipient; liver graft; graft recipient; intravascular catheter; hospital discharge; infection; son.

Cite

8.

Gordonia crocea sp. nov. Isolated from Wound **Infection** After Pacemaker Implantation: Case Report and Literature Review.

Yang Z., Zhang Z., Chen M., Liu Z.

Embase
Infection and Drug Resistance. 15 (pp 2915-2920), 2022. Date of Publication: 2022.

[Article]

AN:
2017107449

Gordonia is a recognized pathogen in patients with immunodeficiency and a normal immune response, which can cause bacteremia, endocarditis, peritonitis and pulmonary **infection**. We report a case of wound **infection** after pacemaker implantation caused by **Gordonia** crocea. Matrix-assisted laser desorption time-of-flight mass spectrometry (MALDI-TOF MS) was routinely used to identify the pathogen, and the results showed that the pathogen could not be accurately identified in the MALDI-TOF database at present. The 16S rRNA gene of the pathogen was further sequenced, and the result was **Gordonia** crocea. To the best of our knowledge, this is the first reported case of **human infection** caused by **Gordonia** crocea.
Copyright © 2022 Yang et al.

Status

Embase

Institution

(Yang, Chen, Liu) Department of Clinical Laboratory, Southwest Hospital, Army Medical University, Chongqing, China (Zhang) Department of Clinical Laboratory, Chongqing General Hospital, University of Chinese Academy of Sciences, Chongqing, China

Publisher

Dove Medical Press Ltd

Keyword Heading

case report, Gordonia crocea, pacemaker implantation, wound infection

Emtree Heading

aged; antibiotic sensitivity; article; bacterium culture; bacterium identification; bacterium isolate; *bacterium isolation; case report; clinical article; coronary angiography; coronary artery atherosclerosis; coronary artery obstruction; dizziness; electrocardiogram; female; *Gordonia; headache; heart palpitation;
human
; hypertension; inguinal hernia/su [Surgery]; local anesthesia; medical history; Meniere disease; nonhuman; outpatient department; *pacemaker implantation; pain; physical examination; sick sinus syndrome; skin redness; skin temperature; thorax radiography; vertigo; very elderly; weakness; *wound infection/co [Complication]; *wound infection/dt [Drug Therapy]; adenine/ec [Endogenous Compound]; amikacin; amoxicillin plus clavulanic acid; ceftriaxone; ciprofloxacin; clarithromycin; cotrimoxazole; cytosine/ec [Endogenous Compound]; guanine/ec [Endogenous Compound]; imipenem; linezolid; minocycline; moxifloxacin/dt [Drug Therapy]; moxifloxacin/po [Oral Drug Administration]; moxifloxacin/pv [Special Situation for Pharmacovigilance]; thymine/ec [Endogenous Compound]; tobramycin; vancomycin/dt [Drug Therapy]; vancomycin/pv [Special Situation for Pharmacovigilance]; vancomycin/tm [Unexpected Outcome of Drug Treatment]; *artificial heart pacemaker/am [Adverse Device Effect]; pacemaker electrode; wound dressing; *Gordonia crocea.

Candidate Terms

*Gordonia crocea [other term].

Device Index Terms

*artificial heart pacemaker / *adverse device effect; pacemaker electrode; wound dressing.

Drug Index Terms

adenine / endogenous compound; amikacin; amoxicillin plus clavulanic acid; ceftriaxone; ciprofloxacin; clarithromycin; cotrimoxazole; cytosine / endogenous compound; guanine / endogenous compound; imipenem; linezolid; minocycline; moxifloxacin / drug therapy / oral drug administration / special situation for pharmacovigilance; thymine / endogenous compound; tobramycin; vancomycin / drug therapy / special situation for pharmacovigilance / unexpected outcome of drug treatment.

Other Index Terms

aged; antibiotic sensitivity; Article; bacterium culture; bacterium identification; bacterium isolate; *bacterium isolation; case report; clinical article; coronary angiography; coronary artery atherosclerosis; coronary artery obstruction; dizziness; electrocardiogram; female; *Gordonia; headache; heart palpitation; human; hypertension; inguinal hernia / surgery; local anesthesia; medical history; Meniere disease; nonhuman; outpatient department; *pacemaker implantation; pain; physical examination; sick sinus syndrome; skin redness; skin temperature; thorax radiography; vertigo; very elderly; weakness; *wound infection / *complication / *drug therapy.

CAS Registry Numbers

22177-51-1 (adenine); 2922-28-3 (adenine); 73-24-5 (adenine); 37517-28-5 (amikacin); 39831-55-5 (amikacin); 110660-83-8 (amikacin); 1257517-67-1 (amikacin); 74469-00-4 (amoxicillin plus clavulanic acid); 79198-29-1 (amoxicillin plus clavulanic acid); 73384-59-5 (ceftriaxone); 74578-69-1 (ceftriaxone); 85721-33-1 (ciprofloxacin); 86393-32-0 (ciprofloxacin); 128074-72-6 (ciprofloxacin); 128074-76-0 (ciprofloxacin); 192934-52-4 (ciprofloxacin); 93107-08-5 (ciprofloxacin); 86483-48-9 (ciprofloxacin); 96186-80-0 (ciprofloxacin); 81103-11-9 (clarithromycin); 8064-90-2 (cotrimoxazole); 71-30-7 (cytosine); 69257-39-2 (guanine); 73-40-5 (guanine); 64221-86-9 (imipenem); 165800-03-3 (linezolid); 10118-90-8 (minocycline); 11006-27-2 (minocycline); 13614-98-7 (minocycline); 151096-09-2 (moxifloxacin); 186826-86-8 (moxifloxacin); 65-71-4 (thymine); 32986-56-4 (tobramycin); 1404-90-6 (vancomycin); 1404-93-9 (vancomycin)

Year of Publication

2022

Cite

9.
Dialysis-related peritonitis caused by **Gordonia** sputi in patient with chronic renal failure: Case report and review of the literature.

Zhang T.-T., Xue W.-C., Wang H.

Embase
Reviews and Research in Medical Microbiology. 26(3) (pp 111-113), 2015. Date of Publication: 01 Jul 2015.

[Review]

AN:
601335061

Gordonia species had been reported to be a rare cause of **human infection**. This may be because they are easily misidentified as Rhodococcus, Nocardia, Arthrobacter or other Actinomycetes by routine biochemical tests. Here, we report a case of dialysis-related peritonitis caused by **Gordonia** sputi in a patient with chronic renal failure. The organism was not identified originally using an automatic identification instrument (VITEK Compact). The strain was finally identified as G. sputi by matrix-assisted laser desorption/ionization time-of-flight mass spectrometry and sequencing of the 16S rRNA gene.
Copyright © 2015 Wolters Kluwer Health, Inc. All rights reserved.

Status

Embase

Institution

(Zhang) Institute of Graduate Studies, Liaoning Medical University, Jinzhou, China (Xue) Clinical Laboratory, General Hospital of Shenyang Military Area Command, Shenyang, China
(Wang) Department of Emergency Medicine, General Hospital of Shenyang Military Area Command, Shenyang 110840, China

Publisher

Lippincott Williams and Wilkins

Keyword Heading

Gordonia sputi, Misidentification, Peritonitis

Emtree Heading

abdominal pain; *Actinomycetales infection/co [Complication]; *Actinomycetales infection/di [Diagnosis]; *Actinomycetales infection/dt [Drug Therapy]; adult; antibiotic therapy; Bacillus licheniformis; case report; *chronic kidney failure/th [Therapy]; continuous ambulatory peritoneal dialysis; decreased appetite; female; gene sequence;
Gordonia

;
human
; matrix assisted laser desorption ionization time of flight mass spectrometry; middle aged; peritoneal cavity; *peritoneal dialysis; *peritonitis/co [Complication]; *peritonitis/di [Diagnosis]; *peritonitis/dt [Drug Therapy]; *peritonitis/et [Etiology]; priority journal; review; catalase/ec [Endogenous Compound]; cefazolin/dt [Drug Therapy]; cefazolin/ip [Intraperitoneal Drug Administration]; genomic DNA/ec [Endogenous Compound]; meropenem/dt [Drug Therapy]; meropenem/iv [Intravenous Drug Administration]; moxifloxacin/dt [Drug Therapy]; moxifloxacin/po [Oral Drug Administration]; peritoneal dialysis fluid; piperacillin/dt [Drug Therapy]; piperacillin/iv [Intravenous Drug Administration]; probiotic agent/dt [Drug Therapy]; probiotic agent/po [Oral Drug Administration]; RNA 16S/ec [Endogenous Compound]; tazobactam/dt [Drug Therapy]; tazobactam/iv [Intravenous Drug Administration]; vancomycin/dt [Drug Therapy]; vancomycin/ip [Intraperitoneal Drug Administration]; *dialysis related peritonitis/co [Complication]; *dialysis related peritonitis/di [Diagnosis]; *dialysis related peritonitis/dt [Drug Therapy]; *dialysis related peritonitis/et [Etiology]; Gordonia sputi; *Gordonia sputi infection/co [Complication]; *Gordonia sputi infection/di [Diagnosis]; *Gordonia sputi infection/dt [Drug Therapy].

Candidate Terms

*dialysis related peritonitis / *complication / *diagnosis / *drug therapy / *etiology [other term]; Gordonia sputi [other term]; *Gordonia sputi infection / *complication / *diagnosis / *drug therapy [other term].

Drug Index Terms

catalase / endogenous compound; cefazolin / drug therapy / intraperitoneal drug administration; genomic DNA / endogenous compound; meropenem / drug therapy / intravenous drug administration; moxifloxacin / drug therapy / oral drug administration; peritoneal dialysis fluid; piperacillin / drug therapy / intravenous drug administration; probiotic agent / drug therapy / oral drug administration; RNA 16S / endogenous compound; tazobactam / drug therapy / intravenous drug administration; vancomycin / drug therapy / intraperitoneal drug administration.

Other Index Terms

abdominal pain; *Actinomycetales infection / *complication / *diagnosis / *drug therapy; adult; antibiotic therapy; Bacillus licheniformis; case report; *chronic kidney failure / *therapy; continuous ambulatory peritoneal dialysis; decreased appetite; female; gene sequence; Gordonia; human; matrix assisted laser desorption ionization time of flight mass spectrometry; middle aged; peritoneal cavity; *peritoneal dialysis; *peritonitis / *complication / *diagnosis / *drug therapy / *etiology; priority journal; Review.

CAS Registry Numbers

9001-05-2 (catalase); 25953-19-9 (cefazolin); 27164-46-1 (cefazolin); 96036-03-2 (meropenem); 151096-09-2 (moxifloxacin); 186826-86-8 (moxifloxacin); 59703-84-3 (piperacillin); 61477-96-1 (piperacillin); 93528-38-2 (tazobactam); 1404-90-6 (vancomycin); 1404-93-9 (vancomycin)

Year of Publication

2015

Cite

10.
Pacemaker-induced endocarditis by **Gordonia** bronchialis.
Endocarditis del marcapasos debido a infeccion por Gordonia bronchialis
<Endocarditis del marcapasos debido a infeccion por
Gordonia
bronchialis.>

Mormeneo Bayo S., Palacian Ruiz M.P., Asin Samper U., Millan Lou M.I., Pascual Catalan A., Villuendas Uson M.C.

Embase
Enfermedades Infecciosas y Microbiologia Clinica. 40(5) (pp 255-257), 2022. Date of Publication: May 2022.

[Article]

AN:
2010642908

Purpose: **Gordonia** species are known to be opportunistic **human** pathogens causing secondary infections. We present the second case in the world of endocarditis caused by **Gordonia** bronchialis and a review of all the cases of endocarditis caused by **Gordonia** spp.
Method(s): The identification was performed by matrix-assisted desorption/ionization time-of-flight mass spectrometry (MALDI-TOF MS) and 16S rRNA gene sequencing were performed to confirm the identification. Antimicrobial susceptibility was performed by MIC test Strip on Mueller-Hinton agar supplemented with 5% defibrinated sheep blood according to Clinical and Laboratory Standards Institute.

Result(s): Pacemaker-induced endocarditis due to **Gordonia** bronchialis **infection** was determined in an 88-year old woman. The patient was treated with ceftriaxone and ciprofloxacin until completing 6 weeks from the pacemaker explant with a good evolution.
Conclusion(s): The case presented supports the pathogenic role of **Gordonia** bronchialis as an opportunistic pathogen and highlights the high risk of suffering infections caused by environmental bacteria.
Copyright © 2020 Sociedad Espanola de Enfermedades Infecciosas y Microbiologia Clinica

PMID

33446400 [https://www.ncbi.nlm.nih.gov/pubmed/?term=33446400]

Status

Embase

Institution

(Mormeneo Bayo, Palacian Ruiz, Millan Lou, Villuendas Uson) Servicio de Microbiologia, Hospital Universitario Miguel Servet Zaragoza, Spain (Asin Samper, Pascual Catalan) Servicio de Medicina Interna y Enfermedades Infecciosas, Hospital Universitario Miguel Servet Zaragoza, Spain

Publisher

Sociedad Espanola de Enfermedades Infecciosas y Microbiologia Clinica

Keyword Heading

Actinomycetales, Bacteremia, Endocarditis, Gordonia, Pacemaker

Emtree Heading

aged; antibiotic sensitivity; antibiotic therapy; article; atrial fibrillation; atrioventricular block; *bacterial endocarditis/co [Complication]; *bacterial endocarditis/di [Diagnosis]; *bacterial endocarditis/dt [Drug Therapy]; bacterial strain; bacterium culture; bacterium identification; bacterium isolation; blood culture; case report; chronic atrial fibrillation; clinical article; combination drug therapy; device removal; erythema/co [Complication]; exudate/co [Complication]; female; fever/co [Complication]; *Gordonia; heart surgery; hospital discharge;
human
; incubation time; matrix assisted laser desorption ionization time of flight mass spectrometry; pacemaker implantation; pruritus/co [Complication]; repeat procedure; RNA sequencing; transthoracic echocardiography; treatment duration; very elderly; wound complication/co [Complication]; wound fluid; amikacin; amoxicillin plus clavulanic acid; ceftriaxone/cb [Drug Combination]; ceftriaxone/dt [Drug Therapy]; ceftriaxone/iv [Intravenous Drug Administration]; ceftriaxone/pv [Special Situation for Pharmacovigilance]; ciprofloxacin/cb [Drug Combination]; ciprofloxacin/dt [Drug Therapy]; ciprofloxacin/iv [Intravenous Drug Administration]; ciprofloxacin/pv [Special Situation for Pharmacovigilance]; cotrimoxazole; imipenem; linezolid; tobramycin; bacterial disease test kit; *cardiac rhythm management device/am [Adverse Device Effect]; Mueller-Hinton agar; *pacemaker electrode/am [Adverse Device Effect]; *Gordonia bronchialis.

Candidate Terms

*Gordonia bronchialis [other term].

Device Index Terms

bacterial disease test kit; *cardiac rhythm management device / *adverse device effect; Mueller-Hinton agar; *pacemaker electrode / *adverse device effect.

Drug Index Terms

amikacin; amoxicillin plus clavulanic acid; ceftriaxone / drug combination / drug therapy / intravenous drug administration / special situation for pharmacovigilance; ciprofloxacin / drug combination / drug therapy / intravenous drug administration / special situation for pharmacovigilance; cotrimoxazole; imipenem; linezolid; tobramycin.

Other Index Terms

aged; antibiotic sensitivity; antibiotic therapy; Article; atrial fibrillation; atrioventricular block; *bacterial endocarditis / *complication / *diagnosis / *drug therapy; bacterial strain; bacterium culture; bacterium identification; bacterium isolation; blood culture; case report; chronic atrial fibrillation; clinical article; combination drug therapy; device removal; erythema / complication; exudate / complication; female; fever / complication; *Gordonia; heart surgery; hospital discharge; human; incubation time; matrix assisted laser desorption ionization time of flight mass spectrometry; pacemaker implantation; pruritus / complication; repeat procedure; RNA sequencing; transthoracic echocardiography; treatment duration; very elderly; wound complication / complication; wound fluid.

CAS Registry Numbers

37517-28-5 (amikacin); 39831-55-5 (amikacin); 110660-83-8 (amikacin); 1257517-67-1 (amikacin); 74469-00-4 (amoxicillin plus clavulanic acid); 79198-29-1 (amoxicillin plus clavulanic acid); 73384-59-5 (ceftriaxone); 74578-69-1 (ceftriaxone); 85721-33-1 (ciprofloxacin); 86393-32-0 (ciprofloxacin); 128074-72-6 (ciprofloxacin); 128074-76-0 (ciprofloxacin); 192934-52-4 (ciprofloxacin); 93107-08-5 (ciprofloxacin); 86483-48-9 (ciprofloxacin); 96186-80-0 (ciprofloxacin); 8064-90-2 (cotrimoxazole); 64221-86-9 (imipenem); 165800-03-3 (linezolid); 32986-56-4 (tobramycin)

Year of Publication

2022

Cite

11.

From NTM (Nontuberculous mycobacterium) to **Gordonia** bronchialis-A Diagnostic Challenge in the COPD Patient.

Franczuk M., Klatt M., Filipczak D., Zabost A., Parniewski P., Kuthan R., Jakubowska L., Augustynowicz-Kopec E.

Embase
Diagnostics. 12(2) (no pagination), 2022. Article Number: 307. Date of Publication: February 2022.

[Article]

AN:
2015580860

In patients with chronic obstructive pulmonary disease, respiratory infections are of various aetiology, predominantly viral and bacterial. However, due to structural and immunological changes within the respiratory system, such patients are also prone to mycobacterial and other relatively rare infections. We present the 70-year old male patient with chronic obstructive pulmonary disease (COPD) and coexisting bronchial asthma, diagnosed due to cough with purulent sputum expectoration lasting over three months. The first microbiological investigation of the sputum sample revealed the growth of mycobacteria. The identification test based on protein MPT64 production indicated an organism belonging to NTM (nontuberculous mycobacterium). However, further species identification by genetic testing verified the obtained culture as not belonging to the Mycobacterium genus. Based on observed morphology, the new characterisation identified an aerobic actinomycete, possibly a Nocardia spp. The isolated strain was recultured on standard microbiological media. The growth of colonies was observed on Columbia blood agar plates and solid Loewenstein-Jensen medium. The Gram and Zhiel-Nielsen stains revealed the presence of Gram-positive acid-fast bacilli. The extraction protocol and identification were performed in two repetitions; the result was G. bronchialis, with a confidence value of 99% and 95%, respectively. The gene sequencing method was applied to confirm the species affiliation of this isolate. The resulting sequence was checked against the 16S ribosomal RNA sequences database (Bacteria and Archaea). The ten best results indicated the genus **Gordonia** (99.04-100%) and 100% similarity of the 16S sequenced region was demonstrated for **Gordonia** bronchialis. The case described indicates that the correct interpretation of microbiological test results requires the use of advanced microbiology diagnosis techniques, including molecular identification of gene sequences. From a clinical point of view, **Gordonia** bronchialis **infection** or colonization may present a mild course, with no febrile episodes and no significant patient status deterioration and thus, it may remain undiagnosed more often than expected.
Copyright © 2022 by the authors. Licensee MDPI, Basel, Switzerland.

Status

Embase

Institution

(Franczuk) Respiratory Physiopathology Department, National Tuberculosis and Lung Diseases Research Institute, Warsaw 01-138, Poland (Klatt, Filipczak, Zabost, Augustynowicz-Kopec) Microbiology Department, National Tuberculosis and Lung Diseases Research Institute, Warsaw 01-138, Poland
(Parniewski) Institute of Medical Biology, Polish Academy of Sciences, Lodz 90-001, Poland
(Kuthan) Department of Medical Microbiology, Medical University of Warsaw, Warsaw 02-091, Poland
(Jakubowska) Radiology Department, National Tuberculosis and Lung Diseases Research Institute, Warsaw 01-138, Poland

Publisher

MDPI

Keyword Heading

Gordonia bronchialis, Microbiological diagnostics, Respiratory infection

Emtree Heading

Actinobacteria; adult; article; Aspergillus fumigatus; asthma; atelectasis; *atypical Mycobacterium; bacterial colonization; bacterial growth; *bacterium; bacterium culture; bacterium isolate; bacterium isolation; bronchiectasis; bronchodilatation; case report; *chronic obstructive lung disease; computer assisted tomography; coughing; DNA extraction; dyspnea; fever; gene sequence; genetic screening;

human

; human tissue; leukocytosis; male; matrix assisted laser desorption ionization time of flight mass spectrometry; Mycobacterium; Mycobacterium tuberculosis; Nocardia; nucleotide sequence; phylogeny; polymerase chain reaction; *respiratory tract infection; species identification; spirometry; alanine aminotransferase; amoxicillin; amphotericin B; cotrimoxazole; fluticasone; immunoglobulin E; lactate dehydrogenase; posaconazole; RNA 16S; salbutamol; salmeterol; tiotropium bromide; voriconazole; *Gordonia bronchialis.

Candidate Terms

*Gordonia bronchialis [other term].

Drug Index Terms

alanine aminotransferase; amoxicillin; amphotericin B; cotrimoxazole; fluticasone; immunoglobulin E; lactate dehydrogenase; posaconazole; RNA 16S; salbutamol; salmeterol; tiotropium bromide; voriconazole.

Other Index Terms

Actinobacteria; adult; Article; Aspergillus fumigatus; asthma; atelectasis; *atypical Mycobacterium; bacterial colonization; bacterial growth; *bacterium; bacterium culture; bacterium isolate; bacterium isolation; bronchiectasis; bronchodilatation; case report; *chronic obstructive lung disease; computer assisted tomography; coughing; DNA extraction; dyspnea; fever; gene sequence; genetic screening; human; human tissue; leukocytosis; male; matrix assisted laser desorption ionization time of flight mass spectrometry; Mycobacterium; Mycobacterium tuberculosis; Nocardia; nucleotide sequence; phylogeny; polymerase chain reaction; *respiratory tract infection; species identification; spirometry.

CAS Registry Numbers

9000-86-6 (alanine aminotransferase); 9014-30-6 (alanine aminotransferase); 26787-78-0 (amoxicillin); 34642-77-8 (amoxicillin); 61336-70-7 (amoxicillin); 1397-89-3 (amphotericin B); 30652-87-0 (amphotericin B); 8064-90-2 (cotrimoxazole); 90566-53-3 (fluticasone); 37341-29-0 (immunoglobulin E); 9001-60-9 (lactate dehydrogenase); 171228-49-2 (posaconazole); 18559-94-9 (salbutamol); 35763-26-9 (salbutamol); 89365-50-4 (salmeterol); 136310-93-5 (tiotropium bromide); 137234-62-9 (voriconazole); 188416-29-7 (voriconazole)

Year of Publication

2022

Cite

12.

A case of prosthetic joint septic arthritis caused by **Gordonia** jacobaea.

Guiraud J., Lescure M., Faganello D., Bebear C., Pereyre S., Menard A.

Embase
Journal of Microbiology, Immunology and Infection. 55(2) (pp 355-357), 2022. Date of Publication: April 2022.

[Letter]

AN:
2014434676

PMID

34489197 [https://www.ncbi.nlm.nih.gov/pubmed/?term=34489197]

Status

Embase

Author NameID

Guiraud, Jennifer; ORCID: https://orcid.org/0000-0002-4288-3265

Institution

(Guiraud, Bebear, Pereyre) Univ. Bordeaux, USC-EA 3671 Mycoplasmal and Chlamydial Infections in Humans, Bordeaux, France
(Guiraud, Bebear, Pereyre) INRA, USC-EA 3671 Mycoplasma and Chlamydia Infections in Humans, Bordeaux, France
(Guiraud, Bebear, Pereyre, Menard) CHU Bordeaux, Bacteriology Department, Bordeaux, France
(Lescure, Faganello) Service des Maladies Infectieuses et Tropicales, Centre Hospitalier Universitaire de Bordeaux, Groupe Hospitalier Pellegrin, Bordeaux, France
(Menard) Univ. Bordeaux, INSERM, UMR1053 Bordeaux Research in Translational Oncology, BaRITOn, Bordeaux, France

Publisher

Elsevier Ltd

Emtree Heading

Actinomyces; aged; antibiotic sensitivity; antibiotic therapy; *bacterial arthritis/co [Complication]; *bacterial arthritis/dt [Drug Therapy]; *bacterial arthritis/et [Etiology]; blood culture; bloodstream infection; case report; catheter removal; clinical article; clinical feature; Corynebacterium; Crohn disease; fever; *Gordonia; Gram positive infection/dt [Drug Therapy]; hospital admission;
human
; joint effusion; knee pain; knee radiography; knee replacement; lavage; letter; leukocyte count; leukocytosis; matrix assisted laser desorption ionization time of flight mass spectrometry; medical history; microorganism detection; neighbor joining method; neutrophil count; Nocardia; nonhuman; nucleotide sequence; polymerase chain reaction; rheumatoid arthritis; RNA sequence; synovial fluid; vein puncture; adalimumab/pv [Special Situation for Pharmacovigilance]; antibiotic agent/pv [Special Situation for Pharmacovigilance]; C reactive protein/ec [Endogenous Compound]; ceftriaxone/dt [Drug Therapy]; ceftriaxone/iv [Intravenous Drug Administration]; ceftriaxone/pv [Special Situation for Pharmacovigilance]; cotrimoxazole/dt [Drug Therapy]; cotrimoxazole/pv [Special Situation for Pharmacovigilance]; levofloxacin/dt [Drug Therapy]; levofloxacin/pv [Special Situation for Pharmacovigilance]; linezolid/dt [Drug Therapy]; linezolid/iv [Intravenous Drug Administration]; linezolid/pv [Special Situation for Pharmacovigilance]; methotrexate/pv [Special Situation for Pharmacovigilance]; prednisolone/pv [Special Situation for Pharmacovigilance]; RNA 16S/ec [Endogenous Compound]; central venous catheter; *joint prosthesis; *Gordonia jacobaea; VIRTUO.

Candidate Terms

*Gordonia jacobaea [other term]; VIRTUO [device term].

Device Index Terms

central venous catheter; *joint prosthesis.

Drug Index Terms

adalimumab / special situation for pharmacovigilance; antibiotic agent / special situation for pharmacovigilance; C reactive protein / endogenous compound; ceftriaxone / drug therapy / intravenous drug administration / special situation for pharmacovigilance; cotrimoxazole / drug therapy / special situation for pharmacovigilance; levofloxacin / drug therapy / special situation for pharmacovigilance; linezolid / drug therapy / intravenous drug administration / special situation for pharmacovigilance; methotrexate / special situation for pharmacovigilance; prednisolone / special situation for pharmacovigilance; RNA 16S / endogenous compound.

Other Index Terms

Actinomyces; aged; antibiotic sensitivity; antibiotic therapy; *bacterial arthritis / *complication / *drug therapy / *etiology; blood culture; bloodstream infection; case report; catheter removal; clinical article; clinical feature; Corynebacterium; Crohn disease; fever; *Gordonia; Gram positive infection / drug therapy; hospital admission; human; joint effusion; knee pain; knee radiography; knee replacement; lavage; Letter; leukocyte count; leukocytosis; matrix assisted laser desorption ionization time of flight mass spectrometry; medical history; microorganism detection; neighbor joining method; neutrophil count; Nocardia; nonhuman; nucleotide sequence; polymerase chain reaction; rheumatoid arthritis; RNA sequence; synovial fluid; vein puncture.

CAS Registry Numbers

331731-18-1 (adalimumab); 1446410-95-2 (adalimumab); 9007-41-4 (C reactive protein); 73384-59-5 (ceftriaxone); 74578-69-1 (ceftriaxone); 8064-90-2 (cotrimoxazole); 100986-85-4 (levofloxacin); 138199-71-0 (levofloxacin); 165800-03-3 (linezolid); 15475-56-6 (methotrexate); 59-05-2 (methotrexate); 7413-34-5 (methotrexate); 7532-09-4 (methotrexate); 6745-93-3 (methotrexate); 51865-79-3 (methotrexate); 60388-53-6 (methotrexate); 50-24-8 (prednisolone)

Year of Publication

2022

Cite

13.
Rare species in the structure of acid-resistent members of the order Actinomycetales isolated from clinical material.

Lyamin A.V.
Embase
Klinicheskaia laboratornaia diagnostika. 65(2) (pp 111-115), 2020. Date of Publication: 2020.

[Article]

AN:
631228023

The article presents data on the structure of acid-resistant members of the order Actinomycetales and rare species that have been isolated and identified using various methods. The study included strains of non-tuberculous mycobacteria (NTM) isolated from clinical material during examination for tuberculosis in the period from 2016 to 2019. The total number of samples with signs of NTMs growth that were included in the study was 316 samples. Primary isolation on Levenshtein-Jensen, Finn II, and MGIT media and NTMs identification by DNA-hybridization. All strains that were not identified prior to the species and culture, identified as microorganisms with a high G+C content (High GC GR +) were re-identified using a MALDI-ToF Microflex LT mass spectrometer (Bruker). By the method of DNA-hybridization, 188 strains isolated by NTM were successfully identified to form 58.5% of all selected cultures. Among the selected species, representatives of slowly growing NTMs (M. avium complex, M. gordonae, M. kansasii) predominated, which amounted to 67.0% of all NTM strains identified to the species. Among the cultures for which DNA hybridization failed to carry out acceptable identification, predominantly NTMs were found, among which M. gordonae, M. avium, M. kansasii dominated. A number of NTMs were represented by rare species: M. iranicum and M. pseudoshottsii. Among this group of microorganisms, other acid-resistant aerobic actinomycetes were isolated, including those of potential clinical significance: **Gordonia** spp., Tsukamurella spp., Rhodococcus spp., Nocardia spp. When identifying cultures containing high concentrations of G+C, the maximum number of microbial associations was revealed, including those consisting of two types of NTMs (M. monacense + M. flavescens, M. avium + M. kansasii), as well as associations of M. gordonae with staphylococci. The same group included rare NTM species: M. fredericbergense, M. szulgai, M. malmoense, M. bohemicum, M. septicum, as well as representatives of the genera Nocardia, **Gordonia**, Tsukamurella.

PMID

32159309 [https://www.ncbi.nlm.nih.gov/pubmed/?term=32159309]

Author NameID

Lyamin A.V.; ORCID: https://orcid.org/0000-0002-5905-1895

Institution

(Lyamin) Samara State Medical University, Samara, Russian Federation

Publisher

NLM (Medline)

Keyword Heading

acid-resistant actinomycetes, non-tuberculous mycobacteria, rare species

Emtree Heading

Actinomycetales; atypical Mycobacterium; *classification; DNA base composition;
human
; isolation and purification; *microbiology; nucleic acid hybridization; tuberculosis.

Other Index Terms

Actinomycetales; atypical Mycobacterium; *classification; DNA base composition; human; isolation and purification; *microbiology; nucleic acid hybridization; tuberculosis.

Year of Publication

2020

Cite

14\.
Peritoneal dialysis-related peritonitis caused by **Gordonia** bronchialis: first pediatric report.

Bruno V., Tjon J., Lin S., Groves H., Kazmi K., Zappitelli M., Harvey E.
Embase
Pediatric Nephrology. 37(1) (pp 217-220), 2022. Date of Publication: January 2022.

[Article]

AN:
2013920051

Introduction: **Gordonia** species, aerobic, weakly acid-fast, Gram-positive bacilli, are a rare cause of peritonitis in patients undergoing peritoneal dialysis (PD). We report the first pediatric case of PD-related peritonitis caused by **Gordonia** bronchialis. Case presentation: A 13-year-old girl with chronic kidney disease (CKD) stage 5D, on continuous cycling PD (CCPD) for 8 years, presented with cloudy PD effluent, with no abdominal discomfort or fever. Intra-peritoneal (IP) loading doses of vancomycin and ceftazidime were started at home after obtaining a PD effluent sample, which showed WBC 2,340 x 10 /L (59% neutrophils) and Gram-positive bacilli. On admission, she was clinically well and afebrile, with no history of methicillin-resistant Staphylococcus aureus (MRSA) **infection**, so vancomycin was discontinued, and IP ceftazidime and cefazolin were started, following a loading dose of intravenous cefazolin. **Gordonia** species grew after 5 days of incubation and later identified as **Gordonia** bronchialis. IP vancomycin was restarted as monotherapy, empirically for a total of 3 weeks therapy. A 2-week course of oral ciprofloxacin was added, based on susceptibility testing. PD catheter replacement was advised due to the risk of recurrence but was refused. A relapse occurred 16 days after discontinuing antibiotics, successfully treated with a 2-week course of IP ceftazidime and vancomycin. The PD catheter was removed and hemodialysis initiated. She received a further 2-week course of oral ciprofloxacin and amoxicillin-clavulanate post PD catheter removal.
Conclusion(s): **Gordonia** bronchialis is an emerging pathogen in PD peritonitis and appears to be associated with a high risk of relapse. PD catheter replacement is strongly suggested.
Copyright © 2021, The Author(s), under exclusive licence to International Pediatric Nephrology Association.

PMID

34633526 [https://www.ncbi.nlm.nih.gov/pubmed/?term=34633526]

Status

In-Process

Author NameID

Bruno, Valentina; ORCID: https://orcid.org/0000-0001-7052-1539 Harvey, Elizabeth; ORCID: https://orcid.org/0000-0002-0705-5034

Institution

(Bruno, Zappitelli, Harvey) Division of Nephrology, The Hospital for Sick Children, 555 University Avenue, Toronto, ON M5G 1X8, Canada (Bruno, Groves, Kazmi, Zappitelli, Harvey) Department of Paediatrics, University of Toronto, Toronto, ON, Canada
(Tjon) Department of Pharmacy, The Hospital for Sick Children, Toronto, ON, Canada
(Lin) Department of Nursing, The Hospital for Sick Children, Toronto, ON, Canada
(Groves, Kazmi) Division of Infectious Diseases, The Hospital for Sick Children, Toronto, ON, Canada

Publisher

Springer Science and Business Media Deutschland GmbH

Keyword Heading

Child, Gordonia bronchialis, Peritoneal dialysis, Peritonitis

Emtree Heading

abdominal discomfort; adolescent; article; case report; child; chronic kidney failure; clinical article; drug combination; drug therapy; effluent; female; fever; *Gordonia; Gram positive bacterium; hemodialysis;
human
; human cell; methicillin resistant Staphylococcus aureus infection; monotherapy; neutrophil; nonhuman; peritoneal dialysis; *peritoneal dialysis catheter; *peritonitis; amoxicillin plus clavulanic acid; cefazolin; ceftazidime; ciprofloxacin; vancomycin.

Drug Index Terms

amoxicillin plus clavulanic acid [m]; cefazolin [m]; ceftazidime [m]; ciprofloxacin [m]; vancomycin [m].

Other Index Terms

abdominal discomfort [m]; adolescent [m]; article [m]; case report [m]; child [m]; chronic kidney failure [m]; clinical article [m]; drug

combination [m]; drug therapy [m]; effluent [m]; female [m]; fever [m]; *Gordonia [m]; Gram positive bacterium [m]; hemodialysis [m]; human [m]; human cell [m]; methicillin resistant Staphylococcus aureus infection [m]; monotherapy [m]; neutrophil [m]; nonhuman [m]; peritoneal dialysis [m]; *peritoneal dialysis catheter [m]; *peritonitis [m].

Year of Publication

2022

Cite

15.
Idiopathic granulomatous mastitis complicated with a breast abscess caused by Gornodia sputi, a case report.
Mastitis granulomatosa idiopatica complicada con absceso de mama producido por Gordonia sputi, caso clinico
<Mastitis granulomatosa idiopatica complicada con absceso de mama producido por Gordonia sputi, caso clinico.>

Gonzalez Aranda A.M., Alvarez Sanchez S., Arnanz Velasco F., Martinez Gomez E., Garrido Sanchez N., Linares Rufo M., Santana Costa A., Zapico Goni A.

Embase
Clinica e Investigacion en Ginecologia y Obstetricia. 49(2) (no pagination), 2022. Article Number: 100739. Date of Publication: 01 Apr 2022.

[Article]

AN:
2016525892

Idiopathic granulomatous mastitis is a rare, benign, chronic entity of unknown cause affecting the breast. It is a diagnosis of exclusion. Among other aetiologies, tuberculosis, infections, sarcoidosis, and other autoimmune diseases such as giant cell arteritis must be ruled out. The main challenges posed by this entity are its differential diagnosis with malignant pathology and its treatment. **Gordonia** spp. are a group of emerging pathogens that are mainly associated with infections associated with medical devices, mainly catheters. Breast involvement is anecdotal and has only been reported in relation to breast prostheses. We present the case of a patient diagnosed with idiopathic granulomatous mastitis complicated with a breast abscess due to **Gordonia** sputi.
Copyright © 2021 Elsevier Espana, S.L.U.

Status

Embase

Institution

(Gonzalez Aranda, Alvarez Sanchez, Arnanz Velasco, Martinez Gomez, Garrido Sanchez, Linares Rufo, Santana Costa, Zapico Goni) Servicio de Ginecologia y Obstetricia, Hospital Universitario Principe de Asturias, Facultad de Medicina, Universidad de Alcala, Alcala de Henares (Madrid), Spain

Publisher

Elsevier Doyma

Keyword Heading

Breast abscess, Gordonia spp., Gordonia sputi, Idiopathic granulomatous mastitis

Emtree Heading

article; *bacterial infection/co [Complication]; *bacterial infection/di [Diagnosis]; *bacterial infection/et [Etiology]; *breast abscess/co [Complication]; *breast abscess/di [Diagnosis]; *breast abscess/et [Etiology]; case report; clinical article; female; *Gordonia; *granulomatous mastitis/di [Diagnosis];

human

; *idiopathic disease/di [Diagnosis]; patient; *Gornodia sputi.

Candidate Terms

*Gornodia sputi [other term].

Other Index Terms

Article; *bacterial infection / *complication / *diagnosis / *etiology; *breast abscess / *complication / *diagnosis / *etiology; case report; clinical article; female; *Gordonia; *granulomatous mastitis / *diagnosis; human; *idiopathic disease / *diagnosis; patient.

Year of Publication

2022

Cite

16.

POS-722 PERITONEAL DIALYSIS-ASSOCIATED PERITONITIS CAUSED BY **GORDONIA** AICHIENSIS: A CASE REPORT AND LITERATURE REVIEW.

WANG B., Cui Z., Cheng X., Luo Q., Xuanhao W., Xinye J., Yizhi C.

Embase

Kidney International Reports. Conference: ISN World Congress of Nephrology (WCN). Kuala Lumpur Malaysia. 7(2 Supplement) (pp S310), 2022. Date of Publication: February 2022.

[Conference Abstract]

AN:
2016904218

Introduction: **Gordonia** species are aerobic and slow-growing gram-positive pathogens that rarely cause **human** infections. However, previous published case reports indicate that **Gordonia** species can cause unusual **human** infections in patients who are immunocompromised or have indwelling catheters. Furthermore, they are uncommon bacilli in patients with peritoneal dialysis-associated peritonitis (PDAP). **Gordonia** aichiensis originally belonged to the genus Rhodococcus and was classified in 1994 as Gordona on the basis of wall characteristics. However, the literature of **Gordonia** aichiensis -related **infection** is quite few and still no report is published about PDAP caused by **Gordonia** aichiensis.

Method(s): PD fluid was collected to two BD Bactec^{TM}Standard Aerobic and Anearobic medium bottles and sent for microbiological analysis. After 12 hours of incubation growth was detected in aerobic medium by Bactec FX Blood culture system. Positive aerobic medium was then inoculated into 5% sheep blood agar and plates were incubated at 37degreeC temperature. Then, the antimicrobial susceptibility test was conducted. Moreover, the dialysate was further investigated via the mNGS method with the sequence of 16s ribosomal RNA blasted in the database.

Result(s): We report the case of a 58-year-old Chinese man with PADP caused by **Gordonia** aichiensis using next generation sequencing method. Combined with the results of drug-sensitivity test, the patient recovered from the **infection** without extubation using effective antibiotic treatment. Meanwhile, we summarized the reported PDAP cases caused by **Gordonia** species, with corresponding treatments and prognosis.

Conclusion(s): This report may help improving the understanding of PDAP caused by gram-positive bacillus and provides clinical insights about the treatment of infections caused by **Gordonia** species. No conflict of interest

Copyright © 2022

Status

CONFERENCE ABSTRACT

Institution

(WANG, Cheng, Luo, Xuanhao, Xinye, Yizhi) Hainan Hospital of Chinese PLA General Hospital, Department of Nephrology, Sanya, China (Cui) Hainan Hospital of Chinese PLA General Hospital, Department of Geriatric Medicine, Sanya, China

Publisher

Elsevier Inc.

Emtree Heading

adult; aerobic cell culture; animal experiment; *antibiotic sensitivity; antibiotic therapy; bacterial growth; bacterium culture; blood culture system; Chinese; conference abstract; dialysate; drug sensitivity; extubation; *Gordonia; Gram positive bacterium; high throughput sequencing;
human
; male; nonhuman; *peritoneal dialysis; *peritonitis; prognosis; Rhodococcus; sheep; agar; endogenous compound; RNA 16S; stirofos.

Drug Index Terms

agar; endogenous compound; RNA 16S; stirofos.

Other Index Terms

adult; aerobic cell culture; animal experiment; *antibiotic sensitivity; antibiotic therapy; bacterial growth; bacterium culture; blood culture system; Chinese; conference abstract; dialysate; drug sensitivity; extubation; *Gordonia; Gram positive bacterium; high throughput sequencing; human; male; nonhuman; *peritoneal dialysis; *peritonitis; prognosis; Rhodococcus; sheep.

CAS Registry Numbers

9002-18-0 (agar); 22248-79-9 (stirofos)

Year of Publication

2022

Cite

17.
An Unusual Case of **Gordonia** sputi Cerebral **Infection**.

Santoiemma P.P., Oyon D.E., Tate M.C., Bolon M.K.

Embase
Infectious Diseases in Clinical Practice. 29(5) (pp E310-E311), 2021. Date of Publication: 01 Sep 2021.

[Article]

AN:
2014747784

Gordonia sputi is an aerobic, gram-positive, weakly acid fast rod found in soil and water. We present the first known case of G. sputi cerebral **infection** as a consequence of iatrogenic intrathecal chemotherapy through an Ommaya reservoir. The patient was successfully treated with a 14-day course of antibiotics without any further complications. There is sparse information on both infections due to and treatment of G. sputi. We present our case report as well as a review of the literature on the epidemiology, susceptibility, and treatment recommendations for G. sputi infections.
Copyright © Wolters Kluwer Health, Inc. All rights reserved.

Status

Embase

Institution

(Santoiemma, Bolon) Department of Internal Medicine (Santoiemma, Bolon) Division of Infectious Diseases
(Oyon, Tate) Department of Neurological Surgery
(Tate) Department of Neurology, Northwestern University, Feinberg School of Medicine, Chicago, IL, United States

Publisher

Lippincott Williams and Wilkins

Keyword Heading

cerebral infection, Gordonia sputi, Ommaya reservoir

Emtree Heading

adult; article; blood culture; *brain infection; breast cancer; case report; clinical article; computer assisted tomography; emergency ward; female; functional neuroimaging; *Gordonia; headache; hemodialysis; hospitalization;
human
; iatrogenic disease; leukocyte count; meningitis; middle aged; nausea and vomiting; nuclear magnetic resonance imaging; oxygen saturation; polymerase chain reaction; thorax radiography; ampicillin; antibiotic agent; C reactive protein; ceftriaxone; trastuzumab; vancomycin; Hickman catheter; Ommaya reservoir.

Device Index Terms

Hickman catheter; Ommaya reservoir.

Drug Index Terms

ampicillin; antibiotic agent; C reactive protein; ceftriaxone; trastuzumab; vancomycin.

Other Index Terms

adult; Article; blood culture; *brain infection; breast cancer; case report; clinical article; computer assisted tomography; emergency ward; female; functional neuroimaging; *Gordonia; headache; hemodialysis; hospitalization; human; iatrogenic disease; leukocyte count; meningitis; middle aged; nausea and vomiting; nuclear magnetic resonance imaging; oxygen saturation; polymerase chain reaction; thorax radiography.

CAS Registry Numbers

69-52-3 (ampicillin); 69-53-4 (ampicillin); 7177-48-2 (ampicillin); 74083-13-9 (ampicillin); 94586-58-0 (ampicillin); 9007-41-4 (C reactive protein); 73384-59-5 (ceftriaxone); 74578-69-1 (ceftriaxone); 180288-69-1 (trastuzumab); 1446410-98-5 (trastuzumab); 1404-90-6 (vancomycin); 1404-93-9 (vancomycin)

Year of Publication

2021

Cite

18.

Conjunctival Microbiota in Patients With Type 2 Diabetes Mellitus and Influences of Perioperative Use of Topical Levofloxacin in Ocular Surgery.

Zhu X., Rong X., Zhang Y., Zhang Q., Wen X., He W., Zhang K., Chen F., Wei L., Lu Y.

Embase
Frontiers in Medicine. 8 (no pagination), 2021. Article Number: 605639. Date of Publication: 06 Apr 2021.

[Article]

AN:
634801432

Background: Patients with type 2 diabetes mellitus (T2DM) are prone to ocular surface infections. We therefore characterized the conjunctival microbiome of T2DM patients and the influence of topical levofloxacin to investigate whether a dysbiosis is associated with this phenomenon.
Method(s): Conjunctival microbiome of 79 T2DM patients and 113 non-diabetic controls was profiled using the 16S rDNA sequencing approach. Furthermore, 21 T2DM and 14 non-diabetic patients who underwent cataract surgeries were followed up perioperatively and the influence of pre- and post-operative levofloxacin on the conjunctival microbiome was further investigated prospectively and compared longitudinally.
Result(s): The alpha-diversity of the conjunctival microbiota was significantly higher in T2DM patients than in controls ($P < 0.05$). Significant differences in both composition and function of the conjunctival microbiome were identified on the ocular surface of T2DM patients as compared to non-diabetic controls. Particularly, phylum Bacteroidetes and Fusobacteria, genus Pseudomonas, Haemophilus, and Empedobacter were enriched, while genus Streptococcus was reduced on the T2DM ocular surface. Microbial genes functioning of bacterial chemotaxis was elevated in the conjunctival microbiome of T2DM patients. Furthermore, compared to the initial status, several genera including Staphylococcus were more abundant in the conjunctival microbiome of T2DM patients after 3-days use of preoperative levofloxacin topically, while no genus was more abundant in the non-diabetic follow-up group. No difference was observed between initial status and 7 days after ceasing all postoperative medications in both diabetic and non-diabetic follow-up groups.
Conclusion(s): The conjunctival microbiome of T2DM patients was more complex and may respond differently to topical antibiotics.
© Copyright © 2021 Zhu, Wei, Rong, Zhang, Zhang, Wen, He, Zhang, Chen, Wei and Lu.

Status

Embase

Institution

(Zhu, Wei, Rong, Zhang, He, Zhang, Lu) Department of Ophthalmology and Eye Institute, Ear, Nose and Throat, ENT) Hospital of Fudan University, Shanghai, China (Zhu, Wei, Rong, Zhang, He, Zhang, Lu) National Health Commission (NHC) Key Laboratory of Myopia (Fudan University), Key Laboratory of Myopia, Chinese Academy of Medical Science, Shanghai, China
(Zhu, Wei, Rong, Zhang, He, Zhang, Lu) Shanghai Key Laboratory of Visual Impairment and Restoration, Shanghai, China

(Zhang, Chen) Central Laboratory, Peking University School and Hospital of Stomatology, Beijing, China
(Wen, Wei) State Key Laboratory of Ophthalmology, Zhongshan Ophthalmic Center, Sun Yat-sen University, Guangzhou, China

Publisher

Frontiers Media S.A.

Keyword Heading

cataract surgery, conjunctival microbiota, levofloxacin, Staphylococcus, type 2 diabetes mellitus

Emtree Heading

Abiotrophia; Actinomyces; Aerococcus; Aeromonas; aged; Aggregatibacter; article; *bacterial microbiome; Bacteroidetes; Bradyrhizobium; Capnocytophaga; *cataract/su [Surgery]; *cataract extraction; chemotaxis; *conjunctiva; conjunctival swab; controlled study; diabetic patient; DNA extraction; DNA sequencing; endophthalmitis; Enterococcus; eye infection; female; Finegoldia; follow up; Fusobacteria; Fusobacterium; Gemella;
Gordonia
; Haemophilus;
human
; Klebsiella; major clinical study; male; microbial gene; Neisseria; *non insulin dependent diabetes mellitus; preoperative treatment; Propionibacterium; Pseudomonas; Ralstonia; Staphylococcus; Stenotrophomonas; Streptococcus; treatment duration; treatment response; Veillonella; diclofenac; DNA 16S; eye drops; *levofloxacin/tp [Topical Drug Administration]; prednisolone acetate; RNA purification kit; spectrophotometer; alkanindiges; Empedobacter; Filifactor.

Candidate Terms

Alkanindiges [other term]; Empedobacter [other term]; Filifactor [other term]; Massiliae [other term]; difei [drug term].

Device Index Terms

RNA purification kit; spectrophotometer.

Drug Index Terms

diclofenac; DNA 16S; eye drops; *levofloxacin / *topical drug administration; prednisolone acetate.

Other Index Terms

Abiotrophia; Actinomyces; Aerococcus; Aeromonas; aged; Aggregatibacter; Article; *bacterial microbiome; Bacteroidetes; Bradyrhizobium; Capnocytophaga; *cataract / *surgery; *cataract extraction; chemotaxis; *conjunctiva; conjunctival swab; controlled study; diabetic patient; DNA extraction; DNA sequencing; endophthalmitis; Enterococcus; eye infection; female; Finegoldia; follow up; Fusobacteria; Fusobacterium; Gemella; Gordonia; Haemophilus; human; Klebsiella; major clinical study; male; microbial gene; Neisseria; *non insulin dependent diabetes mellitus; preoperative treatment; Propionibacterium; Pseudomonas; Ralstonia; Staphylococcus; Stenotrophomonas; Streptococcus; treatment duration; treatment response; Veillonella.

Drug Trade Name

cravit ophthalmic: Santen [Japan], difei: sinqi [China], pred forte: Allergan [Ireland]

CAS Registry Numbers

15307-79-6 (diclofenac); 15307-86-5 (diclofenac); 100986-85-4 (levofloxacin); 138199-71-0 (levofloxacin); 52-21-1 (prednisolone acetate); 52628-64-5 (prednisolone acetate)

Year of Publication

2021

Cite

19.
DISSEMINATED NOCARDIOSIS PRESENTING AS ACUTE LIVER FAILURE AND INTRAPARENCHYMAL HEMORRHAGE.

Talon A., Rahman Halawa A., Arif M., IRANDOST M., Vaccarello A., Marco S.

Embase
Chest. Conference: CHEST 2021 Annual Meeting. Virtual, Online. 160(4 Supplement) (pp A802), 2021. Date of Publication: October 2021.

[Conference Abstract]

AN:

2014929244

TOPIC: Critical Care **TYPE:** Medical Student/Resident Case Reports **INTRODUCTION:** Nocardiosis is an opportunistic **infection** that most commonly manifests as pulmonary disease. Disseminated nocardiosis is rare and is associated with high mortality. We present a case of a woman with disseminated nocardiosis presenting with acute liver failure complicated by intraparenchymal hemorrhage. **CASE PRESENTATION:** A 30-year-old woman with a history of alcohol abuse presented with jaundice, bloating, and diarrhea for 2 weeks. Laboratory examination revealed hemoglobin of 5.2 g/dL, platelets 60,000/microL, INR 3.4, AST 113 U/L, ALT 38 U/L, and total bilirubin of 30 mg/dL. MRCP revealed a normal biliary tree, nodular liver contour and evidence of portal hypertension. Acetaminophen level and acute viral hepatitis panel were negative. HIV serology and autoimmune hepatitis panel were negative. Chest CT revealed a 5 x 3.5 cm subpleural mass in the left upper lobe with areas of gas density (Fig 1). Her hospital course was complicated by a rapid deterioration in mental status and DIC. Head CT revealed an acute left occipital-parietal intraparenchymal hemorrhage. She was intubated and transferred to the ICU. Bronchoscopy with BAL grew Nocardia otitidiscaviarum. Brain MRI revealed FLAIR signal hyperintensity lining the ventricles, concerning for ventriculitis or abscess (Fig 2). An external ventricular drain was placed for interval development of hydrocephalus. The patient received IV trimethoprim-sulfamethoxazole and intrathecal amikacin. Given her poor prognosis, her family elected to withdraw care. **DISCUSSION:** Nocardia is a branching, filamentous gram-positive bacilli found ubiquitously in soil. **Infection** usually occurs by inhalation of aerosolized bacteria resulting in predominant lung involvement with potential hematogenous spread to other organs. Patients with impaired T-cell mediated immunity, such as HIV, chronic steroid use, diabetes, or alcoholism are at risk of developing severe **infection**. Our patient had a longstanding history of heavy alcohol use, likely predisposing her to disseminated **infection**. Nocardiosis is considered disseminated when two or more nocardial abscesses are found at two or more locations. Up to 20% of all Nocardia infections involve the brain. Current guidelines recommend imaging of the brain for any patient with confirmed or suspected pulmonary nocardiosis. Prognosis is usually poor, and mortality can be as high as 64%. First-line treatment is trimethoprim-sulfamethoxazole. However some species, such as N. otitidiscaviarum found in our patient, are occasionally resistant. Due to suspected CNS involvement, dual coverage was recommended with the addition of IV amikacin at dosing for CNS penetrance.
CONCLUSION(S): In patients presenting with a cavitary lung lesion and associated brain lesion, nocardiosis should be considered in the differential. Early brain imaging may be warranted in patients presenting with severe disease. REFERENCE #1: Pulmonary nocardiosis: risk factors and outcomes. AU Martinez Tomas R, Menendez Villanueva R, Reyes Calzada S, Santos Durantez M, Valles Tarazona JM, Modesto Alapont M, Gobernado Serrano M SO Respirology. 2007;12(3):394. REFERENCE #2: Conville PS, Witebsky FG. Nocardia, Rhodococcus, **Gordonia**, Actinomadura, Streptomyces, and other Aerobic Actinomycetes. In: Murray PR, Baron EJ, Jorgensen JH, Landry ML, Pfaller MA, editors. Manual of Clinical Microbiology. 9th ed. Washington, **DC:** ASM Press; 2007. p. 515. **DISCLOSURES:** No relevant relationships by Muhammad Arif, source=Web Response No relevant relationships by Abdul Rahman Halawa, source=Web Response No relevant relationships by MAYKEL IRANDOST, source=Web Response no disclosure on file for Sean Marco; No relevant relationships by Andrew Talon, source=Web Response No relevant relationships by Anthony Vaccarello, source=Web Response
Copyright © 2021 American College of Chest Physicians

Status

CONFERENCE ABSTRACT

Publisher

Elsevier Inc.

Emtree Heading

abscess; Actinobacteria; Actinomadura; *acute liver failure; acute viral hepatitis; adult; alanine aminotransferase blood level; alcohol abuse; alcoholism; aspartate aminotransferase level; autoimmune hepatitis; *bleeding; bloating; brain damage; brain ventriculitis; bronchoscopy; case report; cellular immunity; clinical article; complication; conference abstract; deterioration; diabetes mellitus; diarrhea; District of Columbia; drug combination; drug therapy; drug withdrawal; editor; female;
Gordonia
;
human
; human cell; Human immunodeficiency virus; hydrocephalus; inhalation; international normalized ratio; jaundice; laboratory test; lung lesion; mental health; microbiology; mortality; Nocardia otitidiscaviarum; *nocardiosis; nonhuman; nuclear magnetic resonance imaging; penetrance; portal hypertension; practice guideline; prognosis; Rhodococcus; risk factor; serology; Streptomyces; thorax; thrombocyte; ventriculostomy catheter; amikacin; bilirubin; brodifacoum; cotrimoxazole; endogenous compound; hemoglobin; paracetamol; steroid.

Drug Index Terms

amikacin; bilirubin; brodifacoum; cotrimoxazole; endogenous compound; hemoglobin; paracetamol; steroid.

Other Index Terms

abscess; Actinobacteria; Actinomadura; *acute liver failure; acute viral hepatitis; adult; alanine aminotransferase blood level; alcohol abuse; alcoholism; aspartate aminotransferase level; autoimmune hepatitis; *bleeding; bloating; brain damage; brain ventriculitis; bronchoscopy; case report; cellular immunity; clinical article; complication; conference abstract; deterioration; diabetes mellitus; diarrhea; District of Columbia; drug combination; drug therapy; drug withdrawal; editor; female; Gordonia; human; human cell; Human immunodeficiency virus; hydrocephalus; inhalation; international normalized ratio; jaundice; laboratory test; lung lesion; mental health; microbiology; mortality; Nocardia otitidiscaviarum; *nocardiosis; nonhuman; nuclear magnetic resonance imaging; penetrance; portal hypertension; practice guideline; prognosis; Rhodococcus; risk factor; serology; Streptomyces; thorax; thrombocyte; ventriculostomy catheter.

CAS Registry Numbers

37517-28-5 (amikacin); 39831-55-5 (amikacin); 110660-83-8 (amikacin); 1257517-67-1 (amikacin); 18422-02-1 (bilirubin); 635-65-4 (bilirubin); 56073-10-0 (brodifacoum); 8064-90-2 (cotrimoxazole); 9008-02-0 (hemoglobin); 103-90-2 (paracetamol)

Year of Publication

2021

Cite

20.

Peritoneal dialysis-related peritonitis caused by **gordonia** bronchialis: First pediatric report.

Bruno V., Tjon J., Lin S., Groves H., Kazmi K., Zappitelli M., Harvey E.

Embase

Peritoneal Dialysis International. Conference: Annual Dialysis Conference 2021. Virtual. 41(1 Supplement) (pp 12S), 2021. Date of Publication: February 2021.

[Conference Abstract]

AN:
634495860

Background: **Gordonia** species are aerobic, weakly acidfast, Gram-positive bacilli, which can cause peritonitis in patients undergoing peritoneal dialysis (PD). No PDrelated **infection** has been reported in children.
Aim(s): To describe the first reported pediatric patient with **Gordonia** bronchialis PD-related peritonitis. Approach: A 13-yearold girl with end-stage kidney disease secondary to Focal Segmental Glomerulosclerosis (WT1 mutation), on continuous cycling PD for 8 years, presented with cloudy PD effluent, with no abdominal discomfort or fever. Intraperitoneal (IP) loading doses of vancomycin and ceftazidime were started after obtaining a PD effluent sample.
Result(s): PD effluent showed WBC 2340x10^6/L (59% Neutrophils), and Gram-positive bacilli and grew **Gordonia** species after 5 days incubation, identified as **Gordonia** bronchialis by 16 s rRNA gene sequencing. IP vancomycin was continued empirically, based on clinical response and published evidence. A 2-week course of oral ciprofloxacin was added, based on susceptibility test, along with 3-weeks total therapy with IP vancomycin. PD catheter replacement was advised due to significant risk of recurrence, but was refused. A relapse occurred 16 days after discontinuing antibiotics, with **Gordonia** bronchialis growing after 8 days incubation. A 2-week course of IP ceftazidime and vancomycin was prescribed, with good response. The PD catheter was removed and hemodialysis initiated. She received a 2-week course of oral ciprofloxacin and amoxicillin-clavulanate post PD catheter removal.
Conclusion(s): **Gordonia** bronchialis is an emerging pathogen in PD peritonitis and appears to be associated with a high risk of relapse. PD catheter replacement is strongly suggested.

Status

CONFERENCE ABSTRACT

Institution

(Bruno, Tjon, Lin, Groves, Kazmi, Zappitelli, Harvey) The Hospital for Sick Children, Toronto, ON, Canada

Publisher

SAGE Publications Inc.

Emtree Heading

abdominal discomfort; case report; child; clinical article; conference abstract; drug combination; drug therapy; effluent; end stage renal disease; female; fever; focal glomerulosclerosis; gene mutation; gene sequence; *Gordonia; Gram positive bacterium; hemodialysis;
human
; human cell; neutrophil; nonhuman; pediatric patient; *peritoneal dialysis catheter; *peritonitis; amoxicillin plus clavulanic acid; ceftazidime; ciprofloxacin; endogenous compound; RNA 16S; vancomycin; WT1 protein.

Drug Index Terms

amoxicillin plus clavulanic acid; ceftazidime; ciprofloxacin; endogenous compound; RNA 16S; vancomycin; WT1 protein.

Other Index Terms

abdominal discomfort; case report; child; clinical article; conference abstract; drug combination; drug therapy; effluent; end stage renal disease; female; fever; focal glomerulosclerosis; gene mutation; gene sequence; *Gordonia; Gram positive bacterium; hemodialysis; human; human cell; neutrophil; nonhuman; pediatric patient; *peritoneal dialysis catheter; *peritonitis.

CAS Registry Numbers

74469-00-4 (amoxicillin plus clavulanic acid); 79198-29-1 (amoxicillin plus clavulanic acid); 72558-82-8 (ceftazidime); 85721-33-1 (ciprofloxacin); 86393-32-0 (ciprofloxacin); 1404-90-6 (vancomycin); 1404-93-9 (vancomycin)

Year of Publication

2021

Cite

21.

A genomic approach to investigating ocular surface microorganisms: Monitoring core microbiota on eyelid margin with a dot hybridization assay.

Kuo M.-T., Chao T.-L., Kuo S.-F., Chien C.-C., Chen A., Lai Y.-H., Huang Y.-T.

Embase
International Journal of Molecular Sciences. 21(21) (pp 1-16), 2020. Article Number: 8299. Date of Publication: 01 Nov 2020.

[Article]

AN:
2005418047

A sound ocular surface microbiota has been recognized as a part of ocular surface health following a growing body of evidence from next-generation sequencing technique and metagenomic analysis. However, even from the perspective of contemporary precision medicine, it is difficult to directly apply these new technologies to clinical practice. Therefore, we proposed a model based on dot hybridization assay (DHA) to bridge conventional culture with a metagenomic approach in investigating and monitoring ocular surface microbiota. Endophthalmitis, mostly caused by bacterial **infection**, is the most severe complication of many intraocular surgeries, such as cataract surgery. Hazardous microorganisms hiding and proliferating in the ocular surface microbiota not only increase the risk of endophthalmitis but also jeopardize the effectiveness of the preoperative aseptic procedure and postoperative topical antibiotics. The DHA model enables the simultaneous assessment of bacterial bioburden, detection of target pathogens and microorganisms, and surveillance of methicillin/oxacillin resistance gene mecA in the ocular surface microbiota. This assay revealed heavier bacterial bioburden in men, compatible with a higher risk of endophthalmitis in male patients who underwent cataract surgery. No occurrence of endophthalmitis for these patients was compatible with non-hazardous microorganisms identified by specific dots for target pathogens. Moreover, the mecA dot detected oxacillin-resistant strains, of which culture failed to isolate. Therefore, the DHA model could provide an alternative genomic approach to investigate and monitor ocular surface microorganisms in clinical practice nowadays.
Copyright © 2020 by the authors. Licensee MDPI, Basel, Switzerland.

PMID

33167500 [https://www.ncbi.nlm.nih.gov/pubmed/?term=33167500]

Status

Embase

Institution

(Kuo, Chen, Lai, Huang) Department of Ophthalmology, Kaohsiung Chang Gung Memorial Hospital and Chang Gung University College of Medicine, Kaohsiung 83301, Taiwan (Republic of China) (Chao, Kuo, Chien) Department of Laboratory Medicine, Kaohsiung Chang Gung Memorial Hospital and Chang Gung University College of Medicine, Kaohsiung 83301, Taiwan (Republic of China)
(Kuo) Department of Medical Biotechnology and Laboratory Sciences, College of Medicine, Chang Gung University, Taoyuan 333323, Taiwan (Republic of China)

Publisher

MDPI AG

Keyword Heading

Antibiotic resistance, Cataract, Endophthalmitis, Microbiota, Ocular surface

Emtree Heading

Acinetobacter baumannii; adult; aged; antibiotic resistance; article; Bacillus; Bacillus cereus; bacterial infection; bacterial strain; blood culture; Brevundimonas; cataract extraction; Citrobacter; clinical article; coagulase negative Staphylococcus; cohort analysis; controlled study; disinfection; DNA extraction; *dot hybridization; *endophthalmitis/co [Complication]; *endophthalmitis/dt [Drug Therapy]; *endophthalmitis/pc [Prevention]; Enterococcus faecalis; eye surgery; female;
Gordonia
; high throughput sequencing;
human
; infection risk; Klebsiella pneumoniae; male; matrix assisted laser desorption ionization time of flight mass spectrometry; matrix-assisted laser desorption-ionization mass spectrometry; *metagenomics; *microflora; microorganism; Morganella; *ocular surface disease; Paenibacillus; postoperative infection; Propionibacterium acnes; Pseudomonas aeruginosa; Serratia marcescens; Staphylococcus aureus; Staphylococcus epidermidis; Staphylococcus haemolyticus; Staphylococcus sciuri; Staphylococcus warneri; Streptococcus mitis; Streptococcus oralis; Streptococcus salivarius; antibiotic agent/tp [Topical Drug Administration]; sulfamethoxazole/dt [Drug Therapy]; sulfamethoxazole/tp [Topical Drug Administration]; blood culture system; *eyelid flora; Microbacterium aurum; Bactec FX.

Candidate Terms

*eyelid flora [other term]; Microbacterium aurum [other term]; BACTEC FX [device term].

Device Index Terms

blood culture system.

Drug Index Terms

antibiotic agent / topical drug administration; sulfamethoxazole / drug therapy / topical drug administration.

Other Index Terms

Acinetobacter baumannii; adult; aged; antibiotic resistance; Article; Bacillus; Bacillus cereus; bacterial infection; bacterial strain; blood culture; Brevundimonas; cataract extraction; Citrobacter; clinical article; coagulase negative Staphylococcus; cohort analysis; controlled study; disinfection; DNA extraction; *dot hybridization; *endophthalmitis / *complication / *drug therapy / *prevention; Enterococcus faecalis; eye surgery; female; Gordonia; high throughput sequencing; human; infection risk; Klebsiella pneumoniae; male; matrix assisted laser desorption ionization time of flight mass spectrometry; matrix-assisted laser desorption-ionization mass spectrometry; *metagenomics; *microflora; microorganism; Morganella; *ocular surface disease; Paenibacillus; postoperative infection; Propionibacterium acnes; Pseudomonas aeruginosa; Serratia marcescens; Staphylococcus aureus; Staphylococcus epidermidis; Staphylococcus haemolyticus; Staphylococcus sciuri; Staphylococcus warneri; Streptococcus mitis; Streptococcus oralis; Streptococcus salivarius.

CAS Registry Numbers

723-46-6 (sulfamethoxazole)

Year of Publication

2020

Cite

22.
Gordonia sputi related multiple brain abscesses, an AIDS-presenting illness: Thinking outside the box.

Eribi A., Al-Amri K., Al-Jabri A., Osman A., Mohamed Elfadil O.

Embase

IDCases. 21 (no pagination), 2020. Article Number: e00906. Date of Publication: 2020.

[Article]

AN:
2007006468

HIV/AIDS has been recognized as a global health issue with significant burden on healthcare services worldwide. Diagnostic and therapeutic challenges include wide range of difficult to identify and treat infections. **Gordonia** sputi is known to cause multi-system infections in setting of HIV/AIDS. It is often difficult to isolate this organism requiring high suspicion index and special testing techniques. While there is no guidelines-recommended antibacterials regimen for **Gordonia** sputi **infection**, extended combined broad spectrum antibacterials have been successfully used. Our patient in this report is a 50-year-old male with no past history who presented with progressive weakness on the right side of the body and urinary incontinence over the duration of one month. MRI scan of the brain showed bilateral ring-enhancing lesions. **Gordonia** sputi was identified from a tissue biopsy using 16S ribosomal RNA sequencing technique. HIV test for antibodies came to be reactive and a CD4 cell count of 7/muL. The patient was treated with combination of antibacterials and had remarkable radiological interval changes and relatively slower yet apparent clinical improvement. Unfortunately, and despite initial recovery, patient has later developed multi-drug resistant hospital acquired pneumonia leading to his death in ICU during course of hospitalization. Treatment of **Gordonia** sputi in setting of HIV **infection** with a combination of antibacterials over extended period appears to be safe and effective. To our knowledge, this is the first report of **Gordonia** sputi related multiple brain abscesses as AIDS-presenting illness.
Copyright © 2020 The Author(s)

Status

Embase

Author NameID

Mohamed Elfadil, Osman; ORCID: https://orcid.org/0000-0003-0240-6453

Institution

(Eribi, Al-Amri, Al-Jabri) Division of Infectious Diseases, Department of Medicine, Armed Forces Hospital, Muscat, Oman (Eribi, Mohamed Elfadil) Department of Medicine, Armed Forces Hospital, Muscat, Oman
(Al-Jabri) Department of Infection Prevention and Occupational Safety, Khoula Hospital, Muscat, Oman
(Osman, Mohamed Elfadil) Mayo Clinic, Rochester, MN, United States

Publisher

Elsevier Ltd

Keyword Heading

AIDS-defining illness, Gordonia, Gordonia sputi

Emtree Heading

Acinetobacter; *acquired immune deficiency syndrome/dt [Drug Therapy]; adult; antibiotic sensitivity; article; *brain abscess/di [Diagnosis]; brain biopsy; brain damage; case report; CD4 CD8 ratio; CD4 lymphocyte count; clinical article; *Corynebacterium infection/di [Diagnosis]; *Corynebacterium infection/dt [Drug Therapy]; dyspnea; fever; gene sequence; *Gordonia; hospital acquired pneumonia; hospitalization;
human
; Human immunodeficiency virus infection/dt [Drug Therapy]; human tissue; hyperreflexia; hypotension; lumbar puncture; male; middle aged; minimum inhibitory concentration; multidrug resistant Acinetobacter baumannii; muscle atrophy; muscle hypertonia; night sweat; nuclear magnetic resonance imaging; priority journal; subdural hematoma; thorax radiography; urine incontinence; weakness; amikacin; ceftriaxone; ciprofloxacin; efavirenz plus emtricitabine plus tenofovir disoproxil/dt [Drug Therapy]; gentamicin; imipenem; meropenem/dt [Drug Therapy]; rifampicin/dt [Drug Therapy]; RNA 16S; vancomycin/dt [Drug Therapy]; *Gordonia sputi; *Gordonia sputi infection/di [Diagnosis]; *Gordonia sputi infection/dt [Drug Therapy].

Candidate Terms

*Gordonia sputi [other term]; *Gordonia sputi infection / *diagnosis / *drug therapy [other term].

Drug Index Terms

amikacin; ceftriaxone; ciprofloxacin; efavirenz plus emtricitabine plus tenofovir disoproxil / drug therapy; gentamicin; imipenem; meropenem / drug therapy; rifampicin / drug therapy; RNA 16S; vancomycin / drug therapy.

Other Index Terms

Acinetobacter; *acquired immune deficiency syndrome / *drug therapy; adult; antibiotic sensitivity; Article; *brain abscess / *diagnosis; brain biopsy; brain damage; case report; CD4 CD8 ratio; CD4 lymphocyte count; clinical article; *Corynebacterium infection / *diagnosis / *drug therapy; dyspnea; fever; gene sequence; *Gordonia; hospital acquired pneumonia; hospitalization; human; Human immunodeficiency virus infection / drug therapy; human tissue; hyperreflexia; hypotension; lumbar puncture; male; middle aged; minimum inhibitory concentration; multidrug resistant Acinetobacter baumannii; muscle atrophy; muscle hypertonia; night sweat; nuclear magnetic resonance imaging; priority journal; subdural hematoma; thorax radiography; urine incontinence; weakness.

CAS Registry Numbers

37517-28-5 (amikacin); 39831-55-5 (amikacin); 73384-59-5 (ceftriaxone); 74578-69-1 (ceftriaxone); 85721-33-1 (ciprofloxacin); 1392-48-9 (gentamicin); 1403-66-3 (gentamicin); 1405-41-0 (gentamicin); 64221-86-9 (imipenem); 96036-03-2 (meropenem); 13292-46-1 (rifampicin); 1404-90-6 (vancomycin); 1404-93-9 (vancomycin)

Year of Publication

2020

Cite

23.

Actinomycetoma caused by **Gordonia** westfalica: first reported case of **human infection**.

Gueneau R., Blanchet D., Rodriguez-Nava V., Bergeron E., Soulier M., Bestandji N., Demar M., Couppie P., Blaizot R.

Embase
New Microbes and New Infections. 34 (no pagination), 2020. Article Number: 100658. Date of Publication: March 2020.

[Letter]

AN:
2005202127

Bacteria of the genus **Gordonia** are rarely involved in **human** infections. We report here the case of a 30-year-old man from Guinea Buissau with mycetoma of the foot. 16S DNA sequencing after surgical biopsy identified **Gordonia** westfalica. To our knowledge, this is the first report of **human infection** caused by G. westfalica.
Copyright © 2020 The Authors

Status

Embase

Institution

(Gueneau, Couppie, Blaizot) Dermatology Department, University of French Guiana, Cayenne, French Guiana (Blanchet, Demar) Laboratory of Parasitology and Mycology, University of French Guiana, Cayenne, French Guiana
(Bestandji) Orthopaedics Department, Hopital Andree Rosemon, University of French Guiana, Cayenne, French Guiana
(Demar, Couppie, Blaizot) EA 3593 Ecosystemes Amazoniens et Pathologies Tropicales, University of French Guiana, Cayenne, French Guiana
(Rodriguez-Nava, Bergeron) UMR CNRS 5557, Center for Microbial Ecology, Observatoire Francais des Nocardioses, Laboratoire de Mycologie Fondamentale et Appliquee aux Biotechnologies Industrielles, Faculte de Pharmacie, Universite Claude Bernard Lyon I, Lyon, France
(Soulier) Pathologistes associes, Martigues, France

Publisher

Elsevier Ltd

Keyword Heading

Actinomyces, Gordonia, Mycetoma, Neglected tropical disease, Spectrum analysis

Emtree Heading

Actinobacteria; *actinomycetoma/dt [Drug Therapy]; *actinomycetoma/et [Etiology]; adult; antibiotic therapy; bacterium culture; bacterium identification; case report; clinical article; clinical assessment; clinical examination; DNA sequencing; Fonsecaea; foot; *Gordonia; histopathology;

human
; human tissue; letter; male; medical history; microorganism; mycetoma/di [Diagnosis]; neutrophil; perioperative period; psoriasis; skin nodule; swelling; travel; treatment response; cotrimoxazole/dt [Drug Therapy]; cotrimoxazole/po [Oral Drug Administration]; DNA 16S/ec [Endogenous Compound]; rifampicin/dt [Drug Therapy]; RNA 16S/ec [Endogenous Compound]; *Gordonia westfalica.

Candidate Terms

*Gordonia westfalica [other term].

Drug Index Terms

cotrimoxazole / drug therapy / oral drug administration; DNA 16S / endogenous compound; rifampicin / drug therapy; RNA 16S / endogenous compound.

Other Index Terms

Actinobacteria; *actinomycetoma / *drug therapy / *etiology; adult; antibiotic therapy; bacterium culture; bacterium identification; case report; clinical article; clinical assessment; clinical examination; DNA sequencing; Fonsecaea; foot; *Gordonia; histopathology; human; human tissue; Letter; male; medical history; microorganism; mycetoma / diagnosis; neutrophil; perioperative period; psoriasis; skin nodule; swelling; travel; treatment response.

CAS Registry Numbers

8064-90-2 (cotrimoxazole); 13292-46-1 (rifampicin)

Year of Publication

2020

Cite

24.
Bloodstream **infection** caused by **gordonia** terrae: One case report.

Liu Y., Zhong Q., Hang Y., Chen Y., Fang X., Xiao Y., Cao X., Hu L.

Embase
Chinese Journal of Infection and Chemotherapy. 19(4) (pp 435-437), 2019. Article Number: 1009-7708(2019)04-0435-03. Date of Publication: July 2019.

[Article]

AN:
2003352479

Status

Embase

Institution

(Liu, Zhong, Hang, Chen, Fang, Xiao, Cao, Hu) Department of Laboratory Medicine, the Second Affiliated Hospital of Nanchang University, Nanchang 330006, China

Publisher

Editorial Department of Chinese Journal of Infection (E-mail: yguoshi@sh163.net)

Emtree Heading

*Actinobacteria; article; *bloodstream infection; case report; clinical article;
human
; nonhuman; *Gordonia terrae.

Candidate Terms

*Gordonia terrae [other term].

Other Index Terms

*Actinobacteria; Article; *bloodstream infection; case report; clinical article; human; nonhuman.

Year of Publication

2019

Cite

25.
The impact of antibiotic exposure in water and zebrafish gut microbiomes: A 16S rRNA gene-based metagenomic analysis.

Almeida A.R., Alves M., Domingues I., Henriques I.

Embase
Ecotoxicology and Environmental Safety. 186 (no pagination), 2019. Article Number: 109771. Date of Publication: 30 December 2019.
[Article]
AN:
2003379176

In order to supply **human** demand for food, the aquaculture industry has been growing fast in the last years, being fish usually cultivated in overcrowded conditions. Hence, to prevent the rapidly disease spreading, antibiotics may be applied to both sick and healthy animals. Due to its broad spectrum, oxytetracycline (OTC) is one of the most used antibiotics in food-production. Yet, although useful to prevent infections, antibiotics may reshape aquatic animals' microbiome, disturbing hosts' welfare. However, the impact of this exposure to the organism microbiome and its surrounding environment is poorly understood. Then, the objective of this study was to analyze in detail the long-term effect of OTC in both zebrafish gut and water microbiomes. Zebrafish adults were exposed, via water, for two months to three concentrations of OTC (0, 10 and 10000 mug/L). Total DNA was extracted from gut and water samples and the V3-V4 region of the bacterial 16 S rRNA gene was sequenced using Illumina technology. Results of alpha and beta-diversity analyses revealed that long-term exposure to OTC impacted both zebrafish gut and water microbiomes. In water samples, effects were observed even at the lowest (10 mug/L) OTC concentration tested resulting in an increase in Deltaproteobacteria, namely the Myxococcales and Bdellovibrionales orders. On the other hand, effects on zebrafish gut were only observed at the highest concentration with the selection of Alphaproteobacteria and Actinobacteria classes. Although these classes are common in fish gut, the increase of Actinobacteria may represent a health problem since some genera like **Gordonia** are linked to some **human infection** disease. Nevertheless, in both gut and water, it was observed a decrease in Gamaproteobacteria, probably due to OTC mode of action. In silico functional metagenomic analysis revealed that OTC exposure selected general detoxification mechanisms. In addition, the abundance of functional genes involved in Quorum Sensing (QS) increased under OTC exposure suggesting that QS may help bacteria to survive OTC stress. Thus, future studies should consider post-exposure scenarios for a deeper analysis of the water and zebrafish gut resistome, since bacteria may react differently after exposure ceased.
Copyright © 2019 Elsevier Inc.

PMID

31629904 [https://www.ncbi.nlm.nih.gov/pubmed/?term=31629904]

Status

Embase

Institution

(Almeida, Domingues) Department of Biology & CESAM, University of Aveiro, Campus Universitario de Santiago, Aveiro 3810-193, Portugal (Alves) Universidade Catolica Portuguesa, CBQF - Centro de Biotecnologia e Quimica Fina - Laboratorio Associado, Escola Superior de Biotecnologia, Rua Diogo Botelho 1327, Porto 4169-005, Portugal
(Henriques) CESAM & Department of Life Sciences, Faculty of Sciences and Technology, University of Coimbra, Calcada Martins de Freitas, Coimbra 3000-456, Portugal

Publisher

Academic Press

Keyword Heading

Danio rerio, Microbiome, OTU, Piphillin, Tetracycline

Emtree Heading

Actinobacteria; adult; Alphaproteobacteria; article; Bdellovibrionales; computer model; concentration (parameter); Deltaproteobacteria; DNA extraction; *drug exposure; Gammaproteobacteria; gene sequence;
Gordonia

; intestine examination; *intestine flora; *metagenomics; *microbiome; Myxococcales; next generation sequencing; nonhuman; quorum sensing; water sampling; DNA/ec [Endogenous Compound]; *oxytetracycline; *RNA 16S; *water; *16S rRNA gene.

Candidate Terms

*16S rRNA gene [other term].

Drug Index Terms

DNA / endogenous compound; *oxytetracycline; *RNA 16S; *water.

Other Index Terms

Actinobacteria; adult; Alphaproteobacteria; Article; Bdellovibrionales; computer model; concentration (parameter); Deltaproteobacteria; DNA extraction; *drug exposure; Gammaproteobacteria; gene sequence; Gordonia; intestine examination; *intestine flora; *metagenomics; *microbiome; Myxococcales; next generation sequencing; nonhuman; quorum sensing; water sampling.

CAS Registry Numbers

9007-49-2 (DNA); 2058-46-0 (oxytetracycline); 56761-42-3 (oxytetracycline); 79-57-2 (oxytetracycline); 7732-18-5 (water)

Year of Publication

2019

Cite

26.

Sternal osteomyelitis by **Gordonia** Bronchialis in an immunocompetent patient after open heart surgery.

Ambesh P., Kapoor A., Kazmi D., Elsheshtawy M., Shetty V., Lin Y., Kamholz S.

Embase
Annals of Cardiac Anaesthesia. 22(2) (pp 221-224), 2019. Date of Publication: April-June 2019.

[Article]

AN:
627212882

Gordonia is a catalase-positive, aerobic, nocardioform, Gram-positive staining actinomycete that also shows weak acid-fast staining. Several **Gordonia** species are commonly found in the soil. The bacterium has been isolated from the saliva of domesticated/wild dogs as well. In hospitalized patients, most commonly it is found in the setting of intravascular catheter-related infections. However, recent reports show that it is being increasingly isolated from sternal wounds, skin/neoplastic specimens and from pleural effusions. **Gordonia** shares many common characteristics with Rhodococcus and Nocardia. Ergo, it is commonly misrecognized as Nocardia or Rhodococcus. Since this pathogen requires comprehensive morphological and biochemical testing, it is often difficult and cumbersome to isolate the species. Broad-range Polymerase Chain Reaction (PCR) and sequencing with genes like 16S rRNA or hsp65 are used to correctly identify the species. Identification is essential for choosing and narrowing the right antimicrobial agent. Herein, we report our experience with a patient who presented with sternal osteomyelitis after **infection** with this elusive bug.
Copyright © 2019 Medknow Publications. All rights reserved.

PMID

30971609 [https://www.ncbi.nlm.nih.gov/pubmed/?term=30971609]

Status

Embase

Institution

(Ambesh, Kamholz) Department of Internal Medicine, Maimonides Medical Center, New York City 11219, United States (Kapoor) Department of Cardiology, Sanjay Gandhi Post Graduate Institute of Medical Sciences, Lucknow, India
(Kazmi) Department of Cardiology, Era Medical Institute, Lucknow, India
(Elsheshtawy, Shetty) Department of Cardiology, Maimonides Medical Center, New York City, United States
(Lin) Department of Infectious Disease, Maimonides Medical Center, New York City, United States

Publisher

Wolters Kluwer Medknow Publications (B9, Kanara Business Centre, off Link Road, Ghatkopar (E), Mumbai 400 075, India)

Keyword Heading

Gordonia bronchialis, infection, sternal osteomyelitis

Emtree Heading

abscess/co [Complication]; abscess/di [Diagnosis]; abscess/dt [Drug Therapy]; abscess/et [Etiology]; abscess/su [Surgery]; abscess drainage; aged; article; bacterium identification; case report; *chronic osteomyelitis/co [Complication]; *chronic osteomyelitis/di [Diagnosis]; *chronic osteomyelitis/dt [Drug Therapy]; *chronic osteomyelitis/et [Etiology]; *chronic osteomyelitis/su [Surgery]; clinical article; clinical examination; computer assisted tomography; *coronary artery bypass graft; coronary artery disease/su [Surgery]; debridement; *Gordonia;
human
; *immunocompetence; male; microbiological examination; nonhuman; open heart surgery; sternotomy; *sternum; thorax pain; meropenem/cb [Drug Combination]; meropenem/dt [Drug Therapy]; meropenem/iv [Intravenous Drug Administration]; vancomycin/cb [Drug Combination]; vancomycin/dt [Drug Therapy]; vancomycin/iv [Intravenous Drug Administration]; *Gordonia bronchialis.

Candidate Terms

*Gordonia bronchialis [other term].

Drug Index Terms

meropenem / drug combination / drug therapy / intravenous drug administration; vancomycin / drug combination / drug therapy / intravenous drug administration.

Other Index Terms

abscess / complication / diagnosis / drug therapy / etiology / surgery; abscess drainage; aged; Article; bacterium identification; case report; *chronic osteomyelitis / *complication / *diagnosis / *drug therapy / *etiology / *surgery; clinical article; clinical examination; computer assisted tomography; *coronary artery bypass graft; coronary artery disease / surgery; debridement; *Gordonia; human; *immunocompetence; male; microbiological examination; nonhuman; open heart surgery; sternotomy; *sternum; thorax pain.

CAS Registry Numbers

96036-03-2 (meropenem); 1404-90-6 (vancomycin); 1404-93-9 (vancomycin)

Year of Publication

2019

Cite

27.
Case report of cutaneous nodule caused by **Gordonia** bronchialis in an immunocompetent patient after receiving acupuncture.

Choi M.E., Jung C.J., Won C.H., Chang S.E., Lee M.W., Choi J.H., Lee W.J.

Embase
Journal of Dermatology. 46(4) (pp 343-346), 2019. Date of Publication: April 2019.

[Article]

AN:
626219632

Gordonia species were recently found to cause **human infection**. Most **Gordonia** bronchialis infections are associated with sternal wounds and foreign bodies. Here, we present a case of a firm cutaneous nodule caused by G. bronchialis on an immunocompetent patient's lower extremity after receiving acupuncture. Our present case indicates that spontaneous cutaneous **infection** of G. bronchialis can develop even in a healthy patient. With the popularity of complementary and alternative medicine, physicians should be aware that G. bronchialis **infection** can be associated with Oriental medicine similar to mycobacterial **infection**. Recognizing the diverse clinical features of newly emerging **Gordonia** species will facilitate appropriate diagnosis and management of future patients.
Copyright © 2019 Japanese Dermatological Association

PMID

30710379 [https://www.ncbi.nlm.nih.gov/pubmed/?term=30710379]

Status

Embase

Author NameID

Choi, Myoung Eun; ORCID: https://orcid.org/0000-0001-7514-7873

Institution

(Choi, Jung, Won, Chang, Lee, Choi, Lee) Department of Dermatology, Asan Medical Center, University of Ulsan College of Medicine, Seoul, South Korea

Publisher

Blackwell Publishing Ltd

Keyword Heading

actinomycete, Gordonia bronchialis, infection, nodule, skin

Emtree Heading

*acupuncture; adult; antibiotic sensitivity; antibiotic therapy; article; *bacterial skin disease/co [Complication]; *bacterial skin disease/di [Diagnosis]; *bacterial skin disease/dt [Drug Therapy]; case report; clinical article; female; *Gordonia; Gram staining; histopathology;
human
; immunocompetence; middle aged; nonhuman; punch biopsy; RNA sequence; skin nodule; cefpodoxime proxetil/dt [Drug Therapy]; *Gordonia bronchialis.

Candidate Terms

*Gordonia bronchialis [other term].

Drug Index Terms

cefpodoxime proxetil / drug therapy.

Other Index Terms

*acupuncture; adult; antibiotic sensitivity; antibiotic therapy; Article; *bacterial skin disease / *complication / *diagnosis / *drug therapy; case report; clinical article; female; *Gordonia; Gram staining; histopathology; human; immunocompetence; middle aged; nonhuman; punch biopsy; RNA sequence; skin nodule.

CAS Registry Numbers

87239-81-4 (cefpodoxime proxetil)

Year of Publication

2019

Cite

28.

General characteristics and clinical significance of nocardiaand Gordoniagenera.
OBSHCHAYA XAPAKTIEPISTIKA I KIHICHIESKOIE ZHACHIEHIIE PPIEDESTABITIEIE PODEOB NOCARDIA I GORDONIA
<OBSHCHAYA XAPAKTIEPISTIKA I KIHICHIESKOIE ZHACHIEHIIE PPIEDESTABITIEIE PODEOB NOCARDIA I GORDONIA
.>

Lyamin A.V., Zhestkov A.V., Nikitina T.R., Podsevalov V.S., Trofimov A.R., Ismatullin D.D.

Embase
Russian Journal of Infection and Immunity. 9(3-4) (pp 429-436), 2019. Date of Publication: 2019.

[Article]

AN:
2004131280

Over the last years, an increasing attention in modern medical microbiology has been paid to examining Actinomycetaceae, Corynebacteriaceae, Mycobacteriaceae, Nocardiaceae, Gordoniaceae sps. Members of the Mycobacteriaceae family are increasingly examined in research and real-life practice, whereas bacteria belonging to families such as Nocardiaceae and Gordoniaceae remain poorly investigated despite novel methods emerging in practical microbiology that allow to more accurately identify microorganisms. According to the current classification, the genus Nocardia includes over 80 species, most of which rarely result in **human** disease development. Most often, members of the genus Nocardia cause lesions in bronchopulmonary system, which, however, may also cause development of pathological processes in other anatomical sites. Likewise, members of the genus **Gordonia** may also trigger infectious lesions in **human**, which were previously often incorrectly identified as other actinomycetes or mycobacteria. Owing to use of 16S rRNA sequencing, it substantially improved identification of these bacteria. Currently, an increasing number of microorganisms with potential clinical significance has been recorded. In addition, similar to nocardiosis, diverse primary and secondary immunodeficiencies play a primary role in gordonii-associated development of pathological processes. However, an additional risk factor may be represented by pathological conditions associated with ingestion of foreign bodies colonized by such microorganisms. Most often, members the genus Nocardia cause lesions in the bronchopulmonary system able, however, affect other anatomical areas. Half of all cases of pulmonary nocardiosis are accompanied by pathological processes of extrapulmonary localization, whereas as low as 20% of patients manifest with extrapulmonary form of the disease usually occurring when the pathogen spreads hematogenously or via other routes also highlighted by primary pulmonary lesion. Moreover, members of the genus **Gordonia** may result in similar infectious lesions. Currently, the number of aerobic actinomycetes of potential clinical significance is increasing that may be due to their role in diverse pathological processes of various etiologies, which have been more often reported in scientific publications. Few reports regarding infections caused by the genus **Gordonia** ara available which may be due to a paucity of microorganisms isolated from clinical material or false identification as mycobacteria or Nocardia. Similar to nocardiosis, diverse immunodeficiencies play a primary role in the development of pathological processes associated with **Gordonia**. However, an additional risk factor may be linked to pathological conditions associated with the ingestion of foreign bodies colonized by these microorganisms. Available publications allows to underline etiological significance of **Gordonia** in development of cholecystitis, granulomatous skin lesions, eyelid abscess of other soft tissues, granulomatous mastitis, brain abscess and meningitis, as well as external otitis, bronchitis, endocarditis and mediastinitis. In addition, all these microorganisms can cause bacteremia associated with use of a central venous catheter. Owing to emergence of new detection methods as well as elevated rate of immunocompromised patients, and subsequently increased amount of new cases caused by members of the Nocardiaceae and Gordoniaceae families, an interest they rise will grow progressively.
Copyright © 2019 Saint Petersburg Pasteur Institute. All rights reserved.

Status

Embase

Institution

(Lyamin, Zhestkov, Nikitina, Podsevalov, Trofimov, Ismatullin) Samara State Medical University, Gagarin str., 18, Samara 443079, Russian Federation

Publisher

Saint Petersburg Pasteur Institute (E-mail: izdatelstvo@pasteurorg.ru)

Keyword Heading

Antibiotic resistance, Classification, Clinical significance, Gordonia, Nocardia

Emtree Heading

article; bacterial colonization; *bacterium identification; clinical feature; foreign body; *Gordonia;
human
; immune deficiency; immunocompromised patient; infection risk; lung infection; lung lesion; *Nocardia; nocardiosis; nonhuman; RNA sequence; bacterial RNA/ec [Endogenous Compound]; RNA 16S/ec [Endogenous Compound].

Drug Index Terms

bacterial RNA / endogenous compound; RNA 16S / endogenous compound.

Other Index Terms

Article; bacterial colonization; *bacterium identification; clinical feature; foreign body; *Gordonia; human; immune deficiency; immunocompromised patient; infection risk; lung infection; lung lesion; *Nocardia; nocardiosis; nonhuman; RNA sequence.

Year of Publication

2019

Cite

29.
Gordonia bronchialis- Associated endophthalmitis, Oregon, USA.

Choi R., Strnad L., Flaxel C.J., Lauer A.K., Suhler E.B.

Embase
Emerging Infectious Diseases. 25(5) (pp 1017-1019), 2019. Date of Publication: May 2019.

[Letter]

AN:
2001936375

Gordonia bronchialis is an aerobic actinomycetes that rarely causes infections in humans. Few reports describe **Gordonia** spp. causing eye-related infections. We report a case of chronic infectious endophthalmitis in Oregon, USA, associated with **infection** by G. bronchialis.
Copyright © 2019, Centers for Disease Control and Prevention (CDC). All rights reserved.

PMID

31002051 [https://www.ncbi.nlm.nih.gov/pubmed/?term=31002051]

Status

Embase

Institution

(Flaxel, Lauer, Suhler) Casey Eye Institute, Portland, OR, United States (Strnad) Oregon Health and Science University, Portland, United States
(Suhler) Veterans Administration Portland Health Care System, Portland, United States
(Choi) Casey Eye Institute, Oregon Health and Science University, Portland, OR, United States

Publisher

Centers for Disease Control and Prevention (CDC) (E-mail: cdcinfo@cdc.gov)

Emtree Heading

*Actinobacteria; adult; aerobic bacterium; antibiotic sensitivity; B scan; *bacterial eye infection/di [Diagnosis]; *bacterial eye infection/dt [Drug Therapy]; blood cell count; bloodstream infection; case report; cataract extraction; clinical article; echography; *endophthalmitis/di [Diagnosis]; *endophthalmitis/dt [Drug Therapy]; erythrocyte sedimentation rate; eye inflammation; female; flow cytometry; fluorescent treponema antibody test; gene sequence;
human
; hypopyon; intraocular pressure; laboratory test; letter; matrix assisted laser desorption ionization time of flight mass spectrometry; minimum inhibitory concentration; pars plana vitrectomy; polymerase chain reaction; recurrent disease; skin abscess; thorax radiography; tuberculosis; visual acuity; amikacin/dt [Drug Therapy]; amikacin/vi [Intravitreal Drug Administration]; aminoglycoside; amoxicillin/dt [Drug Therapy]; amoxicillin/vi [Intravitreal Drug Administration]; angiotensin; antinuclear antibody; ceftriaxone/dt [Drug Therapy]; ceftriaxone/vi [Intravitreal Drug Administration]; cephalosporin derivative; ciprofloxacin; clavulanic acid; corticosteroid/tp [Topical Drug Administration]; leukocyte antigen; moxifloxacin/dt [Drug Therapy]; moxifloxacin/po [Oral Drug Administration]; quinoline derived antiinfective agent; rheumatoid factor; RNA 16S; intravascular catheter; lens implant; *Gordonia bronchialis.

Candidate Terms

*Gordonia bronchialis [other term].

Device Index Terms

intravascular catheter; lens implant.

Drug Index Terms

amikacin / drug therapy / intravitreal drug administration; aminoglycoside; amoxicillin / drug therapy / intravitreal drug administration; angiotensin; antinuclear antibody; ceftriaxone / drug therapy / intravitreal drug administration; cephalosporin derivative; ciprofloxacin; clavulanic acid; corticosteroid / topical drug administration; leukocyte antigen; moxifloxacin / drug therapy / oral drug administration; quinoline derived antiinfective agent; rheumatoid factor; RNA 16S.

Other Index Terms

*Actinobacteria; adult; aerobic bacterium; antibiotic sensitivity; B scan; *bacterial eye infection / *diagnosis / *drug therapy; blood cell count; bloodstream infection; case report; cataract extraction; clinical article; echography; *endophthalmitis / *diagnosis / *drug therapy; erythrocyte sedimentation rate; eye inflammation; female; flow cytometry; fluorescent treponema antibody test; gene sequence; human; hypopyon; intraocular pressure; laboratory test; Letter; matrix assisted laser desorption ionization time of flight mass spectrometry; minimum inhibitory concentration; pars plana vitrectomy; polymerase chain reaction; recurrent disease; skin abscess; thorax radiography; tuberculosis; visual acuity.

CAS Registry Numbers

37517-28-5 (amikacin); 39831-55-5 (amikacin); 26787-78-0 (amoxicillin); 34642-77-8 (amoxicillin); 61336-70-7 (amoxicillin); 1407-47-2 (angiotensin); 73384-59-5 (ceftriaxone); 74578-69-1 (ceftriaxone); 85721-33-1 (ciprofloxacin); 58001-44-8 (clavulanic acid); 151096-09-2 (moxifloxacin); 9009-79-4 (rheumatoid factor)

Year of Publication

2019

Cite

30.
Mycetoma in China: A Case Report and Review of the Literature.

Wang R., Yao X., Li R.

Embase
Mycopathologia. 184(2) (pp 327-334), 2019. Date of Publication: 01 Apr 2019.

[Article]

AN:
626883612

Mycetoma is a chronic granulomatous infectious disease that can affect the skin, subcutaneous tissue, fascia and bone. It can be caused by filamentous bacteria or fungi and usually involves the legs and feet. Mycetoma is endemic in tropical and subtropical regions and is easily misdiagnosed in clinical practice because of its nonspecific clinical features and lack of awareness of the disease. Although mycetoma is very rare in mainland China, an increasing number of cases have been reported in recent years. Here, we report a case of mycetoma in a patient who was misdiagnosed many years before receiving the correct treatment, leading to disease progression and motion limitation. The grains that represent microorganismal colonies were important clues for diagnosis. We also reviewed reported cases of mycetoma in mainland China. The majority of cases were reported from southern regions. Actinomycetoma was more commonly reported than was eumycetoma. The causative agents of actinomycetoma included Nocardia brasiliensis, N. asteroides, N. otitidiscaviarum, N. ninae and **Gordonia** terrae, and the causative fungi of eumycetoma were identified as Madurella mycetomatis, Fonsecaea pedrosoi and Acremonium falciforme. Notably, the diagnosis of mycetoma was delayed from months to decades in all of the patients, likely due to a lack of clinical experience. Our literature review suggests the importance of increased awareness of mycetoma in clinical practice, especially in non-endemic regions. Further investigative studies are needed to determine the real incidence of the disease in China.

PMID

30887177 [https://www.ncbi.nlm.nih.gov/pubmed/?term=30887177]

Institution

(Wang) Department of Dermatology, Peking University First Hospital, 8 Xishiku Street ,Xicheng District, Beijing 100034, China (Wang) Beijing Key Laboratory of Molecular Diagnosis on Dermatoses, Beijing, China
(Wang) Research Center for Medical Mycology, Peking University, Beijing, China
(Yao) Department of Dermatology, Peking University People's Hospital, Beijing, China
(Li) Department of Dermatology, Peking University First Hospital, 8 Xishiku Street ,Xicheng District, Beijing 100034, China
(Li) Beijing Key Laboratory of Molecular Diagnosis on Dermatoses, Beijing, China
(Li) Research Center for Medical Mycology, Peking University, Beijing, China

Publisher

NLM (Medline)

Keyword Heading

Actinomycetoma, China, Eumycetoma, Mycetoma, Nocardia

Emtree Heading

adult; aged; case report; China; classification; cytochemistry; female; fungus;
human
; incidence; isolation and purification; male; *microbiology; microscopy; middle aged; mycetoma/di [Diagnosis]; mycetoma/ep [Epidemiology]; Nocardia; *pathology; skin; young adult.

Other Index Terms

adult; aged; case report; China; classification; cytochemistry; female; fungus; human; incidence; isolation and purification; male; *microbiology; microscopy; middle aged; mycetoma / diagnosis / epidemiology; Nocardia; *pathology; skin; young adult.

Year of Publication

2019

Cite

31.
Nipple piercing-associated infections: Case series with review of the literature and an association with granulomatous mastitis.

Baker G.

Embase
Modern Pathology. Conference: 108th Annual Meeting of the United States and Canadian Academy of Pathology, USCAP 2019. National Harbor, MD United States. 32(3) (no pagination), 2019. Date of Publication: March 2019.

[Conference Abstract]

AN:
631812585

Background: Nipple piercing (NP) has been cited as a risk factor for the development of breast abscess; however, the prevalence of NP and the frequency of NP-associated **infection** (NPAI) are unknown. The relevant literature consists predominantly of case reports that lack adequate histologic description. The purpose of this study is to evaluate the microorganisms associated with (w) NPAI and to assess the pattern of associated inflammatory infiltrate.
Design(s): A ten-year single-institution review was performed to identify cases of NPAI; associated microbiology results were identified and the corresponding slides were evaluated. The literature was reviewed for reports in which **infection** was associated w NP.
Result(s): 5 cases of NPAI were identified on institutional review (age range 19-37yo, mean 30yo, median 33yo; all female). Bacterial culture was performed in 4 of 5 cases and all were positive: 2 w coagulase negative Staphylococci, 1 w Corynebacterium amycolatum/xerosis (Propionibacterium acnes and Haemophilus parainfluenzae also present), and 1 w rare gram positive cocci not otherwise specified (NOS). Histologic evaluation demonstrated granulomatous inflammation in all 5 cases; 4 had suppurative granulomatous mastitis w or w/out cystic spaces. Literature review identified 23 additional cases of NPAI (age range 15-60yo, median and mean 28yo; 20 female, 3 male). Culture was performed in 16 cases and 15 were positive (6 polymicrobial; one additional case had positive gram stain only). The organisms identified were Staphylococcus spp (n=6), atypical mycobacteria (n=5), Streptococcus spp (n=4), Prevotella spp (n=2), Actinomyces spp (n=1), **Gordonia** terrae (n=1), Nocardia spp (n=1), Peptostreptococcus spp (n=1), diphtheroids NOS (n=1), and gram positive bacilli NOS (n=1). Histologic description was available for 5 cases: all had granulomatous inflammation.
Conclusion(s): All cases in the present series and at least 5 cases in the literature had granulomatous inflammation. The presence of suppurative granulomatous inflammation w/or w/out cystic spaces is characteristic of Cystic Neutrophilic Granulomatous Mastitis, a disease associated with coryneform bacteria and only reported in one prior case of NPAI. To the author's knowledge, this represents the largest case series of NPAI in the pathology literature and provides the greatest information regarding histology of NPAI. Additionally, the spectrum of NPAI-associated microorganisms is expanded.

Status

CONFERENCE ABSTRACT

Institution

(Baker) Beth Israel Deaconess Medical Center, Boston, MA, United States

Publisher

Springer Nature

Emtree Heading

Actinomyces; adult; age; atypical Mycobacterium; bacterium culture; case report; *case study; clinical article; coagulase negative Staphylococcus; conference abstract; Corynebacterium amycolatum; coryneform bacterium; female;
Gordonia
; Gram positive bacterium; Gram staining; *granulomatous inflammation; Haemophilus parainfluenzae; histology; histopathology;
human
; human cell; human tissue; inflammatory infiltrate; institutional review; male; *mastitis; microbiology; neutrophil; *nipple piercing; Nocardia; nonhuman; Peptostreptococcus; Prevotella; Propionibacterium acnes; Streptococcus; xerosis.

Other Index Terms

Actinomyces; adult; age; atypical Mycobacterium; bacterium culture; case report; *case study; clinical article; coagulase negative Staphylococcus; conference abstract; Corynebacterium amycolatum; coryneform bacterium; female; Gordonia; Gram positive bacterium; Gram staining; *granulomatous inflammation; Haemophilus parainfluenzae; histology; histopathology; human; human cell; human tissue; inflammatory infiltrate; institutional review; male; *mastitis; microbiology; neutrophil; *nipple piercing; Nocardia; nonhuman; Peptostreptococcus; Prevotella; Propionibacterium acnes; Streptococcus; xerosis.

Year of Publication

2019

Cite

32.
Image Gallery: Actinomycetoma caused by **Gordonia** terrae in an immunocompetent woman.

Wang S., Yang Q., Ye H., Si Z., Zhao H., Qiao J.

Embase
British Journal of Dermatology. 179(2) (pp e90), 2018. Date of Publication: August 2018.

[Letter]

AN:
623639545

PMID

30141561 [https://www.ncbi.nlm.nih.gov/pubmed/?term=30141561]

Status

Embase

Author NameID

Qiao J.; ORCID: https://orcid.org/0000-0003-3386-297X

Institution

(Wang, Si, Zhao) Department of Dermatology, Beilun People's Hospital, Ningbo, China (Yang) State Key Laboratory for Diagnosis and Treatment of Infectious Diseases, Collaborative Innovation Center for Diagnosis and Treatment of Infectious Diseases, College of Medicine, Zhejiang University, Hangzhou, China
(Ye) Center of Clinical Laboratory, Beilun People's Hospital, Ningbo, China
(Qiao) Department of Dermatology, The First Affiliated Hospital, College of Medicine, Zhejiang University, Hangzhou, China

Publisher

Blackwell Publishing Ltd

Emtree Heading

*actinomycetoma/di [Diagnosis]; *actinomycetoma/dt [Drug Therapy]; *actinomycetoma/et [Etiology]; adult; case report; cellulitis; clinical article; female; foot disease; *Gordonia; grain; histopathology;
human
; human tissue; immunocompromised patient; letter; medical history; nuclear magnetic resonance imaging; priority journal; RNA sequence; skin biopsy; tissue culture; treatment duration; amoxicillin/dt [Drug Therapy]; clavulanic acid/dt [Drug Therapy]; RNA

16S/ec [Endogenous Compound]; *Gordonia terrae.

Candidate Terms

*Gordonia terrae [other term].

Drug Index Terms

amoxicillin / drug therapy; clavulanic acid / drug therapy; RNA 16S / endogenous compound.

Other Index Terms

*actinomycetoma / *diagnosis / *drug therapy / *etiology; adult; case report; cellulitis; clinical article; female; foot disease; *Gordonia; grain; histopathology; human; human tissue; immunocompromised patient; Letter; medical history; nuclear magnetic resonance imaging; priority journal; RNA sequence; skin biopsy; tissue culture; treatment duration.

CAS Registry Numbers

26787-78-0 (amoxicillin); 34642-77-8 (amoxicillin); 61336-70-7 (amoxicillin); 58001-44-8 (clavulanic acid)

Year of Publication

2018

Cite

33.

Determinants of the Nasal Microbiome: Pilot Study of Effects of Intranasal Medication Use.

Ramakrishnan V.R., Holt J., Nelson L.F., Ir D., Robertson C.E., Frank D.N.

Embase
Allergy and Rhinology. 9 (no pagination), 2018. Date of Publication: 01 Aug 2018.

[Article]

AN:
624185682

Introduction: A role for bacteria and other microbes has long been suspected in the chronic inflammatory sinonasal diseases. Recent studies utilizing culture-independent, sequence-based identification have demonstrated aberrant shifts in the sinus microbiota of chronic rhinosinusitis subjects, compared with ostensibly healthy controls. Examining how such microbiota shifts occur and the potential for physician-prescribed interventions to influence microbiota dynamics are the topics of the current article.
Method(s): The nasal cavity microbiota of 5 subjects was serially examined over an 8-week period using pan-bacterial 16S rRNA gene sequencing. Four of the subjects were administered topical mometasone furoate spray, while 1 subject underwent a mupirocin decolonization procedure in anticipation of orthopedic surgery.
Result(s): Measures of microbial diversity were unaffected by intranasal treatment in 2 patients and were markedly increased in the remaining 3. The increase in microbial diversity was related to clearance of Moraxella spp. and a simultaneous increase in members of the phylum Actinobacteria. Both effects persisted at least 2 weeks beyond cessation of treatment. Transient changes in the relative abundance of several bacterial genera, including Staphylococcus and Priopionibacteria, were also observed during treatment.
Conclusion(s): The effects of intranasal steroids on the sinonasal microbiome are poorly understood, despite their widespread use in treating chronic sinonasal inflammatory disorders. In this longitudinal study, administration of intranasal mometasone furoate or mupirocin resulted in shifts in microbial diversity that persisted to some degree following treatment cessation. Further characterization of these effects as well as elucidation of the mechanism(s) underlying these changes is needed.
Copyright © The Author(s) 2018.

Status

Embase

Institution

(Ramakrishnan, Holt) Department of Otolaryngology-Head and Neck Surgery, University of Colorado School of Medicine, Aurora, CO, United States (Holt) Department of Otolaryngology-Head and Neck Surgery, Oregon Health & Science University, Portland, OR,

United States
(Nelson, Ir, Robertson, Frank) Division of Infectious Diseases, University of Colorado School of Medicine, Aurora, CO, United States

Publisher

SAGE Publications Ltd (E-mail: info@sagepub.co.uk)

Keyword Heading

anterior nares, bacteria, microbiome, nasal steroid, rhinitis, sinusitis

Emtree Heading

Actinobacteria; adult; article; chronic rhinitis/dt [Drug Therapy]; clinical article; commensal; Corynebacterium; *drug use; female; Firmicutes; gene sequence;
Gordonia
;
human
; male; microbial diversity; *microbiome; Moraxella; pathogen load; pilot study; polymerase chain reaction; priority journal; Propionibacterium; prospective study; Proteobacteria; pyrosequencing; Staphylococcus; treatment duration; mometasone furoate/na [Intranasal Drug Administration]; mometasone furoate/tp [Topical Drug Administration]; pseudomonic acid/dt [Drug Therapy]; RNA 16S/ec [Endogenous Compound].

Drug Index Terms

mometasone furoate / intranasal drug administration / topical drug administration; pseudomonic acid / drug therapy; RNA 16S / endogenous compound.

Other Index Terms

Actinobacteria; adult; Article; chronic rhinitis / drug therapy; clinical article; commensal; Corynebacterium; *drug use; female; Firmicutes; gene sequence; Gordonia; human; male; microbial diversity; *microbiome; Moraxella; pathogen load; pilot study; polymerase chain reaction; priority journal; Propionibacterium; prospective study; Proteobacteria; pyrosequencing; Staphylococcus; treatment duration.

Drug Trade Name

nasonex: Merck

CAS Registry Numbers

83919-23-7 (mometasone furoate); 105102-22-5 (mometasone furoate); 12650-69-0 (pseudomonic acid); 40980-51-6 (pseudomonic acid); 71980-98-8 (pseudomonic acid)

Year of Publication

2018

Cite

34.
A case of disseminated crypotococcal **infection** with a rare twist.

Puri I., Ahmed A., Dhillon G.

Embase
Journal of Hospital Medicine. Conference: Hospital Medicine, HM 2018. Orlando, FL United States. 13(4 Supplement 1) (no pagination), 2018. Date of Publication: 2018.

[Conference Abstract]

AN:
629665868

Case Presentation: A 69-year-old patient was referred to us by his PCP for subacute onset of confusion. Two months prior to presentation he was diagnosed with Focal segmental glomerulonephritis and had been on immunosuppressive therapy with Mycophenolate mofetil and Prednisone. He subsequently developed productive cough, and shortness of breath and was found to have a cavitary lung lesion on chest X-ray. Bronchoscopy done at an outside hospital revealed a right middle lobe cavitary pneumonia. Transbronchial biopsy was positive for Cryptococcus Neoformans and he was discharged home on PO fluconazole. Two weeks prior to presentation he developed confusion, lethargy and gait abnormality. On physical examination he had generalized anasarca, lethargy, ataxia, and had diffuse erythematous

papules and plaques all over his torso. A Lumbar puncture was done at our hospital which revealed < 5 WBC, 123 mg/dl of protein and < 10 mg/dl of glucose. CSF PCR for Cryptococcus was negative. Given his history of immunosuppression, recent cryptococcal pneumonia, and focal neurological (ataxia, episodes of complex partial seizure) and cutaneous signs patient was presumptively treated for disseminated cryptococcal meningoencephalitis. Antifungal induction therapy was started with Liposomal Amphotericin B and Flucytosine. CSF fungal culture revealed Cryptococcus neoformans and confirmed our diagnosis of disseminated Cryptococcal meningoencephalitis. Cryptococcal titre on CSF was reported as 1:2560. Due to the lack of clinical response after patient had been on 12 days of Induction antifungal therapy, a second lumbar puncture was performed which revealed Glucose 16 mg/dl, Protein 144 mg/dl WBC 32 with 58% lymphocytes. Cryptococcal titers improved to 1:320 on the second CSF sample. Induction phase was continued for 2 weeks and then transitioned to PO fluconazole since serological improvement in Cryptococcal titer was noted. Two weeks after the second LP, the CSF fluid from the second Lumber puncture reported a new organism (initially misidentified as Rhodococcus), which was identified as **Gordonia** Bronchialis. Sputum sample was also positive for **Gordonia** Bronchialis. In the light of this additional information, patient was treated with IV Vancomycin and Ceftriaxone. Significant neurological improvement was noted with resolution of seizures, ataxia and confusion.

Discussion(s): Most patients with disseminated Cryptococcus **infection** are immunocompromised. It can take several weeks for fungal smear and culture results to be finalized, and delay in treatment approach can be avoided by keeping high clinical suspicion for disseminated cryptococcal **infection**. **Gordonia** species are gram positive, weakly acid-fast aerobic coccobacilli and belong to the actinomycetes family. It rarely causes infections in humans. Microbiologic diagnosis of **Gordonia** species is challenging, often leading to incorrect identification as Rhodococcus or Nocardia, as occurred in this case.

Conclusion(s): We report the first case of **Gordonia** Bronchialis meningitis in an immunosuppressed patient with disseminated cryptococcal meningoencephalitis. Microbiologic diagnosis and management of **Gordonia** species remains challenging in absence of any formal guidelines. (Figure Presented).

Status

CONFERENCE ABSTRACT

Institution

(Puri) Peninsula Regional Medical center, Salisbury, MD, United States (Ahmed, Dhillon) Peninsula Regional Medical Center

Publisher

Frontline Medical Communications

Keyword Heading

Disseminated Cryptococcal infection, Gordonia Bronchialis, Immunocompromised, Meningoencephalitis, Pneumonia

Emtree Heading

aged; anasarca; antifungal therapy; ataxia; bronchoscopy; case report; cerebrospinal fluid; clinical article; complex partial seizure; coughing; diagnosis; drug combination; drug therapy; dyspnea; gait; glomerulonephritis; *Gordonia;
human
; human cell; human tissue; immunosuppressive treatment; lethargy; lumbar puncture; lung lesion; lymphocyte; male; *meningoencephalitis; nervous system; Nocardia; nonhuman; papule; physical examination; polymerase chain reaction; practice guideline; *pulmonary cryptococcosis; Rhodococcus; skin; sputum; thorax radiography; transbronchial biopsy; trunk; amphotericin B lipid complex; ceftriaxone; fluconazole; flucytosine; glucose; mycophenolate mofetil; prednisone; vancomycin; conference abstract.

Candidate Terms

conference abstract [other term].

Drug Index Terms

amphotericin B lipid complex; ceftriaxone; fluconazole; flucytosine; glucose; mycophenolate mofetil; prednisone; vancomycin.

Other Index Terms

aged; anasarca; antifungal therapy; ataxia; bronchoscopy; case report; cerebrospinal fluid; clinical article; complex partial seizure; coughing; diagnosis; drug combination; drug therapy; dyspnea; gait; glomerulonephritis; *Gordonia; human; human cell; human tissue; immunosuppressive treatment; lethargy; lumbar puncture; lung lesion; lymphocyte; male; *meningoencephalitis; nervous system; Nocardia; nonhuman; papule; physical examination; polymerase chain reaction; practice guideline; *pulmonary cryptococcosis; Rhodococcus; skin; sputum; thorax radiography; transbronchial biopsy; trunk.

CAS Registry Numbers

73384-59-5 (ceftriaxone); 74578-69-1 (ceftriaxone); 86386-73-4 (fluconazole); 2022-85-7 (flucytosine); 50-99-7 (glucose); 84778-64-3

(glucose); 116680-01-4 (mycophenolate mofetil); 128794-94-5 (mycophenolate mofetil); 53-03-2 (prednisone); 1404-90-6 (vancomycin); 1404-93-9 (vancomycin)

Year of Publication

2018

Cite

35.

Comment on the letter 'Brain abscess due to Nocardia **infection** in an immunocompetent patient with asymptomatic pulmonary alveolar proteinosis' by de Leon et al.

Keikha M.

Embase

Acta Neurologica Belgica. 120(2) (pp 387-388), 2020. Date of Publication: 01 Apr 2020.

[Letter]

AN:
619366856

PMID

29164406 [https://www.ncbi.nlm.nih.gov/pubmed/?term=29164406]

Status

Embase

Institution

(Keikha) Department of Microbiology, School of Medicine, Isfahan University of Medical Sciences, Isfahan, Iran, Islamic Republic of

Publisher

Springer

Emtree Heading

antibiotic resistance; antibiotic sensitivity; *brain abscess;
Gordonia
;
human
; letter; *lung alveolus proteinosis; methicillin resistant Staphylococcus aureus; multidrug resistance; Mycobacterium tuberculosis; *nocardiosis/co [Complication]; phenotype; proteinosis; Staphylococcus aureus; systemic mycosis.

Other Index Terms

antibiotic resistance; antibiotic sensitivity; *brain abscess; Gordonia; human; Letter; *lung alveolus proteinosis; methicillin resistant Staphylococcus aureus; multidrug resistance; Mycobacterium tuberculosis; *nocardiosis / *complication; phenotype; proteinosis; Staphylococcus aureus; systemic mycosis.

Year of Publication

2020

Cite

36.

First case report of **Gordonia** aichiensis bacteremia.
Premier cas clinique de bacteriemie a Gordonia aichiensis
<Premier cas clinique de bacteriemie a
Gordonia
aichiensis.>

Thomas E., Lejeune F., Caillon J., Wiertlewski S., Cremet L.

Embase

Medecine et Maladies Infectieuses. 47(7) (pp 508-509), 2017. Date of Publication: November 2017.

[Letter]

AN:
618394280

PMID

28943173 [https://www.ncbi.nlm.nih.gov/pubmed/?term=28943173]

Status

Embase

Author NameID

Thomas E.; ORCID: https://orcid.org/0000-0001-5731-7939

Institution

(Thomas, Caillon, Cremet) Service de bacteriologie-hygiene, CHU de Nantes, 9, quai Moncousu, cedex 1, Nantes 44093, France
(Lejeune, Wiertlewski) Service de neurologie, CHU de Nantes, boulevard Jacques-Monod, Saint-Herblain, cedex 1, Nantes 44093, France

Publisher

Elsevier Masson SAS (62 rue Camille Desmoulins, Issy les Moulineaux Cedex 92442, France)

Keyword Heading

Bacteremia, Daptomycin, Gordonia aichiensis

Emtree Heading

bacteremia; case report; clinical article; *Gordonia;
human
; letter; nonhuman; *Gordonia aichiensis.

Candidate Terms

*Gordonia aichiensis [other term].

Other Index Terms

bacteremia; case report; clinical article; *Gordonia; human; Letter; nonhuman.

Year of Publication

2017

Cite

37.
A case of successfully treated relapsing peritoneal dialysis-associated peritonitis caused by **Gordonia** bronchialis in a farmer.

Sukackiene D., Rimsevicius L., Kiveryte S., Marcinkeviciene K., Bratchikov M., Zokaityte D., Tyla R., Laucyte-Cibulskiene A., Miglinas M.

Embase
Nephrologie et Therapeutique. 14(2) (pp 109-111), 2018. Date of Publication: April 2018.

[Article]

AN:
619533234

Gordonia species are aerobic, weakly acid-fast, Gram-positive pathogens that rarely cause **human** infections, usually in immunocompromised patients. It is uncommon bacilli in cases of peritoneal dialysis-related peritonitis. The small number of infections with **Gordonia** species reported for humans may be stipulated by the difficulty in identifying the organism using conventional techniques. Careful review of Gram stains and modified-acid-fast stains should be done, so that confusion with other actinomycetes is minimized, pending the genotypic identification. Here we report a case that was caused by **Gordonia** bronchialis and thus required different considerations of treatment.
Copyright © 2017 Societe francophone de nephrologie, dialyse et transplantation

PMID

29198961 [https://www.ncbi.nlm.nih.gov/pubmed/?term=29198961]

Status

Embase

Institution

(Sukackiene, Rimsevicius, Zokaityte, Tyla, Laucyte-Cibulskiene, Miglinas) Nephrology center, faculty of medicine, Vilnius university, Santariskiu 2, Vilnius 08661, Lithuania (Kiveryte, Marcinkeviciene) Laboratory medicine center, faculty of medicine, Vilnius university, Santariskiu 2, Vilnius 08661, Lithuania
(Bratchikov) Department of physiology, biochemistry, microbiology and laboratory medicine, faculty of medicine, Vilnius university, Santariskiu 2, Vilnius 08661, Lithuania

Publisher

Elsevier Masson SAS (62 rue Camille Desmoulins, Issy les Moulineaux Cedex 92442, France)

Keyword Heading

Catheter exchange, Gordonia bronchialis, Peritonitis

Emtree Heading

adult; agricultural worker; antibiotic sensitivity; antibiotic therapy; article; *bacterial peritonitis/co [Complication]; *bacterial peritonitis/dt [Drug Therapy]; *bacterial peritonitis/su [Surgery]; case report; catheter removal; chronic kidney failure/th [Therapy]; clinical article; confusion; focal glomerulosclerosis; *Gordonia;

human

; male; matrix assisted laser desorption ionization time of flight mass spectrometry; monotherapy; nonhuman; *peritoneal dialysis; *relapse; sequence alignment; Tenckhoff catheter; amoxicillin plus clavulanic acid; ciprofloxacin; clindamycin; gentamicin/cb [Drug Combination]; gentamicin/dt [Drug Therapy]; gentamicin/ip [Intraperitoneal Drug Administration]; penicillin derivative; RNA 16S; vancomycin/cb [Drug Combination]; vancomycin/dt [Drug Therapy]; vancomycin/ip [Intraperitoneal Drug Administration]; *peritoneal catheter/am [Adverse Device Effect]; *Gordonia bronchialis.

Candidate Terms

*Gordonia bronchialis [other term].

Device Index Terms

*peritoneal catheter / *adverse device effect.

Drug Index Terms

amoxicillin plus clavulanic acid; ciprofloxacin; clindamycin; gentamicin / drug combination / drug therapy / intraperitoneal drug administration; penicillin derivative; RNA 16S; vancomycin / drug combination / drug therapy / intraperitoneal drug administration.

Other Index Terms

adult; agricultural worker; antibiotic sensitivity; antibiotic therapy; Article; *bacterial peritonitis / *complication / *drug therapy / *surgery; case report; catheter removal; chronic kidney failure / therapy; clinical article; confusion; focal glomerulosclerosis; *Gordonia; human; male; matrix assisted laser desorption ionization time of flight mass spectrometry; monotherapy; nonhuman; *peritoneal dialysis; *relapse; sequence alignment; Tenckhoff catheter.

CAS Registry Numbers

74469-00-4 (amoxicillin plus clavulanic acid); 79198-29-1 (amoxicillin plus clavulanic acid); 85721-33-1 (ciprofloxacin); 18323-44-9 (clindamycin); 1392-48-9 (gentamicin); 1403-66-3 (gentamicin); 1405-41-0 (gentamicin); 1404-90-6 (vancomycin); 1404-93-9 (vancomycin)

Year of Publication

2018

Cite

38.

Madura foot caused by **Gordonia** terrae misdiagnosed as Nocardia.

Zampella J.G., Kwatra S.G., Kazi N., Aguh C.

Embase
Australasian Journal of Dermatology. 58(3) (pp e129-e131), 2017. Date of Publication: August 2017.

[Article]

AN:
610765868

Actinomycetomas are soft tissue bacterial infections that are in the differential for unusual masses of the extremities. Typical infectious agents include Actinomyces and Nocardia and are treated with long-term antibiotics. We report a rare case of **Gordonia** actinomycetoma that was misdiagnosed as Nocardia and subsequently required surgical excision in addition to antibiotic therapy.
Copyright © 2016 The Australasian College of Dermatologists

PMID

27270783 [https://www.ncbi.nlm.nih.gov/pubmed/?term=27270783]

Status

Embase

Institution

(Zampella, Kwatra, Kazi, Aguh) Department of Dermatology, Johns Hopkins Hospital, Baltimore, MD, United States

Publisher

Blackwell Publishing

Keyword Heading

actinomycetoma, Gordonia terrae, Madura foot

Emtree Heading

actinomycetoma/di [Diagnosis]; actinomycetoma/dt [Drug Therapy]; adult; antibiotic resistance; antibiotic therapy; article; case report; cellulitis/di [Diagnosis]; clinical article; *diagnostic error; differential diagnosis; drug substitution; drug treatment failure; drug withdrawal; excision; female; foot ulcer; *Gordonia; histopathology;
human
; human tissue; *maduromycosis/di [Diagnosis]; *maduromycosis/dt [Drug Therapy]; maduromycosis/dt [Drug Therapy]; melanoma/di [Diagnosis]; *Nocardia; nocardiosis/di [Diagnosis]; nonhuman; nuclear magnetic resonance imaging; recurrent infection; skin biopsy; squamous cell carcinoma/di [Diagnosis]; tissue culture; amoxicillin/cb [Drug Combination]; amoxicillin/dt [Drug Therapy]; clavulanic acid/cb [Drug Combination]; clavulanic acid/dt [Drug Therapy]; penicillin derivative/dt [Drug Therapy]; penicillin derivative/po [Oral Drug Administration]; RNA 16S/ec [Endogenous Compound]; *Gordonia terrae.

Candidate Terms

*Gordonia terrae [other term].

Drug Index Terms

amoxicillin / drug combination / drug therapy; clavulanic acid / drug combination / drug therapy; penicillin derivative / drug therapy / oral drug administration; RNA 16S / endogenous compound.

Other Index Terms

actinomycetoma / diagnosis / drug therapy; adult; antibiotic resistance; antibiotic therapy; Article; case report; cellulitis / diagnosis; clinical article; *diagnostic error; differential diagnosis; drug substitution; drug treatment failure; drug withdrawal; excision; female; foot ulcer; *Gordonia; histopathology; human; human tissue; *maduromycosis / *diagnosis / *drug therapy; maduromycosis / drug therapy; melanoma / diagnosis; *Nocardia; nocardiosis / diagnosis; nonhuman; nuclear magnetic resonance imaging; recurrent infection; skin biopsy; squamous cell carcinoma / diagnosis; tissue culture.

CAS Registry Numbers

26787-78-0 (amoxicillin); 34642-77-8 (amoxicillin); 61336-70-7 (amoxicillin); 58001-44-8 (clavulanic acid)

Year of Publication

2017

Cite

39.
Cerebrospinal fluid shunt-associated meningitis caused by **Gordonia** sputi: Case report and review of the literature.

Martin Iglesias D., Barrios A., Domingo D., Sanchez P., Sanchez M., Ruiz-Dassy A., Miqueleiz A., Sanz J.

Embase
Infezioni in Medicina. 25(2) (pp 174-178), 2017. Date of Publication: 2017.

[Review]

AN:
616875005

We report the first known case of cerebrospinal fluid (CSF) shunt-associated meningitis caused by **Gordonia** sputi and review published cases of **Gordonia** CNS infections.
Copyright © 2017, EDIMES Edizioni Medico Scientifiche. All rights reserved.

PMID

28603239 [https://www.ncbi.nlm.nih.gov/pubmed/?term=28603239]

Status

Embase

Institution

(Martin Iglesias, Barrios, Sanchez, Sanchez, Ruiz-Dassy, Sanz) Internal Medicine-Infectious Diseases Department, Hospital Universitario de La Princesa, Madrid, Spain (Domingo, Miqueleiz) Microbiology Department, Hospital Universitario de La Princesa, Madrid, Spain

Publisher

EDIMES Edizioni Medico Scientifiche (Via Riviera 39, Pavia 27100, Italy. E-mail: infezmed@libero.it)

Keyword Heading

Central nervous system infection, Cerebrospinal fluid, Gordonia sputi, Meningitis, Shunt

Emtree Heading

abdominal abscess; bacterial endocarditis; *bacterial meningitis/co [Complication]; *bacterial meningitis/di [Diagnosis]; *bacterial meningitis/dt [Drug Therapy]; *bacterial meningitis/et [Etiology]; bacterial meningitis/dt [Drug Therapy]; bacterium identification; brain ventricle peritoneum shunt; case report; cerebrospinal fluid; *cerebrospinal fluid shunting; cognitive defect; diagnostic error; drug substitution; fever;
Gordonia
; granulocyte;
human
; limb weakness; malaise; matrix assisted laser desorption ionization time of flight mass spectrometry; nuclear magnetic resonance imaging; review; transthoracic echocardiography; urinary tract infection; urine incontinence; ampicillin/dt [Drug Therapy]; ceftriaxone/dt [Drug Therapy]; ciprofloxacin; gentamicin; glucose/ec [Endogenous Compound]; linezolid/dt [Drug Therapy]; meropenem/dt [Drug Therapy]; protein/ec [Endogenous Compound]; RNA 16S/ec [Endogenous Compound]; vancomycin/dt [Drug Therapy]; Gordonia sputi.

Candidate Terms

Gordonia sputi [other term].

Drug Index Terms

ampicillin / drug therapy; ceftriaxone / drug therapy; ciprofloxacin; gentamicin; glucose / endogenous compound; linezolid / drug therapy; meropenem / drug therapy; protein / endogenous compound; RNA 16S / endogenous compound; vancomycin / drug therapy.

Other Index Terms

abdominal abscess; bacterial endocarditis; *bacterial meningitis / *complication / *diagnosis / *drug therapy / *etiology; bacterial meningitis / drug therapy; bacterium identification; brain ventricle peritoneum shunt; case report; cerebrospinal fluid; *cerebrospinal fluid shunting; cognitive defect; diagnostic error; drug substitution; fever; Gordonia; granulocyte; human; limb weakness; malaise;

matrix assisted laser desorption ionization time of flight mass spectrometry; nuclear magnetic resonance imaging; Review; transthoracic echocardiography; urinary tract infection; urine incontinence.

CAS Registry Numbers

69-52-3 (ampicillin); 69-53-4 (ampicillin); 7177-48-2 (ampicillin); 74083-13-9 (ampicillin); 94586-58-0 (ampicillin); 73384-59-5 (ceftriaxone); 74578-69-1 (ceftriaxone); 85721-33-1 (ciprofloxacin); 1392-48-9 (gentamicin); 1403-66-3 (gentamicin); 1405-41-0 (gentamicin); 50-99-7 (glucose); 84778-64-3 (glucose); 165800-03-3 (linezolid); 96036-03-2 (meropenem); 67254-75-5 (protein); 1404-90-6 (vancomycin); 1404-93-9 (vancomycin)

Year of Publication

2017

Cite

40.

Gordonia sterile wound **infection** treated with ceftaroline: Case report and literature review.

Akrami K., Coletta J., Mehta S., Fierer J.

Embase
JMM Case Reports. 4(9) (no pagination), 2017. Article Number: 005113. Date of Publication: September 2017.

[Review]

AN:
618988301

Introduction. Case reports have emerged with identification of **Gordonia** bronchialis infections including sternal wound infections and foreign bodies such as central lines and shunts. Case presentation. We present a case that demonstrates the need to consider **Gordonia infection** as a cause of sternal wound **infection** and highlights the utility of novel diagnostics to aid in the identification of unusual pathogens that can cause postoperative infections. We report here the first successful use of ceftaroline for treatment of a G. bronchialis sternal wound **infection**. Conclusion. There are only case reports and in vitro assays to date to guide treatment of this **infection**, and we now add ceftaroline as a new drug to consider, though adequate surgical debridement is paramount.
Copyright © 2017 The Authors.

Status

Embase

Institution

(Akrami, Mehta, Fierer) Department of Medicine, Division of Infectious Disease, University of California, San Diego, 9500 Gilman Drive # 0711, La Jolla, CA 92093-0711, United States (Coletta) Department of Cardiothoracic Surgery, Veterans Affairs, 3350 La Jolla Village Dr, San Diego, CA 92161, United States
(Coletta) Department of Cardiothoracic Surgery Sulpizio Cardiovascular Center, University of California, San Diego, 9434 Medical Center Drive, La Jolla, CA 92037, United States
(Mehta, Fierer) Department of Medicine, Division of Infectious Disease, Veterans Affairs, 3350 La Jolla Village Dr, San Diego, CA 92161, United States

Publisher

Microbiology Society

Keyword Heading

Cardiac surgery, Ceftaroline, Gordonia bronchialis, Sternal wound infection

Emtree Heading

*Actinomycetales infection/dt [Drug Therapy]; Actinomycetales infection/dt [Drug Therapy]; aged; antibiotic sensitivity; bacterium identification; cardiopulmonary bypass; case report; cephalic vein; clinical article; computer assisted tomography; coronary artery bypass graft; debridement; device removal; disk diffusion; epsilometer test; erythema; follow up;

Gordonia
; granulation tissue;
human

; incision; male; manubrium; mass spectrometry; methicillin-resistant Staphylococcus epidermidis; minimum inhibitory concentration; osteomyelitis; polymerase chain reaction; postoperative infection/co [Complication]; postoperative infection/di [Diagnosis]; postoperative infection/dt [Drug Therapy]; review; Sanger sequencing; saphenous vein graft; sternal wound infection/dt [Drug Therapy]; sternotomy; surgical drainage; *surgical infection/co [Complication]; *surgical infection/di [Diagnosis]; *surgical infection/dt [Drug Therapy]; surgical infection/dt [Drug Therapy]; thorax pain; treatment outcome; wound dehiscence; wound healing; C reactive protein/ec [Endogenous Compound]; catalase; *ceftaroline/dt [Drug Therapy]; contrast medium/iv [Intravenous Drug Administration]; erythromycin; gentamicin; levofloxacin; linezolid; minocycline; penicillin derivative; tetracycline; vancomycin/iv [Intravenous Drug Administration]; surgical wire; Gordonia bronchialis; *sternal wound infection/co [Complication]; *sternal wound infection/di [Diagnosis]; *sternal wound infection/dt [Drug Therapy].

Candidate Terms

Gordonia bronchialis [other term]; *sternal wound infection / *complication / *diagnosis / *drug therapy [other term].

Device Index Terms

surgical wire.

Drug Index Terms

C reactive protein / endogenous compound; catalase; *ceftaroline / *drug therapy; contrast medium / intravenous drug administration; erythromycin; gentamicin; levofloxacin; linezolid; minocycline; penicillin derivative; tetracycline; vancomycin / intravenous drug administration.

Other Index Terms

*Actinomycetales infection / *drug therapy; Actinomycetales infection / drug therapy; aged; antibiotic sensitivity; bacterium identification; cardiopulmonary bypass; case report; cephalic vein; clinical article; computer assisted tomography; coronary artery bypass graft; debridement; device removal; disk diffusion; epsilometer test; erythema; follow up; Gordonia; granulation tissue; human; incision; male; manubrium; mass spectrometry; methicillin-resistant Staphylococcus epidermidis; minimum inhibitory concentration; osteomyelitis; polymerase chain reaction; postoperative infection / complication / diagnosis / drug therapy; Review; Sanger sequencing; saphenous vein graft; sternal wound infection / drug therapy; sternotomy; surgical drainage; *surgical infection / *complication / *diagnosis / *drug therapy; surgical infection / drug therapy; thorax pain; treatment outcome; wound dehiscence; wound healing.

CAS Registry Numbers

9007-41-4 (C reactive protein); 9001-05-2 (catalase); 189345-04-8 (ceftaroline); 114-07-8 (erythromycin); 70536-18-4 (erythromycin); 1392-48-9 (gentamicin); 1403-66-3 (gentamicin); 1405-41-0 (gentamicin); 100986-85-4 (levofloxacin); 138199-71-0 (levofloxacin); 165800-03-3 (linezolid); 10118-90-8 (minocycline); 11006-27-2 (minocycline); 13614-98-7 (minocycline); 23843-90-5 (tetracycline); 60-54-8 (tetracycline); 64-75-5 (tetracycline); 8021-86-1 (tetracycline); 1404-90-6 (vancomycin); 1404-93-9 (vancomycin)

Year of Publication

2017

Cite

41.

Bacteremia due to **Gordonia** polyisoprenivorans: Case report and review of literature.

Ding X., Yu Y., Chen M., Wang C., Kang Y., Li H., Lou J.

Embase

BMC Infectious Diseases. 17(1) (no pagination), 2017. Article Number: 419. Date of Publication: 12 Jun 2017.

[Article]

AN:
616716067

Background: **Gordonia** polyisoprenivorans is a ubiquitous aerobic actinomycetes bacterium that rarely cause infections in humans. Here, we report a case of G. polyisoprenivorans catheter-related bacteremia in an AIDS patient. Case presentation: A 37-year-old man with a past medical history of AIDS-related lymphoma suffered bacteremia caused by a Gram-positive corynebacterium. The strain was identified as a **Gordonia** species by matrix-assisted laser desorption ionization-time of flight mass spectrometry and confirmed to G. polyisoprenivorans by 16S rRNA combined with gyrB gene sequencing analyses. The patient was treated with imipenem and had a good outcome.
Conclusion(s): The findings from our case and previously reported cases indicate that malignant hematologic disease, immunosuppression, and indwelling catheter heighten the risk for G. polyisoprenivorans **infection**. Molecular methods

should be employed for proper identification of G. polyisoprenivorans to the species level.
Copyright © 2017 The Author(s).

PMID

28606064 [https://www.ncbi.nlm.nih.gov/pubmed/?term=28606064]

Status

Embase

Institution

(Ding, Yu, Chen, Wang, Kang, Li, Lou) Capital Medical University, Department of Clinical Laboratory, Beijing Youan Hospital, Beijing 100069, China

Publisher

BioMed Central Ltd. (E-mail: info@biomedcentral.com)

Keyword Heading

AIDS, Bacteremia, Case report, Gordonia polyisoprenivorans

Emtree Heading

adult; antibiotic sensitivity; article; *bacteremia/di [Diagnosis]; *bacteremia/dt [Drug Therapy]; bacteremia/dt [Drug Therapy]; bacterial strain; bacterium identification; case report; clinical feature; drug effect; gene sequence; *Gordonia;
human
; male; matrix assisted laser desorption ionization time of flight mass spectrometry; nonhuman; positron emission tomography-computed tomography; sequence analysis; amikacin; amoxicillin plus clavulanic acid; ampicillin; catalase/ec [Endogenous Compound]; cefotaxime; ciprofloxacin; cotrifamole; DNA topoisomerase (ATP hydrolysing) B/ec [Endogenous Compound]; imipenem/dt [Drug Therapy]; linezolid; meropenem; minocycline; RNA 16S/ec [Endogenous Compound]; vancomycin; *Gordonia polyisoprenivorans; gyrB gene.

Candidate Terms

*Gordonia polyisoprenivorans [other term]; gyrB gene [other term].

Drug Index Terms

amikacin; amoxicillin plus clavulanic acid; ampicillin; catalase / endogenous compound; cefotaxime; ciprofloxacin; cotrifamole; DNA topoisomerase (ATP hydrolysing) B / endogenous compound; imipenem / drug therapy; linezolid; meropenem; minocycline; RNA 16S / endogenous compound; vancomycin.

Other Index Terms

adult; antibiotic sensitivity; Article; *bacteremia / *diagnosis / *drug therapy; bacteremia / drug therapy; bacterial strain; bacterium identification; case report; clinical feature; drug effect; gene sequence; *Gordonia; human; male; matrix assisted laser desorption ionization time of flight mass spectrometry; nonhuman; positron emission tomography-computed tomography; sequence analysis.

CAS Registry Numbers

37517-28-5 (amikacin); 39831-55-5 (amikacin); 74469-00-4 (amoxicillin plus clavulanic acid); 79198-29-1 (amoxicillin plus clavulanic acid); 69-52-3 (ampicillin); 69-53-4 (ampicillin); 7177-48-2 (ampicillin); 74083-13-9 (ampicillin); 94586-58-0 (ampicillin); 9001-05-2 (catalase); 63527-52-6 (cefotaxime); 64485-93-4 (cefotaxime); 85721-33-1 (ciprofloxacin); 57197-43-0 (cotrifamole); 64221-86-9 (imipenem); 165800-03-3 (linezolid); 96036-03-2 (meropenem); 10118-90-8 (minocycline); 11006-27-2 (minocycline); 13614-98-7 (minocycline); 1404-90-6 (vancomycin); 1404-93-9 (vancomycin)

Year of Publication

2017

Cite

42.
A Chinese patient with peritoneal dialysis-related peritonitis caused by **Gordonia** terrae: A case report.

Hou C., Yang Y., Li Z.

Embase
BMC Infectious Diseases. 17(1) (no pagination), 2017. Article Number: 179. Date of Publication: 28 Feb 2017.

[Article]

AN:
614582837

Background: **Gordonia** terrae is a rare cause of clinical infections, with only 23 reported cases. We report the first case of peritoneal dialysis-related peritonitis caused by **Gordonia** terrae in mainland China. Case presentation: A 52-year-old man developed peritoneal dialysis-related peritonitis and received preliminary antibiotic treatment. After claiming that his symptoms had been resolved, the patient insisted on being discharged (despite our recommendations) and did not receive continued treatment after leaving the hospital. A telephone follow-up with the patient's relatives revealed that the patient died 3 months later. Routine testing did not identify the bacterial strain responsible for the **infection**, although matrix-assisted laser desorption/ionization time-of-flight mass spectrometry identified the strain as **Gordonia** rubropertincta. However, a 16S rRNA sequence analysis using an isolate from the peritoneal fluid culture revealed that the responsible strain was actually **Gordonia** terrae. Similar to this case, all previously reported cases have involved a delayed diagnosis and initial treatment failure, and the definitive diagnosis required a 16S rRNA sequence analysis. Changes from an inappropriate antibiotic therapy to an appropriate one have relied on microbiological testing and were performed 7-32 days after the initial treatment.
Conclusion(s): The findings from our case and the previously reported cases indicate that peritoneal dialysis-related peritonitis caused by **Gordonia** terrae can be difficult to identify and treat. It may be especially challenging to diagnose these cases in countries with limited diagnostic resources.
Copyright © 2017 The Author(s).

PMID

28245799 [https://www.ncbi.nlm.nih.gov/pubmed/?term=28245799]

Status

Embase

Institution

(Hou, Yang, Li) The Shanxi Dayi Hospital, Department of Clinical Laboratory, 99 Longcheng Road, Taiyuan, Shanxi 030032, China

Publisher

BioMed Central Ltd. (E-mail: info@biomedcentral.com)

Keyword Heading

Case report, Gordonia terrae, Peritoneal dialysis, Peritonitis

Emtree Heading

adult; antibiotic resistance; article; *bacterial peritonitis/di [Diagnosis]; *bacterial peritonitis/dt [Drug Therapy]; bacterial peritonitis/dt [Drug Therapy]; bacterial strain; bacterium culture; bacterium identification; case report; Chinese; female; follow up;
Gordonia
; hospital discharge;
human
; inappropriate prescribing; matrix assisted laser desorption ionization time of flight mass spectrometry; middle aged; nonhuman; *peritoneal dialysis; peritoneal fluid; RNA sequence; strain identification; symptom; telephone; treatment failure; amikacin; ampicillin; cefazolin/dt [Drug Therapy]; cefazolin/iv [Intravenous Drug Administration]; ceftriaxone; erythromycin; gentamicin; imipenem; penicillin derivative; RNA 16S/ec [Endogenous Compound]; vancomycin/dt [Drug Therapy]; Gordonia terrae.

Candidate Terms

Gordonia terrae [other term].

Drug Index Terms

amikacin; ampicillin; cefazolin / drug therapy / intravenous drug administration; ceftriaxone; erythromycin; gentamicin; imipenem; penicillin derivative; RNA 16S / endogenous compound; vancomycin / drug therapy.

Other Index Terms

adult; antibiotic resistance; Article; *bacterial peritonitis / *diagnosis / *drug therapy; bacterial peritonitis / drug therapy; bacterial strain; bacterium culture; bacterium identification; case report; Chinese; female; follow up; Gordonia; hospital discharge; human; inappropriate prescribing; matrix assisted laser desorption ionization time of flight mass spectrometry; middle aged; nonhuman; *peritoneal dialysis; peritoneal fluid; RNA sequence; strain identification; symptom; telephone; treatment failure.

CAS Registry Numbers

37517-28-5 (amikacin); 39831-55-5 (amikacin); 69-52-3 (ampicillin); 69-53-4 (ampicillin); 7177-48-2 (ampicillin); 74083-13-9 (ampicillin); 94586-58-0 (ampicillin); 25953-19-9 (cefazolin); 27164-46-1 (cefazolin); 73384-59-5 (ceftriaxone); 74578-69-1 (ceftriaxone); 114-07-8 (erythromycin); 70536-18-4 (erythromycin); 1392-48-9 (gentamicin); 1403-66-3 (gentamicin); 1405-41-0 (gentamicin); 64221-86-9 (imipenem); 1404-90-6 (vancomycin); 1404-93-9 (vancomycin)

Year of Publication

2017

Cite

43.
A novel cause of community-acquired pneumonia in a young immunocompetent host.

James N., Gilman M., Duncan R., Gray A.

Embase
Clinical Respiratory Journal. 10(5) (pp 555-558), 2016. Date of Publication: 01 Sep 2016.

[Article]

AN:
612049029

Diffuse pulmonary infiltrates represent a common problem encountered by pulmonologists. The differential diagnosis is extensive and includes infectious, inflammatory, environmental and malignant conditions. Appropriate evaluation, aside from a thorough history and physical examination, includes serologic, radiographic and procedural elements. We describe a case of a healthy male with diffuse pulmonary infiltrates. Work up revealed a novel infectious etiology. Although this particular microorganism has been described to cause native valve endocarditis, recurrent breast abscesses, osteomyelitis and bacteremia, it has to date not been described as a cause for community acquired pneumonia in immunocompetent hosts.
Copyright © 2014 John Wiley & Sons Ltd

PMID

25524175 [https://www.ncbi.nlm.nih.gov/pubmed/?term=25524175]

Status

Embase

Institution

(James, Gilman, Gray) Department of Pulmonary and Critical Care Medicine, Lahey Hospital and Medical Center, Burlington, MA, United States (Duncan) Department of Infectious Diseases, Lahey Hospital and Medical Center, Burlington, MA, United States

Publisher

Blackwell Publishing Ltd (E-mail: customerservices@oxonblackwellpublishing.com)

Keyword Heading

bacterial infection, diffuse parenchymal lung diseases, imaging/CT MRI etc, pneumonia

Emtree Heading

adult; article; case report; chill; *community acquired pneumonia/di [Diagnosis]; computer assisted tomography; coughing/dt [Drug Therapy]; crackle; diarrhea; digital clubbing; fever/dt [Drug Therapy];
Gordonia
;
human
; *immunocompetence; lung auscultation; lung infiltrate; lung lavage; male; nausea; priority journal; RNA sequence; skin test; sputum; tuberculosis/dt [Drug Therapy]; vomiting; isoniazid/dt [Drug Therapy]; levofloxacin/dt [Drug Therapy]; levofloxacin/iv [Intravenous Drug Administration]; RNA 16S/ec [Endogenous Compound]; Gordonia bronchialis.

Candidate Terms

Gordonia bronchialis [other term].

Drug Index Terms

isoniazid / drug therapy; levofloxacin / drug therapy / intravenous drug administration; RNA 16S / endogenous compound.

Other Index Terms

adult; Article; case report; chill; *community acquired pneumonia / *diagnosis; computer assisted tomography; coughing / drug therapy; crackle; diarrhea; digital clubbing; fever / drug therapy; Gordonia; human; *immunocompetence; lung auscultation; lung infiltrate; lung lavage; male; nausea; priority journal; RNA sequence; skin test; sputum; tuberculosis / drug therapy; vomiting.

CAS Registry Numbers

54-85-3 (isoniazid); 62229-51-0 (isoniazid); 65979-32-0 (isoniazid); 100986-85-4 (levofloxacin); 138199-71-0 (levofloxacin)

Year of Publication

2016

Cite

44.
Dialysis catheter related bacteriemia by **Gordonia** rubropertincta and Sputi in two hemodialysis patients.
Bacteriemia relacionada por Gordonia rubropertincta y Sputi en 2 pacientes en hemodialisis

<Bacteriemia relacionada por Gordonia rubropertincta y Sputi en 2 pacientes en hemodialisis.>

Villanueva L.S., Ortega E., Quiroga B.

Embase
Nefrologia. 36(6) (pp 715-716), 2016. Date of Publication: November - December 2016.

[Letter]

AN:
2000582372

Status

Embase

Institution

(Villanueva, Ortega, Quiroga) Hospital la Princesa, Servicio de Nefrologia, Madrid, Spain

Publisher

Elsevier Espana S.L.

Emtree Heading

aged; bacteremia/dt [Drug Therapy]; blood culture; body weight loss; case report; *catheter infection/co [Complication]; *catheter infection/dt [Drug Therapy]; catheter infection/dt [Drug Therapy]; catheter removal; clinical article; end stage renal disease/th [Therapy]; fever; *Gordonia; *Gordonia rubropertincta; *hemodialysis;
human
; letter; leukocytosis; male; very elderly; weakness; ciprofloxacin/dt [Drug Therapy]; daptomycin/dt [Drug Therapy]; meropenem/dt [Drug Therapy]; vancomycin/dt [Drug Therapy]; *dialysis catheter/am [Adverse Device Effect]; *Gordonia sputi.

Candidate Terms

*Gordonia sputi [other term].

Device Index Terms

*dialysis catheter / *adverse device effect.

Drug Index Terms

ciprofloxacin / drug therapy; daptomycin / drug therapy; meropenem / drug therapy; vancomycin / drug therapy.

Other Index Terms

aged; bacteremia / drug therapy; blood culture; body weight loss; case report; *catheter infection / *complication / *drug therapy; catheter infection / drug therapy; catheter removal; clinical article; end stage renal disease / therapy; fever; *Gordonia; *Gordonia rubropertincta; *hemodialysis; human; Letter; leukocytosis; male; very elderly; weakness.

CAS Registry Numbers

85721-33-1 (ciprofloxacin); 103060-53-3 (daptomycin); 96036-03-2 (meropenem); 1404-90-6 (vancomycin); 1404-93-9 (vancomycin)

Year of Publication

2016

Cite

45.

Catheter-related bacteremia due to **Gordonia** sputi in a patient with acute lymphocytic leukemia: A case report.

Negishi T., Matsumoto T., Saito S., Kasuga E., Horiuchi K., Natori T., Takehara K., Sugano M., Honda T.

Embase
Japanese Journal of Infectious Diseases. 69(4) (pp 342-343), 2016. Date of Publication: 2016.

[Article]

AN:
611371573

PMID

26902218 [https://www.ncbi.nlm.nih.gov/pubmed/?term=26902218]

Status

Embase

Institution

(Negishi, Matsumoto, Kasuga, Horiuchi, Natori, Takehara, Sugano, Honda) Department of Laboratory Medicine, Shinshu University Hospital, Matsumoto 390-8621, Japan (Saito) Department of Pediatrics, Shinshu University Hospital, Matsumoto 390-8621, Japan (Matsumoto) Department of Laboratory Sciences, Gunma University Graduate School of Health Sciences, 3-39-22 Showa-Machi, Maebashi, Gunma 371-8514, Japan

Publisher

National Institute of Health (E-mail: jjid@nih.go.jp)

Keyword Heading

Catheter-related bacteremia, Gordonia sputi, GyrB gene

Emtree Heading

*acute lymphoblastic leukemia; adult; antibiotic resistance; article; bacterial colonization; bacterial gene; bacterium identification; blood sampling; bone marrow suppression; case report; *catheter infection/co [Complication]; *catheter infection/di [Diagnosis]; *catheter infection/dt [Drug Therapy]; *catheter infection/ep [Epidemiology]; catheter infection/dt [Drug Therapy]; catheter related bacteremia/dt [Drug Therapy]; central venous catheter; device removal; drug withdrawal; fever; gene sequence; genetic similarity;
Gordonia
;
human
; leukocyte count; male; medical device complication/co [Complication]; minimum inhibitory concentration; pneumonia; sequence alignment; young adult; amikacin; amoxicillin plus clavulanic acid; C reactive protein/ec [Endogenous Compound]; cefepime; cefotaxime; cefozopran/dt [Drug Therapy]; ceftriaxone; ciprofloxacin; cotrimoxazole; imipenem; linezolid; minocycline; RNA 16S; tobramycin; *catheter related bacteremia/co [Complication]; *catheter related bacteremia/di [Diagnosis]; *catheter related bacteremia/dt [Drug Therapy]; *catheter related bacteremia/ep [Epidemiology]; gyrB gene.

Candidate Terms

*catheter related bacteremia / *complication / *diagnosis / *drug therapy / *epidemiology [other term]; gyrB gene [other term].

Drug Index Terms

amikacin; amoxicillin plus clavulanic acid; C reactive protein / endogenous compound; cefepime; cefotaxime; cefozopran / drug therapy; ceftriaxone; ciprofloxacin; cotrimoxazole; imipenem; linezolid; minocycline; RNA 16S; tobramycin.

Other Index Terms

*acute lymphoblastic leukemia; adult; antibiotic resistance; Article; bacterial colonization; bacterial gene; bacterium identification; blood sampling; bone marrow suppression; case report; *catheter infection / *complication / *diagnosis / *drug therapy / *epidemiology; catheter infection / drug therapy; catheter related bacteremia / drug therapy; central venous catheter; device removal; drug withdrawal; fever; gene sequence; genetic similarity; Gordonia; human; leukocyte count; male; medical device complication / complication; minimum inhibitory concentration; pneumonia; sequence alignment; young adult.

CAS Registry Numbers

37517-28-5 (amikacin); 39831-55-5 (amikacin); 74469-00-4 (amoxicillin plus clavulanic acid); 79198-29-1 (amoxicillin plus clavulanic acid); 9007-41-4 (C reactive protein); 88040-23-7 (cefepime); 63527-52-6 (cefotaxime); 64485-93-4 (cefotaxime); 113359-04-9 (cefozopran); 73384-59-5 (ceftriaxone); 74578-69-1 (ceftriaxone); 85721-33-1 (ciprofloxacin); 8064-90-2 (cotrimoxazole); 64221-86-9 (imipenem); 165800-03-3 (linezolid); 10118-90-8 (minocycline); 11006-27-2 (minocycline); 13614-98-7 (minocycline); 32986-56-4 (tobramycin)

Year of Publication

2016

Cite

46.

Identification of species of nontuberculous mycobacteria clinical isolates from 8 provinces of China.

Liu H., Lian L., Jiang Y., Huang M., Tan Y., Zhao X., Zhang J., Yu Q., Liu J., Dong H., Lu B., Wu Y., Wan K.

Embase
BioMed Research International. 2016 (no pagination), 2016. Article Number: 2153910. Date of Publication: 2016.

[Article]

AN:
613354925

Pulmonary diseases caused by nontuberculous mycobacteria (NTM) are increasing in incidence and prevalence worldwide. In this study, we identified NTM species of the clinical isolates from 8 provinces in China, in order to preliminarily provide some basic scientific data in the different species and distribution of NTM related to pulmonary disease in China. A total of 523 clinical isolates from patients with tuberculosis (TB) diagnosed clinically from 2005 to 2012 were identified to the species using conventional and molecular methods, including multilocus PCR, rpoB and hsp65 PCR-PRA, hsp65, rpoB, and 16S-23S internal transcribed spacer region sequencing. The isolates were identified into 3 bacterium genera, including NTM, **Gordonia** bronchialis, and Nocardia farcinica, and, for the 488 NTM isolates, 27 species were identified. For all the 27 species of NTM which were found to cause pulmonary infections in humans, the most prevalent species was M. intracellulare, followed by M. avium and M. abscessus. And seven other species were for the first time identified in patients with TB in China. NTM species identification is very important for distinguishing between tuberculosis and NTM pulmonary diseases, and the species diversity drives the creation of diverse and integrated identification methods with higher accuracy and efficacy.
Copyright © 2016 Haican Liu et al.

PMID

27882322 [https://www.ncbi.nlm.nih.gov/pubmed/?term=27882322]

Status

Embase

Author NameID

Liu, Haican; ORCID: https://orcid.org/0000-0002-9420-1211 Zhao, Xiuqin; ORCID: https://orcid.org/0000-0003-0084-8119
Wan, Kanglin; ORCID: https://orcid.org/0000-0002-3997-0237
Wu, Yimou; ORCID: https://orcid.org/0000-0001-6883-1677

Institution

(Liu, Lian, Jiang, Zhao, Zhang, Yu, Liu, Dong, Lu, Wan) State Key Laboratory for Infectious Disease Prevention and Control, Collaborative Innovation Center for Diagnosis and Treatment of Infectious Diseases, National Institute for Communicable Disease

Control and Prevention, Chinese Center for Disease Control and Prevention, Beijing 102206, China (Lian, Yu, Wu, Wan) Pathogenic Biology Institute, University of South China, Hengyang, Hunan 421001, China
(Huang) Fuzhou Pulmonary Hospital (Clinical Teaching Hospital of Fujian Medical University), Fuzhou, Fujian 350008, China
(Tan) Hunan Institute for Tuberculosis Control, Hunan Chest Hospital, Changsha, Hunan 410013, China

Publisher

Hindawi Publishing Corporation (410 Park Avenue, 15th Floor, 287 pmb, New York NY 10022, United States)

Emtree Heading

article; *atypical mycobacteriosis/ep [Epidemiology]; *atypical Mycobacterium; *bacterium identification; bacterium isolation; China; gene sequence;
Gordonia
;
human
; *lung infection/ep [Epidemiology]; microbial diversity; Mycobacterium abscessus; Mycobacterium avium; Mycobacterium chelonae; Mycobacterium fortuitum; Mycobacterium gordonae; Mycobacterium intracellulare; Mycobacterium massiliense; Mycobacterium szulgai; Nocardia farcinica; nonhuman; nucleotide sequence; species distribution; heat shock protein 65; internal transcribed spacer; RNA polymerase beta subunit; Gordonia bronchialis; Mycobacterium holsaticum; Mycobacterium monacense; Mycobacterium neoaurum; Mycobacterium seoulense; Mycobacterium shimoidei.

Candidate Terms

Gordonia bronchialis [other term]; Mycobacterium holsaticum [other term]; Mycobacterium monacense [other term]; Mycobacterium neoaurum [other term]; Mycobacterium seoulense [other term]; Mycobacterium shimoidei [other term].

Drug Index Terms

heat shock protein 65; internal transcribed spacer; RNA polymerase beta subunit.

Other Index Terms

Article; *atypical mycobacteriosis / *epidemiology; *atypical Mycobacterium; *bacterium identification; bacterium isolation; China; gene sequence; Gordonia; human; *lung infection / *epidemiology; microbial diversity; Mycobacterium abscessus; Mycobacterium avium; Mycobacterium chelonae; Mycobacterium fortuitum; Mycobacterium gordonae; Mycobacterium intracellulare; Mycobacterium massiliense; Mycobacterium szulgai; Nocardia farcinica; nonhuman; nucleotide sequence; species distribution.

Year of Publication

2016

Cite

47.
Fighting Off Wound Pathogens in Horses with Honeybee Lactic Acid Bacteria.

Olofsson T.C., Butler E., Lindholm C., Nilson B., Michanek P., Vasquez A.

Embase
Current Microbiology. 73(4) (pp 463-473), 2016. Date of Publication: 01 Oct 2016.

[Article]

AN:
610906093

In the global perspective of antibiotic resistance, it is urgent to find potent topical antibiotics for the use in **human** and animal **infection**. Healing of equine wounds, particularly in the limbs, is difficult due to hydrostatic factors and exposure to environmental contaminants, which can lead to heavy bio-burden/biofilm formation and sometimes to **infection**. Therefore, antibiotics are often prescribed. Recent studies have shown that honeybee-specific lactic acid bacteria (LAB), involved in honey production, and inhibit **human** wound pathogens. The aim of this pilot study was to investigate the effects on the healing of hard-to-heal equine wounds after treatment with these LAB symbionts viable in a heather honey formulation. For this, we included ten horses with wound duration of >1 year, investigated the wound microbiota, and treated wounds with the novel honeybee LAB formulation. We identified the microbiota using MALDI-TOF mass spectrometry and DNA sequencing. In addition, the antimicrobial properties of the honeybee LAB formulation were tested against all wound isolates in vitro. Our results indicate a diverse wound microbiota including fifty-three bacterial species that showed 90 % colonization by at least one species of Staphylococcus. Treatment with the formulation promoted wound healing in all cases already after the first application and the wounds were either completely healed (n = 3) in less than 20 days or healing was in progress. Furthermore, the honeybee LAB formulation inhibited all pathogens when tested

in vitro. Consequently, this new treatment option presents as a powerful candidate for the topical treatment of hard-to-heal wounds in horses.
Copyright © 2016, The Author(s).

PMID

27324340 [https://www.ncbi.nlm.nih.gov/pubmed/?term=27324340]

Status

Embase

Institution

(Olofsson, Butler, Vasquez) Laboratory Medicine, Lunds Universitet, Lund, Sweden (Lindholm) Division of Nursing Science, Sophiahemmet Hogskola, Stockholm, Sweden
(Nilson) Laboratory Medicine, Clinical Microbiology, Region Skane, Lund, Sweden
(Nilson) Department of Laboratory Medicine Lund, Medical Microbiology, Lund University, Solvegatan 23, Lund 22362, Sweden
(Michanek) Animal Farm Veterinary Consultants, Degebergavagen, Vollsjo 27568, Sweden

Publisher

Springer New York LLC (E-mail: barbara.b.bertram@gsk.com)

Emtree Heading

Acinetobacter; Acinetobacter lwoffii; Actinomycetales; Aerococcus viridans; Alcaligenes faecalis; antibacterial activity; *antibiotic therapy; Arthrobacter; article; Bacillus cereus; Bacillus mycoides; Bacillus pumilus; Bacillus subtilis; *bacterium identification; Bacteroides; Brevibacterium; Candida; Candida parapsilosis; Carnobacterium; Citrobacter; Clostridium; controlled clinical trial; controlled study; Corynebacterium amycolatum; Corynebacterium diphtheriae; Corynebacterium glutamicum; DNA sequence; Enterobacter; Enterobacter aerogenes; Enterococcus faecalis; Enterococcus faecium; epithelization; gene sequence;
Gordonia
; *honeybee; *horse disease/dt [Drug Therapy]; *horse disease/su [Surgery]; horse disease/dt [Drug Therapy]; Klebsiella oxytoca; matrix assisted laser desorption ionization time of flight mass spectrometry; Micrococcus; nonhuman; Pantoea agglomerans; Pasteurella; pilot study; priority journal; Proteus vulgaris; Psychrobacter; Staphylococcus; Staphylococcus aureus; Staphylococcus epidermidis; Staphylococcus hyicus; Staphylococcus lugdunensis; Staphylococcus pseudintermedius; Staphylococcus schleiferi; Staphylococcus sciuri; Staphylococcus xylosus; Streptococcus; Streptococcus dysgalactiae; Streptococcus equi; Streptococcus equinus; Streptomyces; wound closure; wound healing; *wound infection/dt [Drug Therapy]; *wound infection/su [Surgery]; wound infection/dt [Drug Therapy]; bacterial RNA; *natural product/ct [Clinical Trial]; *natural product/dt [Drug Therapy]; *natural product/pd [Pharmacology]; RNA 16S; Acinetobacter towneri; Aeromonas bestiarum; Aeromonas encheliea; Arthrobacter arilaitensis; Arthrobacter castelli; Arthrobacter gandavensis; Bacteroides pyogenes; Brachybacterium faecium; Brevibacterium conglomeratum; Citrobacter braakii; clostridium absonum; Corynebacterium casei; Enterobacter ludwigii; Gordonia hirsuta; Macrococcus; Pasteurella canis; Peptonophilus indolicus; Psychrobacter sanguinis; Staphylococcus chromogenes; staphylococcus delphini; Staphylococcus equorum; Staphylococcus vitulinus; Streptococcus parauberis; Streptomyces badius.

Candidate Terms

Acinetobacter towneri [other term]; Aeromonas bestiarum [other term]; Aeromonas encheliea [other term]; Arthrobacter arilaitensis [other term]; Arthrobacter castelli [other term]; Arthrobacter gandavensis [other term]; Bacteroides pyogenes [other term]; Brachybacterium faecium [other term]; Brevibacterium conglomeratum [other term]; Citrobacter braakii [other term]; Clostridium absonum [other term]; Corynebacterium casei [other term]; Enterobacter ludwigii [other term]; Gordonia hirsuta [other term]; Macrococcus [other term]; Pasteurella canis [other term]; Peptonophilus indolicus [other term]; Psychrobacter sanguinis [other term]; Staphylococcus chromogenes [other term]; Staphylococcus delphini [other term]; Staphylococcus equorum [other term]; Staphylococcus vitulinus [other term]; Streptococcus parauberis [other term]; Streptomyces badius [other term].

Drug Index Terms

bacterial RNA; *natural product / *clinical trial / *drug therapy / *pharmacology; RNA 16S.

Other Index Terms

Acinetobacter; Acinetobacter lwoffii; Actinomycetales; Aerococcus viridans; Alcaligenes faecalis; antibacterial activity; *antibiotic therapy; Arthrobacter; Article; Bacillus cereus; Bacillus mycoides; Bacillus pumilus; Bacillus subtilis; *bacterium identification; Bacteroides; Brevibacterium; Candida; Candida parapsilosis; Carnobacterium; Citrobacter; Clostridium; controlled clinical trial; controlled study; Corynebacterium amycolatum; Corynebacterium diphtheriae; Corynebacterium glutamicum; DNA sequence; Enterobacter; Enterobacter aerogenes; Enterococcus faecalis; Enterococcus faecium; epithelization; gene sequence; Gordonia; *honeybee; *horse disease / *drug therapy / *surgery; horse disease / drug therapy; Klebsiella oxytoca; matrix assisted laser desorption ionization time of flight mass spectrometry; Micrococcus; nonhuman; Pantoea agglomerans; Pasteurella; pilot study; priority journal; Proteus vulgaris; Psychrobacter; Staphylococcus; Staphylococcus aureus; Staphylococcus epidermidis;

Staphylococcus hyicus; Staphylococcus lugdunensis; Staphylococcus pseudintermedius; Staphylococcus schleiferi; Staphylococcus sciuri; Staphylococcus xylosus; Streptococcus; Streptococcus dysgalactiae; Streptococcus equi; Streptococcus equinus; Streptomyces; wound closure; wound healing; *wound infection / *drug therapy / *surgery; wound infection / drug therapy.

Year of Publication

2016

Cite

48.

Sternal wound **infection** caused by **Gordonia** bronchialis: Identification by MALDI-TOF MS.

Rodriguez-Lozano J., Perez-Llantada E., Aguero J., Rodriguez-Fernandez A., De Alegria C.R., Martinez-Martinez L., Calvo J.

Embase
JMM Case Reports. 3(5) (no pagination), 2016. Date of Publication: 01 Sep 2016.

[Article]

AN:
612796281

Introduction: **Gordonia** spp. infections are uncommon. However, a few clinical cases have been reported in the literature, particularly those involving immunocompromised hosts. Advanced microbiology diagnosis techniques, such as matrix-assisted laser desorption ionization-time of flight MS (MALDI-TOF MS), have been recently introduced in clinical microbiology laboratories in order to improve microbial identification, resulting in better patient management. Case presentation: Here, we present a new clinical case of persistent wound **infection** caused by **Gordonia** bronchialis in a 64-year-old woman after a mitral valve replacement, using two MALDI-TOF-based systems for identifying this micro-organism.
Conclusion(s): Both MALDI-TOF systems were able to identify **Gordonia** spp.; thus, providing a useful tool that overcomes the current limitations of phenotypic identification associated with this micro-organism. Although the technique validation deserves additional verification, our study provides guidance about MALDI-TOF as a fast and easy method for **Gordonia** spp. identification.
Copyright © 2016 The Authors.

Status

Embase

Institution

(Rodriguez-Lozano, Perez-Llantada, Aguero, Rodriguez-Fernandez, De Alegria, Martinez-Martinez, Calvo) Marques de Valdecilla University Hospital, Santander, Cantabria, Spain

Publisher

Microbiology Society

Keyword Heading

Gordonia, MALDI-TOF, Sternal wound infection

Emtree Heading

adult; article; *bacterium identification; case report; computer assisted tomography; female; *Gordonia;
human
; immunocompromised patient; *matrix assisted laser desorption ionization time of flight mass spectrometry; middle aged; mitral valve replacement; phenotype; *wound infection/co [Complication]; *wound infection/dt [Drug Therapy]; wound infection/dt [Drug Therapy]; albumin/ec [Endogenous Compound]; C reactive protein/ec [Endogenous Compound]; ceftazidime/dt [Drug Therapy]; ceftazidime/iv [Intravenous Drug Administration]; ciprofloxacin/cb [Drug Combination]; ciprofloxacin/dt [Drug Therapy]; ciprofloxacin/po [Oral Drug Administration]; clindamycin/dt [Drug Therapy]; clindamycin/iv [Intravenous Drug Administration]; imipenem/dt [Drug Therapy]; imipenem/iv [Intravenous Drug Administration]; rifampicin/dt [Drug Therapy]; rifampicin/po [Oral Drug Administration]; teicoplanin/cb [Drug Combination]; teicoplanin/dt [Drug Therapy]; teicoplanin/iv [Intravenous Drug Administration].

Drug Index Terms

albumin / endogenous compound; C reactive protein / endogenous compound; ceftazidime / drug therapy / intravenous drug

administration; ciprofloxacin / drug combination / drug therapy / oral drug administration; clindamycin / drug therapy / intravenous drug administration; imipenem / drug therapy / intravenous drug administration; rifampicin / drug therapy / oral drug administration; teicoplanin / drug combination / drug therapy / intravenous drug administration.

Other Index Terms

adult; Article; *bacterium identification; case report; computer assisted tomography; female; *Gordonia; human; immunocompromised patient; *matrix assisted laser desorption ionization time of flight mass spectrometry; middle aged; mitral valve replacement; phenotype; *wound infection / *complication / *drug therapy; wound infection / drug therapy.

CAS Registry Numbers

9007-41-4 (C reactive protein); 72558-82-8 (ceftazidime); 85721-33-1 (ciprofloxacin); 18323-44-9 (clindamycin); 64221-86-9 (imipenem); 13292-46-1 (rifampicin); 61036-62-2 (teicoplanin); 61036-64-4 (teicoplanin)

Year of Publication

2016

Cite

49.

Cutaneous abscess due to **Gordonia** bronchialis: case report and literature review.

Bartolome-Alvarez J., Saez-Nieto J.A., Escudero-Jimenez A., Barba-Rodriguez N., Galan-Ros J., Carrasco G., Munoz-Izquierdo M.P.

Embase
Revista espanola de quimioterapia : publicacion oficial de la Sociedad Espanola de Quimioterapia. 29(3) (pp 170-173), 2016. Date of Publication: 01 Jun 2016.

[Article]

AN:
615043875

PMID

27015823 [https://www.ncbi.nlm.nih.gov/pubmed/?term=27015823]

Institution

(Bartolome-Alvarez) Joaquin Bartolome-Alvarez, Servicio de Microbiologia. Hospital General Universitario de Albacete. C/ Hermanos Falco, 37; 02006 Albacete, Spain

Emtree Heading

abscess/dt [Drug Therapy]; case report; female; *Gordonia;
human
; *microbiology; middle aged; subcutaneous drug administration; amoxicillin plus clavulanic acid/dt [Drug Therapy]; antiinfective agent/dt [Drug Therapy]; Actinomycetales Infections/dt [Drug Therapy]; adverse effects; Skin Diseases, Infectious/dt [Drug Therapy].

Candidate Terms

Actinomycetales Infections / drug therapy [other term]; adverse effects [other term]; Skin Diseases, Infectious / drug therapy [other term].

Drug Index Terms

amoxicillin plus clavulanic acid / drug therapy; antiinfective agent / drug therapy.

Other Index Terms

abscess / drug therapy; case report; female; *Gordonia; human; *microbiology; middle aged; subcutaneous drug administration.

CAS Registry Numbers

74469-00-4 (amoxicillin plus clavulanic acid); 79198-29-1 (amoxicillin plus clavulanic acid)

Year of Publication

2016

Cite

50.

Reducing time to identification of positive blood cultures with MALDI-TOF MS analysis after a 5-h subculture.

Verroken A., Defourny L., Lechgar L., Magnette A., Delmee M., Glupczynski Y.

Embase

European Journal of Clinical Microbiology and Infectious Diseases. 34(2) (pp 405-413), 2015. Date of Publication: February 2014.

[Article]

AN:
600349099

Speeding up the turn-around time of positive blood culture identifications is essential in order to optimize the treatment of septic patients. Several sample preparation techniques have been developed allowing direct matrix-assisted laser desorption/ionization time-of-flight mass spectrometry (MALDI-TOF MS) identification of positive blood cultures. Yet, the hands-on time restrains their routine workflow. In this study, we evaluated an approach whereby MALDI-TOF MS identification without any additional steps was carried out on short subcultured colonies from positive blood bottles with the objective of allowing results reporting on the day of positivity detection. Over a 7-month period in 2012, positive blood cultures detected by 9 am with an automated system were inoculated onto a Columbia blood agar and processed after a 5-h incubation on a MALDI-TOF MicroFlex platform (Bruker Daltonik GmbH). Single-spotted colonies were covered with 1 mul formic acid and 1 mul matrix solution. The results were compared to the validated identification techniques. A total of 925 positive blood culture bottles (representing 470 bacteremic episodes) were included. Concordant identification was obtained in 727 (81.1 %) of the 896 monomicrobial blood cultures, with failure being mostly observed with anaerobes and yeasts. In 17 episodes of polymicrobic bacteremia, the identification of one of the two isolates was achieved in 24/29 (82.7 %) positive cultures. Routine implementation of MALDI-TOF MS identification on young positive blood subcultures provides correct results to the clinician in more than 80 % of the bacteremic episodes and allows access to identification results on the day of blood culture positivity detection, potentially accelerating the implementation of targeted clinical treatments.
Copyright © 2014, Springer-Verlag Berlin Heidelberg.

PMID

25252627 [https://www.ncbi.nlm.nih.gov/pubmed/?term=25252627]

Status

Embase

Institution

(Verroken, Delmee) Institut de recherche experimentale et clinique (IREC), pole de microbiologie (MBLG), Universite catholique de Louvain, Brussels, Belgium (Verroken, Defourny, Lechgar, Magnette, Delmee) Laboratoire de microbiologie, Cliniques universitaires Saint-Luc-Universite catholique de Louvain, Brussels, Belgium
(Glupczynski) National Reference Centre for Monitoring of Antimicrobial Resistance in Gram-negative bacteria, CHU Dinant Godinne UCL Namur, Yvoir, Belgium

Publisher

Springer Verlag

Emtree Heading

article; Bacillus cereus; bacterial growth; bacterium contamination; *blood culture; bloodstream infection; Corynebacterium; Corynebacterium jeikeium; Enterobacter aerogenes; Enterobacter cloacae; Enterobacteriaceae; Enterococcus; Enterococcus avium; Enterococcus faecalis; Enterococcus faecium; Escherichia coli; gene sequence;
Gordonia
; Granulicatella adiacens; Haemophilus influenzae; Hafnia alvei;
human
; Klebsiella oxytoca; Klebsiella pneumoniae; *matrix assisted laser desorption ionization time of flight mass spectrometry; Micrococcus luteus; Morganella morganii; Neisseria meningitidis; priority journal; Proteus mirabilis; species identification; Staphylococcus; Staphylococcus aureus; Staphylococcus capitis; Staphylococcus cohnii; Staphylococcus epidermidis; Staphylococcus haemolyticus; Staphylococcus hominis; Staphylococcus lugdunensis; Staphylococcus sciuri; Staphylococcus warneri; Streptococcus; Streptococcus agalactiae; Streptococcus anginosus; Streptococcus dysgalactiae; Streptococcus mitis; Streptococcus pneumoniae; Streptococcus pyogenes; Streptococcus salivarius; Streptococcus sanguinis.

Other Index Terms

Article; Bacillus cereus; bacterial growth; bacterium contamination; *blood culture; bloodstream infection; Corynebacterium; Corynebacterium jeikeium; Enterobacter aerogenes; Enterobacter cloacae; Enterobacteriaceae; Enterococcus; Enterococcus avium; Enterococcus faecalis; Enterococcus faecium; Escherichia coli; gene sequence; Gordonia; Granulicatella adiacens; Haemophilus influenzae; Hafnia alvei; human; Klebsiella oxytoca; Klebsiella pneumoniae; *matrix assisted laser desorption ionization time of flight mass spectrometry; Micrococcus luteus; Morganella morganii; Neisseria meningitidis; priority journal; Proteus mirabilis; species identification; Staphylococcus; Staphylococcus aureus; Staphylococcus capitis; Staphylococcus cohnii; Staphylococcus epidermidis; Staphylococcus haemolyticus; Staphylococcus hominis; Staphylococcus lugdunensis; Staphylococcus sciuri; Staphylococcus warneri; Streptococcus; Streptococcus agalactiae; Streptococcus anginosus; Streptococcus dysgalactiae; Streptococcus mitis; Streptococcus pneumoniae; Streptococcus pyogenes; Streptococcus salivarius; Streptococcus sanguinis.

Year of Publication

2015

Cite

51.

Gordonia species as emerging causes of continuous-ambulatory-peritoneal-dialysis-related peritonitis identified by 16S rRNA and secA1 gene sequencing and matrix-assisted laser desorption ionization-time of flight mass spectrometry (MALDI-TOF MS).

Lam J.Y.W., Wu A.K.L., Leung W.-S., Cheung I., Tsang C.-C., Chen J.H.K., Chan J.F.W., Tse C.W.S., Lee R.A., Lau S.K.P., Wood P.C.Y.

Embase
Journal of Clinical Microbiology. 53(2) (pp 671-676), 2015. Date of Publication: 01 Feb 2015.

[Article]

AN:
601778745

We report here four cases of continuous ambulatory peritoneal dialysis-related peritonitis caused by three different species of **Gordonia**. The portal of entry was likely through Tenckhoff catheters. 16S rRNA and secA1 gene sequencing are so far the most reliable methods for the accurate identification of **Gordonia** species.
Copyright © 2015, American Society for Microbiology. All Rights Reserved.

Status

Embase

Institution

(Lam, Wu, Lee) Department of Clinical Pathology, Pamela Youde Nethersole Eastern Hospital, Hong Kong, Hong Kong (Leung) Department of Medicine and Geriatrics, Princess Margaret Hospital, Hong Kong, Hong Kong
(Cheung, Tse) Department of Pathology, Kwong Wah Hospital, Hong Kong, Hong Kong
(Tsang, Chen, Chan, Lau) Department of Microbiology, Hong Kong, Hong Kong
(Chan, Lau, Wood) Research Centre of Infection and Immunology, Hong Kong, Hong Kong
(Chan, Lau, Wood) State Key Laboratory of Emerging Infectious Diseases, Hong Kong, Hong Kong
(Chan, Lau, Wood) Carol Yu Centre for Infection, University of Hong Kong, Hong Kong, Hong Kong

Publisher

American Society for Microbiology (E-mail: Journals@asmusa.org)

Emtree Heading

abdominal pain; abdominal tenderness; adult; aged; antibiotic resistance; article; *bacterial gene; *bacterial peritonitis/co [Complication]; *bacterial peritonitis/dt [Drug Therapy]; bacterial peritonitis/dt [Drug Therapy]; bacterial strain; case report; *catheter infection/co [Complication]; *catheter infection/dt [Drug Therapy]; catheter infection/dt [Drug Therapy]; *continuous ambulatory peritoneal dialysis; controlled study; diabetic nephropathy; DNA extraction; DNA sequence; end stage renal disease/co [Complication]; end stage renal disease/th [Therapy]; Escherichia coli; female; *gene sequence; *Gordonia;
human
; immunoglobulin A nephropathy; kidney disease; leukocyte count; male; *matrix assisted laser desorption ionization time of flight mass spectrometry; middle aged; minimum inhibitory concentration; polymerase chain reaction; Pseudomonas aeruginosa; Rhodococcus; Staphylococcus; Streptococcus pneumoniae; amikacin/dt [Drug Therapy]; cefazolin/dt [Drug Therapy]; cefazolin/ip [Intraperitoneal Drug Administration]; ceftazidime/dt [Drug Therapy]; cilastatin plus imipenem/dt [Drug Therapy]; cilastatin plus imipenem/iv [Intravenous Drug Administration]; ciprofloxacin/dt [Drug Therapy]; gentamicin/dt [Drug Therapy]; imipenem/dt [Drug

Therapy]; levofloxacin/dt [Drug Therapy]; levofloxacin/po [Oral Drug Administration]; meropenem/dt [Drug Therapy]; meropenem/iv [Intravenous Drug Administration]; *RNA 16S/ec [Endogenous Compound]; vancomycin/dt [Drug Therapy]; vancomycin/iv [Intravenous Drug Administration]; Gordonia bronchialis; Gordonia lacunae; Gordonia sputi; Gordonia terrae; hypertensive nephropathy; *secA1 gene.

Candidate Terms

Gordonia bronchialis [other term]; Gordonia lacunae [other term]; Gordonia sputi [other term]; Gordonia terrae [other term]; hypertensive nephropathy [other term]; *secA1 gene [other term].

Drug Index Terms

amikacin / drug therapy; cefazolin / drug therapy / intraperitoneal drug administration; ceftazidime / drug therapy; cilastatin plus imipenem / drug therapy / intravenous drug administration; ciprofloxacin / drug therapy; gentamicin / drug therapy; imipenem / drug therapy; levofloxacin / drug therapy / oral drug administration; meropenem / drug therapy / intravenous drug administration; *RNA 16S / *endogenous compound; vancomycin / drug therapy / intravenous drug administration.

Other Index Terms

abdominal pain; abdominal tenderness; adult; aged; antibiotic resistance; Article; *bacterial gene; *bacterial peritonitis / *complication / *drug therapy; bacterial peritonitis / drug therapy; bacterial strain; case report; *catheter infection / *complication / *drug therapy; catheter infection / drug therapy; *continuous ambulatory peritoneal dialysis; controlled study; diabetic nephropathy; DNA extraction; DNA sequence; end stage renal disease / complication / therapy; Escherichia coli; female; *gene sequence; *Gordonia; human; immunoglobulin A nephropathy; kidney disease; leukocyte count; male; *matrix assisted laser desorption ionization time of flight mass spectrometry; middle aged; minimum inhibitory concentration; polymerase chain reaction; Pseudomonas aeruginosa; Rhodococcus; Staphylococcus; Streptococcus pneumoniae.

CAS Registry Numbers

37517-28-5 (amikacin); 39831-55-5 (amikacin); 25953-19-9 (cefazolin); 27164-46-1 (cefazolin); 72558-82-8 (ceftazidime); 92309-29-0 (cilastatin plus imipenem); 85721-33-1 (ciprofloxacin); 1392-48-9 (gentamicin); 1403-66-3 (gentamicin); 1405-41-0 (gentamicin); 64221-86-9 (imipenem); 100986-85-4 (levofloxacin); 138199-71-0 (levofloxacin); 96036-03-2 (meropenem); 1404-90-6 (vancomycin); 1404-93-9 (vancomycin)

Year of Publication

2015

Cite

52.

Plastic Expander-Related **Gordonia** Sputi **Infection**: Case Report and Literature Review.

Zhang H.L., Cao B., Li J., Cheng Y., Wang X.J.

Embase
Biomedical and environmental sciences : BES. 28(6) (pp 468-471), 2015. Date of Publication: 01 Jun 2015.

[Article]

AN:
606865529

Gordonia sputi causes rare bacterial infections resulting from a contaminated indwelling medical device. We report the case of a postoperative plastic expander abscess in a woman, with G. sputi identification by 16S ribosomal RNA sequencing. This report indicates that **Gordonia** spp. should be included in the list of organisms causing plastic implant infections.
Copyright © 2015 The Editorial Board of Biomedical and Environmental Sciences. Published by China CDC. All rights reserved.

PMID

26177910 [https://www.ncbi.nlm.nih.gov/pubmed/?term=26177910]

Institution

(Zhang) Department of Plastic and Reconstructive Surgery, Peking Union Medical College Hospital, Chinese Academy of Medical Sciences, Beijing 100730, China (Cao) National Institute for Communicable Disease Control and Prevention, State Key Laboratory for

Infectious Disease Prevention and Control (SKLID), China CDC, Beijing 102206, China
(Li) National Institute for Communicable Disease Control and Prevention, State Key Laboratory for Infectious Disease Prevention and Control (SKLID), China CDC, Beijing 102206, China
(Cheng) Key Laboratory of Surveillance and Early-warning on Infectious Disease, Division of Infectious Disease, Chinese Center for Disease Control and Prevention, Beijing 102206, China
(Wang) Department of Plastic and Reconstructive Surgery, Peking Union Medical College Hospital, Chinese Academy of Medical Sciences, Beijing 100730, China

Emtree Heading

adult; aged; breast implant; breast reconstruction; case report; female;
Gordonia
;
human
; *isolation and purification; male; *medical device contamination; *microbiology; middle aged; newborn; *physiology; *plastic; Actinomycetales Infections/et [Etiology]; *adverse effects; statistics and numerical data.

Candidate Terms

Actinomycetales Infections / etiology [other term]; *adverse effects [other term]; statistics and numerical data [other term].

Drug Index Terms

*plastic.

Other Index Terms

adult; aged; breast implant; breast reconstruction; case report; female; Gordonia; human; *isolation and purification; male; *medical device contamination; *microbiology; middle aged; newborn; *physiology.

Year of Publication

2015

Cite

53.
Identification and characterization of non-tuberculous mycobacteria isolated from tuberculosis suspects in Southern-Central China.

Yu X.-L., Lu L., Chen G.-Z., Liu Z.-G., Lei H., Song Y.-Z., Zhang S.-L.

Embase
PLoS ONE. 9(12) (no pagination), 2014. Article Number: e114353. Date of Publication: 02 Dec 2014.

[Article]

AN:
600627363

The incidence of non-tuberculous mycobacteria (NTM)-related death has increased globally recently. To obtain information of the species and characterization of pathogens involved in NTM pulmonary **infection** in Southern-central China, we identified 160 non-tuberculous **infection** cases from 3995 acid-fast bacilli (AFB)-positive tuberculous suspects. We then randomly selected 101 non-tuberculous patients, isolated bacteria from their sputa and genotyped the pathogens using the 16S rRNA gene and 16S-23S rRNA internal transcribed spacer sequences. M. intracellulare (32.67%, 33/101), M. abscessus (32.67%, 33/101) and M. fortuitum (7.92%, 8/101) are identified in these isolates. Surprisingly, non-mycobacteria including **Gordonia** (8.91%, 9/101), Nocardia (5.94%, 6/101) and Tsukamurella (0.99%, 1/101) are also discovered, and the case of Tsukamurella pulmonis **infection** is first discovered in Southern-central China. Moreover, species of M. mucogenicum group, M. chubuense, M. kansasii, M. gastri, M. avium, M. porcinum and M. smegmatis are identified. In addition, nine immune compromised cases (8.91%, 9/101), including type two diabetes mellitus and HIV/AIDS are found to be infected with non-tuberculous bacteria. This study revealed the distribution and characteristics of non-tuberculous AFB pathogen **infection** occurred in Southern-central China, and suggested that physicians should be alert of the emerging of NTM and non-mycobacteria **infection** in AFB positive cases and take caution when choosing chemotherapy for tuberculosis-like pulmonary infections. Generally, this study may help with the development of new strategy for the diagnosis and treatment of mycobacterial **infection**.
Copyright © 2014 Yu et al.

PMID

25463697 [https://www.ncbi.nlm.nih.gov/pubmed/?term=25463697]

Status

Embase

Institution

(Yu, Lu, Chen, Liu, Lei) School of Biology and Pharmaceutical Engineering, Wuhan Polytechnic University, Wuhan, China (Song) Shanghai Public Health Clinical Center, Shanghai, China
(Zhang) Department of Immunology and Microbiology, Shanghai Jiao Tong University School of Medicine, Shanghai, China

Publisher

Public Library of Science (E-mail: plos@plos.org)

Emtree Heading

acid fast bacterium; acquired immune deficiency syndrome; adult; aged; article; atypical mycobacteriosis; *atypical Mycobacterium; bacterium identification; bacterium isolation; China; female; gene sequence; genotype;
Gordonia
;
human
; lung tuberculosis; major clinical study; male; middle aged; Mycobacterium; Mycobacterium abscessus; Mycobacterium fortuitum; Mycobacterium intracellulare; Mycobacterium kansasii; Mycobacterium mucogenicum; Mycobacterium smegmatis; Nocardia; non insulin dependent diabetes mellitus; phenotype; RNA gene; sputum culture; young adult; internal transcribed spacer/ec [Endogenous Compound]; RNA 16S/ec [Endogenous Compound]; RNA 23S/ec [Endogenous Compound]; Mycobacterium chubuense; mycobacterium gastri; mycobacterium porcinum; Tsukamurella.

Candidate Terms

Mycobacterium chubuense [other term]; Mycobacterium gastri [other term]; Mycobacterium porcinum [other term]; Tsukamurella [other term].

Drug Index Terms

internal transcribed spacer / endogenous compound; RNA 16S / endogenous compound; RNA 23S / endogenous compound.

Other Index Terms

acid fast bacterium; acquired immune deficiency syndrome; adult; aged; Article; atypical mycobacteriosis; *atypical Mycobacterium; bacterium identification; bacterium isolation; China; female; gene sequence; genotype; Gordonia; human; lung tuberculosis; major clinical study; male; middle aged; Mycobacterium; Mycobacterium abscessus; Mycobacterium fortuitum; Mycobacterium intracellulare; Mycobacterium kansasii; Mycobacterium mucogenicum; Mycobacterium smegmatis; Nocardia; non insulin dependent diabetes mellitus; phenotype; RNA gene; sputum culture; young adult.

Year of Publication

2014

Cite

54.
A polymicrobial outbreak of surgical site infections following cardiac surgery at a community hospital in Florida, 2011-2012.

Nguyen D.B., Gupta N., Abou-Daoud A., Klekamp B.G., Rhone C., Winston T., Hedberg T., Scuteri A., Evans C., Jensen B., Moulton-Meissner H., Torok T., Berrios-Torres S.I., Noble-Wang J., Kallen A.

Embase
American Journal of Infection Control. 42(4) (pp 432-435), 2014. Date of Publication: April 2014.

[Article]

AN:
372730958

We describe an outbreak of 22 sternal surgical site infections following cardiac surgery, including 4 **Gordonia** infections. Possible operation room environmental contamination and suboptimal **infection** control practices regarding scrub attire may have contributed to the outbreak. © 2014 by the Association for Professionals in **Infection** Control and Epidemiology, Inc. Published by Elsevier Inc. All rights reserved.

PMID

24679572 [https://www.ncbi.nlm.nih.gov/pubmed/?term=24679572]

Status

Embase

Institution

(Nguyen, Gupta, Jensen, Moulton-Meissner, Berrios-Torres, Noble-Wang, Kallen) Division of Healthcare Quality Promotion, Centers for Disease Control and Prevention, Atlanta, GA, United States (Nguyen, Gupta, Abou-Daoud) Scientific Education and Professional Development Program Office, Centers for Disease Control and Prevention, Atlanta, GA, United States
(Klekamp, Rhone, Winston, Hedberg, Scuteri, Torok) Florida Department of Health, Tallahassee, FL, United States
(Evans) Hospital Corporation of America, FL, United States

Publisher

Mosby Inc. (E-mail: customerservice@mosby.com)

Keyword Heading

Environmental contamination, Gordonia, Infection control

Emtree Heading

adult; aged; antibiotic prophylaxis; article; atmospheric pressure; bacterium contamination; bacterium culture; Candida albicans; cleaning; clinical article; community hospital; *epidemic; Escherichia coli; female;
Gordonia
; *heart surgery;
human
; infection control; male; medical staff; methicillin resistant Staphylococcus aureus; methicillin susceptible Staphylococcus aureus; nonhuman; operating room; patient care; preoperative care; Proteus mirabilis; Pseudomonas aeruginosa; Serratia marcescens; skin care; sternum; surgical attire; *surgical infection/co [Complication]; United States; wound care; cefazolin; vancomycin; Gordonia bronchialis; Gordonia terrae.

Candidate Terms

Gordonia bronchialis [other term]; Gordonia terrae [other term].

Drug Index Terms

cefazolin; vancomycin.

Other Index Terms

adult; aged; antibiotic prophylaxis; article; atmospheric pressure; bacterium contamination; bacterium culture; Candida albicans; cleaning; clinical article; community hospital; *epidemic; Escherichia coli; female; Gordonia; *heart surgery; human; infection control; male; medical staff; methicillin resistant Staphylococcus aureus; methicillin susceptible Staphylococcus aureus; nonhuman; operating room; patient care; preoperative care; Proteus mirabilis; Pseudomonas aeruginosa; Serratia marcescens; skin care; sternum; surgical attire; *surgical infection / *complication; United States; wound care.

CAS Registry Numbers

25953-19-9 (cefazolin); 27164-46-1 (cefazolin); 1404-90-6 (vancomycin); 1404-93-9 (vancomycin)

Year of Publication

2014

Cite

55.
Sternal osteomyelitis caused by **Gordonia** bronchialis after open-heart surgery.

Chang J.-H., Ji M., Hong H.-L., Choi S.-H., Kim Y.-S., Chung C.-H., Sung H., Kim M.-N.
Embase
Infection and Chemotherapy. 46(2) (pp 110-114), 2014. Date of Publication: 2014.
[Article]
AN:
373469221

We report the case of a deep sternal wound **infection** with sternal osteomyelitis caused by **Gordonia** bronchialis after open-heart surgery. The isolate was identified as a G. bronchialis by 16S rRNA and hsp65 gene sequencing, having initially been misidentified as a Rhodococcus by a commercial phenotypic identification system. © 2014 by The Korean Society of Infectious Diseases.

Status

Embase

Institution

(Chang, Ji, Sung, Kim) Department of Laboratory Medicine, Seoul, South Korea (Hong, Choi, Kim) Division of Infectious Diseases, Department of Internal Medicine, Seoul, South Korea
(Chung) Department of Thoracic Surgery, Asan Medical Center, University of Ulsan College of Medicine, Seoul, South Korea

Publisher

Korean Society for Chemotherapy (E-mail: ksinfect@naver.com)

Keyword Heading

16S rRNA gene, Gordonia bronchialis, Hsp65, Osteomyelitis, Wound infection

Emtree Heading

aged; antibiotic sensitivity; antibiotic therapy; article; atelectasis; bacterium culture; bacterium isolate; case report; cellulitis; computer assisted tomography; coronary artery bypass graft; diabetes mellitus; disease exacerbation; drug treatment failure; female; gene sequence; *Gordonia;
human
; hypertension; matrix assisted laser desorption ionization time of flight mass spectrometry; nonhuman; nucleotide sequence; *open heart surgery; *osteomyelitis/co [Complication]; *osteomyelitis/dt [Drug Therapy]; osteomyelitis/dt [Drug Therapy]; postoperative pain/co [Complication]; postoperative pain/dt [Drug Therapy]; pus; Rhodococcus; sternal osteomyelitis/dt [Drug Therapy]; sternotomy; substernal pain/co [Complication]; substernal pain/dt [Drug Therapy]; *surgical infection/co [Complication]; *surgical infection/dt [Drug Therapy]; surgical infection/dt [Drug Therapy]; wound dehiscence; wound healing; amikacin; amoxicillin plus clavulanic acid; C reactive protein/ec [Endogenous Compound]; cefepime; cefotetan/dt [Drug Therapy]; ceftriaxone; ciprofloxacin; clarithromycin; cotrimoxazole; doxycycline; imipenem/dt [Drug Therapy]; imipenem/iv [Intravenous Drug Administration]; linezolid; minocycline; moxifloxacin; opiate/dt [Drug Therapy]; penicillin G/dt [Drug Therapy]; RNA 16S; vancomycin/dt [Drug Therapy]; *Gordonia bronchialis; *sternal osteomyelitis/co [Complication]; *sternal osteomyelitis/dt [Drug Therapy].

Candidate Terms

*Gordonia bronchialis [other term]; *sternal osteomyelitis / *complication / *drug therapy [other term].

Drug Index Terms

amikacin; amoxicillin plus clavulanic acid; C reactive protein / endogenous compound; cefepime; cefotetan / drug therapy; ceftriaxone; ciprofloxacin; clarithromycin; cotrimoxazole; doxycycline; imipenem / drug therapy / intravenous drug administration; linezolid; minocycline; moxifloxacin; opiate / drug therapy; penicillin G / drug therapy; RNA 16S; vancomycin / drug therapy.

Other Index Terms

aged; antibiotic sensitivity; antibiotic therapy; article; atelectasis; bacterium culture; bacterium isolate; case report; cellulitis; computer assisted tomography; coronary artery bypass graft; diabetes mellitus; disease exacerbation; drug treatment failure; female; gene sequence; *Gordonia; human; hypertension; matrix assisted laser desorption ionization time of flight mass spectrometry; nonhuman; nucleotide sequence; *open heart surgery; *osteomyelitis / *complication / *drug therapy; osteomyelitis / drug therapy; postoperative pain / complication / drug therapy; pus; Rhodococcus; sternal osteomyelitis / drug therapy; sternotomy; substernal pain / complication / drug therapy; *surgical infection / *complication / *drug therapy; surgical infection / drug therapy; wound dehiscence; wound healing.

CAS Registry Numbers

37517-28-5 (amikacin); 39831-55-5 (amikacin); 74469-00-4 (amoxicillin plus clavulanic acid); 79198-29-1 (amoxicillin plus clavulanic

acid); 9007-41-4 (C reactive protein); 88040-23-7 (cefepime); 69712-56-7 (cefotetan); 74356-00-6 (cefotetan); 73384-59-5 (ceftriaxone); 74578-69-1 (ceftriaxone); 85721-33-1 (ciprofloxacin); 81103-11-9 (clarithromycin); 8064-90-2 (cotrimoxazole); 10592-13-9 (doxycycline); 17086-28-1 (doxycycline); 564-25-0 (doxycycline); 94088-85-4 (doxycycline); 64221-86-9 (imipenem); 165800-03-3 (linezolid); 10118-90-8 (minocycline); 11006-27-2 (minocycline); 13614-98-7 (minocycline); 151096-09-2 (moxifloxacin); 53663-61-9 (opiate); 8002-76-4 (opiate); 8008-60-4 (opiate); 1406-05-9 (penicillin G); 61-33-6 (penicillin G); 1404-90-6 (vancomycin); 1404-93-9 (vancomycin)

Year of Publication

2014

Cite

56.

Peritoneal-dialysis related peritonitis caused by **Gordonia** species: Report of four cases and literature review.

Ma T.K.-W., Chow K.-M., Kwan B.C.-H., Lee K.-P., Leung C.-B., Li P.K.-T., Szeto C.-C.

Embase
Nephrology. 19(7) (pp 379-383), 2014. Date of Publication: July 2014.

[Review]

AN:
373324470

Aim To investigate the clinical course and outcome of peritoneal dialysis-associated peritonitis secondary to **Gordonia** species. Method We reviewed all **Gordonia** peritonitis episodes occurring in a single dialysis unit from 1994 to 2013. Results During the study period, four episodes of **Gordonia** peritonitis were recorded. All were male patients. One patient responded to vancomycin therapy. One patient had refractory peritonitis despite vancomycin, but responded to imipenem and amikacin combination therapy. One patient had relapsing peritonitis and required catheter removal. The fourth patient had an elective Tenckhoff catheter exchange. No patient died of peritonitis. Causative organism was not fully identified until 7 to 18 days of peritonitis. Conclusion **Gordonia** species is increasingly recognized to cause serious infections. In patients undergoing peritoneal dialysis, **Gordonia** peritonitis should be considered in case of refractory Gram-positive bacilli peritonitis, especially when the exact organism could not be identified one week after the onset of peritonitis. A close liaison with a microbiologist is needed for a timely diagnosis. Summary at a Glance This manuscript discusses four cases of **Gordonia** species peritoneal dialysis related peritonitis in Hong Kong. This is a rare cause of PD peritonitis and this manuscript summarises the previous published cases and discusses the potential difficulties in diagnosis, recommended treatment regimen and the predicted patient outcomes. © 2014 Asian Pacific Society of Nephrology.

PMID

24655159 [https://www.ncbi.nlm.nih.gov/pubmed/?term=24655159]

Status

Embase

Institution

(Ma, Chow, Kwan, Leung, Li, Szeto) Department of Medicine and Therapeutics, Prince of Wales Hospital, Chinese University of Hong Kong, Shatin, Hong Kong (Lee) Department of Microbiology, Prince of Wales Hospital, Chinese University of Hong Kong, Hong Kong, Hong Kong

Publisher

Blackwell Publishing (E-mail: info@asia.blackpublishing.com.au)

Keyword Heading

Gordonia, infection, peritonitis, renal failure, survival

Emtree Heading

abdominal pain; adult; aged; antibiotic therapy; case report; catheter removal; continuous ambulatory peritoneal dialysis; diabetic nephropathy; disease course; disease duration; effluent; end stage renal disease/th [Therapy]; *Gordonia; heart atrium fibrillation;
human
; hypertension; immunoglobulin A nephropathy; ischemic heart disease; kidney transplantation; leukocyte count; male; neutrophil count; outcome assessment; peripheral vascular disease; *peritoneal dialysis; *peritonitis/co [Complication]; *peritonitis/dt [Drug

Therapy]; peritonitis/dt [Drug Therapy]; priority journal; relapse; review; treatment response; amikacin/cb [Drug Combination]; amikacin/dt [Drug Therapy]; amoxicillin plus clavulanic acid; cefazolin/dt [Drug Therapy]; cefazolin/ip [Intraperitoneal Drug Administration]; ceftazidime/dt [Drug Therapy]; ceftazidime/ip [Intraperitoneal Drug Administration]; gentamicin; imipenem/cb [Drug Combination]; imipenem/dt [Drug Therapy]; meropenem/dt [Drug Therapy]; meropenem/ip [Intraperitoneal Drug Administration]; meropenem/iv [Intravenous Drug Administration]; teicoplanin; vancomycin/dt [Drug Therapy]; vancomycin/ip [Intraperitoneal Drug Administration].

Drug Index Terms

amikacin / drug combination / drug therapy; amoxicillin plus clavulanic acid; cefazolin / drug therapy / intraperitoneal drug administration; ceftazidime / drug therapy / intraperitoneal drug administration; gentamicin; imipenem / drug combination / drug therapy; meropenem / drug therapy / intraperitoneal drug administration / intravenous drug administration; teicoplanin; vancomycin / drug therapy / intraperitoneal drug administration.

Other Index Terms

abdominal pain; adult; aged; antibiotic therapy; case report; catheter removal; continuous ambulatory peritoneal dialysis; diabetic nephropathy; disease course; disease duration; effluent; end stage renal disease / therapy; *Gordonia; heart atrium fibrillation; human; hypertension; immunoglobulin A nephropathy; ischemic heart disease; kidney transplantation; leukocyte count; male; neutrophil count; outcome assessment; peripheral vascular disease; *peritoneal dialysis; *peritonitis / *complication / *drug therapy; peritonitis / drug therapy; priority journal; relapse; review; treatment response.

CAS Registry Numbers

37517-28-5 (amikacin); 39831-55-5 (amikacin); 74469-00-4 (amoxicillin plus clavulanic acid); 79198-29-1 (amoxicillin plus clavulanic acid); 25953-19-9 (cefazolin); 27164-46-1 (cefazolin); 72558-82-8 (ceftazidime); 1392-48-9 (gentamicin); 1403-66-3 (gentamicin); 1405-41-0 (gentamicin); 64221-86-9 (imipenem); 96036-03-2 (meropenem); 61036-62-2 (teicoplanin); 61036-64-4 (teicoplanin); 1404-90-6 (vancomycin); 1404-93-9 (vancomycin)

Year of Publication

2014

Cite

57.

Gordonia terrae kidney graft abscess in a renal transplant patient.

Nicodemo A.C., Odongo F.C.A., Doi A.M., Sampaio J.L.M.

Embase
Transplant Infectious Disease. 16(4) (pp 681-686), 2014. Date of Publication: August 2014.

[Article]

AN:
53216030

We present the first report, to our knowledge, of a renal abscess cause by an **infection** from **Gordonia** terrae in a kidney transplant patient. The patient simultaneously had pulmonary tuberculosis and a perirenal allograft abscess caused by G. terrae. After treatment with imipenem, in addition to anti-tuberculous drugs, the patient was cured. © 2014 Wiley Periodicals, Inc.

PMID

24964822 [https://www.ncbi.nlm.nih.gov/pubmed/?term=24964822]

Status

Embase

Institution

(Nicodemo, Odongo) Department of Infectious and Parasitic Diseases, University of Sao Paulo Medical School, Sao Paulo, Brazil
(Doi) Microbiology Laboratory, Clinics Hospital, University of Sao Paulo Medical School, Sao Paulo, Brazil
(Sampaio) Department of Clinical Analysis, School of Pharmacy, University of Sao Paulo, Sao Paulo, Brazil

Publisher

Blackwell Publishing Inc. (E-mail: subscrip@blackwellpub.com)

Keyword Heading

16S rRNA gene, Gordonia terrae, Kidney transplant infection, Rhodococcus misidentification

Emtree Heading

acid fast bacterium; acute respiratory failure; adult; adult respiratory distress syndrome; allograft; ambient air; antimicrobial therapy; arterial gas; article; artificial ventilation; aspiration; blood cell count; chronic kidney failure/su [Surgery]; community acquired pneumonia/dt [Drug Therapy]; computer assisted tomography; controlled study; Cytomegalovirus; digestive system ulcer; disease severity; dyspnea; echography; endotracheal intubation; female; fever; fungemia; gastrointestinal endoscopy; *Gordonia; hemofiltration; hemoglobin blood level; hospital discharge;

human

; hypertension; hypoxemia; immunohistochemistry; immunosuppressive treatment; *kidney abscess/co [Complication]; *kidney abscess/et [Etiology]; kidney allograft; *kidney graft; kidney graft rejection/dt [Drug Therapy]; kidney graft rejection/pc [Prevention]; kidney transplantation; leukocyte count; lung infiltrate; lung nodule; lung tuberculosis/dt [Drug Therapy]; middle aged; miliary tuberculosis; Mycobacterium tuberculosis; nausea; nephrectomy; neutrophil count; pallor; percutaneous drainage; perirenal abscess; prescription; priority journal; protein blood level; pyelonephritis/su [Surgery]; recurrent disease; renal graft dysfunction/co [Complication]; renal graft dysfunction/th [Therapy]; respiratory distress; Rhodococcus; septic shock; thorax radiography; thrombocyte count; trachea mucus; C reactive protein/ec [Endogenous Compound]; ciprofloxacin/cb [Drug Combination]; ciprofloxacin/iv [Intravenous Drug Administration]; cotrimoxazole/dt [Drug Therapy]; ethambutol/cb [Drug Combination]; ethambutol/dt [Drug Therapy]; fluconazole; ganciclovir; hydrocortisone; imipenem/cb [Drug Combination]; imipenem/dt [Drug Therapy]; isoniazid/cb [Drug Combination]; isoniazid/dt [Drug Therapy]; levofloxacin/dt [Drug Therapy]; mycophenolic acid 2 morpholinoethyl ester/dt [Drug Therapy]; piperacillin plus tazobactam; prednisone/dt [Drug Therapy]; pyrazinamide/cb [Drug Combination]; pyrazinamide/dt [Drug Therapy]; rifampicin/cb [Drug Combination]; rifampicin/dt [Drug Therapy]; tacrolimus/dt [Drug Therapy]; vancomycin; vasoactive agent; *Gordonia terrae.

Candidate Terms

*Gordonia terrae [other term].

Drug Index Terms

C reactive protein / endogenous compound; ciprofloxacin / drug combination / intravenous drug administration; cotrimoxazole / drug therapy; ethambutol / drug combination / drug therapy; fluconazole; ganciclovir; hydrocortisone; imipenem / drug combination / drug therapy; isoniazid / drug combination / drug therapy; levofloxacin / drug therapy; mycophenolic acid 2 morpholinoethyl ester / drug therapy; piperacillin plus tazobactam; prednisone / drug therapy; pyrazinamide / drug combination / drug therapy; rifampicin / drug combination / drug therapy; tacrolimus / drug therapy; vancomycin; vasoactive agent.

Other Index Terms

acid fast bacterium; acute respiratory failure; adult; adult respiratory distress syndrome; allograft; ambient air; antimicrobial therapy; arterial gas; article; artificial ventilation; aspiration; blood cell count; chronic kidney failure / surgery; community acquired pneumonia / drug therapy; computer assisted tomography; controlled study; Cytomegalovirus; digestive system ulcer; disease severity; dyspnea; echography; endotracheal intubation; female; fever; fungemia; gastrointestinal endoscopy; *Gordonia; hemofiltration; hemoglobin blood level; hospital discharge; human; hypertension; hypoxemia; immunohistochemistry; immunosuppressive treatment; *kidney abscess / *complication / *etiology; kidney allograft; *kidney graft; kidney graft rejection / drug therapy / prevention; kidney transplantation; leukocyte count; lung infiltrate; lung nodule; lung tuberculosis / drug therapy; middle aged; miliary tuberculosis; Mycobacterium tuberculosis; nausea; nephrectomy; neutrophil count; pallor; percutaneous drainage; perirenal abscess; prescription; priority journal; protein blood level; pyelonephritis / surgery; recurrent disease; renal graft dysfunction / complication / therapy; respiratory distress; Rhodococcus; septic shock; thorax radiography; thrombocyte count; trachea mucus.

CAS Registry Numbers

9007-41-4 (C reactive protein); 85721-33-1 (ciprofloxacin); 8064-90-2 (cotrimoxazole); 10054-05-4 (ethambutol); 1070-11-7 (ethambutol); 3577-94-4 (ethambutol); 74-55-5 (ethambutol); 86386-73-4 (fluconazole); 82410-32-0 (ganciclovir); 50-23-7 (hydrocortisone); 64221-86-9 (imipenem); 54-85-3 (isoniazid); 62229-51-0 (isoniazid); 65979-32-0 (isoniazid); 100986-85-4 (levofloxacin); 138199-71-0 (levofloxacin); 116680-01-4 (mycophenolic acid 2 morpholinoethyl ester); 128794-94-5 (mycophenolic acid 2 morpholinoethyl ester); 53-03-2 (prednisone); 98-96-4 (pyrazinamide); 13292-46-1 (rifampicin); 104987-11-3 (tacrolimus); 1404-90-6 (vancomycin); 1404-93-9 (vancomycin)

Year of Publication

2014

Cite

58.

Trehalolipid biosurfactants from Rhodococcus ruber with anti-adhesive and immunomodulatory activities.
Kuyukina M., Ivshina I., Baeva T., Kochina O., Gein S.

Embase

New Biotechnology. Conference: 16th European Congress on Biotechnology. Edinburgh United Kingdom. Conference Publication: (var.pagings). 31(SUPPL. 1) (pp S54), 2014. Date of Publication: July 2014.

[Conference Abstract]

AN:
71771797

In recent years, glycolipid biosurfactants traditionally considered as emulsifying and solubilizing agents are attracting an increasing attention as possible biomedical agents with expressed biological activities [1]. Trehalolipids (TL) produced by members of closely related actinobacterial genera Rhodococcus, Nocardia, Corynebacterium, **Gordonia**, Mycobacterium, Tsukamurella, and Arthrobacter include,-D-trehalose, a nonreducing disaccharide, which is linked by an ester bond to long-chain fatty acids [2]. Well-known TL of pathogenic Mycobacterium tuberculosis, Corynebacterium diphtheriae play a key role in the infections caused by these actinobacteria, and they are characterized by high immunomodulatory activity [3]. However the pathogenicity of producers and high cytotoxicity of produced TL limit their biomedical applications. Therefore, the search for TL producers among nonpathogenic actinobacteria is essential. In in vitro experiments, TL biosurfactants from Rhodococcus ruber IEGM 231 stimulated both proinflammatory (interleukin (IL)-1b, IL-6, tumor necrosis factor-a (TNF-alpha) and anti-inflammatory (IL-12, IL-18) cytokine production of **human** monocytes, depending on cell culture composition and induction. Also, diverse anti-adhesive effects of TL towards **human** monocytes and bacterial species were revealed. Since TL from R. ruber displayed no cytotoxicity against **human** lymphocytes or bacterial cells, they could be proposed as potential immunomodulatory, antitumor and anti-adhesive agents.

Status

CONFERENCE ABSTRACT

Institution

(Kuyukina, Ivshina, Baeva, Kochina) Institute of Ecology and Genetics of Microorganisms, Russian Federation (Gein) Perm State University, Russian Federation

Publisher

Elsevier

Emtree Heading

*European; *biotechnology; *Rhodococcus ruber;
human
; monocyte; Actinobacteria; cytotoxicity; Arthrobacter; biological activity; Mycobacterium; pathogenicity;
Gordonia
;
infection
; Corynebacterium; Corynebacterium diphtheriae; cell culture; in vitro study; cytokine production; Mycobacterium tuberculosis; Nocardia; Rhodococcus; genus; species; lymphocyte; bacterial cell; *adhesive agent; solubilizer; cytokine; interleukin 6; interleukin 12; long chain fatty acid; factor A; ester; tumor necrosis factor; disaccharide; trehalose; glycolipid.

Drug Index Terms

*adhesive agent; solubilizer; cytokine; interleukin 6; interleukin 12; long chain fatty acid; factor A; ester; tumor necrosis factor; disaccharide; trehalose; glycolipid.

Other Index Terms

*European; *biotechnology; *Rhodococcus ruber; human; monocyte; Actinobacteria; cytotoxicity; Arthrobacter; biological activity; Mycobacterium; pathogenicity; Gordonia; infection; Corynebacterium; Corynebacterium diphtheriae; cell culture; in vitro study; cytokine production; Mycobacterium tuberculosis; Nocardia; Rhodococcus; genus; species; lymphocyte; bacterial cell.

Year of Publication

2014

Cite

59

Gordonia iterans sp. nov., isolated from a patient with pneumonia.

Kang Y.-Q., Ming H., Gonoi T., Chen Y., Cao Y., Wang Y.-Y., Cheng J., Koga T., Mikami Y., Li W.-J.

Embase
International journal of systematic and evolutionary microbiology. Part 10. 64 (pp 3520-3525), 2014. Date of Publication: 01 Oct 2014.

[Article]

AN:
604670535

A second novel clinical actinobacterial strain, designated IFM 10348(T), was isolated from the sputum of the same Japanese patient with bacterial pneumonia from whom the type strain of **Gordonia** araii had been isolated. The strains differed in phylogenetic position and drug-resistance profiles. The taxonomic position of strain IFM 10348(T) was clarified by phenotypic, chemotaxonomic and phylogenetic studies. Phylogenetic analyses based on 16S rRNA gene sequences clearly demonstrated that strain IFM 10348(T) occupied a distinct clade within the genus **Gordonia** and was related closely to **Gordonia** malaquae DSM 45064(T) and **Gordonia** hirsuta DSM 44140(T) (97.3 and 97.1% similarities, respectively). Strain IFM 10348(T) was also clearly differentiated from G. malaquae DSM 45064(T) and G. hirsuta DSM 44140(T) based on gyrB and secA1 gene sequence similarity values. Strain IFM 10348(T) had MK-9(H2) as the predominant menaquonine, contained meso-diaminopimelic acid, arabinose, galactose and glucosamine as cell-wall components, and contained C18:1omega9c, summed feature 3 (C16:1omega7c and/or C16:1omega6c) and C16:0 as the major cellular fatty acids. Mycolic acids were present. The DNA G+C content of strain IFM 10348(T) was 68.0 mol%. DNA-DNA relatedness data coupled with the combination of genotypic and phenotypic data indicated that strain IFM 10348(T) represents a novel species of the genus **Gordonia**, for which the name **Gordonia** iterans sp. nov. is proposed. The type strain is IFM 10348(T) (= CCTCC M2011245(T) = NCCB 100436(T)).
Copyright IUMS.

PMID

25052399 [https://www.ncbi.nlm.nih.gov/pubmed/?term=25052399]

Institution

(Kang) Department of Microbiology, Guiyang Medical College, Guiyang, 550004, PR China Medical Mycology Research Center (MMRC), Chiba University, Inohana, Chuo-ku, Chiba, Japan (Ming) Key Laboratory of Microbial Diversity in Southwest China, Ministry of Education, Yunnan Institute of Microbiology, Yunnan University, Kunming, 650091, PR China
(Gonoi) Medical Mycology Research Center (MMRC), Chiba University, Inohana, Chuo-ku, Chiba, Japan wjli@ynu.edu.cn gonoi@faculty.chiba-u.jp
(Chen) Department of Microbiology, Guiyang Medical College, Guiyang, 550004, PR China
(Cao) Department of Dermatology, the affiliated Hospital of Guiyang Medical College, Guiyang, 550004, PR China
(Wang) Department of Microbiology, Guiyang Medical College, Guiyang, 550004, PR China
(Cheng) Key Laboratory of Microbial Diversity in Southwest China, Ministry of Education, Yunnan Institute of Microbiology, Yunnan University, Kunming, 650091, PR China
(Koga) Department of Respiratory Medicine, Asakura Medical Association Hospital, 836-0069, Raiharu, Asakura-city, Fukuoka, Japan
(Mikami) Medical Mycology Research Center (MMRC), Chiba University, Inohana, Chuo-ku, Chiba, Japan
(Li) Key Laboratory of Microbial Diversity in Southwest China, Ministry of Education, Yunnan Institute of Microbiology, Yunnan University, Kunming, 650091, PR China wjli@ynu.edu.cn gonoi@faculty.chiba-u.jp

Emtree Heading

bacterial gene; bacterial pneumonia; cell wall; chemistry; *classification; DNA base composition; DNA sequence; genetics;
Gordonia
;
human
; isolation and purification; Japan; male; *microbiology; middle aged; molecular genetics; nucleic acid hybridization; *phylogeny; sputum; bacterial DNA; farnoquinone; fatty acid; menaquinone; mycolic acid; RNA 16S; analogs and derivatives.

Candidate Terms

analogs and derivatives [other term].

Drug Index Terms

bacterial DNA; farnoquinone; fatty acid; menaquinone; mycolic acid; RNA 16S.

Other Index Terms

bacterial gene; bacterial pneumonia; cell wall; chemistry; *classification; DNA base composition; DNA sequence; genetics; Gordonia;

human; isolation and purification; Japan; male; *microbiology; middle aged; molecular genetics; nucleic acid hybridization; *phylogeny; sputum.

CAS Registry Numbers

11032-49-8 (farnoquinone); 84-81-1 (farnoquinone); 37281-34-8 (mycolic acid)

Year of Publication

2014

Cite

60.

Other than "rhodococcus".

Su S.-Y., Chao C.-M., Lai C.-C.

Embase

American Journal of Medicine. 126(7) (pp e15), 2013. Date of Publication: July 2013.

[Letter]

AN:

369137708

PMID

23787201 [https://www.ncbi.nlm.nih.gov/pubmed/?term=23787201]

Status

Embase

Institution

(Su) Department of Emergency Medicine, Tainan Municipal Hospital, Tainan, Taiwan (Republic of China) (Chao, Lai) Department of Intensive Care Medicine, Chi Mei Medical Center, Liouying, Tainan, Taiwan (Republic of China)

Publisher

Elsevier Inc. (360 Park Avenue South, New York NY 10010, United States)

Emtree Heading

*Actinobacteria; bacterial colonization; bacterium identification; bacterium isolate; clinical feature; *Gordonia;

human

; letter; lung infection; lung tuberculosis; priority journal; *Rhodococcus equi; *Tsukamurella.

Candidate Terms

*Tsukamurella [other term].

Other Index Terms

*Actinobacteria; bacterial colonization; bacterium identification; bacterium isolate; clinical feature; *Gordonia; human; letter; lung infection; lung tuberculosis; priority journal; *Rhodococcus equi.

Year of Publication

2013

Cite

61.

Gordonia bacteremia.

Ramanan P., Deziel P.J., Wengenack N.L.

Embase

Journal of Clinical Microbiology. 51(10) (pp 3443-3447), 2013. Date of Publication: October 2013.

[Article]

AN:
369914673

Gordonia species are ubiquitous aerobic actinomycetes that rarely cause **infection** in humans. We report the second known case of **Gordonia** otitidis catheter-related bacteremia in an immunocompromised patient and review four additional cases of **Gordonia** bacteremia seen at our institution over the past 14 years. In addition, the existing literature on **Gordonia** infections is reviewed. Copyright © 2013, American Society for Microbiology.

PMID

23884999 [https://www.ncbi.nlm.nih.gov/pubmed/?term=23884999]

Status

Embase

Institution

(Ramanan, Deziel) Division of Infectious Diseases, United States (Wengenack) Division of Clinical Microbiology, Mayo Clinic, Rochester, MN, United States

Publisher

American Society for Microbiology (1752 N Street N.W., Washington DC 20036-2904, United States)

Emtree Heading

acute granulocytic leukemia/th [Therapy]; acute lymphoblastic leukemia; acute monocytic leukemia/th [Therapy]; adult; aged; allogeneic peripheral blood stem cell transplantation; article; bacteremia/dt [Drug Therapy]; *bacteremia/di [Diagnosis]; *bacteremia/dt [Drug Therapy]; *bacteremia/th [Therapy]; blood culture; catheter infection/di [Diagnosis]; catheter infection/dt [Drug Therapy]; catheter infection/th [Therapy]; catheter removal; child; clinical article; cyanosis; dialysis catheter; drug substitution; drug withdrawal; Enterococcus faecalis; female; fever; *Gordonia; graft versus host reaction/dt [Drug Therapy]; hemodialysis; herpes simplex encephalitis/dt [Drug Therapy]; Hickman catheter;
human
; hypotension; immunocompromised patient; kidney failure/th [Therapy]; male; nonhuman; preschool child; priority journal; RNA gene; sepsis/dt [Drug Therapy]; superior cava vein syndrome; swelling; aciclovir/dt [Drug Therapy]; cefazolin/cb [Drug Combination]; cefazolin/dt [Drug Therapy]; cefepime/cb [Drug Combination]; cefepime/dt [Drug Therapy]; ceftriaxone/dt [Drug Therapy]; ceftriaxone/iv [Intravenous Drug Administration]; clarithromycin/dt [Drug Therapy]; clarithromycin/po [Oral Drug Administration]; cotrimoxazole/dt [Drug Therapy]; cotrimoxazole/iv [Intravenous Drug Administration]; penicillin G/dt [Drug Therapy]; penicillin G/iv [Intravenous Drug Administration]; piperacillin plus tazobactam/cb [Drug Combination]; piperacillin plus tazobactam/dt [Drug Therapy]; RNA 16S; steroid/dt [Drug Therapy]; steroid/tp [Topical Drug Administration]; vancomycin/cb [Drug Combination]; vancomycin/dt [Drug Therapy]; vancomycin/iv [Intravenous Drug Administration]; Gordonia bronchialis; Gordonia otitidis; Gordonia polyisoprenivorans; gordonia terrae.

Candidate Terms

Gordonia bronchialis [other term]; Gordonia otitidis [other term]; Gordonia polyisoprenivorans [other term]; Gordonia terrae [other term].

Drug Index Terms

aciclovir / drug therapy; cefazolin / drug combination / drug therapy; cefepime / drug combination / drug therapy; ceftriaxone / drug therapy / intravenous drug administration; clarithromycin / drug therapy / oral drug administration; cotrimoxazole / drug therapy / intravenous drug administration; penicillin G / drug therapy / intravenous drug administration; piperacillin plus tazobactam / drug combination / drug therapy; RNA 16S; steroid / drug therapy / topical drug administration; vancomycin / drug combination / drug therapy / intravenous drug administration.

Other Index Terms

acute granulocytic leukemia / therapy; acute lymphoblastic leukemia; acute monocytic leukemia / therapy; adult; aged; allogeneic peripheral blood stem cell transplantation; article; bacteremia / drug therapy; *bacteremia / *diagnosis / *drug therapy / *therapy; blood culture; catheter infection / diagnosis / drug therapy / therapy; catheter removal; child; clinical article; cyanosis; dialysis catheter; drug substitution; drug withdrawal; Enterococcus faecalis; female; fever; *Gordonia; graft versus host reaction / drug therapy; hemodialysis; herpes simplex encephalitis / drug therapy; Hickman catheter; human; hypotension; immunocompromised patient; kidney failure / therapy; male; nonhuman; preschool child; priority journal; RNA gene; sepsis / drug therapy; superior cava vein syndrome; swelling.

CAS Registry Numbers

59277-89-3 (aciclovir); 25953-19-9 (cefazolin); 27164-46-1 (cefazolin); 88040-23-7 (cefepime); 73384-59-5 (ceftriaxone); 74578-69-1

(ceftriaxone); 81103-11-9 (clarithromycin); 8064-90-2 (cotrimoxazole); 1406-05-9 (penicillin G); 61-33-6 (penicillin G); 1404-90-6 (vancomycin); 1404-93-9 (vancomycin)

Year of Publication

2013

Cite

62.
First identification of **Gordonia** sputi in a continuous ambulatory peritoneal dialysis patient with Peritonitis.

Ou S.M., Lee S.Y., Chen J.Y., Cheng H.W., Wei T.H., Yu K.W., Lin W.M., King K.L., Yang W.C., Ng Y.Y.

Embase
Peritoneal Dialysis International. 33(1) (pp 107-108), 2013. Date of Publication: January - February 2013.

[Letter]

AN:
369110158

PMID

23349205 [https://www.ncbi.nlm.nih.gov/pubmed/?term=23349205]

Status

Embase

Institution

(Ou, Chen, Yang, Ng) Division of nephrology, Taipei Veterans General Hospital, National Yang-Ming University, School of Medicine, Taipei, Taiwan (Republic of China) (Ou, Chen, Wei, Yu, Yang, Ng) Department of Medicine, Taipei Veterans General Hospital, National Yang-Ming University, School of Medicine, Taipei, Taiwan (Republic of China)
(Lee, Cheng, Yu, Lin) Departments of Microbiology and Pathology and Laboratory Medicine, Taipei Veterans General Hospital, National Yang-Ming University, School of Medicine, Taipei, Taiwan (Republic of China)
(King) Department of Surgery, Taipei Veterans General Hospital, National Yang-Ming University, School of Medicine, Taipei, Taiwan (Republic of China)

Publisher

Multimed Inc. (66 Martin Street, Milton ONT L9T 2R2, Canada)

Keyword Heading

Corynebacterium, Gordonia sputi, Peritonitis

Emtree Heading

aged; antibiotic sensitivity; bacterial genome; bacterial peritonitis/dt [Drug Therapy]; *bacterial peritonitis/co [Complication]; *bacterial peritonitis/dt [Drug Therapy]; bacterium culture; broth dilution; case report; computer assisted tomography; *continuous ambulatory peritoneal dialysis; diabetic retinopathy/co [Complication]; diabetic retinopathy/th [Therapy]; dialysate; gene sequence; *Gordonia;
human
; kidney failure/th [Therapy]; letter; male; priority journal; recurrent disease; RNA sequence; sequence analysis; treatment duration; cefazolin/dt [Drug Therapy]; cefazolin/ip [Intraperitoneal Drug Administration]; gentamicin/dt [Drug Therapy]; gentamicin/ip [Intraperitoneal Drug Administration]; RNA 16S/ec [Endogenous Compound]; *gordonia sputi.

Candidate Terms

*Gordonia sputi [other term].

Drug Index Terms

cefazolin / drug therapy / intraperitoneal drug administration; gentamicin / drug therapy / intraperitoneal drug administration; RNA 16S / endogenous compound.

Other Index Terms

aged; antibiotic sensitivity; bacterial genome; bacterial peritonitis / drug therapy; *bacterial peritonitis / *complication / *drug therapy; bacterium culture; broth dilution; case report; computer assisted tomography; *continuous ambulatory peritoneal dialysis; diabetic retinopathy / complication / therapy; dialysate; gene sequence; *Gordonia; human; kidney failure / therapy; letter; male; priority journal; recurrent disease; RNA sequence; sequence analysis; treatment duration.

CAS Registry Numbers

25953-19-9 (cefazolin); 27164-46-1 (cefazolin); 1392-48-9 (gentamicin); 1403-66-3 (gentamicin); 1405-41-0 (gentamicin)

Year of Publication

2013

Cite

63.

Hickman catheter-related bacteremia caused by **gordonia** sputi in a patient with breast cancer.

Kofteridis D.P., Valachis A., Scoulica E., Christidou A., Maraki S., Samonis G.

Embase
Journal of Infection in Developing Countries. 6(2) (pp 188-191), 2012. Date of Publication: February 2012.

[Article]

AN:
364281997

The case of a Hickman catheter-related bacteremia caused by **Gordonia** sputi in a patient with breast cancer is presented. Blood cultures grew a Gram-positive rod, susceptible to several antimicrobials, subsequently identified by 16S rRNA gene sequencing as **Gordonia** sputi. The **infection** resolved after successful treatment with antibiotics and catheter removal. © 2012 Kofteridis et al.

PMID

22337850 [https://www.ncbi.nlm.nih.gov/pubmed/?term=22337850]

Status

Embase

Institution

(Kofteridis, Valachis, Samonis) Departments of Internal Medicine, University Hospital of Heraklion, Crete, Greece (Scoulica, Christidou, Maraki) Clinical Microbiology, University Hospital of Heraklion, Crete, Greece

Publisher

Journal of Infection in Developing Countries (E-mail: srubino@jidc.org)

Keyword Heading

Bacteremia, Central venous catheter, Gordonia sputi

Emtree Heading

Actinomycetales infection/dt [Drug Therapy]; aged; antibiotic sensitivity; antibiotic therapy; article; *bacteremia/dt [Drug Therapy]; bacterial growth; bacterial strain; bacterium culture; bacterium identification; bacterium isolate; blood culture; blood sampling; brain metastasis/dt [Drug Therapy]; breast cancer/dt [Drug Therapy]; breast cancer/rt [Radiotherapy]; breast cancer/su [Surgery]; cancer adjuvant therapy; cancer radiotherapy; cancer recurrence; cancer surgery; case report; *catheter infection/dt [Drug Therapy]; catheter removal; central venous catheter; colony forming unit; disease duration; female; fever; gene identification; gene sequence; *Gordonia;
human
; incubation temperature; liver metastasis/dt [Drug Therapy]; malaise; minimum inhibitory concentration; treatment duration; agar; amikacin/dt [Drug Therapy]; amoxicillin plus clavulanic acid/dt [Drug Therapy]; amoxicillin plus clavulanic acid/po [Oral Drug Administration]; antineoplastic agent/dt [Drug Therapy]; cefoxitin; ceftriaxone/dt [Drug Therapy]; gemcitabine/dt [Drug Therapy]; genomic DNA; meropenem/dt [Drug Therapy]; rifampicin; RNA 16S; teicoplanin/dt [Drug Therapy]; trastuzumab/dt [Drug Therapy]; *gordonia sputi.

Candidate Terms

*Gordonia sputi [other term].

Drug Index Terms

agar; amikacin / drug therapy; amoxicillin plus clavulanic acid / drug therapy / oral drug administration; antineoplastic agent / drug

therapy; cefoxitin; ceftriaxone / drug therapy; gemcitabine / drug therapy; genomic DNA; meropenem / drug therapy; rifampicin; RNA 16S; teicoplanin / drug therapy; trastuzumab / drug therapy.

Other Index Terms

Actinomycetales infection / drug therapy; aged; antibiotic sensitivity; antibiotic therapy; article; *bacteremia / *drug therapy; bacterial growth; bacterial strain; bacterium culture; bacterium identification; bacterium isolate; blood culture; blood sampling; brain metastasis / drug therapy; breast cancer / drug therapy / radiotherapy / surgery; cancer adjuvant therapy; cancer radiotherapy; cancer recurrence; cancer surgery; case report; *catheter infection / *drug therapy; catheter removal; central venous catheter; colony forming unit; disease duration; female; fever; gene identification; gene sequence; *Gordonia; human; incubation temperature; liver metastasis / drug therapy; malaise; minimum inhibitory concentration; treatment duration.

CAS Registry Numbers

9002-18-0 (agar); 37517-28-5 (amikacin); 39831-55-5 (amikacin); 74469-00-4 (amoxicillin plus clavulanic acid); 79198-29-1 (amoxicillin plus clavulanic acid); 33564-30-6 (cefoxitin); 35607-66-0 (cefoxitin); 73384-59-5 (ceftriaxone); 74578-69-1 (ceftriaxone); 103882-84-4 (gemcitabine); 96036-03-2 (meropenem); 13292-46-1 (rifampicin); 61036-62-2 (teicoplanin); 61036-64-4 (teicoplanin); 180288-69-1 (trastuzumab); 1446410-98-5 (trastuzumab)

Year of Publication

2012

Cite

64.
Gordonia bronchialis sternal wound **infection** in 3 patients following open heart surgery: Intraoperative transmission from a healthcare worker.

Wright S.N., Gerry J.S., Busowski M.T., Klochko A.Y., McNulty S.G., Brown S.A., Sieger B.E., Ken Michaels P., Wallace M.R.

Embase
Infection Control and Hospital Epidemiology. 33(12) (pp 1238-1241), 2012. Date of Publication: December 2012.

[Article]

AN:
366068966

We describe an investigation of 3 postoperative **Gordonia** bronchialis sternal infections. A nurse anesthetist was identified as the source of the outbreak, her scrubs likely becoming contaminated by her home washing machine. The outbreak ended after disposal of the implicated washing machine. Domestic laundering of surgical scrubs may need reevaluation. © 2012 by The Society for Healthcare Epidemiology of America. All rights reserved.

PMID

23143362 [https://www.ncbi.nlm.nih.gov/pubmed/?term=23143362]

Status

Embase

Institution

(Wright, Brown) Department of Infection Prevention and Control, Orlando Health, Orlando, FL, United States (Gerry) Department of Advanced Practice Nursing, Division of Cardiovascular Surgery, Orlando Regional Medical Center, Orlando, FL, United States
(Busowski, Klochko, Sieger, Wallace) Department of Internal Medicine, Division of Infectious Diseases, Orlando Health, Orlando, FL, United States
(McNulty) Department of Microbiology, Division of Mycobacteria/Nocardia Research Laboratory, University of Texas Health Science Center, Tyler, TX, United States
(Ken Michaels) Department of Family Medicine, Orlando Health, Orlando, FL, United States

Publisher

University of Chicago Press (1427 E. 60th Street, Chicago IL 60637-2954, United States)

Emtree Heading

*Actinomycetales infection/di [Diagnosis]; *Actinomycetales infection/dt [Drug Therapy]; *Actinomycetales infection/et [Etiology];

[illegible]

Gordonia

disease transmission; DNA fingerprinting; Gordon bronchialis sternal wound infection/dt [Drug Therapy];

; Gordonia bronchialis sternal wound infection/dt [Drug Therapy]; health care personnel; heart surgery;

human

; human tissue; intraoperative period; laundry; leukocytosis; machine; male; nurse anesthetist; pulsed field gel electrophoresis; scrub; sternum; treatment duration; treatment response; *wound infection/di [Diagnosis]; *wound infection/dt [Drug Therapy]; *wound infection/et [Etiology]; wound infection/dt [Drug Therapy]; imipenem/dt [Drug Therapy]; imipenem/iv [Intravenous Drug Administration]; linezolid/dt [Drug Therapy]; minocycline/dt [Drug Therapy]; moxifloxacin/dt [Drug Therapy]; Gordon bronchialis sternal wound infection/dt [Drug Therapy]; Gordonia bronchialis; *Gordonia bronchialis sternal wound infection/di [Diagnosis]; *Gordonia bronchialis sternal wound infection/dt [Drug Therapy]; *Gordonia bronchialis sternal wound infection/et [Etiology]; washing machine.

Candidate Terms

Gordon bronchialis sternal wound infection / drug therapy [other term]; Gordonia bronchialis [other term]; *Gordonia bronchialis sternal wound infection / *diagnosis / *drug therapy / *etiology [other term]; washing machine [other term].

Drug Index Terms

imipenem / drug therapy / intravenous drug administration; linezolid / drug therapy; minocycline / drug therapy; moxifloxacin / drug therapy.

Other Index Terms

*Actinomycetales infection / *diagnosis / *drug therapy / *etiology; Actinomycetales infection / drug therapy; adult; aged; antibiotic therapy; article; bacterium colony; bacterium culture; case report; disease transmission; DNA fingerprinting; Gordon bronchialis sternal wound infection / drug therapy; Gordonia; Gordonia bronchialis sternal wound infection / drug therapy; health care personnel; heart surgery; human; human tissue; intraoperative period; laundry; leukocytosis; machine; male; nurse anesthetist; pulsed field gel electrophoresis; scrub; sternum; treatment duration; treatment response; *wound infection / *diagnosis / *drug therapy / *etiology; wound infection / drug therapy.

CAS Registry Numbers

64221-86-9 (imipenem); 165800-03-3 (linezolid); 10118-90-8 (minocycline); 11006-27-2 (minocycline); 13614-98-7 (minocycline); 151096-09-2 (moxifloxacin)

Year of Publication

2012

Cite

65.

Tibial osteomyelitis caused by **Gordonia** bronchialis in an immunocompetent patient.

Siddiqui N., Toumeh A., Georgescu C.

Embase

Journal of Clinical Microbiology. 50(9) (pp 3119-3121), 2012. Date of Publication: September 2012.

[Article]

AN:
365469453

Gordonia species are aerobic Gram-positive bacilli that rarely cause **human** infections, often in the setting of indwelling intravascular catheters. We report the first case of osteomyelitis caused by **Gordonia** bronchialis in a healthy immunocompetent host in the absence of an intravascular catheter. Copyright © 2012, American Society for Microbiology. All Rights Reserved.

PMID

22692736 [https://www.ncbi.nlm.nih.gov/pubmed/?term=22692736]

Status

Embase

Institution

(Siddiqui, Toumeh) Department of Internal Medicinea, University of Toledo Medical Center, Toledo, OH, United States (Georgescu) Division of Infectious Diseases, University of Toledo Medical Center, Toledo, OH, United States

Publisher

American Society for Microbiology (1752 N Street N.W., Washington DC 20036-2904, United States)

Emtree Heading

abscess/co [Complication]; adult; antibiotic sensitivity; article; bacterium isolate; case report; drug hypersensitivity/si [Side Effect]; drug substitution; drug withdrawal; erythema/co [Complication]; female; gene sequence; *Gordonia; hamstring;

human

; immunocompetence; leg amputation; leg pain/co [Complication]; leg swelling/co [Complication]; meniscectomy; minimum inhibitory concentration; nonhuman; nucleotide sequence; osteomyelitis/dt [Drug Therapy]; *osteomyelitis/dt [Drug Therapy]; prescription; priority journal; tibial osteomyelitis/dt [Drug Therapy]; treatment outcome; amikacin; amoxicillin plus clavulanic acid; ceftriaxone; ciprofloxacin/dt [Drug Therapy]; ciprofloxacin/po [Oral Drug Administration]; clarithromycin; cotrimoxazole/ae [Adverse Drug Reaction]; cotrimoxazole/po [Oral Drug Administration]; doxycycline; imipenem; linezolid; minocycline; moxifloxacin; quinoline derived antiinfective agent/dt [Drug Therapy]; RNA 16S/ec [Endogenous Compound]; tobramycin; vancomycin/dt [Drug Therapy]; vancomycin/iv [Intravenous Drug Administration]; *Gordonia bronchialis; *tibial osteomyelitis/dt [Drug Therapy].

Candidate Terms

*gordonia bronchialis [other term]; *tibial osteomyelitis / *drug therapy [other term].

Drug Index Terms

amikacin; amoxicillin plus clavulanic acid; ceftriaxone; ciprofloxacin / drug therapy / oral drug administration; clarithromycin; cotrimoxazole / adverse drug reaction / oral drug administration; doxycycline; imipenem; linezolid; minocycline; moxifloxacin; quinoline derived antiinfective agent / drug therapy; RNA 16S / endogenous compound; tobramycin; vancomycin / drug therapy / intravenous drug administration.

Other Index Terms

abscess / complication; adult; antibiotic sensitivity; article; bacterium isolate; case report; drug hypersensitivity / side effect; drug substitution; drug withdrawal; erythema / complication; female; gene sequence; *Gordonia; hamstring; human; immunocompetence; leg amputation; leg pain / complication; leg swelling / complication; meniscectomy; minimum inhibitory concentration; nonhuman; nucleotide sequence; osteomyelitis / drug therapy; *osteomyelitis / *drug therapy; prescription; priority journal; tibial osteomyelitis / drug therapy; treatment outcome.

CAS Registry Numbers

37517-28-5 (amikacin); 39831-55-5 (amikacin); 74469-00-4 (amoxicillin plus clavulanic acid); 79198-29-1 (amoxicillin plus clavulanic acid); 73384-59-5 (ceftriaxone); 74578-69-1 (ceftriaxone); 85721-33-1 (ciprofloxacin); 81103-11-9 (clarithromycin); 8064-90-2 (cotrimoxazole); 10592-13-9 (doxycycline); 17086-28-1 (doxycycline); 564-25-0 (doxycycline); 94088-85-4 (doxycycline); 64221-86-9 (imipenem); 165800-03-3 (linezolid); 10118-90-8 (minocycline); 11006-27-2 (minocycline); 13614-98-7 (minocycline); 151096-09-2 (moxifloxacin); 32986-56-4 (tobramycin); 1404-90-6 (vancomycin); 1404-93-9 (vancomycin)

Year of Publication

2012

Cite

66.

Cutaneous **infection** caused by **Gordonia** amicalis after a traumatic injury.

Lai C.-C., Hsieh J.-H., Tsai H.-Y., Liao C.-H., Hsueh P.-R.

Embase
Journal of Clinical Microbiology. 50(5) (pp 1821-1822), 2012. Date of Publication: May 2012.

[Article]

AN:
364660156

Gordonia amicalis **infection** has never been reported in humans. We report here the first case of G. amicalis-related cutaneous **infection** after a traumatic injury. The isolate was confirmed by 16S rRNA sequencing analysis, and the patient responded well to repeated debridement and antibiotic treatment. Copyright © 2012, American Society for Microbiology. All Rights Reserved.

PMID

22337976 [https://www.ncbi.nlm.nih.gov/pubmed/?term=22337976]

Status

Embase

Institution

(Lai) Department of Intensive Care Medicine, Chi Mei Medical Center, Liouying, Tainan, Taiwan (Republic of China) (Hsieh, Liao) Department of Surgery, National Taiwan University Hospital, National Taiwan University College of Medicine, Taipei, Taiwan (Republic of China)
(Tsai) Department of Internal Medicine, Far Eastern Memorial Hospital, New Taipei City, Taiwan (Republic of China)
(Tsai, Hsueh) Department of Internal Medicine, National Taiwan University Hospital, National Taiwan University College of Medicine, Taipei, Taiwan (Republic of China)
(Hsueh) Department of Laboratory Medicine, National Taiwan University Hospital, National Taiwan University College of Medicine, Taipei, Taiwan (Republic of China)

Publisher

American Society for Microbiology (1752 N Street N.W., Washington DC 20036-2904, United States)

Emtree Heading

adult; alanine aminotransferase blood level; arm swelling; article; aspartate aminotransferase blood level; bacterium culture; case report; cell infiltration; creatinine blood level; debridement; edema; *finger injury/su [Surgery]; finger phalanx; *Gordonia; Gram positive infection/dt [Drug Therapy]; granulation tissue; histopathology;
human
; leukocyte count; male; nuclear magnetic resonance imaging; nucleotide sequence; physical examination; postoperative period; priority journal; *skin infection; skin injury; skin ulcer; urea nitrogen blood level; alanine aminotransferase/ec [Endogenous Compound]; amoxicillin plus clavulanic acid/dt [Drug Therapy]; amoxicillin plus clavulanic acid/po [Oral Drug Administration]; aspartate aminotransferase/ec [Endogenous Compound]; creatinine/ec [Endogenous Compound]; nitrogen/ec [Endogenous Compound]; sodium/ec [Endogenous Compound]; sultamicillin/dt [Drug Therapy]; sultamicillin/iv [Intravenous Drug Administration]; urea/ec [Endogenous Compound]; *Gordonia amicalis.

Candidate Terms

*Gordonia amicalis [other term].

Drug Index Terms

alanine aminotransferase / endogenous compound; amoxicillin plus clavulanic acid / drug therapy / oral drug administration; aspartate aminotransferase / endogenous compound; creatinine / endogenous compound; nitrogen / endogenous compound; sodium / endogenous compound; sultamicillin / drug therapy / intravenous drug administration; urea / endogenous compound.

Other Index Terms

adult; alanine aminotransferase blood level; arm swelling; article; aspartate aminotransferase blood level; bacterium culture; case report; cell infiltration; creatinine blood level; debridement; edema; *finger injury / *surgery; finger phalanx; *Gordonia; Gram positive infection / drug therapy; granulation tissue; histopathology; human; leukocyte count; male; nuclear magnetic resonance imaging; nucleotide sequence; physical examination; postoperative period; priority journal; *skin infection; skin injury; skin ulcer; urea nitrogen blood level.

CAS Registry Numbers

9000-86-6 (alanine aminotransferase); 9014-30-6 (alanine aminotransferase); 74469-00-4 (amoxicillin plus clavulanic acid); 79198-29-1 (amoxicillin plus clavulanic acid); 9000-97-9 (aspartate aminotransferase); 19230-81-0 (creatinine); 60-27-5 (creatinine); 7727-37-9 (nitrogen); 7440-23-5 (sodium); 76497-13-7 (sultamicillin); 57-13-6 (urea)

Year of Publication

2012

Cite

67.

Pattern of antimicrobial susceptibility obtained from blood isolates of a rare but emerging **human** pathogen, **Gordonia** polyisoprenivorans.

Moser B.D., Pellegrini G.J., Lasker B.A., Brown J.M.

Embase
Antimicrobial Agents and Chemotherapy. 56(9) (pp 4991-4993), 2012. Date of Publication: September 2012.

[Letter]

AN:
365513123

PMID

22751545 [https://www.ncbi.nlm.nih.gov/pubmed/?term=22751545]

Status

Embase

Institution

(Moser, Pellegrini, Lasker, Brown) Special Bacteriology Reference Laboratory, Division of High-Consequence Pathogens and Pathology, Centers for Disease Control and Prevention, Atlanta, GA, United States

Publisher

American Society for Microbiology (1752 N Street N.W., Washington DC 20036-2904, United States)

Emtree Heading

antibiotic resistance; *antibiotic sensitivity; antibiotic therapy; bacterium isolate; biofilm; bloodstream infection; endocarditis/dt [Drug Therapy]; *Gordonia;
human
; letter; minimum inhibitory concentration; nonhuman; opportunistic infection; pneumonia; priority journal; amikacin/cr [Drug Concentration]; amoxicillin plus clavulanic acid/cr [Drug Concentration]; ampicillin/cr [Drug Concentration]; biosurfactant; ceftriaxone/cr [Drug Concentration]; ciprofloxacin/cr [Drug Concentration]; clarithromycin/cr [Drug Concentration]; cotrimoxazole/cr [Drug Concentration]; DNA topoisomerase (ATP hydrolysing) B; imipenem/cr [Drug Concentration]; linezolid/cr [Drug Concentration]; minocycline/cr [Drug Concentration]; piperacillin plus tazobactam/dt [Drug Therapy]; RNA 16S; tigecycline/cr [Drug Concentration]; vancomycin/cr [Drug Concentration]; *Gordonia polyisoprenivorans.

Candidate Terms

*Gordonia polyisoprenivorans [other term].

Drug Index Terms

amikacin / drug concentration; amoxicillin plus clavulanic acid / drug concentration; ampicillin / drug concentration; biosurfactant; ceftriaxone / drug concentration; ciprofloxacin / drug concentration; clarithromycin / drug concentration; cotrimoxazole / drug concentration; DNA topoisomerase (ATP hydrolysing) B; imipenem / drug concentration; linezolid / drug concentration; minocycline / drug concentration; piperacillin plus tazobactam / drug therapy; RNA 16S; tigecycline / drug concentration; vancomycin / drug concentration.

Other Index Terms

antibiotic resistance; *antibiotic sensitivity; antibiotic therapy; bacterium isolate; biofilm; bloodstream infection; endocarditis / drug therapy; *Gordonia; human; letter; minimum inhibitory concentration; nonhuman; opportunistic infection; pneumonia; priority journal.

CAS Registry Numbers

37517-28-5 (amikacin); 39831-55-5 (amikacin); 74469-00-4 (amoxicillin plus clavulanic acid); 79198-29-1 (amoxicillin plus clavulanic acid); 69-52-3 (ampicillin); 69-53-4 (ampicillin); 7177-48-2 (ampicillin); 74083-13-9 (ampicillin); 94586-58-0 (ampicillin); 73384-59-5 (ceftriaxone); 74578-69-1 (ceftriaxone); 85721-33-1 (ciprofloxacin); 81103-11-9 (clarithromycin); 8064-90-2 (cotrimoxazole); 64221-86-9 (imipenem); 165800-03-3 (linezolid); 10118-90-8 (minocycline); 11006-27-2 (minocycline); 13614-98-7 (minocycline); 220620-09-7 (tigecycline); 1404-90-6 (vancomycin); 1404-93-9 (vancomycin)

Year of Publication

2012

Cite

68.

Peritonitis caused by Haemophilus parainfluenzae, Leifsonia aquatica, and **Gordonia** spp. in a patient undergoing continuous ambulatory peritoneal dialysis.

Gardenier J.C., Sawyer R.G., Sifri C.D., Brayman K., Wispelway B., Bonatti H.

Embase
Surgical Infections. 13(6) (pp 409-412), 2012. Date of Publication: 01 Dec 2012.

[Article]

AN:
368001936

Background: Peritonitis has remained the most common serious complication of continuous ambulatory peritoneal dialysis (CAPD). In most cases, these infections are monomicrobial, and the pathogens involved most commonly are Staphylococci. Recently, polymicrobial infections with rare organisms have been reported more often. Case Report: We describe a patient who developed recurrent episodes of CAPD-associated peritonitis with a total of four pathogens: Methicillin-resistant S. aureus, Haemophilus parainfluenzae, Leifsonia aquatica, and **Gordonia** spp. The **infection** most likely was acquired when the patient used tap water for dialysis during a camping trip. All episodes were treated successfully with antibiotics. Finally, the device was removed, and later, a new catheter was implanted, which still is in use.
Conclusion(s): Peritoneal dialysis-associated peritonitis may be caused by rare organisms. Antibiotics may be able to treat disease temporarily, but removal of contaminated catheters usually is required. © Copyright 2012, Mary Ann Liebert, Inc. 2012.

PMID

23268614 [https://www.ncbi.nlm.nih.gov/pubmed/?term=23268614]

Status

Embase

Institution

(Gardenier, Sawyer, Brayman, Bonatti) Department of Surgery, University of Virginia Health System, Charlottesville, VA, United States
(Sifri, Wispelway) Division of Infectious Diseases and International Health, Department of Medicine, University of Virginia Health System, Charlottesville, VA, United States
(Bonatti) Department of Surgery, Vanderbilt University Medical Center, Nashville, TN, United States

Publisher

Mary Ann Liebert Inc. (140 Huguenot Street, New Rochelle NY 10801-5215, United States)

Emtree Heading

abdominal pain; adult; antibiotic sensitivity; article; breast cancer/rt [Radiotherapy]; breast cancer/su [Surgery]; cancer chemotherapy; case report; chronic kidney disease/th [Therapy]; continuous ambulatory peritoneal dialysis; disk diffusion; female; fever; *Gordonia; *Gram positive bacterium; *Haemophilus parainfluenzae; hemodialysis;

human

; leukocytosis; mastectomy; methicillin resistant Staphylococcus aureus; minimum inhibitory concentration; peritoneal fluid; *peritonitis/co [Complication]; *peritonitis/et [Etiology]; priority journal; amoxicillin plus clavulanic acid/cb [Drug Combination]; ceftriaxone; ciprofloxacin; cotrimoxazole/cb [Drug Combination]; vancomycin/cb [Drug Combination]; vancomycin/iv [Intravenous Drug Administration]; *Leifsonia aquatica.

Candidate Terms

*Leifsonia aquatica [other term].

Drug Index Terms

amoxicillin plus clavulanic acid / drug combination; ceftriaxone; ciprofloxacin; cotrimoxazole / drug combination; vancomycin / drug combination / intravenous drug administration.

Other Index Terms

abdominal pain; adult; antibiotic sensitivity; article; breast cancer / radiotherapy / surgery; cancer chemotherapy; case report; chronic kidney disease / therapy; continuous ambulatory peritoneal dialysis; disk diffusion; female; fever; *Gordonia; *Gram positive bacterium; *Haemophilus parainfluenzae; hemodialysis; human; leukocytosis; mastectomy; methicillin resistant Staphylococcus aureus; minimum inhibitory concentration; peritoneal fluid; *peritonitis / *complication / *etiology; priority journal.

CAS Registry Numbers

74469-00-4 (amoxicillin plus clavulanic acid); 79198-29-1 (amoxicillin plus clavulanic acid); 73384-59-5 (ceftriaxone); 74578-69-1 (ceftriaxone); 85721-33-1 (ciprofloxacin); 8064-90-2 (cotrimoxazole); 1404-90-6 (vancomycin); 1404-93-9 (vancomycin)

Year of Publication

2012

Cite

69.

Gordona: A rare cause of peritoneal dialysis peritonitis.

Imran M., Livesley P., Bell G., Pai P., Neal T., Anijeet H.

Embase
Peritoneal Dialysis International. 32(3) (pp 344-346), 2012. Date of Publication: May-June 2012.

[Short Survey]

AN:
364922854

PMID

22641739 [https://www.ncbi.nlm.nih.gov/pubmed/?term=22641739]

Status

Embase

Institution

(Imran, Livesley, Bell, Pai, Anijeet) Department of Nephrology, Royal Liverpool University Hospital NHS Trust, United Kingdom (Neal) Department of Medical Microbiology, Royal Liverpool University Hospital NHS Trust, Liverpool, United Kingdom

Publisher

Multimed Inc. (66 Martin Street, Milton ONT L9T 2R2, Canada)

Emtree Heading

abdominal pain; aged; antibiotic sensitivity; *bacterial peritonitis/co [Complication]; *bacterial peritonitis/dt [Drug Therapy]; *bacterial peritonitis/et [Etiology]; bacterial peritonitis/dt [Drug Therapy]; bacterium identification; case report; catheter removal; *continuous ambulatory peritoneal dialysis; drug efficacy; drug tolerability; female; *Gordonia;
human
; leukocyte count; priority journal; short survey; gentamicin/dt [Drug Therapy]; gentamicin/ip [Intraperitoneal Drug Administration]; RNA 16S; teicoplanin/dt [Drug Therapy]; teicoplanin/iv [Intravenous Drug Administration]; vancomycin/dt [Drug Therapy]; vancomycin/ip [Intraperitoneal Drug Administration].

Drug Index Terms

gentamicin / drug therapy / intraperitoneal drug administration; RNA 16S; teicoplanin / drug therapy / intravenous drug administration; vancomycin / drug therapy / intraperitoneal drug administration.

Other Index Terms

abdominal pain; aged; antibiotic sensitivity; *bacterial peritonitis / *complication / *drug therapy / *etiology; bacterial peritonitis / drug therapy; bacterium identification; case report; catheter removal; *continuous ambulatory peritoneal dialysis; drug efficacy; drug tolerability; female; *Gordonia; human; leukocyte count; priority journal; short survey.

CAS Registry Numbers

1392-48-9 (gentamicin); 1403-66-3 (gentamicin); 1405-41-0 (gentamicin); 61036-62-2 (teicoplanin); 61036-64-4 (teicoplanin); 1404-90-6 (vancomycin); 1404-93-9 (vancomycin)

Year of Publication

2012

Cite

70.
Mycolic acids - Potential biomarkers of opportunistic infections caused by bacteria of the suborder corynebacterineae.
Kwasy mikolowe - Potencjalne markery diagnostyki oportunistycznych zakazen mikroorganizmami z podrzedu corynebacterineae
<Kwasy mikolowe - Potencjalne markery diagnostyki oportunistycznych zakazen mikroorganizmami z podrzedu corynebacterineae.>

Kowalski K., Szewczyk R., Druszczynska M.

Embase
Postepy Higieny i Medycyny Doswiadczalnej. 66 (pp 461-468), 2012. Date of Publication: 2012.

[Review]

AN:
365930795

Mycolic acids are one of the basic elements of the cell wall structure of bacteria belonging to the suborder Corynebacterineae, constituting from 20% to 40% of dry weight. Additionally, they show high structural diversity within each family and species. Nowadays, profiles of mycolic acids are widely described for the genus Mycobacterium, the causative agent of tuberculosis. However, the suborder Corynebacterineae also includes many representatives of opportunistic **human** pathogens, e.g. Dietzia, **Gordonia**, Nocardia and Rhodococcus. Currently, an increased **infection** risk caused by this group of microorganisms especially in immunocompromised patients has been observed. Better knowledge of mycolic acid profiles for Corynebacterineae may allow identification of mycolic acids as diagnostic markers in the detection of opportunistic bacterial infections. Modern techniques of chemical analysis, including mass spectrometry, may enable the development of new chemotaxonomic methods for the detection and differentiation of bacteria within the suborder Corynebacterineae.

PMID

22922146 [https://www.ncbi.nlm.nih.gov/pubmed/?term=22922146]

Institution

(Kowalski, Druszczynska) Zaklad Immunologii Komorkowej, Katedra Immunologii i Biologii Infekcyjnej, Instytut Mikrobiologii, Biotechnologii i Immunologii, Uniwersytet Lodzki, Poland (Kowalski) Laboratorium Chemii Biomedycznej, Instytut Immunologii i Terapii Doswiadczalnej, PAN im. L. Hirszfelda, u. R.Weigla 12, 53-114 Wroclaw, Poland
(Szewczyk) Katedra Mikrobiologii Przemyslowej i Biotechnologii, Instytut Mikrobiologii, Biotechnologii i Immunologii, Uniwersytet Lodzki, Poland

Publisher

Polska Akademia Nauk (Skrytka Pocztowa 24, Warsaw 00-901, Poland)

Keyword Heading

Biomarkers, Corynebacterineae, Mycolic acids

Emtree Heading

*Actinomycetales infection/di [Diagnosis]; cell wall; chemistry; Corynebacterium infection/di [Diagnosis];
Gordonia
;
human
; metabolism; microbiology; Nocardia; *opportunistic infection/di [Diagnosis]; review; Rhodococcus; biological marker/an [Drug Analysis]; *mycolic acid/an [Drug Analysis].

Drug Index Terms

biological marker / drug analysis; *mycolic acid / *drug analysis.

Other Index Terms

*Actinomycetales infection / *diagnosis; cell wall; chemistry; Corynebacterium infection / diagnosis; Gordonia; human; metabolism; microbiology; Nocardia; *opportunistic infection / *diagnosis; review; Rhodococcus.

CAS Registry Numbers

37281-34-8 (mycolic acid)

Year of Publication

2012

Cite

71.
Improved identification of **Gordonia**, Rhodococcus and Tsukamurella species by 5'-end 16s rRNA gene sequencing.

Wang T., Kong F., Chen S., Xiao M., Sorrell T., Wang X., Wang S., Sintchenko V.

Embase
Pathology. 43(1) (pp 58-63), 2011. Date of Publication: January 2011.

[Article]

AN:
362442064

Objectives: The identification of fastidious aerobic Actinomycetes such as **Gordonia**, Rhodococcus, and Tsukamurella has remained a challenge leading to clinically significant misclassifications. This study is intended to examine the feasibility of partial 50-end 16S rRNA gene sequencing for the identification of **Gordonia**, Rhodococcus, and Tsukamurella, and defined potential reference sequences for species from each of these genera.
Method(s): The 16S rRNA gene sequence based identification algorithm for species identification was used and enhanced by aligning test sequences with reference sequences from the List of Prokaryotic Names with Standing in Nomenclature.
Result(s): Conventional PCR based 16S rRNA gene sequencing and the alignment of the isolate 16S rRNA gene sequence with reference sequences accurately identified 100% of clinical strains of aerobic Actinomycetes. While partial 16S rRNA gene sequences of reference type strains matched with the 16S rRNA gene sequences of 19 isolates in our data set, another 13 strains demonstrated a degree of polymorphism with a 1-4 bp difference in the regions of difference.
Conclusion(s): 50-end 606 bp 16S rRNA gene sequencing, coupled with the assignment of well defined reference sequences to clinically relevant species of bacteria, can be a useful strategy for improving the identification of clinically relevant aerobic Actinomycetes. © 2010 Royal College of Pathologists of Australasia.

PMID

21240067 [https://www.ncbi.nlm.nih.gov/pubmed/?term=21240067]

Institution

(Wang, Wang) Department of Neurosurgery, Beijing Tian Tan Hospital, Capital Medical University, China (Xiao) Life Science College, Peking University, Beijing, China
(Wang) Departments of Neurosurgery, Affiliated Hospital of Inner Mongolia Medical College, Inner Mongolia, China
(Wang) Departments of Dermatology and Venereology, Affiliated Hospital of Inner Mongolia Medical College, Inner Mongolia, China
(Kong, Chen, Sorrell, Sintchenko) Centre for Infectious Diseases and Microbiology, Westmead Hospital, Darcy Road, Westmead, NSW 2145, Australia
(Chen, Sorrell, Sintchenko) Sydney Medical School, University of Sydney, Sydney, NSW, Australia

Publisher

Lippincott Williams and Wilkins (530 Walnut Street,P O Box 327, Philadelphia PA 19106-3621, United States)

Keyword Heading

16S rRNA gene, Actinomycetes, Gordonia, Reference sequence, Rhodococcus, Sequence based identification, Sequence polymorphisms, Tsukamurella

Emtree Heading

Actinomycetales infection/di [Diagnosis]; article; chemistry; *DNA flanking region; genetics; *Gordonia;
human
; isolation and purification; metabolism; microbiological examination; microbiology; molecular genetics; *Rhodococcus; sensitivity and specificity; sequence analysis; species difference; bacterial DNA/an [Drug Analysis]; bacterial RNA/an [Drug Analysis]; *RNA 16S.

Drug Index Terms

bacterial DNA / drug analysis; bacterial RNA / drug analysis; *RNA 16S.

Other Index Terms

Actinomycetales infection / diagnosis; article; chemistry; *DNA flanking region; genetics; *Gordonia; human; isolation and purification; metabolism; microbiological examination; microbiology; molecular genetics; *Rhodococcus; sensitivity and specificity; sequence analysis; species difference.

Year of Publication

2011

Cite

72.

Tsukamurella tyrosinosolvens intravascular catheter-related bacteremia in a haematology patient: a case report.

Karunakaran R., Halim H.A., Ng K.P., Hanifah Y.A., Chin E., Jaafar F.L., Abubakar S.

Embase
European review for medical and pharmacological sciences. 15(11) (pp 1343-1346), 2011. Date of Publication: Nov 2011.

[Article]

AN:
560021198

Tsukamurella spp. are a rare but important cause of intravascular catheter-related bacteremia in immunocompromised patients. The organism is an aerobic, Gram-positive, weakly acid-fast bacillus that is difficult to differentiate using standard laboratory methods from other aerobic actinomycetales such as Nocardia spp., Rhododoccus spp., **Gordonia** spp., and the rapid growing Mycobacterium spp. We report a case of Tsukamurella tyrosinosolvens catheter-related bacteremia in a 51-year-old haematology patient who responded to treatment with imipenem and subsequent line removal. 16srRNA sequencing allowed for the prompt identification of this organism.

PMID

22195371 [https://www.ncbi.nlm.nih.gov/pubmed/?term=22195371]

Institution

(Karunakaran) Department of Medical Microbiology, Faculty of Medicine, University of Malaya, 50603 Kuala Lumpur, Malaysia.

Emtree Heading

Actinomycetales; *Actinomycetales infection/dt [Drug Therapy]; acute granulocytic leukemia/co [Complication]; acute granulocytic leukemia/dt [Drug Therapy]; article; *bacteremia; *catheter infection/dt [Drug Therapy]; central venous catheterization; drug effect; female; genetics;
human
; methicillin resistant Staphylococcus aureus; microbiology; middle aged; antiinfective agent/dt [Drug Therapy]; bacterial RNA/an [Drug Analysis]; imipenem/dt [Drug Therapy]; RNA 16S/an [Drug Analysis].

Drug Index Terms

antiinfective agent / drug therapy; bacterial RNA / drug analysis; imipenem / drug therapy; RNA 16S / drug analysis.

Other Index Terms

Actinomycetales; *Actinomycetales infection / *drug therapy; acute granulocytic leukemia / complication / drug therapy; article; *bacteremia; *catheter infection / *drug therapy; central venous catheterization; drug effect; female; genetics; human; methicillin resistant Staphylococcus aureus; microbiology; middle aged.

CAS Registry Numbers

64221-86-9 (imipenem)

Year of Publication

2011

Cite

73.
Gordonia bronchialis bacteremia and pleural **infection**: Case report and review of the literature.

Johnson J.A., Onderdonk A.B., Cosimi L.A., Yawetz S., Lasker B.A., Bolcen S.J., Brown J.M., Marty F.M.

Embase
Journal of Clinical Microbiology. 49(4) (pp 1662-1666), 2011. Date of Publication: April 2011.

[Article]

AN:
361590455

Gordonia species are aerobic actinomycetes recently recognized as causing **human** disease, often in the setting of intravascular catheter-related infections. We describe a case of **Gordonia** bronchialis bacteremia and pleural space **infection** in the absence of an indwelling intravascular catheter and review the breadth of reported infections with this emerging pathogen. Copyright © 2011, American Society for Microbiology.

PMID

21270217 [https://www.ncbi.nlm.nih.gov/pubmed/?term=21270217]

Status

Embase

Institution

(Johnson, Cosimi, Yawetz, Marty) Division of Infectious Diseases, Brigham and Women's Hospital, Harvard Medical Schoo, 75 Francis Street, Boston, MA 02115, United States (Onderdonk) Department of Pathology, Brigham and Women's Hospital, Harvard Medical Schoo, Boston, MA, United States
(Lasker, Bolcen, Brown) Bacterial Special Pathogens Branch, Division of High-Consequence Pathogens and Pathology, National Center for Emerging and Zoonotic Infectious Diseases, Atlanta, GA, United States

Publisher

American Society for Microbiology (1752 N Street N.W., Washington DC 20036-2904, United States)

Emtree Heading

Actinomycetales infection/dt [Drug Therapy]; adult; antibiotic sensitivity; anticoagulation; aorta valve disease; article; artificial heart pacemaker; *bacteremia/dt [Drug Therapy]; bacteremia/dt [Drug Therapy]; bacterium identification; bacterium isolate; blood culture; breast cancer/dt [Drug Therapy]; breast cancer/su [Surgery]; case report; catheter infection; central venous catheter; clinical feature; female; *Gordonia; heart atrium fibrillation; heart surgery; heart valve prosthesis; Hodgkin disease;
human
; human tissue; indwelling catheter; mastectomy; *nucleotide sequence; pleura biopsy; *pleura disease; pleura effusion; priority journal; spleen disease/su [Surgery]; splenectomy; video assisted thoracoscopic surgery; amikacin/dt [Drug Therapy]; amoxicillin plus clavulanic acid/dt [Drug Therapy]; anastrozole/dt [Drug Therapy]; ceftazidime/dt [Drug Therapy]; ceftriaxone/dt [Drug Therapy]; cilastatin plus imipenem/dt [Drug Therapy]; ciprofloxacin/dt [Drug Therapy]; ciprofloxacin/po [Oral Drug Administration]; cotrimoxazole/dt [Drug Therapy]; cotrimoxazole/iv [Intravenous Drug Administration]; cotrimoxazole/po [Oral Drug Administration]; linezolid/dt [Drug Therapy]; minocycline/dt [Drug Therapy]; minocycline/po [Oral Drug Administration]; tamoxifen/dt [Drug Therapy]; tigecycline/dt [Drug Therapy]; vancomycin/dt [Drug Therapy]; *Gordonia bronchialis; Gordonia otitidis; Gordonia polyisoprenivorans; gordonia sputi; gordonia terrae.

Candidate Terms

*Gordonia bronchialis [other term]; Gordonia otitidis [other term]; Gordonia polyisoprenivorans [other term]; Gordonia sputi [other term]; Gordonia terrae [other term].

Drug Index Terms

amikacin / drug therapy; amoxicillin plus clavulanic acid / drug therapy; anastrozole / drug therapy; ceftazidime / drug therapy; ceftriaxone / drug therapy; cilastatin plus imipenem / drug therapy; ciprofloxacin / drug therapy / oral drug administration; cotrimoxazole / drug therapy / intravenous drug administration / oral drug administration; linezolid / drug therapy; minocycline / drug therapy / oral drug administration; tamoxifen / drug therapy; tigecycline / drug therapy; vancomycin / drug therapy.

Other Index Terms

Actinomycetales infection / drug therapy; adult; antibiotic sensitivity; anticoagulation; aorta valve disease; article; artificial heart pacemaker; *bacteremia / *drug therapy; bacteremia / drug therapy; bacterium identification; bacterium isolate; blood culture; breast

cancer / drug therapy / surgery; case report; catheter infection; central venous catheter; clinical feature; female; *Gordonia; heart atrium fibrillation; heart surgery; heart valve prosthesis; Hodgkin disease; human; human tissue; indwelling catheter; mastectomy; *nucleotide sequence; pleura biopsy; *pleura disease; pleura effusion; priority journal; spleen disease / surgery; splenectomy; video assisted thoracoscopic surgery.

CAS Registry Numbers

37517-28-5 (amikacin); 39831-55-5 (amikacin); 74469-00-4 (amoxicillin plus clavulanic acid); 79198-29-1 (amoxicillin plus clavulanic acid); 120511-73-1 (anastrozole); 72558-82-8 (ceftazidime); 73384-59-5 (ceftriaxone); 74578-69-1 (ceftriaxone); 92309-29-0 (cilastatin plus imipenem); 85721-33-1 (ciprofloxacin); 8064-90-2 (cotrimoxazole); 165800-03-3 (linezolid); 10118-90-8 (minocycline); 11006-27-2 (minocycline); 13614-98-7 (minocycline); 10540-29-1 (tamoxifen); 220620-09-7 (tigecycline); 1404-90-6 (vancomycin); 1404-93-9 (vancomycin)

Year of Publication

2011

Cite

74.

Gordonia araii **infection** associated with an orthopedic device and review of the literature on medical device-associated **Gordonia** infections.

Jannat-Khah D.P., Halsey E.S., Lasker B.A., Steigerwalt A.G., Hinrikson H.P., Brown J.M.

Embase
Journal of Clinical Microbiology. 47(2) (pp 499-502), 2009. Date of Publication: February 2009.

[Article]

AN:
354341613

Gordonia infections in humans are rare and usually affect immunocompromised patients. We present the first case of **Gordonia** araii **infection** associated with a medical device in an immunocompetent patient. Sequencing was required for conclusive identification. We compared our case to the 16 **Gordonia** species-associated medical device infections reported to date.

PMID

19109476 [https://www.ncbi.nlm.nih.gov/pubmed/?term=19109476]

Status

Embase

Institution

(Jannat-Khah, Lasker, Steigerwalt, Hinrikson, Brown) Division of Foodborne, Bacterial, and Mycotic Diseases, National Center for Zoonotic, Vector-Borne, and Enteric Diseases, Centers for Disease Control and Prevention, Atlanta, GA 30333, United States
(Halsey) Department of Infectious Diseases, Wright-Patterson Medical Center, Wright-Patterson Air Force Base, Dayton, OH 454332, United States
(Brown) Centers for Disease Control and Prevention, Mailstop G-34, Atlanta, GA 30333, United States

Publisher

American Society for Microbiology (1752 N Street N.W., Washington DC 20036-2904, United States)

Emtree Heading

adult; anterior cruciate ligament injury/su [Surgery]; antibiotic sensitivity; article; *bacterial infection/co [Complication]; bacterium culture; case report; fever; follow up; gene sequence; high performance liquid chromatography;
human
; infection/dt [Drug Therapy]; knee meniscus rupture/su [Surgery]; knee pain; leukocyte count; male; minimum inhibitory concentration; nucleotide sequence; orthopedic surgery; orthopedics; priority journal; amikacin; amoxicillin plus clavulanic acid; azithromycin; ceftriaxone; ciprofloxacin; clarithromycin; cotrimoxazole/dt [Drug Therapy]; cotrimoxazole/iv [Intravenous Drug Administration]; imipenem; levofloxacin/dt [Drug Therapy]; levofloxacin/iv [Intravenous Drug Administration]; linezolid; minocycline; vancomycin/dt [Drug Therapy]; vancomycin/iv [Intravenous Drug Administration]; *Gordonia araii infection/co [Complication].

Candidate Terms

*Gordonia araii infection / *complication [other term].

Drug Index Terms

amikacin; amoxicillin plus clavulanic acid; azithromycin; ceftriaxone; ciprofloxacin; clarithromycin; cotrimoxazole / drug therapy / intravenous drug administration; imipenem; levofloxacin / drug therapy / intravenous drug administration; linezolid; minocycline; vancomycin / drug therapy / intravenous drug administration.

Other Index Terms

adult; anterior cruciate ligament injury / surgery; antibiotic sensitivity; article; *bacterial infection / *complication; bacterium culture; case report; fever; follow up; gene sequence; high performance liquid chromatography; human; infection / drug therapy; knee meniscus rupture / surgery; knee pain; leukocyte count; male; minimum inhibitory concentration; nucleotide sequence; orthopedic surgery; orthopedics; priority journal.

CAS Registry Numbers

37517-28-5 (amikacin); 39831-55-5 (amikacin); 74469-00-4 (amoxicillin plus clavulanic acid); 79198-29-1 (amoxicillin plus clavulanic acid); 83905-01-5 (azithromycin); 73384-59-5 (ceftriaxone); 74578-69-1 (ceftriaxone); 85721-33-1 (ciprofloxacin); 81103-11-9 (clarithromycin); 8064-90-2 (cotrimoxazole); 64221-86-9 (imipenem); 100986-85-4 (levofloxacin); 138199-71-0 (levofloxacin); 165800-03-3 (linezolid); 10118-90-8 (minocycline); 11006-27-2 (minocycline); 13614-98-7 (minocycline); 1404-90-6 (vancomycin); 1404-93-9 (vancomycin)

Year of Publication

2009

Cite

75.

Characterization of clinical isolates of **Gordonia** species in Japanese clinical samples during 1998-2008.

Aoyama K., Kang Y., Yazawa K., Gonoi T., Kamei K., Mikami Y.

Embase
Mycopathologia. 168(4) (pp 175-183), 2009. Date of Publication: Oct 2009.

[Article]

AN:
355558974

During 1998-2008, there were 31 strains of **Gordonia** species isolated from clinical specimens in our laboratory. Our identification of the 31 strains of **Gordonia** species showed that major pathogenic **Gordonia** species in Japan were classifiable, respectively into 14 and 13 strains of **Gordonia** sputi and **Gordonia** bronchialis. The four remaining strains were identified as three **Gordonia** species: G. aichiensis (2 strains), and G. terrae (1 strain), and G. otitidis (1 strain). Results of drug susceptibility tests for these 31 strains of **Gordonia** isolates are reported herein.

PMID

19488833 [https://www.ncbi.nlm.nih.gov/pubmed/?term=19488833]

Institution

(Aoyama, Kang, Yazawa, Gonoi, Kamei, Mikami) Medical Mycology Research Center (MMRC), Chiba University, Chiba-city, Chiba, Japan.

Emtree Heading

*Actinomycetales infection; adult; aged; article; chemistry; classification; cluster analysis; DNA sequence; drug effect; female; genetics; *Gordonia;
human
; infant; isolation and purification; Japan; male; microbiological examination; microbiology; middle aged; molecular genetics; phylogeny; preschool child; antiinfective agent/pd [Pharmacology]; bacterial DNA; ribosome DNA; RNA 16S.

Drug Index Terms

antiinfective agent / pharmacology; bacterial DNA; ribosome DNA; RNA 16S.

Other Index Terms

*Actinomycetales infection; adult; aged; article; chemistry; classification; cluster analysis; DNA sequence; drug effect; female; genetics; *Gordonia; human; infant; isolation and purification; Japan; male; microbiological examination; microbiology; middle aged; molecular genetics; phylogeny; preschool child.

Year of Publication

2009

Cite

76.
Filamentous "contaminants" in the mycobacteriology laboratory; their culture, identification and clinical significance.
Vlaknite "kontaminanty" v mykobakteriologicke laboratori jejich kultivace, identifikace a klinicky vyznam
<Vlaknite "kontaminanty" v mykobakteriologicke laboratori jejich kultivace, identifikace a klinicky vyznam.>

Scharfen Jr. J., Buncek M., Jezek P., Urbaskova P., Fridrichova M., Kristufek V., Chronakova A.

Embase
Klinicka Mikrobiologie a Infekcni Lekarstvi. 16(2) (pp 48-57), 2010. Date of Publication: 2010.

[Review]

AN:
358995601

Frequent "contaminants" detected during mycobacterial culture of decontaminated samples are bacteria of the order Actinomycetales. These are usually bacteria classified as the family Corynebacterineae, genera Corynebacterium, Dietzia, **Gordonia**, Nocardia, Rhodococcus and Tsukamurella. These bacteria frequently colonize the airways and, under certain circumstances, they may cause life-threatening diseases. In severely immunocompromised patients, they regularly cause life-threatening infections with bacteria of the genus Nocardia. These filamentous bacteria, developing aerial mycelium in the culture, are partly acid-resistant and resistant to lysozy-me. They cause nocardiosis, a rare but serious disease in patients with various types of immune deficiency. Differential diagnosis must distinguish between the genera Streptomyces, Actinomadura and Nocardiopsis and other soil saprophytes that are not acid-resistant, sensitive to lysozyme and faster growing. They frequently colonize the airways of patients with lung disease but very rarely cause diseases. The diagnosis of aerobic actinomycetes and determination of their sensitivity to antibiotics are problematic since they grow longer, are difficult to stain and are involved in atypical biochemical reactions. Precise identification of the genera and species requires polyphasic identification of isolates using molecular microbiology methods. If diagnosed early, infections caused by aerobic actinomycetes are easy to treat with targeted antibiotic therapy.

PMID

20503156 [https://www.ncbi.nlm.nih.gov/pubmed/?term=20503156]

Status

Embase

Institution

(Scharfen Jr.) Narodni Referencni Laborator Pro Patogenni Aktinomycety, Odd. Lekarske Mikrobiologie a Imunologie, Oblastni Nemocnice Trutnov, a. s., M. Gorkeho 77, 541 21 Trutnov, Czechia (Buncek) GENERI BIOTECH, a. s., Hradec Kralove, Czechia
(Jezek) Oddeleni Klinicke Microbiologie, Nemocnice Pribram, Czechia
(Urbaskova, Fridrichova) Narodni Referencni Laborator Pro Antibiotika, Statni Zdravotni Ustav v Praze, Czechia
(Kristufek, Chronakova) Biologicke Centrum AV CR, v. v. i., Ustav Pudni Biologie, Ceske Budejovice, Czechia

Publisher

Trios spol. s.r.o. (Zakourilova 142, Praha 4 149 00, Czechia)

Keyword Heading

Contamination, Corynebacterium, Decontaminated samples, Diagnosis, Dietzia, Epidemiology, Gordonia, Identification, Mycobacteriology, Nocardia, Nocardiosis, Rhodococcus, Sensitivity tests, Streptomyces, Treatment, Tsukamurella

Emtree Heading

Actinomadura; antibiotic resistance; bacterial colonization; *bacterium contamination; *bacterium culture; bacterium identification;

Gordonia

; Gram staining;

human

; Mycobacterium; Nocardia; nocardiosis/dt [Drug Therapy]; nonhuman; review; Rhodococcus; species identification; Streptomyces; amikacin/cb [Drug Combination]; amikacin/dt [Drug Therapy]; cefotaxime/cb [Drug Combination]; cefotaxime/dt [Drug Therapy]; ceftriaxone/cb [Drug Combination]; ceftriaxone/dt [Drug Therapy]; cotrimoxazole/cb [Drug Combination]; cotrimoxazole/dt [Drug Therapy]; imipenem/cb [Drug Combination]; imipenem/dt [Drug Therapy]; meropenem/cb [Drug Combination]; meropenem/dt [Drug Therapy]; minocycline/dt [Drug Therapy].

Drug Index Terms

amikacin / drug combination / drug therapy; cefotaxime / drug combination / drug therapy; ceftriaxone / drug combination / drug therapy; cotrimoxazole / drug combination / drug therapy; imipenem / drug combination / drug therapy; meropenem / drug combination / drug therapy; minocycline / drug therapy.

Other Index Terms

Actinomadura; antibiotic resistance; bacterial colonization; *bacterium contamination; *bacterium culture; bacterium identification; Corynebacterium; Gordonia; Gram staining; human; Mycobacterium; Nocardia; nocardiosis / drug therapy; nonhuman; review; Rhodococcus; species identification; Streptomyces.

CAS Registry Numbers

37517-28-5 (amikacin); 39831-55-5 (amikacin); 63527-52-6 (cefotaxime); 64485-93-4 (cefotaxime); 73384-59-5 (ceftriaxone); 74578-69-1 (ceftriaxone); 8064-90-2 (cotrimoxazole); 64221-86-9 (imipenem); 96036-03-2 (meropenem); 10118-90-8 (minocycline); 11006-27-2 (minocycline); 13614-98-7 (minocycline)

Year of Publication

2010

Cite

77.

Investigation of an apparent outbreak of Rhodococcus equi bacteremia.

Langer A.J., Feja K., Lasker B.A., Hinrikson H.P., Morey R.E., Pellegrini G.J., Smith T.L., Robertson C.

Embase
Diagnostic Microbiology and Infectious Disease. 67(1) (pp 95-100), 2010. Date of Publication: May 2010.

[Article]

AN:
358704733

During January to April 2007, hospital staff reported 3 patients with Rhodococcus equi bloodstream infections. Isolates were analyzed at the Centers for Disease Control and Prevention, Atlanta, GA, to confirm identification and to assess strain relatedness; 2 were R. equi but genetically distinct, and 1 was identified as **Gordonia** polyisoprenivorans. Rapid reference laboratory support prevented an unnecessary outbreak investigation. © 2010.

PMID

20385352 [https://www.ncbi.nlm.nih.gov/pubmed/?term=20385352]

Status

Embase

Institution

(Langer, Robertson) New Jersey Department of Health and Senior Services, Trenton, NJ 08625, United States (Langer) Epidemic Intelligence Service, Centers for Disease Control and Prevention, Atlanta, GA 30333, United States
(Feja) Saint Peter's University Hospital, New Brunswick, NJ 08901, United States
(Feja) School of Public Health, University of Medicine and Dentistry of New Jersey, Piscataway, NJ 08854, United States
(Lasker, Hinrikson, Morey, Pellegrini, Smith) Bacterial Zoonoses Branch, Division of Foodborne, Bacterial, and Mycotic Diseases,

National Center for Zoonotic, Vector-Borne, and Enteric Diseases, Centers for Disease Control and Prevention, Atlanta, GA 30333, United States

Publisher

Elsevier Inc. (360 Park Avenue South, New York NY 10010, United States)

Keyword Heading

16S rRNA gene, Bacteremia, DNA Sequencing, MLST, Rhodococcus equi

Emtree Heading

acute granulocytic leukemia/dt [Drug Therapy]; acute lymphocytic leukemia/dt [Drug Therapy]; adolescent; article; *bacteremia/dt [Drug Therapy]; *bacteremia/et [Etiology]; bacteremia/dt [Drug Therapy]; bacterial infection/dt [Drug Therapy]; bacterial strain; bacterium identification; bacterium isolate; bloodstream infection; case report; catheter infection/dt [Drug Therapy]; child; drug withdrawal; female; Gordonia rubripertinctus; hospital personnel;

human

; infant; nucleotide sequence; preschool child; priority journal; public health service; *Rhodococcus equi; amikacin; amoxicillin plus clavulanic acid; ampicillin; antifungal agent/cb [Drug Combination]; antifungal agent/dt [Drug Therapy]; antiinfective agent/cb [Drug Combination]; antiinfective agent/dt [Drug Therapy]; antiinfective agent/pa [Parenteral Drug Administration]; asparaginase macrogol/cb [Drug Combination]; asparaginase macrogol/dt [Drug Therapy]; azithromycin/cb [Drug Combination]; azithromycin/dt [Drug Therapy]; azithromycin/po [Oral Drug Administration]; ceftazidime/cb [Drug Combination]; ceftriaxone/dt [Drug Therapy]; cefuroxime axetil/dt [Drug Therapy]; cefuroxime axetil/po [Oral Drug Administration]; ciprofloxacin/cb [Drug Combination]; ciprofloxacin/dt [Drug Therapy]; ciprofloxacin/iv [Intravenous Drug Administration]; ciprofloxacin/po [Oral Drug Administration]; clarithromycin; cotrimoxazole; cytarabine/cb [Drug Combination]; cytarabine/dt [Drug Therapy]; etoposide/cb [Drug Combination]; etoposide/dt [Drug Therapy]; gentamicin/cb [Drug Combination]; gentamicin/dt [Drug Therapy]; gentamicin/iv [Intravenous Drug Administration]; imipenem; linezolid; meropenem/cb [Drug Combination]; meropenem/dt [Drug Therapy]; methotrexate/cb [Drug Combination]; methotrexate/dt [Drug Therapy]; minocycline; rifampicin/cb [Drug Combination]; sulfamethoxazole; vancomycin/cb [Drug Combination]; vancomycin/dt [Drug Therapy]; vancomycin/iv [Intravenous Drug Administration]; vincristine/cb [Drug Combination]; vincristine/dt [Drug Therapy].

Drug Index Terms

amikacin; amoxicillin plus clavulanic acid; ampicillin; antifungal agent / drug combination / drug therapy; antiinfective agent / drug combination / drug therapy / parenteral drug administration; asparaginase macrogol / drug combination / drug therapy; azithromycin / drug combination / drug therapy / oral drug administration; ceftazidime / drug combination; ceftriaxone / drug therapy; cefuroxime axetil / drug therapy / oral drug administration; ciprofloxacin / drug combination / drug therapy / intravenous drug administration / oral drug administration; clarithromycin; cotrimoxazole; cytarabine / drug combination / drug therapy; etoposide / drug combination / drug therapy; gentamicin / drug combination / drug therapy / intravenous drug administration; imipenem; linezolid; meropenem / drug combination / drug therapy; methotrexate / drug combination / drug therapy; minocycline; rifampicin / drug combination; sulfamethoxazole; vancomycin / drug combination / drug therapy / intravenous drug administration; vincristine / drug combination / drug therapy.

Other Index Terms

acute granulocytic leukemia / drug therapy; acute lymphocytic leukemia / drug therapy; adolescent; article; *bacteremia / *drug therapy / *etiology; bacteremia / drug therapy; bacterial infection / drug therapy; bacterial strain; bacterium identification; bacterium isolate; bloodstream infection; case report; catheter infection / drug therapy; child; drug withdrawal; female; Gordonia rubripertinctus; hospital personnel; human; infant; nucleotide sequence; preschool child; priority journal; public health service; *Rhodococcus equi.

CAS Registry Numbers

37517-28-5 (amikacin); 39831-55-5 (amikacin); 74469-00-4 (amoxicillin plus clavulanic acid); 79198-29-1 (amoxicillin plus clavulanic acid); 69-52-3 (ampicillin); 69-53-4 (ampicillin); 7177-48-2 (ampicillin); 74083-13-9 (ampicillin); 94586-58-0 (ampicillin); 130167-69-0 (asparaginase macrogol); 83905-01-5 (azithromycin); 72558-82-8 (ceftazidime); 73384-59-5 (ceftriaxone); 74578-69-1 (ceftriaxone); 64544-07-6 (cefuroxime axetil); 85721-33-1 (ciprofloxacin); 81103-11-9 (clarithromycin); 8064-90-2 (cotrimoxazole); 147-94-4 (cytarabine); 69-74-9 (cytarabine); 33419-42-0 (etoposide); 1392-48-9 (gentamicin); 1403-66-3 (gentamicin); 1405-41-0 (gentamicin); 64221-86-9 (imipenem); 165800-03-3 (linezolid); 96036-03-2 (meropenem); 15475-56-6 (methotrexate); 59-05-2 (methotrexate); 7413-34-5 (methotrexate); 10118-90-8 (minocycline); 11006-27-2 (minocycline); 13614-98-7 (minocycline); 13292-46-1 (rifampicin); 723-46-6 (sulfamethoxazole); 1404-90-6 (vancomycin); 1404-93-9 (vancomycin); 57-22-7 (vincristine)

Year of Publication

2010

Cite

78.

A rubber-degrading organism growing from a **human** body.

Gupta M., Prasad D., Khara H.S., Alcid D.

Embase
International Journal of Infectious Diseases. 14(1) (pp e75-e76), 2010. Date of Publication: January 2010.

[Article]

AN:
50536992

Patients with hematological malignancies are susceptible to unusual infections, because of the use of broad-spectrum anti-infective agents, invasive procedures, and other immunocompromising procedures and medications. **Gordonia** polyisoprenivorans, a ubiquitous environmental aerobic actinomycete belonging to the family of Gordoniaceae in the order Actinomycetales, is a very rare cause of bacteremia in these patients. We report the first case of pneumonia with associated bacteremia due to this organism, which was initially described in 1999 as a rubber-degrading bacterium following isolation from stagnant water inside a deteriorated automobile tire. We believe that hematologically immunocompromised patients on broad-spectrum antibiotics and with long-term central catheters select the possibility of **infection** with G. polyisoprenivorans. These infections can be prevented by handling catheters under aseptic conditions. We propose that blood cultures of persistently febrile neutropenic patients should be incubated for at least 4 weeks. Being a rare **infection**, there are no data available on treatment other than early removal of the foreign bodies. © 2009 International Society for Infectious Diseases.

PMID

19501006 [https://www.ncbi.nlm.nih.gov/pubmed/?term=19501006]

Status

Embase

Institution

(Gupta, Prasad, Khara, Alcid) Department of Internal Medicine, Drexel University College, Medicine - Saint Peter's University Hospital, 254 Easton Avenue, New Brunswick, NJ 08901, United States

Publisher

Elsevier (P.O. Box 211, Amsterdam 1000 AE, Netherlands)

Keyword Heading

Bacteremia, Gordonia polyisoprenivorans, Leukemia, Pneumonia, Rubber-degrading organism

Emtree Heading

Actinobacteria; adolescent; antibiotic therapy; article; *bacteremia/et [Etiology]; blood culture; case report; *catheter infection; febrile neutropenia; female; foreign body;
Gordonia
; hematologic malignancy;
human
; immune deficiency; infection sensitivity; pneumonia/et [Etiology]; rubber; *Gordonia polyisoprenivorans.

Candidate Terms

*Gordonia polyisoprenivorans [other term].

Drug Index Terms

rubber.

Other Index Terms

Actinobacteria; adolescent; antibiotic therapy; article; *bacteremia / *etiology; blood culture; case report; *catheter infection; febrile neutropenia; female; foreign body; Gordonia; hematologic malignancy; human; immune deficiency; infection sensitivity; pneumonia / etiology.

CAS Registry Numbers

9006-04-6 (rubber)

Year of Publication

2010

Cite

79.
Infections caused by **Gordonia** species at a medical centre in Taiwan, 1997 to 2008.

Lai C.C., Wang C.Y., Liu C.Y., Tan C.K., Lin S.H., Liao C.H., Chou C.H., Huang Y.T., Lin H.I., Hsueh P.R.

Embase
Clinical Microbiology and Infection. 16(9) (pp 1448-1453), 2010. Date of Publication: September 2010.

[Article]

AN:
359444765

The inability of conventional identification systems to accurately identify **Gordonia** spp. often results in the misdiagnosis of infections by these rare pathogens, which require genomic sequencing for precise identification. In the present study, we describe nine cases of the various types of **infection** caused by **Gordonia** spp. From 1997 to 2008, 66 isolates (from 30 patients) initially identified as Rhodoccus spp. by conventional biochemical methods, by the Bacteriology Laboratory of National Taiwan University Hospital, were retrospectively analysed to assess the accuracy of species identification. Fifteen of these isolates (from nine patients) were later found to be **Gordonia** spp. by two molecular methods: PCR-restriction fragment length polymorphism for heat shock protein gene (hsp65) and the 16S rRNA gene sequencing analysis. **Gordonia** sputi (n = 8) was the most common species, followed by **Gordonia** terrae (n = 7). Most of the isolates were isolated from blood (n = 11), followed by soft tissue (n = 2) and eye (n = 2). Five patients presented with bacteraemia and two of these had catheter-related bloodstream **infection.** Two patients had soft tissue infections and another two patients had infective keratitis and conjunctivitis. The random amplified polymorphic DNA patterns for isolates from different patients were different, indicating that they were genetically unrelated. Accurate identification with molecular methods is required if the role of **Gordonia** spp. in causing **infection** is to be recognized. © 2010 The Authors. Journal Compilation © 2010 European Society of Clinical Microbiology and Infectious Diseases.

PMID

19832703 [https://www.ncbi.nlm.nih.gov/pubmed/?term=19832703]

Status

Embase

Institution

(Lai, Wang, Lin) Department of Internal Medicine, Cardinal Tien Hospital, Taipei County, Taiwan (Republic of China) (Liu, Liao) Department of Internal Medicine, Far Eastern Memorial Hospital, Taipei, Taiwan (Republic of China)
(Tan) Department of Intensive Care Medicine, Chi-Mei Medical Center, Tainan, Taiwan (Republic of China)
(Lin) Department of Internal Medicine, Taipei County Hospital, Taipei County, Taiwan (Republic of China)
(Chou) Department of Internal Medicine, National Taiwan University Hospital Yun-Lin Branch, Yun-Lin, Taiwan (Republic of China)
(Huang, Hsueh) Departments of Laboratory Medicine and Internal Medicine, National Taiwan University Hospital, National Taiwan University College of Medicine, Taipei, Taiwan (Republic of China)

Publisher

Blackwell Publishing Ltd (9600 Garsington Road, Oxford OX4 2XG, United Kingdom)

Keyword Heading

Bacteraemia, Catheter related infection, Gordonia spp., Keratitis, Molecular diagnosis

Emtree Heading

accuracy; Actinomycetales infection/dt [Drug Therapy]; *Actinomycetales infection/di [Diagnosis]; *Actinomycetales infection/dt [Drug Therapy]; adolescent; adult; aged; antibiotic sensitivity; article; bacteremia/dt [Drug Therapy]; bacterium identification; bacterium isolate; blood culture; catheter infection/co [Complication]; catheter infection/dt [Drug Therapy]; catheter removal; cellulitis/dt [Drug Therapy]; clinical article; conjunctivitis/dt [Drug Therapy]; controlled study; debridement; female; *Gordonia;
human
; keratitis/dt [Drug Therapy]; male; minimum inhibitory concentration; newborn; nonhuman; nucleotide sequence; polymerase chain reaction; priority journal; random amplified polymorphic DNA; restriction fragment length polymorphism; retrospective study; Rhodococcus; sequence analysis; soft tissue infection; Taiwan; wound infection/co [Complication]; wound infection/dt [Drug Therapy];

wound infection/su [Surgery]; amikacin/cb [Drug Combination]; amikacin/dt [Drug Therapy]; amoxicillin plus clavulanic acid/dt [Drug Therapy]; ampicillin; antibiotic agent/dt [Drug Therapy]; antibiotic agent/tp [Topical Drug Administration]; bacterial DNA; cefoxitin/dt [Drug Therapy]; chloramphenicol; ciprofloxacin/cb [Drug Combination]; ciprofloxacin/dt [Drug Therapy]; ciprofloxacin/iv [Intravenous Drug Administration]; clindamycin; cotrimoxazole; dalfopristin plus quinupristin; erythromycin; genomic DNA; gentamicin; heat shock protein 65/ec [Endogenous Compound]; imipenem/cb [Drug Combination]; imipenem/dt [Drug Therapy]; levofloxacin; linezolid; moxifloxacin; nitrofurantoin; oxacillin; penicillin G; rifampicin; RNA 16S/ec [Endogenous Compound]; streptomycin; sulfamethoxazole/dt [Drug Therapy]; sulfamethoxazole/tp [Topical Drug Administration]; teicoplanin; tetracycline; unindexed drug; vancomycin/cb [Drug Combination]; vancomycin/dt [Drug Therapy]; gordonia sputi; gordonia terrae.

Candidate Terms

Gordonia sputi [other term]; Gordonia terrae [other term].

Drug Index Terms

amikacin / drug combination / drug therapy; amoxicillin plus clavulanic acid / drug therapy; ampicillin; antibiotic agent / drug therapy / topical drug administration; bacterial DNA; cefoxitin / drug therapy; chloramphenicol; ciprofloxacin / drug combination / drug therapy / intravenous drug administration; clindamycin; cotrimoxazole; dalfopristin plus quinupristin; erythromycin; genomic DNA; gentamicin; heat shock protein 65 / endogenous compound; imipenem / drug combination / drug therapy; levofloxacin; linezolid; moxifloxacin; nitrofurantoin; oxacillin; penicillin G; rifampicin; RNA 16S / endogenous compound; streptomycin; sulfamethoxazole / drug therapy / topical drug administration; teicoplanin; tetracycline; unindexed drug; vancomycin / drug combination / drug therapy.

Other Index Terms

accuracy; Actinomycetales infection / drug therapy; *Actinomycetales infection / *diagnosis / *drug therapy; adolescent; adult; aged; antibiotic sensitivity; article; bacteremia / drug therapy; bacterium identification; bacterium isolate; blood culture; catheter infection / complication / drug therapy; catheter removal; cellulitis / drug therapy; clinical article; conjunctivitis / drug therapy; controlled study; debridement; female; *Gordonia; human; keratitis / drug therapy; male; minimum inhibitory concentration; newborn; nonhuman; nucleotide sequence; polymerase chain reaction; priority journal; random amplified polymorphic DNA; restriction fragment length polymorphism; retrospective study; Rhodococcus; sequence analysis; soft tissue infection; Taiwan; wound infection / complication / drug therapy / surgery.

CAS Registry Numbers

37517-28-5 (amikacin); 39831-55-5 (amikacin); 74469-00-4 (amoxicillin plus clavulanic acid); 79198-29-1 (amoxicillin plus clavulanic acid); 69-52-3 (ampicillin); 69-53-4 (ampicillin); 7177-48-2 (ampicillin); 74083-13-9 (ampicillin); 94586-58-0 (ampicillin); 33564-30-6 (cefoxitin); 35607-66-0 (cefoxitin); 134-90-7 (chloramphenicol); 2787-09-9 (chloramphenicol); 56-75-7 (chloramphenicol); 85721-33-1 (ciprofloxacin); 18323-44-9 (clindamycin); 8064-90-2 (cotrimoxazole); 126602-89-9 (dalfopristin plus quinupristin); 114-07-8 (erythromycin); 70536-18-4 (erythromycin); 1392-48-9 (gentamicin); 1403-66-3 (gentamicin); 1405-41-0 (gentamicin); 64221-86-9 (imipenem); 100986-85-4 (levofloxacin); 138199-71-0 (levofloxacin); 165800-03-3 (linezolid); 151096-09-2 (moxifloxacin); 54-87-5 (nitrofurantoin); 67-20-9 (nitrofurantoin); 1173-88-2 (oxacillin); 66-79-5 (oxacillin); 7240-38-2 (oxacillin); 1406-05-9 (penicillin G); 61-33-6 (penicillin G); 13292-46-1 (rifampicin); 57-92-1 (streptomycin); 723-46-6 (sulfamethoxazole); 61036-62-2 (teicoplanin); 61036-64-4 (teicoplanin); 23843-90-5 (tetracycline); 60-54-8 (tetracycline); 64-75-5 (tetracycline); 8021-86-1 (tetracycline); 1404-90-6 (vancomycin); 1404-93-9 (vancomycin)

Year of Publication

2010

Cite

80.

Gordonia araii pneumonia: Case report and review of treatment options.

Erlandson K.M., Madinger N.E.

Embase
Infectious Diseases in Clinical Practice. 18(6) (pp 367-373), 2010. Date of Publication: November 2010.

[Review]

AN:
51040982

Gordonia are a rare cause of **human** disease and are most commonly reported as causative agents of bacteremia in immunocompromised patients. We describe the second case of pulmonary **infection** due to **Gordonia** araii and review all previously published cases of **Gordonia infection** where clinical information including treatment regimens and outcome are provided. Fifty-one reported cases of **Gordonia infection** include treatment regimen and clinical outcome. Most cases are catheter-related bloodstream infections or wound infections. Of the 5 cases of **Gordonia** spp. with pulmonary

involvement, 2 individuals died and the other 3 individuals required a mean of 4 months of multidrug therapy. Based on limited case reports, and in contrast to catheter-associated bacteremia and abscesses amenable to surgical drainage, pulmonary involvement with **Gordonia** appears to be associated with aggressive, necrotizing pneumonia, requires prolonged multidrug therapy, and may require surgical resection for optimal outcome. Copyright © 2010 by Lippincott Williams & Wilkins.

Status

Embase

Institution

(Erlandson, Madinger) University of Colorado Denver, Mail Stop B168, 12700 E 19th Ave, Aurora, CO 80045, United States

Publisher

Lippincott Williams and Wilkins (530 Walnut Street,P O Box 327, Philadelphia PA 19106-3621, United States)

Keyword Heading

Actinomycetales, Gordonia, Gordonia araii

Emtree Heading

adult; antibiotic resistance; antibiotic sensitivity; *antibiotic therapy; bacteremia; bacterial pneumonia/dt [Drug Therapy]; *bacterial pneumonia/dt [Drug Therapy]; *bacterial pneumonia/et [Etiology]; *bacterial pneumonia/su [Surgery]; bacterium culture; bacterium isolate; bronchoscopy; case report; catheter infection; cause of death; drug substitution; *Gordonia;
human
; human tissue; lung abscess/su [Surgery]; male; nonhuman; outcome assessment; physical examination; polypharmacy; review; Rhodococcus; surgical drainage; thorax radiography; transbronchial biopsy; treatment duration; treatment response; wound infection; amikacin; amoxicillin plus clavulanic acid; azithromycin/cb [Drug Combination]; azithromycin/dt [Drug Therapy]; ceftriaxone/cb [Drug Combination]; ceftriaxone/dt [Drug Therapy]; ceftriaxone/iv [Intravenous Drug Administration]; ciprofloxacin/dt [Drug Therapy]; ciprofloxacin/po [Oral Drug Administration]; clarithromycin; imipenem; linezolid; meropenem/cb [Drug Combination]; meropenem/dt [Drug Therapy]; metronidazole/cb [Drug Combination]; metronidazole/dt [Drug Therapy]; minocycline/dt [Drug Therapy]; piperacillin plus tazobactam/cb [Drug Combination]; piperacillin plus tazobactam/dt [Drug Therapy]; sulfadoxine plus trimethoprim; tobramycin; vancomycin/cb [Drug Combination]; vancomycin/dt [Drug Therapy]; *Gordonia araii.

Candidate Terms

*Gordonia araii [other term].

Drug Index Terms

amikacin; amoxicillin plus clavulanic acid; azithromycin / drug combination / drug therapy; ceftriaxone / drug combination / drug therapy / intravenous drug administration; ciprofloxacin / drug therapy / oral drug administration; clarithromycin; imipenem; linezolid; meropenem / drug combination / drug therapy; metronidazole / drug combination / drug therapy; minocycline / drug therapy; piperacillin plus tazobactam / drug combination / drug therapy; sulfadoxine plus trimethoprim; tobramycin; vancomycin / drug combination / drug therapy.

Other Index Terms

adult; antibiotic resistance; antibiotic sensitivity; *antibiotic therapy; bacteremia; bacterial pneumonia / drug therapy; *bacterial pneumonia / *drug therapy / *etiology / *surgery; bacterium culture; bacterium isolate; bronchoscopy; case report; catheter infection; cause of death; drug substitution; *Gordonia; human; human tissue; lung abscess / surgery; male; nonhuman; outcome assessment; physical examination; polypharmacy; review; Rhodococcus; surgical drainage; thorax radiography; transbronchial biopsy; treatment duration; treatment response; wound infection.

CAS Registry Numbers

37517-28-5 (amikacin); 39831-55-5 (amikacin); 74469-00-4 (amoxicillin plus clavulanic acid); 79198-29-1 (amoxicillin plus clavulanic acid); 83905-01-5 (azithromycin); 73384-59-5 (ceftriaxone); 74578-69-1 (ceftriaxone); 85721-33-1 (ciprofloxacin); 81103-11-9 (clarithromycin); 64221-86-9 (imipenem); 165800-03-3 (linezolid); 96036-03-2 (meropenem); 39322-38-8 (metronidazole); 443-48-1 (metronidazole); 10118-90-8 (minocycline); 11006-27-2 (minocycline); 13614-98-7 (minocycline); 39295-60-8 (sulfadoxine plus trimethoprim); 32986-56-4 (tobramycin); 1404-90-6 (vancomycin); 1404-93-9 (vancomycin)

Year of Publication

2010

Cite

81.

Five cases of bacteraemia due to **Gordonia** species.

Brust J.C.M., Whittier S., Scully B.E., McGregor C.C., Yin M.T.

Embase
Journal of Medical Microbiology. 58(10) (pp 1376-1378), 2009. Date of Publication: October 2009.

[Article]

AN:
355314442

Gordonia species are aerobic Gram-positive bacilli and a rare cause of **human** disease. To our knowledge, there are only two cases of **human infection** with **Gordonia** sputi reported in the literature. We report five cases of bacteraemia due to **Gordonia** species at our institution since 2005, including four caused by G. sputi. Three of these cases were likely related to chronic indwelling central venous catheters. © 2009 SGM.

PMID

19528153 [https://www.ncbi.nlm.nih.gov/pubmed/?term=19528153]

Status

Embase

Institution

(Brust) Divisions of General Internal Medicine and Infectious Diseases, Montefiore Medical Center, Albert Einstein College of Medicine, Bronx, NY, United States (Whittier) Department of Microbiology, College of Physicians and Surgeons, Columbia University, New York, NY, United States
(Scully, Yin) Division of Infectious Diseases, College of Physicians and Surgeons, Columbia University, New York, NY, United States
(McGregor) Division of Pulmonary, Allergy, and Critical Care Medicine, College of Physicians and Surgeons, Columbia University, New York, NY, United States

Publisher

Society for General Microbiology (Basingstoke Road, Spencers Wood, Reading, Berkshire RG7 1AE, United Kingdom)

Emtree Heading

adult; aged; anamnesis; antibiotic resistance; antibiotic sensitivity; antibiotic therapy; article; aspiration pneumonia/dt [Drug Therapy]; bacteremia/dt [Drug Therapy]; *bacteremia/dt [Drug Therapy]; bronchoscopy; catheter infection/co [Complication]; catheter infection/dt [Drug Therapy]; catheter removal; central venous catheter; chronic obstructive lung disease/dt [Drug Therapy]; clinical article; Clostridium difficile infection/dt [Drug Therapy]; computed tomographic angiography; computer assisted tomography; controlled study; disease course; female; *Gordonia;
human
; indwelling catheter; laboratory test; male; priority journal; transthoracic echocardiography; amikacin/dt [Drug Therapy]; erythromycin; gentamicin; imipenem/dt [Drug Therapy]; levofloxacin/dt [Drug Therapy]; linezolid/dt [Drug Therapy]; linezolid/po [Oral Drug Administration]; metronidazole/dt [Drug Therapy]; minocycline/dt [Drug Therapy]; piperacillin plus tazobactam/dt [Drug Therapy]; steroid/dt [Drug Therapy]; steroid/po [Oral Drug Administration]; tobramycin/dt [Drug Therapy]; vancomycin/dt [Drug Therapy]; *Gordonia bronchialis; *gordonia sputi.

Candidate Terms

*Gordonia bronchialis [other term]; *Gordonia sputi [other term].

Drug Index Terms

amikacin / drug therapy; erythromycin; gentamicin; imipenem / drug therapy; levofloxacin / drug therapy; linezolid / drug therapy / oral drug administration; metronidazole / drug therapy; minocycline / drug therapy; piperacillin plus tazobactam / drug therapy; steroid / drug therapy / oral drug administration; tobramycin / drug therapy; vancomycin / drug therapy.

Other Index Terms

adult; aged; anamnesis; antibiotic resistance; antibiotic sensitivity; antibiotic therapy; article; aspiration pneumonia / drug therapy; bacteremia / drug therapy; *bacteremia / *drug therapy; bronchoscopy; catheter infection / complication / drug therapy; catheter removal; central venous catheter; chronic obstructive lung disease / drug therapy; clinical article; Clostridium difficile infection / drug

therapy; computed tomographic angiography; computer assisted tomography; controlled study; disease course; female; *Gordonia; human; indwelling catheter; laboratory test; male; priority journal; transthoracic echocardiography.

CAS Registry Numbers

37517-28-5 (amikacin); 39831-55-5 (amikacin); 114-07-8 (erythromycin); 70536-18-4 (erythromycin); 1392-48-9 (gentamicin); 1403-66-3 (gentamicin); 1405-41-0 (gentamicin); 64221-86-9 (imipenem); 100986-85-4 (levofloxacin); 138199-71-0 (levofloxacin); 165800-03-3 (linezolid); 39322-38-8 (metronidazole); 443-48-1 (metronidazole); 10118-90-8 (minocycline); 11006-27-2 (minocycline); 13614-98-7 (minocycline); 32986-56-4 (tobramycin); 1404-90-6 (vancomycin); 1404-93-9 (vancomycin)

Year of Publication

2009

Cite

82.
Gordonia sputi bacteremia.

Renvoise A., Harle J.-R., Raoult D., Roux V.

Embase
Emerging Infectious Diseases. 15(9) (pp 1535-1537), 2009. Date of Publication: September 2009.

[Letter]

AN:
355257475

PMID

19788838 [https://www.ncbi.nlm.nih.gov/pubmed/?term=19788838]

Status

Embase

Institution

(Renvoise, Raoult, Roux) Laboratoire de Bacteriologie-Virologie, Hopital de la Timone, IFR48, 264 rue Saint-Pierre, 13385 Marseille, Cedex 05, France (Harle) Hopital de La Conception, Marseille, France

Publisher

Centers for Disease Control and Prevention (CDC) (1600 Clifton Road, Mailstop C-12, Atlanta GA 30333, United States)

Emtree Heading

aged; bacteremia/dt [Drug Therapy]; *bacteremia/di [Diagnosis]; *bacteremia/dt [Drug Therapy]; blood sampling; case report; clinical feature; comorbidity; *Gordonia;
human
; laboratory test; letter; male; nucleotide sequence; ciprofloxacin/dt [Drug Therapy]; ciprofloxacin/po [Oral Drug Administration]; granulocyte colony stimulating factor receptor/dt [Drug Therapy]; granulocyte colony stimulating factor receptor/po [Oral Drug Administration]; timentin/dt [Drug Therapy]; timentin/po [Oral Drug Administration]; *gordonia sputi.

Candidate Terms

*Gordonia sputi [other term].

Drug Index Terms

ciprofloxacin / drug therapy / oral drug administration; granulocyte colony stimulating factor receptor / drug therapy / oral drug administration; timentin / drug therapy / oral drug administration.

Other Index Terms

aged; bacteremia / drug therapy; *bacteremia / *diagnosis / *drug therapy; blood sampling; case report; clinical feature; comorbidity; *Gordonia; human; laboratory test; letter; male; nucleotide sequence.

CAS Registry Numbers

85721-33-1 (ciprofloxacin); 86482-18-0 (timentin)

Year of Publication

2009

Cite

83.

Mycobacterium Fortuitum and Anaerobic Breast Abscess Following Nipple Piercing: Case Presentation and Review of the Literature.

Bengualid V., Singh V., Singh H., Berger J.

Embase
Journal of Adolescent Health. 42(5) (pp 530-532), 2008. Date of Publication: May 2008.

[Article]

AN:
50057858

Purpose: Body piercing has become increasingly prevalent. We describe a case of breast **infection** with combined mycobacteria and anaerobe following nipple piercing, and review the literature. Case: A 17-year-old female developed a breast abscess 4 months after nipple piercing. Cultures grew Prevotalla melangenica and Mycobacterium fortuitum. She required drainage and antibiotic treatment. Three months into her treatment she stopped her medications, relapsed, and required drainage. Two months later, on antimycobacteria therapy, her wound is healing.
Discussion(s): Review of the infectious complications of nipple piercing yielded 12 cases, 5 of which had a foreign body. The pathogens isolated (coagulase negative staphylococcus, mycobacteria, streptococcus, anaerobe, and **gordonia**) are not the usual organisms to be isolated from a breast abscess. This could result from reporting bias or the presence of a foreign body, the nipple ring. The three cases of mycobacteria, in addition to ours, are reviewed. The average age is 22 years. Three to 9 months elapsed between piercing and **infection**. All cases required drainage. Antimycobacteria therapy was used in three of the four cases for 10 days to 6 months.
Conclusion(s): With the increasing prevalence of body piercing, it is important to document and report infections. We describe a breast abscess following nipple piercing with combined anaerobic and a mycobacterial pathogens. This underscores the need for obtaining cultures including anaerobes and mycobacteria. © 2008 Society for Adolescent Medicine.

PMID

18407050 [https://www.ncbi.nlm.nih.gov/pubmed/?term=18407050]

Status

Embase

Institution

(Bengualid, Berger) St. Barnabas Hospital, Department of Infectious Diseases, Bronx, NY, United States (Singh) St. Barnabas Hospital, Department of Internal Medicine, Bronx, NY, United States
(Singh) St. Barnabas Hospital, Department of Family Medicine, Bronx, NY, United States

Publisher

Elsevier USA

Keyword Heading

Breast abscess, Heading, Infection, Nipple pearcing

Emtree Heading

abscess drainage; adolescent; anaerobic infection/dt [Drug Therapy]; anaerobic infection/et [Etiology]; anaerobic infection/su [Surgery]; antibiotic therapy; article; *body piercing; *breast abscess/dt [Drug Therapy]; *breast abscess/et [Etiology]; *breast abscess/su [Surgery]; case report; female;
human
; *Mycobacterium fortuitum; nipple; Prevotella melaninogenica; priority journal; relapse; treatment duration; wound healing; amoxicillin plus clavulanic acid/dt [Drug Therapy]; cefazolin/dt [Drug Therapy]; ciprofloxacin/dt [Drug Therapy]; clarithromycin/dt [Drug Therapy]; metronidazole/dt [Drug Therapy]; baterium culture.

Candidate Terms

baterium culture [other term].

Drug Index Terms

amoxicillin plus clavulanic acid / drug therapy; cefazolin / drug therapy; ciprofloxacin / drug therapy; clarithromycin / drug therapy; metronidazole / drug therapy.

Other Index Terms

abscess drainage; adolescent; anaerobic infection / drug therapy / etiology / surgery; antibiotic therapy; article; *body piercing; *breast abscess / *drug therapy / *etiology / *surgery; case report; female; human; *Mycobacterium fortuitum; nipple; Prevotella melaninogenica; priority journal; relapse; treatment duration; wound healing.

CAS Registry Numbers

74469-00-4 (amoxicillin plus clavulanic acid); 79198-29-1 (amoxicillin plus clavulanic acid); 25953-19-9 (cefazolin); 27164-46-1 (cefazolin); 85721-33-1 (ciprofloxacin); 81103-11-9 (clarithromycin); 39322-38-8 (metronidazole); 443-48-1 (metronidazole)

Year of Publication

2008

Cite

84.

Gordonia species: Emerging pathogens in pediatric patients that are identified by 16S ribosomal RNA gene sequencing.

Blaschke A.J., Bender J., Byington C.L., Korgenski K., Daly J., Petti C.A., Pavia A.T., Ampofo K.

Embase
Clinical Infectious Diseases. 45(4) (pp 483-486), 2007. Date of Publication: 15 Aug 2007.

[Article]

AN:
47204467

Gordonia species are emerging pathogens that are often misidentified as Rhodococcus or Nocardia species but are reliably distinguished by 16S ribosomal RNA gene sequencing. We present a case series of 6 episodes of catheter-associated **infection** caused by **Gordonia** species in 5 patients seen at a tertiary care pediatric hospital and describe the management and outcomes of this **infection** in adults and children. © 2007 by the Infectious Diseases Society of America. All rights reserved.

PMID

17638199 [https://www.ncbi.nlm.nih.gov/pubmed/?term=17638199]

Status

Embase

Institution

(Blaschke, Bender, Byington, Pavia, Ampofo) Department of Pediatrics, Division of Pediatric Infectious Diseases, University of Utah, Salt Lake City, UT, United States (Daly) Department of Pathology, University of Utah, Salt Lake City, UT, United States
(Petti) Department of Medicine, University of Utah, Salt Lake City, UT, United States
(Korgenski, Daly) Primary Children's Medical Center Microbiology Laboratory, Salt Lake City, UT, United States
(Petti) Associated Regional and University Pathologists Laboratories, Salt Lake City, UT, United States
(Blaschke) Dept. of Pediatrics, Division of Pediatric Infectious Diseases, University of Utah, 2A100 SOM, 30 N. 1900 East, Salt lake City, UT, United States

Publisher

Oxford University Press (2001 Evans Road, Cary NC 27513, United States)

Emtree Heading

article; bacterium identification; bacterium isolate; catheter infection/dt [Drug Therapy]; catheter infection/et [Etiology]; child; clinical article; *Gordonia;
human

; infant; Nocardia; nonhuman; nucleotide sequence; outcome assessment; priority journal; reliability; Rhodococcus; *RNA sequence; tertiary health care; amikacin/cb [Drug Combination]; amikacin/dt [Drug Therapy]; ampicillin; azithromycin; ceftriaxone/cb [Drug Combination]; ceftriaxone/dt [Drug Therapy]; ciprofloxacin/dt [Drug Therapy]; clarithromycin/cb [Drug Combination]; clarithromycin/dt [Drug Therapy]; cotrimoxazole/cb [Drug Combination]; cotrimoxazole/dt [Drug Therapy]; doxycycline; gatifloxacin; gentamicin/cb [Drug Combination]; gentamicin/dt [Drug Therapy]; imipenem/cb [Drug Combination]; imipenem/dt [Drug Therapy]; linezolid; meropenem/cb [Drug Combination]; meropenem/dt [Drug Therapy]; penicillin G; rifampicin/cb [Drug Combination]; rifampicin/dt [Drug Therapy]; *RNA 16S/ec [Endogenous Compound]; vancomycin/cb [Drug Combination]; vancomycin/dt [Drug Therapy]; Gordonia bronchialis; Gordonia otitidis; gordonia terrae.

Candidate Terms

Gordonia bronchialis [other term]; Gordonia otitidis [other term]; Gordonia terrae [other term].

Drug Index Terms

amikacin / drug combination / drug therapy; ampicillin; azithromycin; ceftriaxone / drug combination / drug therapy; ciprofloxacin / drug therapy; clarithromycin / drug combination / drug therapy; cotrimoxazole / drug combination / drug therapy; doxycycline; gatifloxacin; gentamicin / drug combination / drug therapy; imipenem / drug combination / drug therapy; linezolid; meropenem / drug combination / drug therapy; penicillin G; rifampicin / drug combination / drug therapy; *RNA 16S / *endogenous compound; vancomycin / drug combination / drug therapy.

Other Index Terms

article; bacterium identification; bacterium isolate; catheter infection / drug therapy / etiology; child; clinical article; *Gordonia; human; infant; Nocardia; nonhuman; nucleotide sequence; outcome assessment; priority journal; reliability; Rhodococcus; *RNA sequence; tertiary health care.

CAS Registry Numbers

37517-28-5 (amikacin); 39831-55-5 (amikacin); 69-52-3 (ampicillin); 69-53-4 (ampicillin); 7177-48-2 (ampicillin); 74083-13-9 (ampicillin); 94586-58-0 (ampicillin); 83905-01-5 (azithromycin); 73384-59-5 (ceftriaxone); 74578-69-1 (ceftriaxone); 85721-33-1 (ciprofloxacin); 81103-11-9 (clarithromycin); 8064-90-2 (cotrimoxazole); 10592-13-9 (doxycycline); 17086-28-1 (doxycycline); 564-25-0 (doxycycline); 112811-59-3 (gatifloxacin); 180200-66-2 (gatifloxacin); 1392-48-9 (gentamicin); 1403-66-3 (gentamicin); 1405-41-0 (gentamicin); 64221-86-9 (imipenem); 165800-03-3 (linezolid); 96036-03-2 (meropenem); 1406-05-9 (penicillin G); 61-33-6 (penicillin G); 13292-46-1 (rifampicin); 1404-90-6 (vancomycin); 1404-93-9 (vancomycin)

Year of Publication

2007

Cite

85.

Endophthalmitis due to Williamsia muralis.

Murray R.J., Aravena-Roman M., Kampfer P.

Embase
Journal of Medical Microbiology. 56(10) (pp 1410-1412), 2007. Date of Publication: October 2007.

[Article]

AN:
350105614

A case of endophthalmitis caused by Williamsia muralis is described. The **infection** occurred following a procedure known as intravitreal triamcinolone acetonide injection for the treatment of diabetic maculopathy. This is the first report of W. muralis as a causative agent of endophthalmitis. © 2007 SGM.

PMID

17893183 [https://www.ncbi.nlm.nih.gov/pubmed/?term=17893183]

Status

Embase

Institution

(Murray) Department of Microbiology and Infectious Diseases, PathWest Laboratory Services, Royal Perth Hospital, Wellington St, Perth, WA 6000, Australia (Aravena-Roman) Division of Microbiology and Infectious Diseases, PathWest Laboratory Services, Queen Elizabeth II Medical Centre, Hospital Avenue, Nedlands, WA 6009, Australia
(Kampfer) Institut fur Angewandte Mikrobiologie, Justus-Liebig Universitat Giessen, D-35392 Giessen, Germany

Publisher

Society for General Microbiology (Basingstoke Road, Spencers Wood, Reading, Berkshire RG7 1AE, United Kingdom)

Emtree Heading

*Actinomyces; aged; anamnesis; article; bacterial growth; bacterial strain; bacterium colony; case report; clinical feature; diabetes mellitus/dt [Drug Therapy]; DNA hybridization; electron microscopy; endophthalmitis/si [Side Effect]; *endophthalmitis/di [Diagnosis]; *endophthalmitis/dt [Drug Therapy]; *endophthalmitis/et [Etiology]; *endophthalmitis/si [Side Effect];
Gordonia
; Gram staining;
human
; human tissue; hydrolysis; incubation time; inoculation; male; nucleotide sequence; physical examination; priority journal; retina maculopathy/dt [Drug Therapy]; RNA sequence; slit lamp; treatment outcome; vitreous body; agar; alkaline phosphatase; alpha glucosidase; bacterial DNA; catalase; ceftazidime/iv [Intravenous Drug Administration]; ciprofloxacin; penicillin G; RNA 16S; triamcinolone acetonide/ae [Adverse Drug Reaction]; triamcinolone acetonide/dt [Drug Therapy]; vancomycin/dt [Drug Therapy]; vancomycin/vi [Intravitreal Drug Administration]; *williamsia muralis.

Candidate Terms

*Williamsia muralis [other term].

Drug Index Terms

agar; alkaline phosphatase; alpha glucosidase; bacterial DNA; catalase; ceftazidime / intravenous drug administration; ciprofloxacin; penicillin G; RNA 16S; triamcinolone acetonide / adverse drug reaction / drug therapy; vancomycin / drug therapy / intravitreal drug administration.

Other Index Terms

*Actinomyces; aged; anamnesis; article; bacterial growth; bacterial strain; bacterium colony; case report; clinical feature; diabetes mellitus / drug therapy; DNA hybridization; electron microscopy; endophthalmitis / side effect; *endophthalmitis / *diagnosis / *drug therapy / *etiology / *side effect; Gordonia; Gram staining; human; human tissue; hydrolysis; incubation time; inoculation; male; nucleotide sequence; physical examination; priority journal; retina maculopathy / drug therapy; RNA sequence; slit lamp; treatment outcome; vitreous body.

CAS Registry Numbers

9002-18-0 (agar); 9001-78-9 (alkaline phosphatase); 9001-42-7 (alpha glucosidase); 9001-05-2 (catalase); 72558-82-8 (ceftazidime); 85721-33-1 (ciprofloxacin); 1406-05-9 (penicillin G); 61-33-6 (penicillin G); 76-25-5 (triamcinolone acetonide); 1404-90-6 (vancomycin); 1404-93-9 (vancomycin)

Year of Publication

2007

Cite

86.
Improved outcome of Trypanosoma cruzi **infection** in rats following treatment in early life with suspensions of heat-killed environmental Actinomycetales.

Fontanella G.H., Pascutti M.F., Daurelio L., Perez A.R., Nocito A.L., Wojdyla D., Bottasso O., Revelli S.S., Stanford J.L.

Embase
Vaccine. 25(17) (pp 3492-3500), 2007. Date of Publication: 30 Apr 2007.

[Article]

AN:
46527982

The well-established model of Chagas' disease in "l" rats was used to evaluate the effects of three injections of heat-killed **Gordonia** bronchialis, Rhodococcus coprophilus or saline on Trypanosoma cruzi parasitaemia and acute and chronic myocarditis, sequelae of the **infection**. Two vaccinating injections were given prior to challenge with T. cruzi, and the third, immunotherapeutic, injection was given 7 days after challenge. Treatment with either actinomycete significantly

reduced acute parasitaemia ($p < 0.04$), modified cellular infiltration during acute myocarditis and limited chronic myocarditis ($p < 0.03$) in comparison with the saline-treated control animals. Immunological investigations showed that both bacterial preparations achieved their results through different mechanisms. The relevance of our findings to **human** Chagas' disease is discussed. © 2006 Elsevier Ltd. All rights reserved.

PMID

17368877 [https://www.ncbi.nlm.nih.gov/pubmed/?term=17368877]

Status

Embase

Institution

(Fontanella, Pascutti, Daurelio, Perez, Nocito, Wojdyla, Bottasso, Revelli) Instituto de Inmunologia, Facultad de Ciencias Medicas, Universidad Nacional de Rosario, Santa Fe 3100, 2000 Rosario, Santa Fe, Argentina (Stanford) Centre for Infectious Diseases and International Health, Windeyer Institute of Medical Sciences, University College London, 46 Cleveland Street, London, United Kingdom

Publisher

Elsevier Ltd (Langford Lane, Kidlington, Oxford OX5 1GB, United Kingdom)

Keyword Heading

Actinomycetales, Immunomodulation, Trypanosoma cruzi

Emtree Heading

*Actinomycetales; acute disease; animal experiment; animal model; animal tissue; article; cell infiltration; chronic disease; controlled study; *Gordonia; immunotherapy; male; myocarditis; nonhuman; outcome assessment; *parasitemia/pc [Prevention]; *parasitemia/th [Therapy]; priority journal; rat; *Rhodococcus; statistical analysis; treatment duration; *Trypanosoma cruzi; sodium chloride; *Gordonia bronchialis; *rhodococcus coprophilus.

Candidate Terms

*Gordonia bronchialis [other term]; *rhodococcus coprophilus [other term].

Drug Index Terms

sodium chloride.

Other Index Terms

*Actinomycetales; acute disease; animal experiment; animal model; animal tissue; article; cell infiltration; chronic disease; controlled study; *Gordonia; immunotherapy; male; myocarditis; nonhuman; outcome assessment; *parasitemia / *prevention / *therapy; priority journal; rat; *Rhodococcus; statistical analysis; treatment duration; *Trypanosoma cruzi.

CAS Registry Numbers

7647-14-5 (sodium chloride)

Year of Publication

2007

Cite

87.

Isolation of **Gordonia** terrae from a patient with catheter-related bacteraemia.

Grisold A.J., Roll P., Hoenigl M., Feierl G., Vicenzi-Moser R., Marth E.

Embase
Journal of Medical Microbiology. 56(12) (pp 1687-1688), 2007. Date of Publication: December 2007.

[Article]

AN:
350276521

A cornyeform bacterium was isolated from a blood culture from a 24-year-old man with familial hypertrophic non-obstructive cardiomyopathy, chronic abuse of anabolic steroids and prior admission to hospital because of clinical signs of sepsis. 16S rRNA gene analysis unambiguously identified **Gordonia** terrae. © 2007 SGM.

PMID

18033840 [https://www.ncbi.nlm.nih.gov/pubmed/?term=18033840]

Status

Embase

Institution

(Grisold, Hoenigl, Feierl, Marth) Institute of Hygiene, Medical University of Graz, A-8010 Graz, Austria (Roll) Institute for Forensic Medicine, Medical University of Graz, A-8010 Graz, Austria
(Vicenzi-Moser) Department of Anaesthesiology and Intensive Care Medicine, Medical University of Graz, A-8036 Graz, Austria

Publisher

Society for General Microbiology (Basingstoke Road, Spencers Wood, Reading, Berkshire RG7 1AE, United Kingdom)

Emtree Heading

adult; article; *bacteremia/dt [Drug Therapy]; *bacteremia/et [Etiology]; bacterium identification; bacterium isolation; blood culture; case report; *catheter infection/dt [Drug Therapy]; *catheter infection/et [Etiology]; drug abuse; drug sensitivity; familial hypertrophic cardiomyopathy; genetic analysis;
Gordonia
; hospital admission;
human
; male; nucleotide sequence; priority journal; sepsis; amikacin; amoxicillin plus clavulanic acid; anabolic agent; ceftazidime; ceftriaxone; ciprofloxacin; clindamycin; gentamicin; imipenem; levofloxacin/dt [Drug Therapy]; meropenem; metandienone; moxifloxacin; oxacillin; penicillin G; piperacillin plus tazobactam/dt [Drug Therapy]; RNA 16S; vancomycin.

Drug Index Terms

amikacin; amoxicillin plus clavulanic acid; anabolic agent; ceftazidime; ceftriaxone; ciprofloxacin; clindamycin; gentamicin; imipenem; levofloxacin / drug therapy; meropenem; metandienone; moxifloxacin; oxacillin; penicillin G; piperacillin plus tazobactam / drug therapy; RNA 16S; vancomycin.

Other Index Terms

adult; article; *bacteremia / *drug therapy / *etiology; bacterium identification; bacterium isolation; blood culture; case report; *catheter infection / *drug therapy / *etiology; drug abuse; drug sensitivity; familial hypertrophic cardiomyopathy; genetic analysis; Gordonia; hospital admission; human; male; nucleotide sequence; priority journal; sepsis.

CAS Registry Numbers

37517-28-5 (amikacin); 39831-55-5 (amikacin); 74469-00-4 (amoxicillin plus clavulanic acid); 79198-29-1 (amoxicillin plus clavulanic acid); 72558-82-8 (ceftazidime); 73384-59-5 (ceftriaxone); 74578-69-1 (ceftriaxone); 85721-33-1 (ciprofloxacin); 18323-44-9 (clindamycin); 1392-48-9 (gentamicin); 1403-66-3 (gentamicin); 1405-41-0 (gentamicin); 64221-86-9 (imipenem); 100986-85-4 (levofloxacin); 138199-71-0 (levofloxacin); 96036-03-2 (meropenem); 72-63-9 (metandienone); 151096-09-2 (moxifloxacin); 1173-88-2 (oxacillin); 66-79-5 (oxacillin); 7240-38-2 (oxacillin); 1406-05-9 (penicillin G); 61-33-6 (penicillin G); 1404-90-6 (vancomycin); 1404-93-9 (vancomycin)

Year of Publication

2007

Cite

88.
Characterization of clinical isolates of pathogenic Nocardia strains and related actinomycetes in Thailand from 1996 to 2003.

Poonwan N., Mekha N., Yazawa K., Thunyaharn S., Yamanaka A., Mikami Y.

Embase
Mycopathologia. 159(3) (pp 361-368), 2005. Date of Publication: April 2005.

[Article]

AN:

40747830

In Thailand from 1996 to 2003, 171 strains of pathogenic aerobic actinomycetes from clinical specimens were isolated. Of those strains, 134 were mycolic acid containing actinomycetes, including 96 strains of Nocardia species. Others included 10 strains of **Gordonia**, 14 strains of Rhodococcus, and 22 strains of Mycobacterium. One strain each of the genera Tsukamurella and Corynebacterium were also isolated. Also identified were 27 strains of non-mycolic acid containing actinomycetes. Our identification studies of 96 strains of Nocardia species showed that significant pathogens in Thailand were N. beijingensis (18 strains), N. cyriacigeorgica (13 strains), and N. farcinica (34 strains); the most prevalent species was N. farcinica (35.4%). We also isolated four strains of N. asiatica, five strains of N. asteroides sensu stricto, four strains of N. nova, seven strains of N. otitidiscaviarum, eight strains of N. transvalensis, and two strains of N. pseudobrasiliensis. © Springer 2005.

PMID

15883719 [https://www.ncbi.nlm.nih.gov/pubmed/?term=15883719]

Institution

(Poonwan, Mekha) National Institute of Health, Department of Medical Sciences, Ministry of Public Health, Nonthaburi, 11000, Thailand (Yazawa, Mikami) Research Center for Pathogenic Fungi and Microbial Toxicoses, Chiba University, Chuo-ku, Chiba, 260-8673, Japan
(Thunyaharn) Department of Clinical Pathology, Pramongkutklao Army Hospital, Bangkok, Thailand
(Yamanaka) Department of Medical Science, State University of Campinas, Sao Paulo, Brazil
(Mikami) Research Center for Pathogenic Fungi and Microbial Toxicoses, Chiba University, 1-8-1, Inohana, Chuo-ku, Chibu (260-8673), Japan

Publisher

Springer Netherlands (Van Godewijckstraat 30, Dordrecht 3311 GZ, Netherlands)

Keyword Heading

Clinical sample, Isolation and identification, Nocardia, Pathogenic actinomycetes, Thailand

Emtree Heading

*Actinobacteria; article; bacterial infection/ep [Epidemiology]; comparative study; genetics;

human

; isolation and purification; microbiology; molecular genetics; *Nocardia; *nucleotide sequence; pathogenicity; respiratory tract infection/ep [Epidemiology]; species difference; Thailand/ep [Epidemiology]; mycolic acid/an [Drug Analysis].

Drug Index Terms

mycolic acid / drug analysis.

Other Index Terms

*Actinobacteria; article; bacterial infection / epidemiology; comparative study; genetics; human; isolation and purification; microbiology; molecular genetics; *Nocardia; *nucleotide sequence; pathogenicity; respiratory tract infection / epidemiology; species difference; Thailand / epidemiology.

CAS Registry Numbers

37281-34-8 (mycolic acid)

Year of Publication

2005

Cite

89.

Etiological misidentification by routine biochemical tests of bacteremia caused by **Gordonia** terrae **infection** in the course

of an episode of acute cholecystitis.

Gil-Sande E., Brun-Otero M., Campo-Cerecedo F., Esteban E., Aguilar L., Garcia-De-Lomas J.

Embase

Journal of Clinical Microbiology. 44(7) (pp 2645-2647), 2006. Date of Publication: July 2006.

[Article]

AN:
44092451

Gordonia terrae has been reported to be a rare cause of bacteremia. We report the first case of bacteremia associated with acute cholecystitis. Commercial biochemical testing was not able to identify the strain at the genus level, classifying it instead as Rhodococcus sp. Definitive identification was obtained by sequencing of the 16S rRNA gene. Copyright © 2006, American Society for Microbiology. All Rights Reserved.

PMID

16825404 [https://www.ncbi.nlm.nih.gov/pubmed/?term=16825404]

Status

Embase

Institution

(Gil-Sande) Microbiology Department, Hospital San Rafael, Las Jubias 82, 15006 A Coruna, Spain (Brun-Otero) Internal Medicine, Hospital San Rafael, Las Jubias 82, 15006 A Coruna, Spain
(Campo-Cerecedo) Pathology Department, Hospital San Rafael, Las Jubias 82, 15006 A Coruna, Spain
(Esteban, Aguilar, Garcia-De-Lomas) Instituto Valenciano de Microbiologia, Masia El Romeral, Ctra. Betera a San Antonio Km. 0.3, 46117 Betera, Valencia, Spain

Publisher

American Society for Microbiology (1752 N Street N.W., Washington DC 20036-2904, United States)

Emtree Heading

*acute cholecystitis/di [Diagnosis]; *acute cholecystitis/dt [Drug Therapy]; *acute cholecystitis/et [Etiology]; *acute cholecystitis/su [Surgery]; adult; antibiotic sensitivity; article; *bacteremia/co [Complication]; *bacteremia/di [Diagnosis]; *bacteremia/dt [Drug Therapy]; *bacteremia/et [Etiology]; bacterial strain; bacterium culture; bacterium identification; biochemistry; case report; cholecystectomy; cholelithiasis; fever; *Gordonia; hepatitis C;
human
; hypertension; male; minimum inhibitory concentration; nucleotide sequence; priority journal; Rhodococcus; RNA sequence; cefotaxime; gentamicin/cb [Drug Combination]; gentamicin/dt [Drug Therapy]; gentamicin/iv [Intravenous Drug Administration]; penicillin G; piperacillin plus tazobactam/cb [Drug Combination]; piperacillin plus tazobactam/dt [Drug Therapy]; piperacillin plus tazobactam/iv [Intravenous Drug Administration]; RNA 16S/ec [Endogenous Compound]; vancomycin; *gordonia terrae.

Candidate Terms

*Gordonia terrae [other term].

Drug Index Terms

cefotaxime; gentamicin / drug combination / drug therapy / intravenous drug administration; penicillin G; piperacillin plus tazobactam / drug combination / drug therapy / intravenous drug administration; RNA 16S / endogenous compound; vancomycin.

Other Index Terms

*acute cholecystitis / *diagnosis / *drug therapy / *etiology / *surgery; adult; antibiotic sensitivity; article; *bacteremia / *complication / *diagnosis / *drug therapy / *etiology; bacterial strain; bacterium culture; bacterium identification; biochemistry; case report; cholecystectomy; cholelithiasis; fever; *Gordonia; hepatitis C; human; hypertension; male; minimum inhibitory concentration; nucleotide sequence; priority journal; Rhodococcus; RNA sequence.

CAS Registry Numbers

63527-52-6 (cefotaxime); 64485-93-4 (cefotaxime); 1392-48-9 (gentamicin); 1403-66-3 (gentamicin); 1405-41-0 (gentamicin); 1406-05-9 (penicillin G); 61-33-6 (penicillin G); 1404-90-6 (vancomycin); 1404-93-9 (vancomycin)

Year of Publication

2006

Cite

90.
Native valve endocarditis due to **Gordonia** polyisoprenivorans: Case report and review of literature of bloodstream infections caused by **Gordonia** species.

Verma P., Brown J.M., Nunez V.H., Morey R.E., Steigerwalt A.G., Pellegrini G.J., Kessler H.A.

Embase
Journal of Clinical Microbiology. 44(5) (pp 1905-1908), 2006. Date of Publication: May 2006.

[Review]

AN:
43726299

We report the first case of endocarditis caused by Gordzonia polyisoprenivorans and concisely review the English literature regarding bloodstream infections caused by **Gordonia** species. Copyright © 2006, American Society for Microbiology. All Rights Reserved.

PMID

16672437 [https://www.ncbi.nlm.nih.gov/pubmed/?term=16672437]

Status

Embase

Institution

(Verma) Division of Clinical Microbiology, Rush Medical College, Rush University Medical Center, Chicago, IL 60612, United States
(Verma) Department of Pathology, Rush Medical College, Rush University Medical Center, Chicago, IL 60612, United States
(Verma, Nunez, Kessler) Department of Internal Medicine (Section of Infectious Diseases), Rush Medical College, Rush University Medical Center, Chicago, IL 60612, United States
(Brown, Morey, Steigerwalt, Pellegrini) Division of Bacterial and Mycotic Diseases, National Center for Infectious Diseases, Centers for Disease Control and Prevention, Atlanta, GA 30333, United States
(Verma) Department of Pathology and Clinical Laboratories, Virginia Mason Medical Center, Mail Stop C6-LAB, 1100 Ninth Avenue, Seattle, WA 98101, United States

Publisher

American Society for Microbiology (1752 N Street N.W., Washington DC 20036-2904, United States)

Emtree Heading

aged; *bacterial endocarditis/dt [Drug Therapy]; blood culture; case report; clinical feature; disease course; *Gordonia;
human
; male; minimum inhibitory concentration; nonhuman; *nucleotide sequence; priority journal; review; sepsis; amikacin; amoxicillin plus clavulanic acid; ampicillin/cb [Drug Combination]; ampicillin/dt [Drug Therapy]; ampicillin/iv [Intravenous Drug Administration]; ceftriaxone; cilastatin plus imipenem/dt [Drug Therapy]; cilastatin plus imipenem/iv [Intravenous Drug Administration]; ciprofloxacin; clarithromycin; cotrimoxazole; gentamicin/cb [Drug Combination]; gentamicin/dt [Drug Therapy]; gentamicin/iv [Intravenous Drug Administration]; imipenem; levofloxacin/cb [Drug Combination]; levofloxacin/dt [Drug Therapy]; linezolid; minocycline; vancomycin/cb [Drug Combination]; vancomycin/dt [Drug Therapy]; *Gordonia polyisoprenivorans.

Candidate Terms

*Gordonia polyisoprenivorans [other term].

Drug Index Terms

amikacin; amoxicillin plus clavulanic acid; ampicillin / drug combination / drug therapy / intravenous drug administration; ceftriaxone; cilastatin plus imipenem / drug therapy / intravenous drug administration; ciprofloxacin; clarithromycin; cotrimoxazole; gentamicin / drug combination / drug therapy / intravenous drug administration; imipenem; levofloxacin / drug combination / drug therapy; linezolid; minocycline; vancomycin / drug combination / drug therapy.

Other Index Terms

aged; *bacterial endocarditis / *drug therapy; blood culture; case report; clinical feature; disease course; *Gordonia; human; male; minimum inhibitory concentration; nonhuman; *nucleotide sequence; priority journal; review; sepsis.

CAS Registry Numbers

37517-28-5 (amikacin); 39831-55-5 (amikacin); 74469-00-4 (amoxicillin plus clavulanic acid); 79198-29-1 (amoxicillin plus clavulanic acid); 69-52-3 (ampicillin); 69-53-4 (ampicillin); 7177-48-2 (ampicillin); 74083-13-9 (ampicillin); 94586-58-0 (ampicillin); 73384-59-5 (ceftriaxone); 74578-69-1 (ceftriaxone); 92309-29-0 (cilastatin plus imipenem); 85721-33-1 (ciprofloxacin); 81103-11-9 (clarithromycin); 8064-90-2 (cotrimoxazole); 1392-48-9 (gentamicin); 1403-66-3 (gentamicin); 1405-41-0 (gentamicin); 64221-86-9 (imipenem); 100986-85-4 (levofloxacin); 138199-71-0 (levofloxacin); 165800-03-3 (linezolid); 10118-90-8 (minocycline); 11006-27-2 (minocycline); 13614-98-7 (minocycline); 1404-90-6 (vancomycin); 1404-93-9 (vancomycin)

Year of Publication

2006

Cite

91.

Recurrent breast abscess caused by **Gordonia** bronchialis in an immunocompetent patient.

Werno A.M., Anderson T.P., Chambers S.T., Laird H.M., Murdoch D.R.

Embase
Journal of Clinical Microbiology. 43(6) (pp 3009-3010), 2005. Date of Publication: June 2005.

[Article]

AN:
40828002

We present the first reported case of a recurrent breast **infection** caused by **Gordonia** bronchialis. The **infection** occurred in a 43-year-old immunocompetent female, and species level identification was obtained with 16S rRNA sequencing. Copyright © 2005, American Society for Microbiology. All Rights Reserved.

PMID

15956447 [https://www.ncbi.nlm.nih.gov/pubmed/?term=15956447]

Status

Embase

Institution

(Werno, Anderson, Laird, Murdoch) Microbiology Unit, Canterbury Health Laboratories, Christchurch, New Zealand (Chambers) Department of Infectious Diseases, Christchurch Hospital, Christchurch, New Zealand
(Chambers, Murdoch) Department of Pathology, Christchurch School of Medicine and Health Sciences, Christchurch, New Zealand
(Murdoch) Canterbury Health Laboratories, P.O. Box 151, Christchurch, New Zealand

Publisher

American Society for Microbiology (1752 N Street N.W., Washington DC 20036-2904, United States)

Emtree Heading

abscess drainage; adult; anamnesis; article; bacterial growth; bacterium identification; bacterium isolate; *breast abscess/di [Diagnosis]; *breast abscess/dt [Drug Therapy]; *breast abscess/et [Etiology]; *breast abscess/su [Surgery]; case report; clinical feature; echography; female; *Gordonia; Gram staining;
human
; human tissue; immunocompetence; laboratory test; mammography; microscopy; morphology; nucleotide sequence; physical examination; priority journal; *recurrent disease/di [Diagnosis]; *recurrent disease/dt [Drug Therapy]; *recurrent disease/et [Etiology]; *recurrent disease/su [Surgery]; Rhodococcus; RNA sequence; Staphylococcus; amoxicillin plus clavulanic acid/dt [Drug Therapy]; amoxicillin plus clavulanic acid/po [Oral Drug Administration]; ceftriaxone; ciprofloxacin; clindamycin/dt [Drug Therapy]; clindamycin/po [Oral Drug Administration]; doxycycline/dt [Drug Therapy]; doxycycline/po [Oral Drug Administration]; erythromycin; flucloxacillin/ad [Drug Administration]; flucloxacillin/cb [Drug Combination]; flucloxacillin/iv [Intravenous Drug Administration]; metronidazole/cb [Drug Combination]; metronidazole/dt [Drug Therapy]; penicillin G/dt [Drug Therapy]; penicillin G/iv [Intravenous Drug Administration]; *RNA 16S/ec [Endogenous Compound]; tetracycline; vancomycin; *Gordonia bronchialis.

Candidate Terms

*Gordonia bronchialis [other term].

Drug Index Terms

amoxicillin plus clavulanic acid / drug therapy / oral drug administration; ceftriaxone; ciprofloxacin; clindamycin / drug therapy / oral drug administration; doxycycline / drug therapy / oral drug administration; erythromycin; flucloxacillin / drug administration / drug combination / intravenous drug administration; metronidazole / drug combination / drug therapy; penicillin G / drug therapy / intravenous drug administration; *RNA 16S / *endogenous compound; tetracycline; vancomycin.

Other Index Terms

abscess drainage; adult; anamnesis; article; bacterial growth; bacterium identification; bacterium isolate; *breast abscess / *diagnosis / *drug therapy / *etiology / *surgery; case report; clinical feature; echography; female; *Gordonia; Gram staining; human; human tissue; immunocompetence; laboratory test; mammography; microscopy; morphology; nucleotide sequence; physical examination; priority journal; *recurrent disease / *diagnosis / *drug therapy / *etiology / *surgery; Rhodococcus; RNA sequence; Staphylococcus.

CAS Registry Numbers

74469-00-4 (amoxicillin plus clavulanic acid); 79198-29-1 (amoxicillin plus clavulanic acid); 73384-59-5 (ceftriaxone); 74578-69-1 (ceftriaxone); 85721-33-1 (ciprofloxacin); 18323-44-9 (clindamycin); 10592-13-9 (doxycycline); 17086-28-1 (doxycycline); 564-25-0 (doxycycline); 114-07-8 (erythromycin); 70536-18-4 (erythromycin); 1847-24-1 (flucloxacillin); 5250-39-5 (flucloxacillin); 39322-38-8 (metronidazole); 443-48-1 (metronidazole); 1406-05-9 (penicillin G); 61-33-6 (penicillin G); 23843-90-5 (tetracycline); 60-54-8 (tetracycline); 64-75-5 (tetracycline); 1404-90-6 (vancomycin); 1404-93-9 (vancomycin)

Year of Publication

2005

Cite

92.

Gordonia polyisoprenivorans septicemia in a bone marrow transplant patient.

Kempf V.A.J., Schmalzing M., Yassin A.F., Schaal K.P., Baumeister D., Arenskotter M., Steinbuchel A., Autenrieth I.B.

Embase
European Journal of Clinical Microbiology and Infectious Diseases. 23(3) (pp 226-228), 2004. Date of Publication: March 2004.

[Article]

AN:
38471435

PMID

14760540 [https://www.ncbi.nlm.nih.gov/pubmed/?term=14760540]

Status

Embase

Institution

(Kempf, Autenrieth) Inst. Med. Mikrobiol. K., Eberhard-Karls-Universitat, Elfriede-Aulhorn Strasse 6, 72076 Tubingen, Germany
(Schmalzing) Department of Internal Medicine, Eberhard-Karls-Universitat, Otfried-Muller-Strasse 10, 72076 Tubingen, Germany
(Yassin, Schaal) Inst. Med. Mikrobiol. und Immunol., Friedrich-Wilhelms-Universitat, Sigmund-Freud-Strasse 25, 53105 Bonn, Germany
(Baumeister, Arenskotter, Steinbuchel) Inst. Molec. Mikrobiol. und Biotech., Westfalische Wilhems-Univ., Corrensstrasse 3, 48149 Munster, Germany

Publisher

Springer Verlag (Tiergartenstrasse 17, Heidelberg D-69121, Germany)

Emtree Heading

adult; allogenic bone marrow transplantation; antibiotic therapy; article; bacterium identification; *bone marrow transplantation; case report; catheter infection/co [Complication]; catheter infection/dt [Drug Therapy]; catheter infection/et [Etiology]; chemotaxonomy; female;
human
; minimum inhibitory concentration; nucleotide sequence; priority journal; *septicemia/co [Complication]; *septicemia/dt [Drug Therapy]; *septicemia/et [Etiology]; sequence alignment; amoxicillin/cb [Drug Combination]; amoxicillin/dt [Drug Therapy];

amoxicillin/po [Oral Drug Administration]; amoxicillin plus clavulanic acid; ciprofloxacin/cb [Drug Combination]; ciprofloxacin/dt [Drug Therapy]; clindamycin; gentamicin; penicillin G; piperacillin plus tazobactam/cb [Drug Combination]; piperacillin plus tazobactam/dt [Drug Therapy]; vancomycin.

Drug Index Terms

amoxicillin / drug combination / drug therapy / oral drug administration; amoxicillin plus clavulanic acid; ciprofloxacin / drug combination / drug therapy; clindamycin; gentamicin; penicillin G; piperacillin plus tazobactam / drug combination / drug therapy; vancomycin.

Other Index Terms

adult; allogenic bone marrow transplantation; antibiotic therapy; article; bacterium identification; *bone marrow transplantation; case report; catheter infection / complication / drug therapy / etiology; chemotaxonomy; female; human; minimum inhibitory concentration; nucleotide sequence; priority journal; *septicemia / *complication / *drug therapy / *etiology; sequence alignment.

CAS Registry Numbers

26787-78-0 (amoxicillin); 34642-77-8 (amoxicillin); 61336-70-7 (amoxicillin); 74469-00-4 (amoxicillin plus clavulanic acid); 79198-29-1 (amoxicillin plus clavulanic acid); 85721-33-1 (ciprofloxacin); 18323-44-9 (clindamycin); 1392-48-9 (gentamicin); 1403-66-3 (gentamicin); 1405-41-0 (gentamicin); 1406-05-9 (penicillin G); 61-33-6 (penicillin G); 1404-90-6 (vancomycin); 1404-93-9 (vancomycin)

Year of Publication

2004

Cite

93.

Bacteremia caused by **Gordonia** bronchialis in a patient with sequestrated lung.

Sng L.-H., Koh T.H., Toney S.R., Floyd M., Butler W.R., Tan B.H.

Embase
Journal of Clinical Microbiology. 42(6) (pp 2870-2871), 2004. Date of Publication: June 2004.

[Article]

AN:
38747563

Gordonia species have been recognized as pathogens in immunocompromised and immunocompetent patients. We report the first case of bacteremia due to **Gordonia** bronchialis in a diabetic patient with a sequestrated lung. Species identification was confirmed with mycolic acid analysis by high-performance liquid chromatography and sequencing of the 16S rRNA gene.

PMID

15184495 [https://www.ncbi.nlm.nih.gov/pubmed/?term=15184495]

Status

Embase

Institution

(Sng, Koh) Department of Pathology, Singapore General Hospital, 169608 Singapore, Singapore (Tan) Division of Internal Medicine, Singapore General Hospital, 169608 Singapore, Singapore
(Toney, Floyd, Butler) Div. of AIDS, STD and TB Lab. Res., Natl. Ctr. for HIV, STD and TB Prev., Ctr. for Dis. Contr. and Prevention, Atlanta, GA 30333, United States
(Sng) Department of Pathology, Singapore General Hospital, 1 Hospital Dr., 169608 Singapore, Singapore

Publisher

American Society for Microbiology (1752 N Street N.W., Washington DC 20036-2904, United States)

Emtree Heading

abscess/di [Diagnosis]; abscess/dt [Drug Therapy]; abscess/su [Surgery]; adult; article; *bacteremia/di [Diagnosis]; *bacteremia/dt [Drug Therapy]; *bacteremia/et [Etiology]; blood culture; case report; computer assisted tomography; diabetes mellitus; female; gene

human

; leukocyte count; lung cyst/su [Surgery]; *lung sequestration/di [Diagnosis]; minimum inhibitory concentration; nuclear magnetic resonance imaging; nucleotide sequence; physical examination; pleura effusion/su [Surgery]; pneumonia/dt [Drug Therapy]; priority journal; RNA sequence; species identification; thorax radiography; amoxicillin plus clavulanic acid/dt [Drug Therapy]; amoxicillin plus clavulanic acid/po [Oral Drug Administration]; ampicillin; bacterial DNA; ceftazidime; ceftriaxone/cb [Drug Combination]; ceftriaxone/dt [Drug Therapy]; clarithromycin/cb [Drug Combination]; clarithromycin/dt [Drug Therapy]; cloxacillin/dt [Drug Therapy]; ester; mycolic acid; ribosome RNA; vancomycin/cb [Drug Combination]; vancomycin/dt [Drug Therapy]; vancomycin/iv [Intravenous Drug Administration]; *Gordonia bronchialis.

Candidate Terms

*Gordonia bronchialis [other term].

Drug Index Terms

amoxicillin plus clavulanic acid / drug therapy / oral drug administration; ampicillin; bacterial DNA; ceftazidime; ceftriaxone / drug combination / drug therapy; clarithromycin / drug combination / drug therapy; cloxacillin / drug therapy; ester; mycolic acid; ribosome RNA; vancomycin / drug combination / drug therapy / intravenous drug administration.

Other Index Terms

abscess / diagnosis / drug therapy / surgery; adult; article; *bacteremia / *diagnosis / *drug therapy / *etiology; blood culture; case report; computer assisted tomography; diabetes mellitus; female; gene sequence; *Gordonia; high performance liquid chromatography; human; leukocyte count; lung cyst / surgery; *lung sequestration / *diagnosis; minimum inhibitory concentration; nuclear magnetic resonance imaging; nucleotide sequence; physical examination; pleura effusion / surgery; pneumonia / drug therapy; priority journal; RNA sequence; species identification; thorax radiography.

CAS Registry Numbers

74469-00-4 (amoxicillin plus clavulanic acid); 79198-29-1 (amoxicillin plus clavulanic acid); 69-52-3 (ampicillin); 69-53-4 (ampicillin); 7177-48-2 (ampicillin); 74083-13-9 (ampicillin); 94586-58-0 (ampicillin); 72558-82-8 (ceftazidime); 73384-59-5 (ceftriaxone); 74578-69-1 (ceftriaxone); 81103-11-9 (clarithromycin); 61-72-3 (cloxacillin); 642-78-4 (cloxacillin); 37281-34-8 (mycolic acid); 1404-90-6 (vancomycin); 1404-93-9 (vancomycin)

Year of Publication

2004

Cite

94.

Gordonia terrae-induced suppurative granulomatous mastitis following nipple piercing [3].

Zardawi I.M., Jones F., Clark D.A., Holland J.

Embase
Pathology. 36(3) (pp 275-278), 2004. Date of Publication: June 2004.

[Letter]

AN:
38748092

PMID

15203738 [https://www.ncbi.nlm.nih.gov/pubmed/?term=15203738]

Status

Embase

Institution

(Zardawi) Anatomical Pathology, Hunter Area Pathology Service, John Hunter Hospital, New Lambton, NSW, Australia (Jones, Clark) Breast Centre, Christo Road Hospital, Waratah, NSW, Australia
(Holland) Mayne Health Laverty Pathology, North Ryde, NSW, Australia

Publisher

Lippincott Williams and Wilkins (345 Hudson St., 16th Fl., New York NY 10014-4502, United States)

Emtree Heading

*breast abscess/et [Etiology]; cytology;
Gordonia
; granulomatous inflammation; histology;
human
; letter; *mastitis/et [Etiology].

Other Index Terms

*breast abscess / *etiology; cytology; Gordonia; granulomatous inflammation; histology; human; letter; *mastitis / *etiology.

Year of Publication

2004

Cite

95.

Catheter-related bacteremia caused by the nocardioform actinomycete **Gordonia** terrae.

Pham A.S., De I., Rolston K.V., Tarrand J.J., Han X.Y.

Embase
Clinical Infectious Diseases. 36(4) (pp 524-527), 2003. Date of Publication: 15 Feb 2003.

[Article]

AN:
36241323

Five cases of catheter-related bacteremia caused by **Gordonia** terrae are reported. All patients who also had the primary diagnosis of cancer experienced nonneutropenic fever as a result of G. terrae **infection**. All patients were treated successfully with antibiotics, with the requirement of catheter removal for 2 patients who had systemic infections.

PMID

12567313 [https://www.ncbi.nlm.nih.gov/pubmed/?term=12567313]

Status

Embase

Institution

(Pham, Tarrand, Han) Section of Clinical Microbiology, Univ. Texas M. D. Anderson Cancer C., Houston, TX, United States (De, Rolston) Section of Infectious Diseases, Univ. Texas M. D. Anderson Cancer C., Houston, TX, United States
(Han) Section of Clinical Microbiology, Univ. Texas M. D. Anderson Cancer C., Unit 84, 1515 Holcombe Blvd., Houston, TX 77030, United States

Publisher

Oxford University Press (2001 Evans Road, Cary NC 27513, United States)

Emtree Heading

*Actinobacteria; acute granulocytic leukemia; adult; antibiotic sensitivity; antibiotic therapy; article; *bacteremia/co [Complication]; *bacteremia/di [Diagnosis]; *bacteremia/dt [Drug Therapy]; *bacteremia/et [Etiology]; bacterium identification; blood culture; brain tumor; *cancer patient; *catheter infection/co [Complication]; *catheter infection/di [Diagnosis]; *catheter infection/dt [Drug Therapy]; *catheter infection/et [Etiology]; chronic myeloid leukemia; clinical article; DNA sequence; female; fever;
human
; liver metastasis; male; nucleotide sequence; polymerase chain reaction; priority journal; Rhodococcus; thyroid cancer; ampicillin; *antibiotic agent/dt [Drug Therapy]; azithromycin/cb [Drug Combination]; azithromycin/dt [Drug Therapy]; aztreonam/dt [Drug Therapy]; ceftazidime/cb [Drug Combination]; ceftazidime/dt [Drug Therapy]; ceftriaxone; ciprofloxacin; clindamycin/cb [Drug Combination]; clindamycin/dt [Drug Therapy]; cotrimoxazole; doxycycline; erythromycin/cb [Drug Combination]; erythromycin/dt [Drug Therapy]; genomic DNA/ec [Endogenous Compound]; gentamicin; imipenem/cb [Drug Combination]; imipenem/dt [Drug Therapy]; levofloxacin/cb [Drug Combination]; levofloxacin/dt [Drug Therapy]; oxacillin; penicillin G; rifampicin; RNA 16S/ec [Endogenous Compound]; vancomycin/cb [Drug Combination]; vancomycin/dt [Drug Therapy]; gordonia terrae.

Candidate Terms

gordonia terrae [other term].

Drug Index Terms

ampicillin; *antibiotic agent / *drug therapy; azithromycin / drug combination / drug therapy; aztreonam / drug therapy; ceftazidime / drug combination / drug therapy; ceftriaxone; ciprofloxacin; clindamycin / drug combination / drug therapy; cotrimoxazole; doxycycline; erythromycin / drug combination / drug therapy; genomic DNA / endogenous compound; gentamicin; imipenem / drug combination / drug therapy; levofloxacin / drug combination / drug therapy; oxacillin; penicillin G; rifampicin; RNA 16S / endogenous compound; vancomycin / drug combination / drug therapy.

Other Index Terms

*Actinobacteria; acute granulocytic leukemia; adult; antibiotic sensitivity; antibiotic therapy; article; *bacteremia / *complication / *diagnosis / *drug therapy / *etiology; bacterium identification; blood culture; brain tumor; *cancer patient; *catheter infection / *complication / *diagnosis / *drug therapy / *etiology; chronic myeloid leukemia; clinical article; DNA sequence; female; fever; human; liver metastasis; male; nucleotide sequence; polymerase chain reaction; priority journal; Rhodococcus; thyroid cancer.

CAS Registry Numbers

69-52-3 (ampicillin); 69-53-4 (ampicillin); 7177-48-2 (ampicillin); 74083-13-9 (ampicillin); 94586-58-0 (ampicillin); 83905-01-5 (azithromycin); 78110-38-0 (aztreonam); 72558-82-8 (ceftazidime); 73384-59-5 (ceftriaxone); 74578-69-1 (ceftriaxone); 85721-33-1 (ciprofloxacin); 18323-44-9 (clindamycin); 8064-90-2 (cotrimoxazole); 10592-13-9 (doxycycline); 17086-28-1 (doxycycline); 564-25-0 (doxycycline); 114-07-8 (erythromycin); 70536-18-4 (erythromycin); 1392-48-9 (gentamicin); 1403-66-3 (gentamicin); 1405-41-0 (gentamicin); 64221-86-9 (imipenem); 100986-85-4 (levofloxacin); 138199-71-0 (levofloxacin); 1173-88-2 (oxacillin); 66-79-5 (oxacillin); 7240-38-2 (oxacillin); 1406-05-9 (penicillin G); 61-33-6 (penicillin G); 13292-46-1 (rifampicin); 1404-90-6 (vancomycin); 1404-93-9 (vancomycin)

Year of Publication

2003

Cite

96.
Lipoarabinomannans: From structure to biosynthesis.

Nigou J., Gilleron M., Puzo G.

Embase
Biochimie. 85(1-2) (pp 153-166), 2003. Date of Publication: January/February 2003.

[Review]

AN:
36625990

Mycobacterium tuberculosis, the causative agent of tuberculosis, is one of the most effective **human** pathogens and the molecular basis of its virulence remains poorly understood. Here, we review our current knowledge about the structure and biosynthesis of the mycobacterial cell-wall lipoglycans, lipoarabinomannans (LAM). LAM are ubiquitous of mycobacteria and appear as the most potent non-peptidic molecules to modulate the host immune response. Nevertheless, LAM structure differs according to the mycobacterial species and three types of LAM have been described: mannose-capped LAM (ManLAM), phospho-wyo-inositol-capped LAM (PILAM) and non-capped LAM (AraLAM). The type of capping is a major structural feature determining the ability of LAM to modulate the immune response. ManLAM, found in slow-growing mycobacteria, such as M. tuberculosis, have been demonstrated to be powerful anti-inflammatory molecules and emerge as key virulence factors that may be relevant drug targets. LAM-like molecules are not only confined to mycobacteria but are also present in actinomycetes (including the genera Rhodococcus, Corynebacterium or **Gordonia**). This offers the possibility of comparative studies that should help in deciphering the structure-function relationships and biosynthesis of these complex molecules in the future. © 2003 Editions scientifiques et medicales Elsevier SAS and Societe francaise de biochimie et biologie moleculaire. All rights reserved.

PMID

12765785 [https://www.ncbi.nlm.nih.gov/pubmed/?term=12765785]

Status

Embase

Institution

(Nigou, Gilleron, Puzo) Inst. de Pharmacol./de Biol. Struct., CNRS UMR 5089, 205 Route de Narbonne, 31077 Toulouse cedex 4, France

Publisher

Elsevier (P.O. Box 211, Amsterdam 1000 AE, Netherlands)

Keyword Heading

Lipoglycan, Mycobacterium, Rhodococcus, Tuberculosis

Emtree Heading

Actinobacteria; antiinflammatory activity; bacterial cell wall; bacterial gene; bacterial virulence; *carbohydrate analysis; *carbohydrate synthesis; Corynebacterium; immune response; molecular dynamics; Mycobacterium; Mycobacterium tuberculosis; nonhuman; protein motif; review; Rhodococcus; signal transduction; tuberculosis; inositol phosphate/ec [Endogenous Compound]; *lipoarabinomannan/ec [Endogenous Compound]; mannan/ec [Endogenous Compound]; mannose/ec [Endogenous Compound].

Drug Index Terms

inositol phosphate / endogenous compound; *lipoarabinomannan / *endogenous compound; mannan / endogenous compound; mannose / endogenous compound.

Other Index Terms

Actinobacteria; antiinflammatory activity; bacterial cell wall; bacterial gene; bacterial virulence; *carbohydrate analysis; *carbohydrate synthesis; Corynebacterium; immune response; molecular dynamics; Mycobacterium; Mycobacterium tuberculosis; nonhuman; protein motif; review; Rhodococcus; signal transduction; tuberculosis.

CAS Registry Numbers

15421-51-9 (inositol phosphate); 51395-96-1 (mannan); 9036-88-8 (mannan); 31103-86-3 (mannose); 3458-28-4 (mannose)

Year of Publication

2003

Cite

97.

Pathogenic Nocardia, Rhodococcus, and related organisms are highly susceptible to imidazole antifungals.

Dabbs E.R., Naidoo S., Lephoto C., Nikitina N.

Embase
Antimicrobial Agents and Chemotherapy. 47(4) (pp 1476-1478), 2003. Date of Publication: 01 Apr 2003.

[Article]

AN:
36368625

Rhodococcus equi and species of Nocardia and **Gordonia** may be **human** opportunistic pathogens. We find that these, as well as several isolates from closely related genera, are highly susceptible to the imidazoles bifonazole, clotrimazole, econazole, and miconazole, whose MICs are <=1 mug/ml. In liquid cultures 1 mug of the drug/ml was bacteriostatic and 10 mug/ml was bactericidal. On solid media at 10 mug of azole/ml no resistant mutants could be isolated. An MIC of 1 to 15 mu/ml was observed with ketoconazole, whereas none of these organisms was inhibited by the triazoles fluconazole and voriconazole (100 mug/ml). Imidazoles may offer the prospect of treatment of nocardioform mycetomas and may provide the basis for the development of additional antimicrobial agents to combat these pathogens.

PMID

12654698 [https://www.ncbi.nlm.nih.gov/pubmed/?term=12654698]

Status

Embase

Institution

(Dabbs, Naidoo, Lephoto, Nikitina) School of Molecular Biology, University of the Witwatersrand, Johannesburg P O WITS 2050, South Africa

Publisher

American Society for Microbiology (1752 N Street N.W., Washington DC 20036-2904, United States)

Emtree Heading

*antibiotic sensitivity; article; bactericidal activity; bacteriostasis; culture medium; fungus culture; fungus isolation; minimum inhibitory concentration; mycetoma; *Nocardia; nonhuman; priority journal; *Rhodococcus; *antifungal agent; bifonazole; clotrimazole; econazole; *imidazole derivative; ketoconazole; voriconazole.

Drug Index Terms

*antifungal agent; bifonazole; clotrimazole; econazole; *imidazole derivative; ketoconazole; voriconazole.

Other Index Terms

*antibiotic sensitivity; article; bactericidal activity; bacteriostasis; culture medium; fungus culture; fungus isolation; minimum inhibitory concentration; mycetoma; *Nocardia; nonhuman; priority journal; *Rhodococcus.

CAS Registry Numbers

60628-96-8 (bifonazole); 23593-75-1 (clotrimazole); 24169-02-6 (econazole); 27220-47-9 (econazole); 65277-42-1 (ketoconazole); 137234-62-9 (voriconazole)

Year of Publication

2003

Cite

98.
Bacteremia and endocarditis caused by a **Gordonia** species in a patient with a central venous catheter.

Lesens O., Hansmann Y., Riegel P., Heller R., Benaissa-Djellouli M., Martinot M., Petit H., Christmann D.

Embase
Emerging Infectious Diseases. 6(4) (pp 382-385), 2000. Date of Publication: 2000.

[Article]

AN:
30643660

We report the first case of endocarditis caused by a **Gordonia** species genetically related to G. sputi but exhibiting some atypical biochemical features in a 31-year-old woman with a central venous catheter. This unusual pathogen may be a new cause of opportunistic infections in patients with severe underlying diseases.

PMID

10905972 [https://www.ncbi.nlm.nih.gov/pubmed/?term=10905972]

Status

Embase

Institution

(Lesens, Hansmann, Benaissa-Djellouli, Martinot, Christmann) Service des Maladies Infectieuses et Tropicales, Clinique Medicale A, Hopitaux Universitaires, Strasbourg, France (Riegel, Heller) Laboratoire de Bacteriologie, Faculte de Medecine, Hopitaux Universitaires, Strasbourg, France
(Petit) Service de Chirurgie Cardio-Vasculaire, Hopitaux Universitaires, Strasbourg, France
(Lesens) Department of Infectious Diseases, Strasbourg Hospital, France
(Lesens) Service des Maladies Infectieuses et Tropicales, Clinique Medicale A, Federation des Services de Medecine Interne, 1 Place de l'Hopital, 67091 Strasbourg Cedex, France

Publisher

Centers for Disease Control and Prevention (CDC) (1600 Clifton Road, Mailstop C-12, Atlanta GA 30333, United States)

Emtree Heading

adult; article; *bacteremia; *bacterial endocarditis/et [Etiology]; case report; central venous catheter; *Corynebacterium; female;
human
; infection risk; opportunistic infection; physical examination; *Gordonia.

Candidate Terms

*gordonia [other term].

Other Index Terms

adult; article; *bacteremia; *bacterial endocarditis / *etiology; case report; central venous catheter; *Corynebacterium; female; human; infection risk; opportunistic infection; physical examination.

Year of Publication

2000

Cite

99.
Rhodococcus Equi. [Review]

Ayoade F, Alam MU

Ovid MEDLINE(R) and Epub Ahead of Print, In-Process, In-Data-Review & Other Non-Indexed Citations, Daily and Versions StatPearls Publishing. 2021 01.

[Review]

UI:
28723007

Human infection due to *Rhodococcus equi* was first reported in 1967 by a young man on immunosuppressant agents who was working in a stockyard. As the name implies, the **infection** is closely linked to animals, horses and foal are considered as the natural host. Other species of *Rhodococcus* similarly described as **human** pathogens include *Rhodococcus fascians, Rhodococcus rhodochrous, and Rhodococcus erythropolis*. Rhodococcus belongs to the Nocardiaceae family which also comprises *Nocardia*, *Mycobacterium*, *Corynebacterium*, and ***Gordonia*** with some similarities among the group.[1][2] Immunosuppression, especially defects in cell-mediated immunity, plays a major role in disease and is present in most reported cases even though **infection** has also been described less commonly in immunocompetent hosts. Common immunocompromised conditions where **infection** had been described include HIV, solid organ and stem cell transplant recipients, leukemia, lymphoma, lung cancer, and following chemotherapy, monoclonal antibodies, or prolonged steroid use.[3]

Copyright © 2021, StatPearls Publishing LLC.

Book Title

StatPearls

Version ID

1

Related Item DOI

From MEDLINE, a database of the U.S. National Library of Medicine.

Authors Full Name

Ayoade, Folusakin, Alam, Mohammed U.

Institution

Ayoade, Folusakin. University of Miami Alam, Mohammed U.. Louisiana State University HSC

Publisher

StatPearls Publishing

Year of Publication

2021

Cite

100.
Recurrent Skin and Soft Tissue **Infection** following Breast Reduction Surgery Caused by ***Gordonia*** *bronchialis*: A Case Report.

Davidson AL, Driscoll CR, Luther VP, Katz AJ

Ovid MEDLINE(R) and Epub Ahead of Print, In-Process, In-Data-Review & Other Non-Indexed Citations, Daily and Versions
Plastic and Reconstructive Surgery - Global Open. 10(6):e4395, 2022 Jun.

[Case Reports]

UI:
35702356

The expanding knowledge of the breast microbiome and its constituents necessitates understanding of how it plays into **human** disease. Consideration of how to identify novel organisms in breast tissue is a topic of hot debate. We report a case of a 26-year-old woman with repeat incisional break-down and sanguinopurulent drainage who required repeat incision and drainage procedures after bilateral breast reduction. Cultures revealed no growth until 4 months postoperation when matrix-assisted laser desorption/ionization-time of flight (MALDI-TOF) revealed ***Gordonia*** *bronchialis*, a fastidious, slow-growing organism. To date, there are fewer than 30 reported cases of *G. bronchialis* infections and only one with breast involvement. Our patient required 6 weeks of amoxicillin-clavulanate therapy and frequent follow-up for symptom resolution. This case demonstrates the need for additional microbiologic data in patients with delayed, persistent infections after breast surgery.

Copyright © 2022 The Authors. Published by Wolters Kluwer Health, Inc. on behalf of The American Society of Plastic Surgeons.

Version ID

1

Related Item DOI

From MEDLINE, a database of the U.S. National Library of Medicine.

Status

PubMed-not-MEDLINE

Authors Full Name

Davidson, Amelia L, Driscoll, Cassandra R, Luther, Vera P, Katz, Adam J

Institution

Davidson, Amelia L. Wake Forest University School of Medicine, Winston-Salem, N.C. Driscoll, Cassandra R. Department of Plastic and Reconstructive Surgery, Atrium Health Wake Forest Baptist, Winston-Salem, N.C.
Luther, Vera P. Department of Infectious Disease, Atrium Health Wake Forest Baptist, Winston-Salem, N.C.
Katz, Adam J. Department of Plastic and Reconstructive Surgery, Atrium Health Wake Forest Baptist, Winston-Salem, N.C.

PMID

https://www.ncbi.nlm.nih.gov/pmc/articles/PMC9187191

Year of Publication

2022

Cite

101.
Gordonia *crocea* sp. nov. Isolated from Wound **Infection** After Pacemaker Implantation: Case Report and Literature Review.

Yang Z, Zhang Z, Chen M, Liu Z

Ovid MEDLINE(R) and Epub Ahead of Print, In-Process, In-Data-Review & Other Non-Indexed Citations, Daily and Versions
Infection & Drug Resistance. 15:2915-2920, 2022.

[Case Reports]

UI:
35698534

Gordonia is a recognized pathogen in patients with immunodeficiency and a normal immune response, which can cause bacteremia, endocarditis, peritonitis and pulmonary **infection**. We report a case of wound **infection** after pacemaker implantation caused by ***Gordonia*** *crocea*. Matrix-assisted laser desorption time-of-flight mass spectrometry (MALDI-TOF MS) was routinely used to identify the pathogen, and the results showed that the pathogen could not be accurately identified in the MALDI-TOF database at present. The 16S rRNA gene of the pathogen was further sequenced, and the result was ***Gordonia*** *crocea*. To the best of our knowledge, this is the first reported case of **human infection** caused by ***Gordonia*** *crocea*.

Copyright © 2022 Yang et al.

Version ID

1

Related Item DOI

From MEDLINE, a database of the U.S. National Library of Medicine.

Status

PubMed-not-MEDLINE

Author Initials

Liu, Zhiyong; ORCID: https://orcid.org/0000-0003-0246-3183

Authors Full Name

Yang, Zhulan, Zhang, Zhen, Chen, Ming, Liu, Zhiyong

Institution

Yang, Zhulan. Department of Clinical Laboratory, Southwest Hospital, Army Medical University, Chongqing, People's Republic of China. Zhang, Zhen. Department of Clinical Laboratory, Chongqing General Hospital, University of Chinese Academy of Sciences, Chongqing, People's Republic of China.
Chen, Ming. Department of Clinical Laboratory, Southwest Hospital, Army Medical University, Chongqing, People's Republic of China.
Liu, Zhiyong. Department of Clinical Laboratory, Southwest Hospital, Army Medical University, Chongqing, People's Republic of China.

PMID

https://www.ncbi.nlm.nih.gov/pmc/articles/PMC9188390

Keyword Heading

Gordonia crocea case report
pacemaker implantation
wound infection.

Year of Publication

2022

Cite

102.
Actinomycetoma caused by ***Gordonia*** *westfalica*: first reported case of **human infection**.

Gueneau R, Blanchet D, Rodriguez-Nava V, Bergeron E, Soulier M, Bestandji N, Demar M, Couppie P, Blaizot R

Ovid MEDLINE(R) and Epub Ahead of Print, In-Process, In-Data-Review & Other Non-Indexed Citations, Daily and Versions
New Microbes & New Infections. 34:100658, 2020 Mar.

[Journal Article]

UI:
32194964

Bacteria of the genus ***Gordonia*** are rarely involved in **human** infections. We report here the case of a 30-year-old man from Guinea Buissau with mycetoma of the foot. 16S DNA sequencing after surgical biopsy identified ***Gordonia westfalica***. To our knowledge, this is the first report of **human infection** caused by *G. westfalica*.

Copyright © 2020 The Authors.

Version ID

1

Related Item DOI

From MEDLINE, a database of the U.S. National Library of Medicine.

Status

PubMed-not-MEDLINE

Authors Full Name

Gueneau, R, Blanchet, D, Rodriguez-Nava, V, Bergeron, E, Soulier, M, Bestandji, N, Demar, M, Couppie, P, Blaizot, R

Institution

Gueneau, R. Dermatology Department, University of French Guiana, Cayenne, French Guiana. Blanchet, D. Laboratory of Parasitology and Mycology, University of French Guiana, Cayenne, French Guiana.
Rodriguez-Nava, V. UMR CNRS 5557, Center for Microbial Ecology, Observatoire Francais des Nocardioses, Laboratoire de Mycologie Fondamentale et Appliquee aux Biotechnologies Industrielles, Faculte de Pharmacie, Universite Claude Bernard Lyon I, Lyon, France.
Bergeron, E. UMR CNRS 5557, Center for Microbial Ecology, Observatoire Francais des Nocardioses, Laboratoire de Mycologie Fondamentale et Appliquee aux Biotechnologies Industrielles, Faculte de Pharmacie, Universite Claude Bernard Lyon I, Lyon, France.
Soulier, M. Pathologistes associes, Martigues, France.
Bestandji, N. Orthopaedics Department, Hopital Andree Rosemon, University of French Guiana, Cayenne, French Guiana.
Demar, M. Laboratory of Parasitology and Mycology, University of French Guiana, Cayenne, French Guiana.
Demar, M. EA 3593 Ecosystemes Amazoniens et Pathologies Tropicales, University of French Guiana, Cayenne, French Guiana.
Couppie, P. Dermatology Department, University of French Guiana, Cayenne, French Guiana.
Couppie, P. EA 3593 Ecosystemes Amazoniens et Pathologies Tropicales, University of French Guiana, Cayenne, French Guiana.
Blaizot, R. Dermatology Department, University of French Guiana, Cayenne, French Guiana.
Blaizot, R. EA 3593 Ecosystemes Amazoniens et Pathologies Tropicales, University of French Guiana, Cayenne, French Guiana.

PMID

https://www.ncbi.nlm.nih.gov/pmc/articles/PMC7078390

Keyword Heading

Actinomyces Gordonia
Mycetoma
Neglected tropical disease
Spectrum analysis.

Year of Publication

2020

Cite

103.

Sternal osteomyelitis by ***Gordonia*** *Bronchialis* in an immunocompetent patient after open heart surgery.

Ambesh P, Kapoor A, Kazmi DH, Elsheshtawy M, Shetty V, Lin YS, Kamholz S

Ovid MEDLINE(R) and Epub Ahead of Print, In-Process, In-Data-Review & Other Non-Indexed Citations, Daily and Versions
Annals of Cardiac Anaesthesia. 22(2):221-224, 2019 Apr-Jun.

[Case Reports. Journal Article]

UI:
30971609

Gordonia is a catalase-positive, aerobic, nocardioform, Gram-positive staining actinomycete that also shows weak acid-fast staining. Several **Gordonia** species are commonly found in the soil. The bacterium has been isolated from the saliva of domesticated/wild dogs as well. In hospitalized patients, most commonly it is found in the setting of intravascular catheter-related infections. However, recent reports show that it is being increasingly isolated from sternal wounds, skin/neoplastic specimens and from pleural effusions. **Gordonia** shares many common characteristics with Rhodococcus and Nocardia. Ergo, it is commonly misrecognized as Nocardia or Rhodococcus. Since this pathogen requires comprehensive morphological and biochemical testing, it is often difficult and cumbersome to isolate the species. Broad-range Polymerase Chain Reaction (PCR) and sequencing with genes like 16S rRNA or hsp65 are used to correctly identify the species. Identification is essential for choosing and narrowing the right antimicrobial agent. Herein, we report our experience with a patient who presented with sternal osteomyelitis after **infection** with this elusive bug.

Version ID

1

Related Item DOI

From MEDLINE, a database of the U.S. National Library of Medicine.

Status

MEDLINE

Authors Full Name

Ambesh, Paurush, Kapoor, Aditya, Kazmi, Danish H, Elsheshtawy, Moustafa, Shetty, Vijay, Lin, Yu S, Kamholz, Stephan

Institution

Ambesh, Paurush. Department of Internal Medicine, Maimonides Medical Center, New York City, USA. Kapoor, Aditya. Department of Cardiology, Sanjay Gandhi Post Graduate Institute of Medical Sciences, Lucknow, India.
Kazmi, Danish H. Department of Cardiology, Era Medical Institute, Lucknow, India.
Elsheshtawy, Moustafa. Department of Cardiology, Maimonides Medical Center, New York City, USA.
Shetty, Vijay. Department of Cardiology, Maimonides Medical Center, New York City, USA.
Lin, Yu S. Department of Infectious Disease, Maimonides Medical Center, New York City, USA.
Kamholz, Stephan. Department of Internal Medicine, Maimonides Medical Center, New York City, USA.

PMID

https://www.ncbi.nlm.nih.gov/pmc/articles/PMC6489382

Emtree Heading

*Actinobacteria/ip [Isolation & Purification]. *Actinomycetales Infections/di [Diagnosis]. Actinomycetales Infections/th [Therapy]. Aged. *Cardiac Surgical Procedures. Humans. Immunocompetence. Male. Osteomyelitis/di [Diagnosis]. *Osteomyelitis/mi [Microbiology]. Osteomyelitis/th [Therapy]. *Postoperative Complications/mi [Microbiology]. Postoperative Complications/th [Therapy]. *Sternum/mi [Microbiology].

Keyword Heading

*Gordonia bronchialis *infection
*sternal osteomyelitis.

Year of Publication

2019

Cite

104.
An insight into the ecology, diversity and adaptations of **Gordonia** species. [Review]

Sowani H, Kulkarni M, Zinjarde S

Ovid MEDLINE(R) and Epub Ahead of Print, In-Process, In-Data-Review & Other Non-Indexed Citations, Daily and Versions
Critical Reviews in Microbiology. 44(4):393-413, 2018 Aug.

[Journal Article. Review]

UI:
29276839

The bacterial genus **Gordonia** encompasses a variety of versatile species that have been isolated from a multitude of environments. **Gordonia** was described as a genus about 20 years ago, and to date, 39 different species have been identified. **Gordonia** is recognized for symbiotic associations with multiple hosts, including aquatic (marine and fresh water) biological forms and terrestrial invertebrates. Some **Gordonia** species isolated from clinical specimens are known to be opportunistic **human** pathogens causing secondary infections in immunocompromised and immunosuppressive individuals. They are also predominant in mangrove ecosystems and terrestrial sites. Members of the genus **Gordonia** are ecologically adaptable and show marked variations in their properties and products. They generate diverse bioactive compounds and produce a variety of extracellular enzymes. In addition, production of surface active compounds and carotenoid pigments allows this group of microorganisms to grow under different conditions. Several isolates from water and soil have been implicated in bioremediation of different environments and plant associated species have been explored for agricultural applications. This review highlights the prevalence of the members of this versatile genus in diverse environments, details its associations with living forms, summarizes the biotechnologically relevant products that can be obtained and discusses the salient genomic features that allow this Actinomycete to survive in different ecological niches.

Version ID

1

Related Item DOI

From MEDLINE, a database of the U.S. National Library of Medicine.

Status

MEDLINE

Authors Full Name

Sowani, Harshada, Kulkarni, Mohan, Zinjarde, Smita

Institution

Sowani, Harshada. a Department of Chemistry, Biochemistry Division Savitribai Phule Pune University, Pune, India. Kulkarni, Mohan. a Department of Chemistry, Biochemistry Division Savitribai Phule Pune University, Pune, India.
Zinjarde, Smita. b Institute of Bioinformatics and Biotechnology, Savitribai Phule Pune University, Pune, India.
Zinjarde, Smita. c Department of Microbiology, Savitribai Phule Pune University, Pune, India.

Emtree Heading

*Actinomycetales Infections/mi [Microbiology]. Adaptation, Physiological. Animals. *Biodiversity. Ecosystem. *Environmental Microbiology. Gordonia Bacterium/cl [Classification]. Gordonia Bacterium/ge [Genetics]. *Gordonia Bacterium/ip [Isolation & Purification]. *Gordonia Bacterium/ph [Physiology]. Humans. Phylogeny.

Keyword Heading

Gordonia biosurfactants
carotenoids
marine
soil.

Year of Publication

2018

Cite

105.

16S rRNA gene amplicon sequencing reveals dominance of Actinobacteria in Rhodnius pallescens compared to Triatoma maculata midgut microbiota in natural populations of vector insects from Colombia.

Montoya-Porras LM, Omar TC, Alzate JF, Moreno-Herrera CX, Cadavid-Restrepo GE

Ovid MEDLINE(R) and Epub Ahead of Print, In-Process, In-Data-Review & Other Non-Indexed Citations, Daily and Versions
Acta Tropica. 178:327-332, 2018 Feb.

[Journal Article]

UI:
29154947

Chagas disease affects more than 6 million people in Latin America, it is a parasitic disease caused by the protozoan Trypanosoma cruzi, which is transmitted mainly by bloodsucking insects of the Triatominae subfamily. Studies on microbial communities that inhabit the insect gut are important to understanding their role in the parasite transmission and development. The present work aims to evaluate the gut bacterial composition of natural populations of triatomine species from Vichada and Magdalena, administrative states called departments in Colombia, using high-throughput sequencing technologies. The insects were collected from housing peridomestic area and Attalea butyracea palms; they were identified by conventional taxonomy as Triatoma maculata and Rhodnius pallescens, and their guts were dissected under aseptic conditions in order to obtain total DNA. After DNA quality confirmation, the sequencing of the V4 region of 16S rRNA gene was carried out using the Illumina platform MiSeq. The results showed that 13 predominant bacterial genera were present in both species, being Burkholderia, **Gordonia**, and Ralstonia, the most prevailing bacterial genera. Furthermore, representative genera of each species were found. Williamsia and Kocuria were the most common in R. pallescens; and Dietzia, Aeromonas, and Pelomonas were only observed in T. maculata samples. This is the first study of microbiota associated with these triatomine species using massive sequencing methods The approach allowed inferring the presence of a dominant population of bacteria according to the triatomine species in Colombia, which may suggest a strong association between microbiota and their host.

Copyright © 2017 Elsevier B.V. All rights reserved.

Version ID

1

Related Item DOI

From MEDLINE, a database of the U.S. National Library of Medicine.

Status

MEDLINE

Authors Full Name

Montoya-Porras, Luisa M, Omar, Triana-Chavez, Alzate, Juan F, Moreno-Herrera, Claudia X, Cadavid-Restrepo, Gloria E

Institution

Montoya-Porras, Luisa M. Grupo de Microbiodiversidad y Bioprospeccion-MICROBIOP, Laboratorio de Biologia Celular y Molecular, Facultad de Ciencias, Universidad Nacional de Colombia Sede Medellin, Calle 59A # 63-20, Postal Code 050024, Colombia. Omar, Triana-Chavez. Grupo Biologia y Control de Enfermedades Infecciosas-BCEI, Universidad de Antioquia, Calle 70 # 52-21 Medellin, Colombia.
Alzate, Juan F. Centro Nacional de Secuenciacion Genomica-CNSG, Sede de Investigacion Universitaria-SIU, Facultad de Medicina, Universidad de Antioquia Carrera 53 # 61-30 Medellin, Colombia.
Moreno-Herrera, Claudia X. Grupo de Microbiodiversidad y Bioprospeccion-MICROBIOP, Laboratorio de Biologia Celular y Molecular, Facultad de Ciencias, Universidad Nacional de Colombia Sede Medellin, Calle 59A # 63-20, Postal Code 050024, Colombia.
Cadavid-Restrepo, Gloria E. Grupo de Microbiodiversidad y Bioprospeccion-MICROBIOP, Laboratorio de Biologia Celular y Molecular, Facultad de Ciencias, Universidad Nacional de Colombia Sede Medellin, Calle 59A # 63-20, Postal Code 050024, Colombia. Electronic address: gecadavi@unal.edu.co.

Emtree Heading

*Actinobacteria/ge [Genetics]. Actinobacteria/ip [Isolation & Purification]. Animals. Chagas Disease/ep [Epidemiology]. *Chagas Disease/tm [Transmission]. Colombia/ep [Epidemiology]. Humans. Insect Vectors/ps [Parasitology]. Microbiota. RNA, Bacterial/ge [Genetics]. *RNA, Ribosomal, 16S/ge [Genetics]. *Rhodnius/mi [Microbiology]. *Triatoma/mi [Microbiology]. Trypanosoma cruzi/ge [Genetics].

Keyword Heading

American trypanosomiasis Bacterial diversity
NGS
Triatomines
Trypanosoma cruzi.

CAS Registry Numbers

0 (RNA, Bacterial). 0 (RNA, Ribosomal, 16S).

Year of Publication

2018

Cite

106.
First identification of **Gordonia** sputi in a post-traumatic endophthalmitis patient - a case report and literatures review.

Fang W, Li J, Cui HS, Jin X, Zhai J, Dai Y, Li Y

Ovid MEDLINE(R) and Epub Ahead of Print, In-Process, In-Data-Review & Other Non-Indexed Citations, Daily and Versions
BMC Ophthalmology. 17(1):190, 2017 Oct 11.

[Case Reports. Journal Article]

UI:
29020920

BACKGROUND: We present a case of post-traumatic endophthalmitis with relatively good prognosis caused by **Gordonia** sputi, which, to our knowledge is the first case in the literature.
CASE PRESENTATION: A 24 year old man, who underwent an intraocular foreign body extraction half a month before presentation in the left eye, was referred to us complaining of blurred vision and slight pain for 5 days. His first presentation showed moderate intracameral and intravitreous purulent inflammation with a best corrected vision of counting fingers. After gram staining of the intravitreous samples revealed a gram-positive bacilli **infection**, a combination of amikacin and vancomycin was initially injected intravitreously. The left eye kept stable for three days but deteriorated on the 4th day. On the 5th day after presentation conventional culture characterized the bacterium as an Actinomyces sp. while 16S ribosomal RNA gene sequencing confirmed it as **Gordonia** sputi. Thereby a complete pars plana vitrectomy combined with lensectomy and silicone oil tamponade was performed. During the surgery an intraocular irrigation with penicillin G was adopted, followed by administration of intravenous penicillin G twice one day for a week. A relatively normal fundus with slight intracameral inflammation was observed a week after the operation, and the best corrected vision recovered to 0.15. One year later his vision remained 0.1.
CONCLUSION: **Gordonia** sputi should be taken into consideration in patients with post-traumatic endophthalmitis especially due to foreign body penetration. Compared to conventional laboratories, molecular methods are recommended for an accurate diagnosis. A comprehensive strategy of antimicrobial agents and vitrectomy may render a satisfactory result.

Version ID

1

Related Item DOI

From MEDLINE, a database of the U.S. National Library of Medicine.

Status

MEDLINE

Authors Full Name

Fang, Wei, Li, Jiuke, Cui, Hu-Shan, Jin, Xiaohong, Zhai, Jing, Dai, Yuanmin, Li, Yumin

Institution

Fang, Wei. Ophthalmology Department of SIR RUN RUN SHAW hospital, SIR RUN RUN SHAW Institute of Clinical Medicine of Zhejiang University, #3 Qingchun East Road, Hangzhou, Zhejiang, 310016, People's Republic of China. Li, Jiuke. Ophthalmology Department of SIR RUN RUN SHAW hospital, SIR RUN RUN SHAW Institute of Clinical Medicine of Zhejiang University, #3 Qingchun East Road, Hangzhou, Zhejiang, 310016, People's Republic of China.
Cui, Hu-Shan. Ophthalmology Department of SIR RUN RUN SHAW hospital, SIR RUN RUN SHAW Institute of Clinical Medicine of Zhejiang University, #3 Qingchun East Road, Hangzhou, Zhejiang, 310016, People's Republic of China.
Jin, Xiaohong. Ophthalmology Department of SIR RUN RUN SHAW hospital, SIR RUN RUN SHAW Institute of Clinical Medicine of Zhejiang University, #3 Qingchun East Road, Hangzhou, Zhejiang, 310016, People's Republic of China.
Zhai, Jing. Ophthalmology Department of SIR RUN RUN SHAW hospital, SIR RUN RUN SHAW Institute of Clinical Medicine of Zhejiang University, #3 Qingchun East Road, Hangzhou, Zhejiang, 310016, People's Republic of China.
Dai, Yuanmin. Ophthalmology Department of SIR RUN RUN SHAW hospital, SIR RUN RUN SHAW Institute of Clinical Medicine of Zhejiang University, #3 Qingchun East Road, Hangzhou, Zhejiang, 310016, People's Republic of China.
Li, Yumin. Ophthalmology Department of SIR RUN RUN SHAW hospital, SIR RUN RUN SHAW Institute of Clinical Medicine of Zhejiang University, #3 Qingchun East Road, Hangzhou, Zhejiang, 310016, People's Republic of China. liyumin77@hotmail.com.

PMID

https://www.ncbi.nlm.nih.gov/pmc/articles/PMC5637324

Emtree Heading

*Actinomycetales Infections/di [Diagnosis]. Actinomycetales Infections/mi [Microbiology]. Actinomycetales Infections/th [Therapy]. Anti-Bacterial Agents/tu [Therapeutic Use]. Combined Modality Therapy. *Endophthalmitis/di [Diagnosis]. Endophthalmitis/mi [Microbiology]. Endophthalmitis/th [Therapy]. Endotamponade. *Eye Foreign Bodies/di [Diagnosis]. Eye Foreign Bodies/mi [Microbiology]. Eye Foreign Bodies/th [Therapy]. *Eye Infections, Bacterial/di [Diagnosis]. Eye Infections, Bacterial/mi [Microbiology]. Eye Infections, Bacterial/th [Therapy]. *Eye Injuries, Penetrating/di [Diagnosis]. Eye Injuries, Penetrating/mi [Microbiology]. Eye Injuries, Penetrating/th [Therapy]. Gordonia Bacterium/ge [Genetics]. *Gordonia Bacterium/ip [Isolation & Purification]. Humans. Lens, Crystalline/su [Surgery]. Male. Penicillin G/tu [Therapeutic Use]. RNA, Bacterial/ge [Genetics]. RNA, Ribosomal, 16S/ge [Genetics]. Silicone Oils/ad [Administration & Dosage]. Vitrectomy. Young Adult.

Keyword Heading

Actinomyces Case report
Endophthalmitis
Gordonia sputi
Traumatic.

CAS Registry Numbers

0 (Anti-Bacterial Agents). 0 (RNA, Bacterial). 0 (RNA, Ribosomal, 16S). 0 (Silicone Oils). Q42T66VG0C (Penicillin G).

Year of Publication

2017

Cite

107.
Gordonia: isolation and identification in clinical samples and role in biotechnology. [Review]

Andalibi F, Fatahi-Bafghi M

Ovid MEDLINE(R) and Epub Ahead of Print, In-Process, In-Data-Review & Other Non-Indexed Citations, Daily and Versions
Folia Microbiologica. 62(3):245-252, 2017 May.

[Journal Article. Review]

UI:
28105601

Gordonia spp. are members of the actinomycete family, and the environment, especially soil, is the natural habitat of this genus of bacteria. **Gordonia** spp. are important for two aspects: first, some **Gordonia** species cause a broad spectrum of diseases in healthy and immunocompromised individuals; second, these bacteria are capable of producing useful secondary metabolites, which may be used in various industries; therefore, discrimination of the genus **Gordonia** from other genera in the actinomycete family is important. Phenotypic and molecular techniques are necessary for accurate identification of **Gordonia** at the species level.

Version ID

1

Related Item DOI

From MEDLINE, a database of the U.S. National Library of Medicine.

Status

MEDLINE

Authors Full Name

Andalibi, Fatemeh, Fatahi-Bafghi, Mehdi

Institution

Andalibi, Fatemeh. Department of Pathobiology, School of Public Health, Tehran University of Medical Sciences, Tehran, Iran. Fatahi-Bafghi, Mehdi. Department of Microbiology, Faculty of Medicine, Shahid Sadoughi University of Medical Sciences, Yazd, Iran. mehdifatahi@ssu.ac.ir.

Emtree Heading

*Actinomycetales Infections/di [Diagnosis]. *Actinomycetales Infections/mi [Microbiology]. Bacteriological Techniques/mt [Methods]. *Biotechnology/mt [Methods]. Environmental Microbiology. *Gordonia Bacterium/ip [Isolation & Purification]. *Gordonia Bacterium/me [Metabolism]. Humans. Molecular Diagnostic Techniques/mt [Methods]. Secondary Metabolism.

Year of Publication

2017

Cite

108.

Identification of Species of Nontuberculous Mycobacteria Clinical Isolates from 8 Provinces of China.

Liu H, Lian L, Jiang Y, Huang M, Tan Y, Zhao X, Zhang J, Yu Q, Liu J, Dong H, Lu B, Wu Y, Wan K

Ovid MEDLINE(R) and Epub Ahead of Print, In-Process, In-Data-Review & Other Non-Indexed Citations, Daily and Versions
BioMed Research International. 2016:2153910, 2016.

[Journal Article]

UI:
27882322

Pulmonary diseases caused by nontuberculous *mycobacteria* (NTM) are increasing in incidence and prevalence worldwide. In this study, we identified NTM species of the clinical isolates from 8 provinces in China, in order to preliminarily provide some basic scientific data in the different species and distribution of NTM related to pulmonary disease in China. A total of 523 clinical isolates from patients with tuberculosis (TB) diagnosed clinically from 2005 to 2012 were identified to the species using conventional and molecular methods, including multilocus PCR, *rpoB* and *hsp65* PCR-PRA, *hsp65*, *rpoB*, and *16S-23S* internal transcribed spacer region sequencing. The isolates were identified into 3 bacterium genera, including NTM, ***Gordonia*** *bronchialis,* and *Nocardia farcinica*, and, for the 488 NTM isolates, 27 species were identified. For all the 27 species of NTM which were found to cause pulmonary infections in humans, the most prevalent species was *M. intracellulare*, followed by *M. avium* and *M. abscessus*. And seven other species were for the first time identified in patients with TB in China. NTM species identification is very important for distinguishing between tuberculosis and NTM pulmonary diseases, and the species diversity drives the creation of diverse and integrated identification methods with higher accuracy and efficacy.

Version ID

1

Related Item DOI

From MEDLINE, a database of the U.S. National Library of Medicine.

Status

MEDLINE
Author Initials

Liu, Haican; ORCID: https://orcid.org/0000-0002-9420-1211 Zhao, Xiuqin; ORCID: https://orcid.org/0000-0003-0084-8119
Wu, Yimou; ORCID: https://orcid.org/0000-0001-6883-1677
Wan, Kanglin; ORCID: https://orcid.org/0000-0002-3997-0237

Authors Full Name

Liu, Haican, Lian, Lulu, Jiang, Yi, Huang, Mingxiang, Tan, Yunhong, Zhao, Xiuqin, Zhang, Jingrui, Yu, Qin, Liu, Jiao, Dong, Haiyan, Lu, Bing, Wu, Yimou, Wan, Kanglin

Institution

Liu, Haican. State Key Laboratory for Infectious Disease Prevention and Control, Collaborative Innovation Center for Diagnosis and Treatment of Infectious Diseases, National Institute for Communicable Disease Control and Prevention, Chinese Center for Disease Control and Prevention, Beijing 102206, China. Lian, Lulu. State Key Laboratory for Infectious Disease Prevention and Control, Collaborative Innovation Center for Diagnosis and Treatment of Infectious Diseases, National Institute for Communicable Disease Control and Prevention, Chinese Center for Disease Control and Prevention, Beijing 102206, China; Pathogenic Biology Institute, University of South China, Hengyang, Hunan 421001, China.
Jiang, Yi. State Key Laboratory for Infectious Disease Prevention and Control, Collaborative Innovation Center for Diagnosis and Treatment of Infectious Diseases, National Institute for Communicable Disease Control and Prevention, Chinese Center for Disease Control and Prevention, Beijing 102206, China.
Huang, Mingxiang. Fuzhou Pulmonary Hospital (Clinical Teaching Hospital of Fujian Medical University), Fuzhou, Fujian 350008, China.
Tan, Yunhong. Hunan Institute for Tuberculosis Control/Hunan Chest Hospital, Changsha, Hunan 410013, China.
Zhao, Xiuqin. State Key Laboratory for Infectious Disease Prevention and Control, Collaborative Innovation Center for Diagnosis and Treatment of Infectious Diseases, National Institute for Communicable Disease Control and Prevention, Chinese Center for Disease Control and Prevention, Beijing 102206, China.
Zhang, Jingrui. State Key Laboratory for Infectious Disease Prevention and Control, Collaborative Innovation Center for Diagnosis and Treatment of Infectious Diseases, National Institute for Communicable Disease Control and Prevention, Chinese Center for Disease Control and Prevention, Beijing 102206, China.
Yu, Qin. State Key Laboratory for Infectious Disease Prevention and Control, Collaborative Innovation Center for Diagnosis and Treatment of Infectious Diseases, National Institute for Communicable Disease Control and Prevention, Chinese Center for Disease Control and Prevention, Beijing 102206, China; Pathogenic Biology Institute, University of South China, Hengyang, Hunan 421001, China.
Liu, Jiao. State Key Laboratory for Infectious Disease Prevention and Control, Collaborative Innovation Center for Diagnosis and Treatment of Infectious Diseases, National Institute for Communicable Disease Control and Prevention, Chinese Center for Disease Control and Prevention, Beijing 102206, China.
Dong, Haiyan. State Key Laboratory for Infectious Disease Prevention and Control, Collaborative Innovation Center for Diagnosis and Treatment of Infectious Diseases, National Institute for Communicable Disease Control and Prevention, Chinese Center for Disease Control and Prevention, Beijing 102206, China.
Lu, Bing. State Key Laboratory for Infectious Disease Prevention and Control, Collaborative Innovation Center for Diagnosis and Treatment of Infectious Diseases, National Institute for Communicable Disease Control and Prevention, Chinese Center for Disease Control and Prevention, Beijing 102206, China.
Wu, Yimou. Pathogenic Biology Institute, University of South China, Hengyang, Hunan 421001, China.
Wan, Kanglin. State Key Laboratory for Infectious Disease Prevention and Control, Collaborative Innovation Center for Diagnosis and Treatment of Infectious Diseases, National Institute for Communicable Disease Control and Prevention, Chinese Center for Disease Control and Prevention, Beijing 102206, China; Pathogenic Biology Institute, University of South China, Hengyang, Hunan 421001, China.

PMID

https://www.ncbi.nlm.nih.gov/pmc/articles/PMC5110891

Emtree Heading

Bacterial Proteins/ge [Genetics]. Chaperonin 60/ge [Genetics]. China. DNA, Ribosomal Spacer/ge [Genetics]. Geography. Gordonia Bacterium/ge [Genetics]. Humans. Lung Diseases/ep [Epidemiology]. Lung Diseases/mi [Microbiology]. Multilocus Sequence Typing. *Mycobacterium/ge [Genetics]. *Mycobacterium Infections, Nontuberculous/ep [Epidemiology]. *Mycobacterium Infections, Nontuberculous/mi [Microbiology]. Nocardia/ge [Genetics]. *Nontuberculous Mycobacteria/ge [Genetics]. Nontuberculous Mycobacteria/ip [Isolation & Purification]. Polymerase Chain Reaction. Prevalence. Reproducibility of Results. Species Specificity.

CAS Registry Numbers

0 (Bacterial Proteins). 0 (Chaperonin 60). 0 (DNA, Ribosomal Spacer). 0 (heat-shock protein 65, Mycobacterium).

Year of Publication

2016

Cite

109.

[Skin **infection** due to **Gordonia** araii]. [Spanish]

Infeccion cutanea por Gordonia araii.

<Infeccion cutanea por
Gordonia
araii.>

Munoz-Pena C, Ocana-Cano MJ, Amores-Antequera C, Cantudo-Munoz P

Ovid MEDLINE(R) and Epub Ahead of Print, In-Process, In-Data-Review & Other Non-Indexed Citations, Daily and Versions
Enfermedades Infecciosas y Microbiologia Clinica. 34(10):685-686, 2016 Dec.

[Case Reports. Letter]

UI:
26993438

Version ID

1

Related Item DOI

From MEDLINE, a database of the U.S. National Library of Medicine.

Status

MEDLINE

Authors Full Name

Munoz-Pena, Cristina, Ocana-Cano, Maria Jose, Amores-Antequera, Carmen, Cantudo-Munoz, Purificacion

Institution

Munoz-Pena, Cristina. Unidad de Gestion Clinica de Laboratorio, Hospital Universitario San Agustin, Linares, Jaen, Espana. Electronic address: cristinamupe@gmail.com. Ocana-Cano, Maria Jose. Unidad de Dermatologia y Venereologia, Hospital Universitario San Agustin, Linares, Jaen, Espana.
Amores-Antequera, Carmen. Unidad de Gestion Clinica de Laboratorio, Unidad de Microbiologia, Hospital Universitario San Agustin, Linares, Jaen, Espana.
Cantudo-Munoz, Purificacion. Unidad de Gestion Clinica de Laboratorio, Unidad de Microbiologia, Hospital Universitario San Agustin, Linares, Jaen, Espana.

Emtree Heading

*Actinomycetales Infections/mi [Microbiology]. Aged. DNA, Bacterial/ip [Isolation & Purification]. Gordonia Bacterium/ge [Genetics]. *Gordonia Bacterium/ip [Isolation & Purification]. Humans. Male. Phylogeny. RNA, Ribosomal, 16S/ip [Isolation & Purification]. Sequence Analysis, DNA. *Skin Diseases, Bacterial/mi [Microbiology].

CAS Registry Numbers

0 (DNA, Bacterial). 0 (RNA, Ribosomal, 16S).

Year of Publication

2016

Cite

110.

Antibiotic resistance genes and **human** bacterial pathogens: Co-occurrence, removal, and enrichment in municipal sewage sludge digesters.

Ju F, Li B, Ma L, Wang Y, Huang D, Zhang T

Ovid MEDLINE(R) and Epub Ahead of Print, In-Process, In-Data-Review & Other Non-Indexed Citations, Daily and Versions
Water Research. 91:1-10, 2016 Mar 15.

[Journal Article. Research Support, Non-U.S. Gov't]

UI:

26773390

Understanding which/how antibiotic resistance genes (ARGs) contribute to increased acquisition of resistance by pathogens in aquatic environments are challenges of profound significance. We explored the co-occurrence and removal versus enrichment of ARGs and **human** bacterial pathogens (HBPs) in municipal sewage sludge digesters. We combined metagenomic detection of a wide spectrum of 323 ARGs and 83 HBPs with a correlation-based statistical approach and charted a network of their co-occurrence relationships. The results indicate that most ARGs and a minor proportion of HBPs (mainly Collinsella aerofaciens, Streptococcus salivarius and **Gordonia** bronchialis) could not be removed by anaerobic digestion, revealing a biological risk of post-digestion sludge in disseminating antibiotic resistance and pathogenicity. Moreover, preferential co-occurrence patterns were evident within one ARG type (e.g., multidrug, beta-lactam, and aminoglycoside) and between two different ARG types (i.e., aminoglycoside and beta-lactam), possibly implicating co-effects of antibiotic selection pressure and co-resistance on shaping antibiotic resistome in sewage sludge. Unlike beta-lactam resistance genes, ARGs of multidrug and macrolide-lincosamide-streptogramin tended to co-occur more with HBPs. Strikingly, we presented evidence that the most straightforward biological origin of an ARG-species co-occurring event is a hosting relationship. Furthermore, a significant and robust HBP-species co-occurrence correlation provides a proper scenario for nominating HBP indicators (e.g., Bifidobacterium spp. are perfect indicators of C. aerofaciens; $r = 0.92$-0.99 and P-values < 0.01). Combined, this study demonstrates a creative and effective network-based metagenomic approach for exploring ARG hosts and HBP indicators and assessing ARGs acquisition by HBPs in human-impacted environments where ARGs and HBPs may co-thrive.

Copyright © 2015 Elsevier Ltd. All rights reserved.

Version ID

1

Related Item DOI

From MEDLINE, a database of the U.S. National Library of Medicine.

Status

MEDLINE

Authors Full Name

Ju, Feng, Li, Bing, Ma, Liping, Wang, Yubo, Huang, Danping, Zhang, Tong

Institution

Ju, Feng. Environmental Biotechnology Lab, The University of Hong Kong, Hong Kong SAR, China. Li, Bing. Key Laboratory of Microorganism Application and Risk Control of Shenzhen, Graduate School at Shenzhen, Tsinghua University, China.
Ma, Liping. Environmental Biotechnology Lab, The University of Hong Kong, Hong Kong SAR, China.
Wang, Yubo. Environmental Biotechnology Lab, The University of Hong Kong, Hong Kong SAR, China.
Huang, Danping. Environmental Biotechnology Lab, The University of Hong Kong, Hong Kong SAR, China.
Zhang, Tong. Environmental Biotechnology Lab, The University of Hong Kong, Hong Kong SAR, China. Electronic address: zhangt@hku.hk.

Emtree Heading

Anti-Bacterial Agents/pd [Pharmacology]. Bacteria/de [Drug Effects]. *Bacteria/ge [Genetics]. *Bacterial Infections/mi [Microbiology]. *Drug Resistance, Bacterial/ge [Genetics]. *Genes, Bacterial. Humans. *Metagenome. Sequence Analysis, DNA. *Sewage/mi [Microbiology].

Keyword Heading

Anaerobic digesters Antibiotic resistance genes
Human bacterial pathogens
Metagenomics
Network analysis
Sewage sludge.

CAS Registry Numbers

0 (Anti-Bacterial Agents). 0 (Sewage).

Year of Publication

2016

Cite

111.
Bruker biotyper matrix-assisted laser desorption ionization-time of flight mass spectrometry system for identification of Nocardia, Rhodococcus, Kocuria, **Gordonia**, Tsukamurella, and Listeria species.

Hsueh PR, Lee TF, Du SH, Teng SH, Liao CH, Sheng WH, Teng LJ

Ovid MEDLINE(R) and Epub Ahead of Print, In-Process, In-Data-Review & Other Non-Indexed Citations, Daily and Versions
Journal of Clinical Microbiology. 52(7):2371-9, 2014 Jul.

[Evaluation Study. Journal Article]

UI:
24759706

We evaluated whether the Bruker Biotyper matrix-associated laser desorption ionization-time of flight mass spectrometry (MALDI-TOF MS) system provides accurate species-level identifications of 147 isolates of aerobically growing Gram-positive rods (GPRs). The bacterial isolates included Nocardia (n = 74), Listeria (n = 39), Kocuria (n = 15), Rhodococcus (n = 10), **Gordonia** (n = 7), and Tsukamurella (n = 2) species, which had all been identified by conventional methods, molecular methods, or both. In total, 89.7% of Listeria monocytogenes, 80% of Rhodococcus species, 26.7% of Kocuria species, and 14.9% of Nocardia species (n = 11, all N. nova and N. otitidiscaviarum) were correctly identified to the species level (score values, >= 2.0). A clustering analysis of spectra generated by the Bruker Biotyper identified six clusters of Nocardia species, i.e., cluster 1 (N. cyriacigeorgica), cluster 2 (N. brasiliensis), cluster 3 (N. farcinica), cluster 4 (N. puris), cluster 5 (N. asiatica), and cluster 6 (N. beijingensis), based on the six peaks generated by ClinProTools with the genetic algorithm, i.e., m/z 2,774.477 (cluster 1), m/z 5,389.792 (cluster 2), m/z 6,505.720 (cluster 3), m/z 5,428.795 (cluster 4), m/z 6,525.326 (cluster 5), and m/z 16,085.216 (cluster 6). Two clusters of L. monocytogenes spectra were also found according to the five peaks, i.e., m/z 5,594.85, m/z 6,184.39, and m/z 11,187.31, for cluster 1 (serotype 1/2a) and m/z 5,601.21 and m/z 11,199.33 for cluster 2 (serotypes 1/2b and 4b). The Bruker Biotyper system was unable to accurately identify Nocardia (except for N. nova and N. otitidiscaviarum), Tsukamurella, or **Gordonia** species. Continuous expansion of the MALDI-TOF MS databases to include more GPRs is necessary.

Copyright © 2014, American Society for Microbiology. All Rights Reserved.

Version ID

1

Related Item DOI

From MEDLINE, a database of the U.S. National Library of Medicine.

Status

MEDLINE

Authors Full Name

Hsueh, Po-Ren, Lee, Tai-Fen, Du, Shin-Hei, Teng, Shih-Hua, Liao, Chun-Hsing, Sheng, Wang-Hui, Teng, Lee-Jene

Institution

Hsueh, Po-Ren. Department of Laboratory Medicine, National Taiwan University Hospital, National Taiwan University College of Medicine, Taipei, Taiwan Department of Internal Medicine, National Taiwan University Hospital, National Taiwan University College of Medicine, Taipei, Taiwan hsporen@ntu.edu.tw. Lee, Tai-Fen. Department of Laboratory Medicine, National Taiwan University Hospital, National Taiwan University College of Medicine, Taipei, Taiwan Department and Graduate Institute of Clinical Laboratory Sciences and Medical Biotechnology, National Taiwan University, Taipei, Taiwan.
Du, Shin-Hei. Department of Laboratory Medicine, National Taiwan University Hospital, National Taiwan University College of Medicine, Taipei, Taiwan.
Teng, Shih-Hua. Department of Graduate Institute of Biomedical Sciences, Chang Gung University, Tao-Yuan, Taiwan Bruker Taiwan Co., Ltd., Taipei, Taiwan.
Liao, Chun-Hsing. Department of Internal Medicine, Far Eastern Memorial Hospital, Taipei, Taiwan.
Sheng, Wang-Hui. Department of Internal Medicine, National Taiwan University Hospital, National Taiwan University College of Medicine, Taipei, Taiwan.

Teng, Lee-Jene. Department of Laboratory Medicine, National Taiwan University Hospital, National Taiwan University College of Medicine, Taipei, Taiwan Department and Graduate Institute of Clinical Laboratory Sciences and Medical Biotechnology, National Taiwan University, Taipei, Taiwan.

PMID

https://www.ncbi.nlm.nih.gov/pmc/articles/PMC4097692

Emtree Heading

Actinomycetales/ch [Chemistry]. *Actinomycetales/cl [Classification]. Actinomycetales/ip [Isolation & Purification]. *Actinomycetales Infections/di [Diagnosis]. Actinomycetales Infections/mi [Microbiology]. Bacteria, Aerobic/ch [Chemistry]. Bacteria, Aerobic/cl [Classification]. Bacteria, Aerobic/ip [Isolation & Purification]. *Bacteriological Techniques/mt [Methods]. Cluster Analysis. Gram-Positive Rods/ch [Chemistry]. Gram-Positive Rods/cl [Classification]. Gram-Positive Rods/ip [Isolation & Purification]. Humans. Listeria/ch [Chemistry]. *Listeria/cl [Classification]. Listeria/ip [Isolation & Purification]. *Listeriosis/di [Diagnosis]. Listeriosis/mi [Microbiology]. Sensitivity and Specificity. *Spectrometry, Mass, Matrix-Assisted Laser Desorption-Ionization/mt [Methods].

Year of Publication

2014

Cite

112.
[Subacute sternal osteomyelitis caused by **Gordonia** bronchialis after open-heart surgery]. [Spanish]
Osteomielitis esternal subaguda por Gordonia bronchialis tras cirugia cardiaca.
<Osteomielitis esternal subaguda por
Gordonia
bronchialis tras cirugia cardiaca.>

Vasquez MA, Marne C, Villuendas MC, Arazo P

Ovid MEDLINE(R) and Epub Ahead of Print, In-Process, In-Data-Review & Other Non-Indexed Citations, Daily and Versions
Enfermedades Infecciosas y Microbiologia Clinica. 31(8):559-60, 2013 Oct.

[Case Reports. Letter]

UI:
23587704

Version ID

1

Related Item DOI

From MEDLINE, a database of the U.S. National Library of Medicine.

Status

MEDLINE

Authors Full Name

Vasquez, Maria Alejandra, Marne, Carmen, Villuendas, Maria Cruz, Arazo, Piedad

Institution

Vasquez, Maria Alejandra. Servicio de Microbiologia, IIS Aragon, Hospital Universitario Miguel Servet, Zaragoza, Espana.

Emtree Heading

Actinomycetales Infections/di [Diagnosis]. Actinomycetales Infections/dt [Drug Therapy]. *Actinomycetales Infections/mi [Microbiology]. Actinomycetales Infections/su [Surgery]. Aged. Anti-Bacterial Agents/pd [Pharmacology]. Anti-Bacterial Agents/tu [Therapeutic Use]. *Coronary Artery Bypass. Cutaneous Fistula/et [Etiology]. Debridement. Diagnosis, Differential. Exudates and Transudates/mi [Microbiology]. Female. Gordonia Bacterium/de [Drug Effects]. *Gordonia Bacterium/ip [Isolation & Purification]. Humans. Microbial Sensitivity Tests. Nocardia Infections/di [Diagnosis]. Osteomyelitis/dt [Drug Therapy]. *Osteomyelitis/mi [Microbiology]. Osteomyelitis/su [Surgery]. Spectrometry, Mass, Matrix-Assisted Laser Desorption-Ionization. *Sternotomy/ae [Adverse Effects]. *Sternum/mi [Microbiology]. Sternum/su [Surgery]. Surgical Wound Infection/dt [Drug Therapy]. *Surgical Wound Infection/mi [Microbiology]. Surgical Wound Infection/su [Surgery].

CAS Registry Numbers

0 (Anti-Bacterial Agents).

Year of Publication

2013

Cite

113.

The strengths and weaknesses of **Gordonia**: a review of an emerging genus with increasing biotechnological potential. [Review]

Drzyzga O

Ovid MEDLINE(R) and Epub Ahead of Print, In-Process, In-Data-Review & Other Non-Indexed Citations, Daily and Versions
Critical Reviews in Microbiology. 38(4):300-16, 2012 Nov.

[Journal Article. Review]

UI:
22551505

This review about the genus **Gordonia** provides a current overview of recent research on a young genus that was introduced in the year 1997 (Stackebrandt et al., 1997). This emerging genus has attracted increasing environmental, industrial, biotechnological and medical interest during the last few years, in particular due to the capabilities of its members to degrade, transform, and synthesize organic compounds as well as to the pathogenic effects that have been described in many case studies. The number of publications about **Gordonia** has increased significantly after the year 2004 (the year of the first **Gordonia** review published by Arenskotter et al.) describing 13 new validly published species (type strains), many newly described physiological and metabolic capabilities, new patent applications and many new case reports of bacterial infections. Members of the genus **Gordonia** are widely distributed in nature and it is therefore important to unravel the species richness and metabolic potential of gordoniae in future studies to demonstrate their environmental impact especially on the degradation of persistent organic compounds and their ecological participation in the carbon cycle of organic material in soil and water. This review summarizes mainly the current state of importance and potential of the members of this genus for the environmental and biotechnological industry ("the strengthsa) and briefly its pathogenic impact to humans ("the weaknessesa).

Version ID

1

Related Item DOI

From MEDLINE, a database of the U.S. National Library of Medicine.

Status

MEDLINE

Authors Full Name

Drzyzga, Oliver

Institution

Drzyzga, Oliver. Department of Biochemistry and Molecular Biology I, Complutense University of Madrid, Madrid, Spain. drzyzga@bio.ucm.es

Emtree Heading

*Actinomycetales Infections/mi [Microbiology]. *Biotechnology. Environmental Pollutants/me [Metabolism]. Gordonia Bacterium/cl [Classification]. Gordonia Bacterium/ge [Genetics]. *Gordonia Bacterium/me [Metabolism]. *Gordonia Bacterium/py [Pathogenicity]. Humans.

CAS Registry Numbers

0 (Environmental Pollutants).

Year of Publication

2012

Cite

114.
Gordonia terrae: a difficult-to-diagnose emerging pathogen?.

Blanc V, Dalle M, Markarian A, Debunne MV, Duplay E, Rodriguez-Nava V, Boiron P

Ovid MEDLINE(R) and Epub Ahead of Print, In-Process, In-Data-Review & Other Non-Indexed Citations, Daily and Versions
Journal of Clinical Microbiology. 45(3):1076-7, 2007 Mar.

[Case Reports. Comment. Letter]

UI:
17192419

Version ID

1

Related Item DOI

From MEDLINE, a database of the U.S. National Library of Medicine.

Status

MEDLINE

Authors Full Name

Blanc, V, Dalle, M, Markarian, A, Debunne, M V, Duplay, E, Rodriguez-Nava, V, Boiron, P

Comments

Comment on (CON)

PMID

https://www.ncbi.nlm.nih.gov/pmc/articles/PMC1829133

Emtree Heading

Actinomycetales Infections/di [Diagnosis]. Actinomycetales Infections/mi [Microbiology]. *Actinomycetales Infections. Adult. Bacterial Typing Techniques. DNA, Bacterial/an [Analysis]. DNA, Ribosomal/an [Analysis]. Eyelid Diseases/di [Diagnosis]. Eyelid Diseases/mi [Microbiology]. *Eyelids/mi [Microbiology]. Female. Gordonia Bacterium/cl [Classification]. Gordonia Bacterium/ge [Genetics]. Gordonia Bacterium/ip [Isolation & Purification]. *Gordonia Bacterium. Humans. RNA, Ribosomal, 16S/ge [Genetics]. Sequence Analysis, DNA.

CAS Registry Numbers

0 (DNA, Bacterial). 0 (DNA, Ribosomal). 0 (RNA, Ribosomal, 16S).

Year of Publication

2007

Cite

115.
[Evaluation of API Coryne System, version 2.0, for diphteroid gram-positive rods identification with clinical relevance]. [Spanish]
Evaluacion del sistema API coryne, version 2.0, para la identificacion de bacilos gram-positivos difteroides de importancia clinica.
<Evaluacion del sistema API coryne, version 2.0, para la identificacion de bacilos gram-positivos difteroides de importancia clinica.>

Almuzara MN, De Mier C, Rodriguez CR, Famiglietti AM, Vay CA

Ovid MEDLINE(R) and Epub Ahead of Print, In-Process, In-Data-Review & Other Non-Indexed Citations, Daily and Versions
Revista Argentina de Microbiologia. 38(4):197-201, 2006 Oct-Dec.

[Evaluation Study. Journal Article. Research Support, Non-U.S. Gov't]

UI:
17370571

The ability of the API Coryne system, version 2.0, to identify 178 strains of gram-positive rods was evaluated. Seventy eight isolates belonged to genus Corynebacterium and one hundred to related genera, all strains were isolated from clinical samples at the Laboratory of Bacteriology, Hospital de Clinicas Jose de San Martin (UBA) between 1995 and 2004. The isolates were identified according to von Graevenitz and Funke's scheme. One hundred and sixty two out of 178 strains (91%) were correctly identified at genus and species level (IC95 = 85.6-94.6), in 44 of them (24.7%) additional tests were needed to final identification. Sixteen strains (9%) were not correctly identified (IC95 = 5.4-14.4); none of the 178 strains remained unidentified. The API Coryne system, version 2.0, is useful to identify the majority of Corynebacterium species with clinical relevance: Corynebacterium jeikeium, Corynebacterium urealyticum, Corynebacterium striatum, Corynebacterium pseudodiphtheriticum, Corynebacterium amycolatum and related species such as Arcanobacterium haemolyticum, Dermabacter hominis, Listeria monocytogenes, among others. Nevertheless for yellow-pigmented diphteroid gram-positive rods (Aureobacterium spp., Leifsonia aquatica, Microbacterium spp. and Cellulomonas spp.) and for acid fast gram-positive rods (Rhodococcus, **Gordonia**, Tsukamurella and Nocardia) the identification usefulness the system is limited.

Version ID

1

Related Item DOI

From MEDLINE, a database of the U.S. National Library of Medicine.

Status

MEDLINE

Authors Full Name

Almuzara, M N, De Mier, C, Rodriguez, C R, Famiglietti, A M R, Vay, C A

Institution

Almuzara, M N. Laboratorio de Bacteriologia, Departamento de Bioqufmica Clinica, Hospital de Clinicas Jose de San Martin, Facultad de Farmacia y Bioquimica, Universidad de Buenos Aires. marisaalmuzara@arnet.com.ar

Emtree Heading

Argentina. Bacterial Typing Techniques/is [Instrumentation]. *Bacterial Typing Techniques/mt [Methods]. Catalase. Corynebacterium/cl [Classification]. Corynebacterium/ip [Isolation & Purification]. Corynebacterium/me [Metabolism]. Corynebacterium Infections/mi [Microbiology]. *Gram-Positive Asporogenous Rods/cl [Classification]. Gram-Positive Asporogenous Rods/ip [Isolation & Purification]. Gram-Positive Asporogenous Rods/me [Metabolism]. Humans. Listeria monocytogenes/ip [Isolation & Purification]. Listeria monocytogenes/me [Metabolism]. Rhodococcus/cl [Classification]. Rhodococcus/ip [Isolation & Purification]. Rhodococcus/me [Metabolism]. Species Specificity. Staining and Labeling.

CAS Registry Numbers

EC 1-11-1-6 (Catalase).

Year of Publication

2006

Cite

116.

Gordonia araii sp. nov. and **Gordonia** effusa sp. nov., isolated from patients in Japan.

Kageyama A, Iida S, Yazawa K, Kudo T, Suzuki SI, Koga T, Saito H, Inagawa H, Wada A, Kroppenstedt RM, Mikami Y

Ovid MEDLINE(R) and Epub Ahead of Print, In-Process, In-Data-Review & Other Non-Indexed Citations, Daily and Versions
International Journal of Systematic & Evolutionary Microbiology. 56(Pt 8):1817-1821, 2006 Aug.

[Journal Article]

UI:
16902014

Two bacterial strains, IFM 10211(T) and IFM 10200(T), were isolated from the sputum of two Japanese patients, and were subjected to a polyphasic taxonomic study. The two strains were found to have morphological, physiological and chemotaxonomic properties that were consistent with their assignment to the genus **Gordonia**, except for a few chemotaxonomic characteristics. Almost complete 16S rRNA gene sequences of the two strains were determined; the data showed that they are related distantly to **Gordonia** amarae, **Gordonia** hirsuta, **Gordonia** hydrophobica and **Gordonia** sihwensis, showing 16S rRNA gene sequence similarities to the type strains of these species of 96.2-97.9 %. DNA-DNA relatedness data coupled with the combination of genotypic and phenotypic data indicated that the two strains are representatives of two novel, separate species. The names proposed to accommodate these two strains are **Gordonia** araii sp. nov. (type strain IFM 10211(T)=DSM 44811(T)=NBRC 100433(T)=JCM 12131(T)) and **Gordonia** effusa sp. nov. (type strain IFM 10200(T)=DSM 44810(T)=NBRC 100432(T)=JCM 12130(T)).

Version ID

1

Related Item DOI

From MEDLINE, a database of the U.S. National Library of Medicine.

Status

MEDLINE

Authors Full Name

Kageyama, Akiko, Iida, Soji, Yazawa, Katsukiyo, Kudo, Takuji, Suzuki, Shin-Ichi, Koga, Takeharu, Saito, Hiromi, Inagawa, Hiroko, Wada, Akihito, Kroppenstedt, Reiner M, Mikami, Yuzuru

Institution

Kageyama, Akiko. Research Center for Pathogenic Fungi and Microbial Toxicoses, Chiba University, 1-8-1 Inohana, Chuo-ku, Chiba 260-8673, Japan. Iida, Soji. Research Center for Pathogenic Fungi and Microbial Toxicoses, Chiba University, 1-8-1 Inohana, Chuo-ku, Chiba 260-8673, Japan.
Yazawa, Katsukiyo. Research Center for Pathogenic Fungi and Microbial Toxicoses, Chiba University, 1-8-1 Inohana, Chuo-ku, Chiba 260-8673, Japan.
Kudo, Takuji. Japan Collection of Microorganisms, RIKEN BioResource Center, Wako, Saitama 351-0198, Japan.
Suzuki, Shin-Ichi. Discovery Research Laboratories, Tanabe Seiyaku Co. Ltd, 2-2-50 Kawagishi, Toda, Saitama 335-8505, Japan.
Koga, Takeharu. First Department of Internal Medicine, Kurume University School of Medicine, 67 Asahimachi, Kurume 830-0011, Japan.
Saito, Hiromi. Laboratory of Clinical Microbiology, Toranomon Hospital, Toranomon 2-2-2, Minato-ku, Tokyo 105-8470, Japan.
Inagawa, Hiroko. Laboratory of Clinical Microbiology, Toranomon Hospital, Toranomon 2-2-2, Minato-ku, Tokyo 105-8470, Japan.
Wada, Akihito. National Institute of Infectious Diseases, Toyama, Shinjuku-ku, Tokyo 162-8640, Japan.
Kroppenstedt, Reiner M. Deutsche Sammlung von Mikroorganismen und Zellkulturen, Braunschweig, Germany.
Mikami, Yuzuru. Research Center for Pathogenic Fungi and Microbial Toxicoses, Chiba University, 1-8-1 Inohana, Chuo-ku, Chiba 260-8673, Japan.

Emtree Heading

Aged. Base Composition. Corynebacterium/ch [Chemistry]. *Corynebacterium/cl [Classification]. Corynebacterium/ph [Physiology]. Corynebacterium Infections/co [Complications]. Corynebacterium Infections/mi [Microbiology]. DNA, Bacterial/ch [Chemistry]. DNA, Bacterial/ge [Genetics]. Humans. Japan. Kidney Diseases/co [Complications]. Male. Middle Aged. Molecular Sequence Data. Phenotype. Pneumonia, Bacterial/mi [Microbiology]. RNA, Bacterial/ge [Genetics]. RNA, Ribosomal, 16S/ge [Genetics]. Sequence Homology, Nucleic Acid. Species Specificity. Sputum/mi [Microbiology].

CAS Registry Numbers

0 (DNA, Bacterial). 0 (RNA, Bacterial). 0 (RNA, Ribosomal, 16S).

Year of Publication

2006

Cite

117.

Gordonia otitidis sp. nov., isolated from a patient with external otitis.

Iida S, Taniguchi H, Kageyama A, Yazawa K, Chibana H, Murata S, Nomura F, Kroppenstedt RM, Mikami Y

Ovid MEDLINE(R) and Epub Ahead of Print, In-Process, In-Data-Review & Other Non-Indexed Citations, Daily and Versions
International Journal of Systematic & Evolutionary Microbiology. 55(Pt 5):1871-1876, 2005 Sep.

[Journal Article]

UI:
16166681

The taxonomic positions of two clinically isolated actinomycetes were established using a polyphasic approach. The two strains, IFM 10032(T), isolated from ear discharge of a 28-year-old Japanese female patient with external otitis, and IFM 10148, isolated from pleural fluid of a 60-year-old Japanese male patient with bronchitis, possessed meso-diaminopimelic acid as the diagnostic amino acid, MK-9(H(2)) as the predominant menaquinone and mycolic acids ranging from 58 to 64 carbons. The 16S rRNA gene sequences of the two strains were most closely related to those of **Gordonia** aichiensis, **Gordonia** sputi and 'Gordonia jacobaea'. Differences in several phenotypic characteristics together with genotypic distinctiveness distinguish strains IFM 10032(T) and IFM 10148 from these three species. DNA-DNA hybridization results and the combination of genotypic and phenotypic data showed that the two strains belong to a single species, and merit recognition of a novel species within the genus **Gordonia**. The name proposed for this taxon is **Gordonia** otitidis sp. nov.; the type strain is IFM 10032(T) (=DSM 44809(T)=JCM 12355(T)=NBRC 100426(T)).

Version ID

1

Related Item DOI

From MEDLINE, a database of the U.S. National Library of Medicine.

Status

MEDLINE

Authors Full Name

Iida, Soji, Taniguchi, Hiroko, Kageyama, Akiko, Yazawa, Katsukiyo, Chibana, Hiroji, Murata, Shota, Nomura, Fumio, Kroppenstedt, Reiner M, Mikami, Yuzuru

Institution

Iida, Soji. Research Center for Pathogenic Fungi and Microbial Toxicoses, Chiba University, 1-8-1, Inohana, Chuo-ku, Chiba 260-8673, Japan. Taniguchi, Hiroko. Research Center for Pathogenic Fungi and Microbial Toxicoses, Chiba University, 1-8-1, Inohana, Chuo-ku, Chiba 260-8673, Japan.
Kageyama, Akiko. Research Center for Pathogenic Fungi and Microbial Toxicoses, Chiba University, 1-8-1, Inohana, Chuo-ku, Chiba 260-8673, Japan.
Yazawa, Katsukiyo. Research Center for Pathogenic Fungi and Microbial Toxicoses, Chiba University, 1-8-1, Inohana, Chuo-ku, Chiba 260-8673, Japan.
Chibana, Hiroji. Research Center for Pathogenic Fungi and Microbial Toxicoses, Chiba University, 1-8-1, Inohana, Chuo-ku, Chiba 260-8673, Japan.
Murata, Shota. Clinical Laboratory Chiba University Hospital, Chiba University, 1-8-1, Inohana, Chuo-ku, Chiba 260-8673, Japan.
Nomura, Fumio. Department of Laboratory Medicine, Chiba University School of Medicine, Chiba University, 1-8-1, Inohana, Chuo-ku, Chiba 260-8673, Japan.
Kroppenstedt, Reiner M. Deutsche Sammlung von Mikroorganismen und Zellkulturen, Braunschweig, Germany.
Mikami, Yuzuru. Research Center for Pathogenic Fungi and Microbial Toxicoses, Chiba University, 1-8-1, Inohana, Chuo-ku, Chiba 260-8673, Japan.

Emtree Heading

*Actinomycetales Infections/mi [Microbiology]. Adult. *Bronchitis/mi [Microbiology]. DNA, Bacterial. Female. Genes, rRNA. Genotype. Gordonia Bacterium/ch [Chemistry]. *Gordonia Bacterium/cl [Classification]. Gordonia Bacterium/ge [Genetics]. Gordonia Bacterium/ip [Isolation & Purification]. Humans. Male. Middle Aged. Molecular Sequence Data. Nucleic Acid Hybridization. *Otitis Externa/mi [Microbiology]. Phenotype. Phylogeny. *Pleural Effusion/mi [Microbiology]. RNA, Ribosomal, 16S.

CAS Registry Numbers

0 (DNA, Bacterial). 0 (RNA, Ribosomal, 16S).

Year of Publication

2005

Cite

118.
Biology of the metabolically diverse genus **Gordonia**. [Review] [95 refs]

Arenskotter M, Broker D, Steinbuchel A

Ovid MEDLINE(R) and Epub Ahead of Print, In-Process, In-Data-Review & Other Non-Indexed Citations, Daily and Versions
Applied & Environmental Microbiology. 70(6):3195-204, 2004 Jun.

[Journal Article. Research Support, Non-U.S. Gov't. Review]

UI:
15184112

Version ID

1

Related Item DOI

From MEDLINE, a database of the U.S. National Library of Medicine.

Status

MEDLINE

Authors Full Name

Arenskotter, Matthias, Broker, Daniel, Steinbuchel, Alexander

Institution

Arenskotter, Matthias. Institut fur Molekulare Mikrobiologie und Biotechnologie, Westfalische Wilhelms-Universitat Munster, Muenster, Germany.

PMID

https://www.ncbi.nlm.nih.gov/pmc/articles/PMC427784

Emtree Heading

Actinomycetales Infections/mi [Microbiology]. Base Sequence. Biotechnology. Environmental Microbiology. *Genetic Variation. Gordonia Bacterium/cl [Classification]. Gordonia Bacterium/ge [Genetics]. Gordonia Bacterium/me [Metabolism]. Gordonia Bacterium/py [Pathogenicity]. *Gordonia Bacterium. Humans. Industrial Microbiology. Molecular Sequence Data.

Year of Publication

2004

Cite

119.
Mycetoma of the hand caused by Gordona terrae: a case report.

Bakker XR, Spauwen PH, Dolmans WM

Ovid MEDLINE(R) and Epub Ahead of Print, In-Process, In-Data-Review & Other Non-Indexed Citations, Daily and Versions
Journal of Hand Surgery - British Volume. 29(2):188-90, 2004 Apr.

[Case Reports. Journal Article]

UI:
15010171

Version ID

1

Related Item DOI

From MEDLINE, a database of the U.S. National Library of Medicine.

Status

MEDLINE

Authors Full Name

Bakker, X R, Spauwen, P H M, Dolmans, W M V

Institution

Bakker, X R. Departments of Plastic Surgery and Tropical Medicine, University Medical Centre Nijmegen, Nijmegen, The Netherlands. xrbakker@planet.nl

Emtree Heading

*Actinomycetales Infections/di [Diagnosis]. Actinomycetales Infections/su [Surgery]. Adolescent. *Gordonia Bacterium/ip [Isolation & Purification]. Granuloma/mi [Microbiology]. Granuloma/su [Surgery]. *Hand/mi [Microbiology]. Hand/su [Surgery]. Humans. Male. *Mycetoma/di [Diagnosis]. Mycetoma/su [Surgery].

Year of Publication

2004

Cite

120.

Novel diagnostic algorithm for identification of mycobacteria using genus-specific amplification of the 16S-23S rRNA gene spacer and restriction endonucleases.

Roth A, Reischl U, Streubel A, Naumann L, Kroppenstedt RM, Habicht M, Fischer M, Mauch H

Ovid MEDLINE(R) and Epub Ahead of Print, In-Process, In-Data-Review & Other Non-Indexed Citations, Daily and Versions
Journal of Clinical Microbiology. 38(3):1094-104, 2000 Mar.

[Journal Article]

UI:
10699003

A novel genus-specific PCR for mycobacteria with simple identification to the species level by restriction fragment length polymorphism (RFLP) was established using the 16S-23S ribosomal RNA gene (rDNA) spacer as a target. Panspecificity of primers was demonstrated on the genus level by testing 811 bacterial strains (122 species in 37 genera from 286 reference strains and 525 clinical isolates). All mycobacterial isolates (678 strains among 48 defined species and 5 indeterminate taxons) were amplified by the new primers. Among nonmycobacterial isolates, only **Gordonia** terrae was amplified. The RFLP scheme devised involves estimation of variable PCR product sizes together with HaeIII and CfoI restriction analysis. It yielded 58 HaeIII patterns, of which 49 (84%) were unique on the species level. Hence, HaeIII digestion together with CfoI results was sufficient for correct identification of 39 of 54 mycobacterial taxons and one of three or four of seven RFLP genotypes found in Mycobacterium intracellulare and Mycobacterium kansasii, respectively. Following a clearly laid out diagnostic algorithm, the remaining unidentified organisms fell into five clusters of closely related species (i.e., the Mycobacterium avium complex or Mycobacterium chelonae-Mycobacterium abscessus) that were successfully separated using additional enzymes (TaqI, MspI, DdeI, or AvaII). Thus, next to slowly growing mycobacteria, all rapidly growing species studied, including M. abscessus, M. chelonae, Mycobacterium farcinogenes, Mycobacterium fortuitum, Mycobacterium peregrinum, and Mycobacterium senegalense (with a very high 16S rDNA sequence similarity) were correctly identified. A high intraspecies sequence stability and the good discriminative power of patterns indicate that this method is very suitable for rapid and cost-effective identification of a wide variety of mycobacterial species without the need for sequencing. Phylogenetically, spacer sequence data stand in good agreement with 16S rDNA sequencing results, as was shown by including strains with unsettled taxonomy. Since this approach recognized significant subspecific genotypes while identification of a broad spectrum of mycobacteria rested on identification of one specific RFLP pattern within a species, this method can be used by both reference (or research) and routine laboratories.

Version ID

1

Related Item DOI

From MEDLINE, a database of the U.S. National Library of Medicine.

Status

MEDLINE

Authors Full Name

Roth, A, Reischl, U, Streubel, A, Naumann, L, Kroppenstedt, R M, Habicht, M, Fischer, M, Mauch, H

Institution

Roth, A. Institut fur Mikrobiologie und Immunologie, Lungenklinik Heckeshorn, 14109 Berlin, Germany. mikromau@zedat.fu-berlin.de

PMID

https://www.ncbi.nlm.nih.gov/pmc/articles/PMC86348

Emtree Heading

Algorithms. Base Sequence. Conserved Sequence. DNA Primers. DNA, Bacterial/ge [Genetics]. *DNA, Ribosomal/ge [Genetics]. Deoxyribonucleases, Type II Site-Specific. Humans. Molecular Sequence Data. Mycobacterium/cl [Classification]. *Mycobacterium/ge [Genetics]. *Mycobacterium/ip [Isolation & Purification]. *Mycobacterium Infections/di [Diagnosis]. Mycobacterium Infections/mi [Microbiology]. Mycobacterium avium Complex/cl [Classification]. Mycobacterium avium Complex/ge [Genetics]. Mycobacterium avium Complex/ip [Isolation & Purification]. Polymorphism, Restriction Fragment Length. RNA, Bacterial/ge [Genetics]. *RNA, Ribosomal, 16S/ge [Genetics]. *RNA, Ribosomal, 23S/ge [Genetics]. Restriction Mapping. Sequence Alignment. Sequence Homology, Nucleic Acid.

CAS Registry Numbers

0 (DNA Primers). 0 (DNA, Bacterial). 0 (DNA, Ribosomal). 0 (RNA, Bacterial). 0 (RNA, Ribosomal, 16S). 0 (RNA, Ribosomal, 23S). EC 3-1-21-4 (Deoxyribonucleases, Type II Site-Specific). EC 3-1-21-4 (GCGC-specific type II deoxyribonucleases). EC 3-1-21-4 (GGCC-specific type II deoxyribonucleases).

Year of Publication

2000

Cite
